# Iron‐Electrocatalyzed C−H Arylations: Mechanistic Insights into Oxidation‐Induced Reductive Elimination for Ferraelectrocatalysis

**DOI:** 10.1002/chem.201904018

**Published:** 2019-12-09

**Authors:** Cuiju Zhu, Maximilian Stangier, João C. A. Oliveira, Leonardo Massignan, Lutz Ackermann

**Affiliations:** ^1^ Institut für Organische und Biomolekulare Chemie Georg-August-Universität Göttingen Tammannstraße 2 37077 Göttingen Germany; ^2^ Woehler Research Institute for Sustainable Chemistry (WISCh) Georg-August-Universität Göttingen Tammannstraße 2 37077 Göttingen Germany

**Keywords:** arylation, C−H activation, electricity, electrocatalysis, iron, sustainability

## Abstract

Despite major advances, organometallic C−H transformations are dominated by precious 5d and 4d transition metals, such as iridium, palladium and rhodium. In contrast, the unique potential of less toxic Earth‐abundant 3d metals has been underexplored. While iron is the most naturally abundant transition metal, its use in oxidative, organometallic C−H activation has faced major limitations due to the need for superstoichiometric amounts of corrosive, cost‐intensive DCIB as the sacrificial oxidant. To fully address these restrictions, we describe herein the unprecedented merger of electrosynthesis with iron‐catalyzed C−H activation through oxidation‐induced reductive elimination. Thus, ferra‐ and manganaelectro‐catalyzed C−H arylations were accomplished at mild reaction temperatures with ample scope by the action of sustainable iron catalysts, employing electricity as a benign oxidant.

## Graphical abstract

**Electro with Ironman**: Electricity‐enabled resource‐economic C−H activations by iron and manganese catalysis, avoiding precious metals and toxic chemical oxidants.

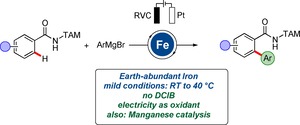

C−H activation has surfaced as an increasingly powerful tool for molecular engineering,^1^ with transformative applications throughout the material sciences,^2^ natural product syntheses,^3^ late‐stage diversification,^4^ and have also been used on pharmaceutical industrial scales.^5^ In particular, arylations of otherwise inert C−H bonds have proven instrumental as a step‐economical alternative to the Nobel Prize winning palladium‐catalyzed cross‐couplings,^6^ avoiding lengthy prefunctionalization protocols and thereby preventing undesired waste formation.^7^ While these C−H activations have thus far been dominated by rare and toxic 4d transition metals (Figure 1 a), considerable recent impetus was gained by identifying viable catalysts based on Earth‐abundant^8^ 3d base metals.^9^ In particular, inexpensive iron catalysis has gained considerable recent momentum due to its non‐toxic nature (Figure 1 b),^10^ with major potential for translational applications on scale, particularly when considering trace metal impurities. Despite these major advances, all documented iron‐catalyzed C−H arylations continue to be strongly limited by the need for superstoichiometric quantities of dichloroisobutane (DCIB) as the sacrificial oxidant, while simple vicinal dihalides or other chemical oxidants are generally not effective in iron‐catalyzed C−H activations.10c Unfortunately, DCIB^11^ is elusive on commercial scale, features considerable safety hazards, generates overstoichiometric amounts of corrosive by‐products, which overall significantly deteriorates the environmental footprint of oxidative iron catalysis. Importantly, DCIB is characterized by costs that are comparable to those of the typical noble transition metal catalyst Pd(OAc)_2_ (Figure 1 c), hence jeopardizing the inherent sustainable nature of the iron‐catalyzed C−H activation approach. As a significantly more sustainable alternative, we have now devised a strategy for the unprecedented DCIB‐free, iron‐catalyzed C−H arylation through the action of user‐friendly electricity^12^ as environmentally benign oxidant. Salient features of our findings include (a) first electrochemical iron‐catalyzed C−H activation, (b) electrolysis devoid of toxic chemical oxidants, and (c) versatility by iron‐ or manganese‐electrocatalysis, which were guided by (d) detailed experimental and computational mechanistic insights into an as of yet elusive electrooxidative iron(II/III/I) regime, (d) spin‐crossover for iron‐electrocatalysis, and (e) manganese electrocatalysis for direct arylations (Figure 1 d). Thus, the present study provides a proof‐of‐concept for illustrating that cost‐intensive chemical halide oxidants can be replaced by user‐friendly electricity in a “low‐valent” metal catalysis, highlighting iron‐catalyzed10n C−H arylations (Figure 1 e). In terms of cost of goods, viable trace metal impurities in biorelevant drugs and inherent metal toxicities,^13^ iron as the most Earth‐abundant transition metal compares favourably with all other previously explored transition metals for electrocatalytic strong bond activation, including cobalt,^14^ copper,^15^ manganese,^16^ and nickel.^17^

**Figure 1:**
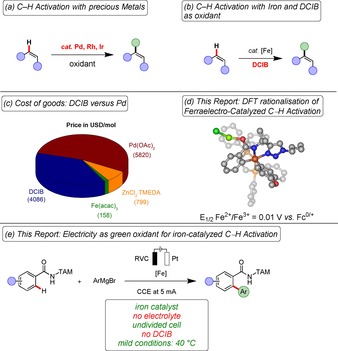
Strategies for C−H arylation. (a) Precious metal catalyzed C−H arylation. (b) Iron‐catalyzed C−H activation with DCIB as oxidant. (c) Cost of goods: DCIB is as cost‐intensive as is Pd(OAc)_2_. (d) Computed oxidation potential. (e) This report: Electricity as oxidant for iron‐catalyzed C−H activation.

At the outset of our studies we explored the oxidation potential of the putative iron(II/III) manifold by means of computation (Figure 2 a and the Supporting Information).^18^ Our findings were indicative of an iron(II) active catalyst, being supported by Mössbauer spectroscopy studies,^19^ revealing a viable redox event at *E*_1/2_=0.01 V versus Fc^0/+^.^18^ The latter could be rationalized by the oxidation of bimetallic magnesium‐iron complex, S‐3′′, generated through transmetalation followed by single‐electron‐transfer. With these computational insights in hand, we set out to identify viable reaction conditions for the elusive electrooxidative iron‐catalyzed C−H arylation of TAM‐benzamide **1** bearing a peptide‐isosteric click‐triazole^20^ in a user‐friendly undivided cell setup (Figure 2 b). After considerable preliminary experimentation, we observed that the desired electrochemical C−H arylation product **3** was obtained at an exceedingly mild reaction temperature of 40 °C, when using a RVC anode, along with a platinum cathode. Notably, the electrochemical C−H activation was even operative at room temperature, reflecting the outstanding performance of the electrocatalysis manifold. Among a representative set of iron sources, Fe(acac)_3_ was found to be optimal.^18^ Control experiments confirmed the essential role of the electricity, the iron catalyst and the additive. The iron‐catalyzed electrooxidative C−H arylation proved likewise viable in the biomass‐derived^21^ solvent 2‐MeTHF,^18^ further substantiating the sustainable nature of our electrocatalysis. The ferraelectrocatalysis was also conveniently conducted with commercially available equipment, mirroring its user‐friendly nature.

**Figure 2:**
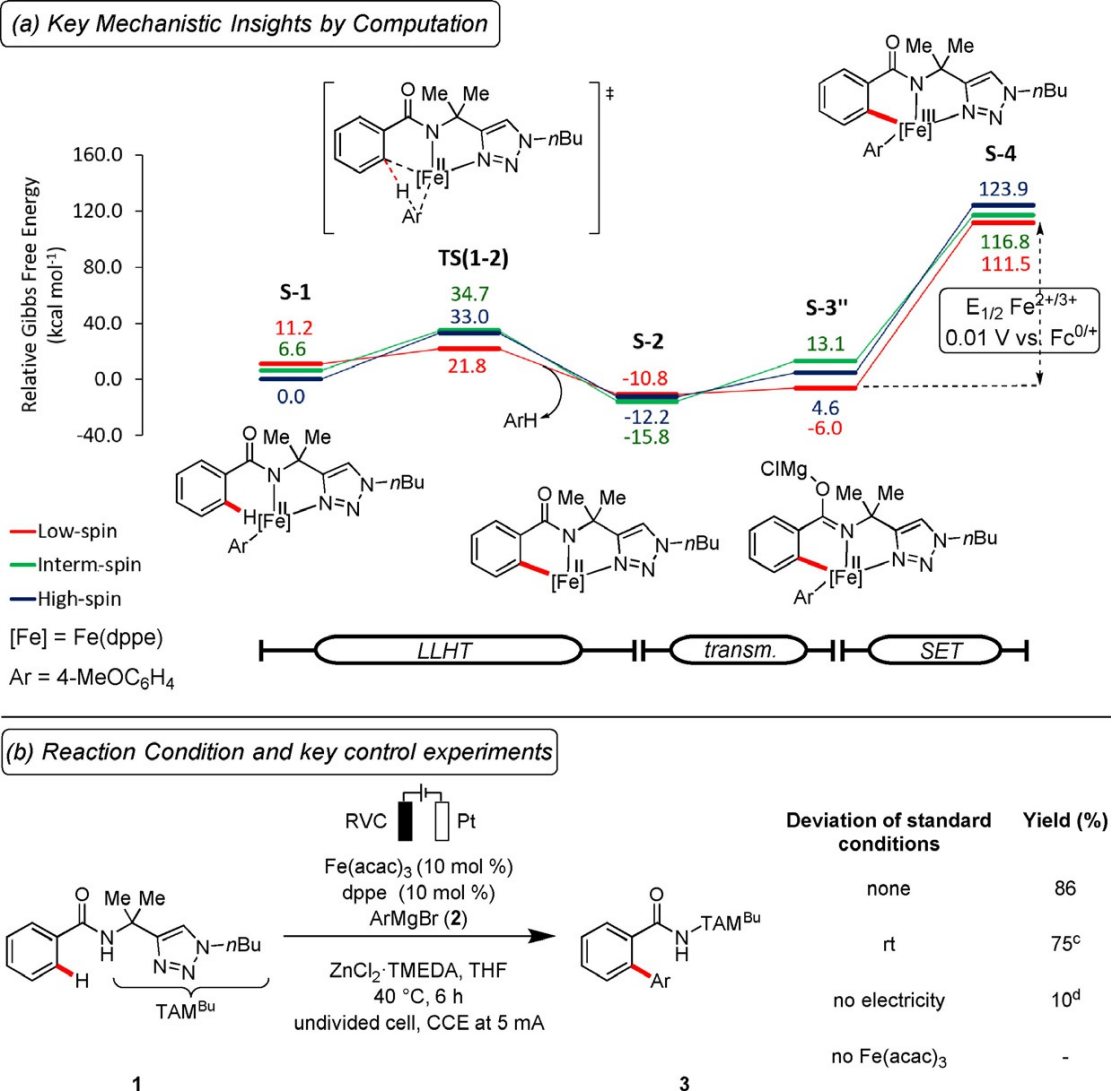
Computation‐guided development of ferraelectrooxidative C−H activation. (a) Computational analysis of the key redox event at the PW6B95‐D3(BJ)/def2‐TZVP+SMD(THF)//TPSS‐D3(BJ)/def2‐SVP level of theory. (b) Reaction conditions: **1** (0.25 mmol), **2** (1.75 mmol), Fe(acac)_3_ (10 mol %), dppe (10 mol %), ZnCl_2_⋅TMEDA (0.75 mmol), THF (5.0 mL), 6 h, constant current electrolysis (CCE) at 5 mA, undivided cell, RVC as anode, Pt‐plate as cathode, Ar=4‐MeOC_6_H_4_, isolated yields. (c) With IKA ElectraSyn at rt. (d) Under N_2_, without electricity.

With the optimized reaction conditions for the electrooxidative iron‐catalyzed C−H arylation being identified, we next probed its robustness with a representative set of benzamides **1** (Scheme 1 a). Differently N‐substituted triazoles **1** were selectively converted into the desired products. Likewise, the robust electrocatalysis enabled the efficient C−H arylation on amides with *para*‐ or *meta*‐substitution patterns. Notably, valuable electrophilic chloro groups as well as oxidation‐sensitive sulfides were fully tolerated (**8**, and **10**), which should prove invaluable for further post‐synthetic late‐stage diversification. Moreover, heteroarene thiophene and ferrocene delivered the desired arylated products **16** and **17** with high catalytic efficacy. The electrochemical C−H activation approach was not limited to TAM‐benzamides. Indeed, the synthetically useful pyridine PIP‐derivative^22^ **18** and **19** proved to be amenable to the C−H activation likewise. In sharp contrast, mono‐dentate pyridine, imine and amide fell thus far short in providing effective electrocatalysis (**3‐I**–**3‐III**). Thereafter, we tested the viable arylation motifs in the iron‐catalyzed electrochemical C−H arylation (Scheme 1 b). Here, a diversity of aromatic scaffolds could be introduced in a programmable fashion, as well as heteroaromatic motifs with excellent levels of chemo‐ and site‐selectivity.

**Scheme 1:**
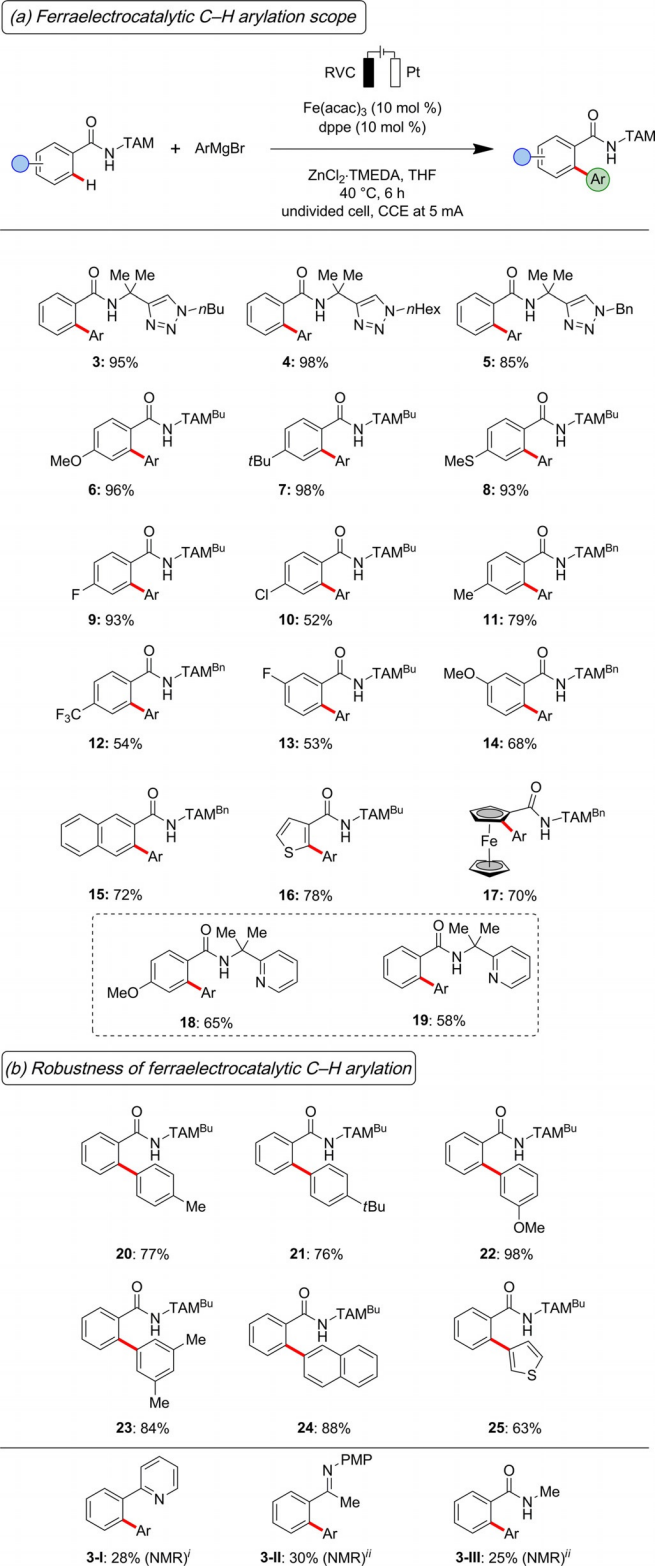
Robustness of the electrochemical C−H arylation of amides **1**. (a) The iron‐electrocatalysis enabled direct arylations of various arenes, ferrocenes and heteroarenes with excellent levels of mono‐, chemo‐, and site‐selectivity, Ar=4‐MeOC_6_H_4_. (b) Various aryl motifs could be introduced by the electrooxidative C−H activation. (*i*) 1,10‐phenanthroline as ligand. (*ii*) 4,4′‐di‐*tert*‐butyl‐2,2′‐bi‐pyridyl (dtbpy) as ligand.

Given the unique features of the DCIB‐free electrochemical C−H activation, we next compared the performance of the heterogeneous ferraelectrocatalysis regime with the optimized homogenous DCIB‐mediated10n transformation. Thus, the performance of the electrocatalysis outperformed the chemical oxidant in terms of the isolated yields (Scheme 2 a) and the kinetic profile (Scheme 2 b), both of which were found to be considerably improved by the iron‐electrocatalysis manifold.

**Scheme 2:**
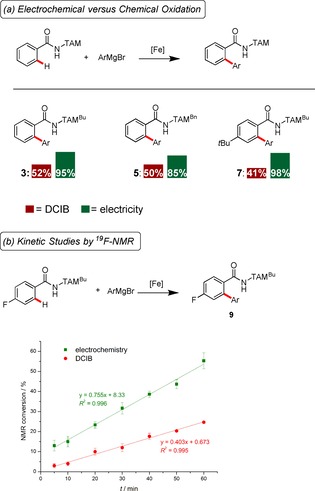
Iron‐electrocatalysis, mechanistic insights. (a) Performance of electrochemical oxidation versus chemical oxidation, isolated yields. (b) Kinetic profiles: Chemical oxidant versus sustainable electricity.

Intrigued by the outstanding efficacy of the electrochemical C−H arylation, we became attracted to unravelling its mode of action. To this end, intermolecular competition experiments revealed electron‐rich substrates to feature an inherent higher reactivity (Scheme 3 a). This finding is not in agreement with an C−H oxidative addition or a concerted‐metalation‐deprotonation (CMD) pathway.^23^ Instead, it can be explained in terms of a ligand‐to‐ligand hydrogen transfer (LLHT)^24^ pathway or base‐assisted internal electrophilic‐type substitution (BIES)^25^ working mode (vide supra).

**Scheme 3:**
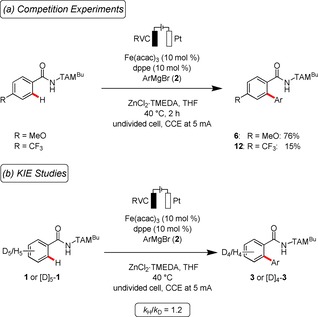
(a) Competition experiments highlight electron**‐**rich arenes to react faster. (b) Kinetic isotope effect studies reveal a fast and facile C−H scission.

Detailed mechanistic studies by cyclic voltammetry have been conducted to delineate the catalyst's mode of action (Scheme 4). First, the electrochemistry of the chemical oxidant DCIB was probed, featuring an irreversible onset potential at *E*_p_=−1.80 V vs. Fc^0/+^ (Scheme 4 a). Second, the addition of the diphosphine ligand dppe shifts the redox‐potential of Fe(acac)_3_ at *E*_1/2_=−1.3 V vs. Fc^0/+^ by 171 mV towards more positive potential (Scheme 4 b). This observation renders a coordination of the phosphine ligand likely to be operative. Third, in the presence of ZnCl_2_⋅TMEDA the reversible redox event becomes quasi‐reversible by shifting the oxidation potential to *E*_p_=−0.46 V vs. Fc^0/+^ (Scheme 4 c, blue). Fourth, it is especially noteworthy that the addition of the Grignard reagent leads to the disappearance of the reversible oxidation of Fe(acac)_3_. In contrast, two new reversible redox events emerge, which can be assigned to the corresponding iron(I)/iron(II) and iron(II)/iron(III) redox events at *E*_1/2_=−0.6 V and at *E*_1/2_=−0.1 V vs. Fc^0/+^, respectively (Scheme 4 c, green). Our observations are in good agreement with cyclic voltammetric studies by Jutand^26^ on iron‐catalyzed Kumada–Corriu‐type cross‐coupling reactions, as well as our previous Mössbauer spectroscopic studies^19^ and current computational findings (vide infra) on iron(II/III/I) catalysis. Our cyclic voltammetry studies on iron‐catalyzed C−H arylations provide experimental insights into oxidation‐induced reductive elimination towards an iron(II/III/I) manifold.

**Scheme 4:**
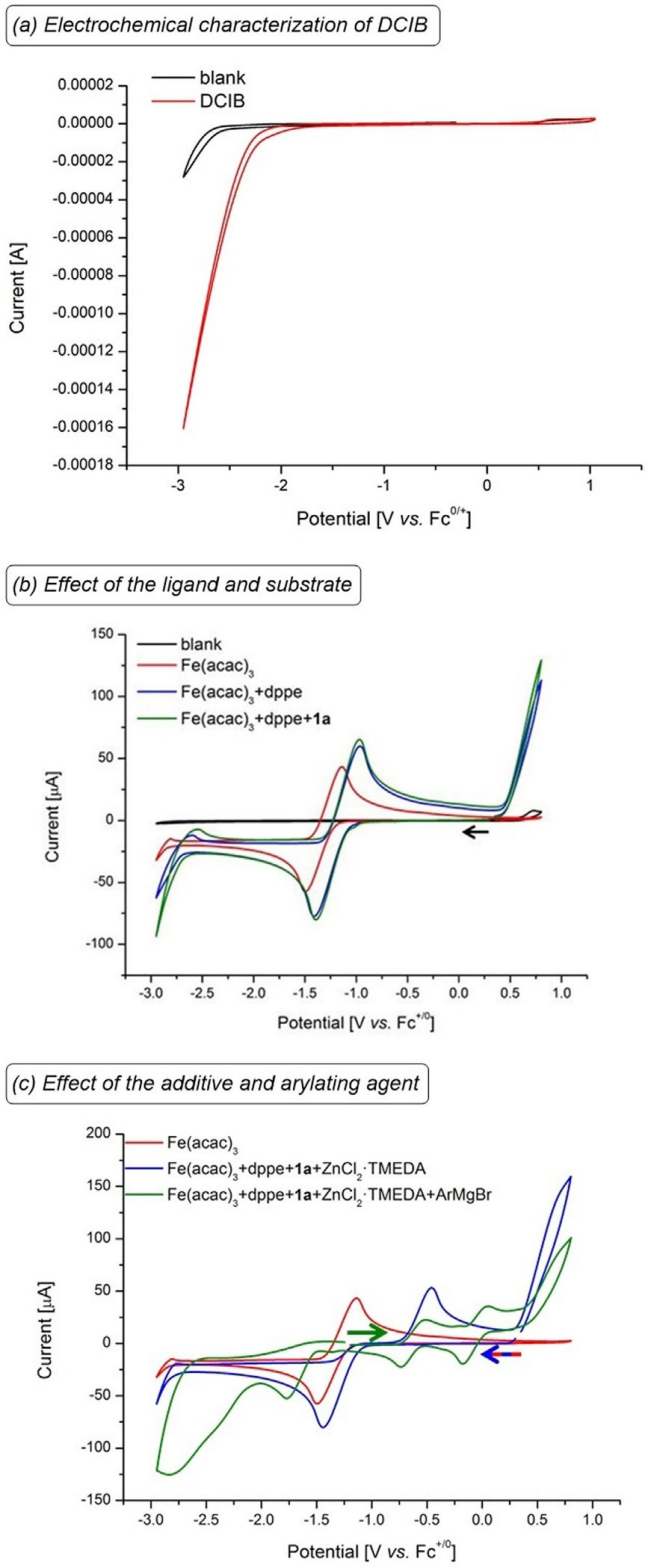
CVs recorded at 100 mV s^−1^ with *n*Bu_4_NPF_6_ (0.1 m in THF), concentrations of substrates 5 mm (ArMgBr 20 mm). (a) DCIB. (b) Ligand effect. (c) Additive and Grignard agent.

While we thus rationalized the anodic oxidation elementary steps, we next interrogated the nature of the cathodic event. Here, detailed analyses of the electrode material by means of scanning electron microscopy energy‐dispersive X‐ray spectroscopy (SEM‐EDS), which clearly highlighted the crucial role of the zinc additive at the surface of the electrode (Scheme 5 a). Thus, the zinc additive serves multiple roles, including the adjustment of the conductivity. Based on these mechanistic studies, a proposed catalytic cycle for the iron‐electrocatalytic C−H functionalization commences by a facile organometallic C−H cleavage (Figure S9 in the Supporting Information).^18^ Thereafter, the key anodic single‐electron‐transfer (SET) oxidation and subsequent transmetalation occur to furnish a five‐membered ferra(III)cycle S‐4, which subsequently undergoes reductive elimination, delivering the desired product **3** and the key iron(I) intermediate S‐6. The catalytically active iron(II) intermediate S‐1 is regenerated by anodic oxidation.

**Scheme 5:**
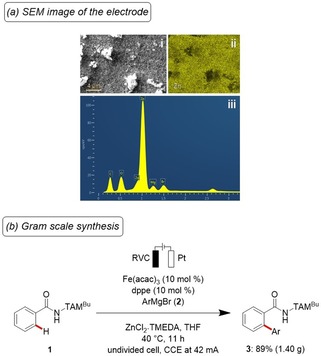
(a) SEM studies on the post‐catalysis cathode material. i) SEM image of deposition. ii) SEM‐EDS mapping with location of zinc. iii) Elemental distribution. (b) Gram‐scale synthesis.

Finally, the robustness of our strategy for electrochemical DCIB‐free C−H arylations was further illustrated by the gram‐scale synthesis of product **3** with comparable levels of efficacy (Scheme 5 b).

In order to shape our understanding of the iron‐electrocatalyzed C−H arylation, we probed its mode of action by DFT calculation at the PW6B95‐D3(BJ)/def2‐TZVP+SMD(THF)//TPSS‐D3(BJ)/def2‐SVP level of theory.^27^ Our findings are hence indicative of a facile C−H activation step, which proceeds through a ligand‐to‐ligand hydrogen transfer transition‐state structure TS(1–2), by means of spin‐crossover with an activation barrier of 21.8 kcal mol^−1^. In order to access the electrooxidative step several pathways were explored. First, we considered an oxidation through SET from intermediate S‐2,^28^ along with transmetalation to give intermediate S‐4 (Figure 3). This route has an endergonic activation barrier with an oxidation potential (Table S4) not comparable to the one that was experimentally observed by CV. The subsequent reductive elimination is very facile. These results indicate the electrooxidation as the rate determining step, which is in good agreement with the reversible C−H activation with an experimentally observed KIE of 1.2.

**Figure 3:**
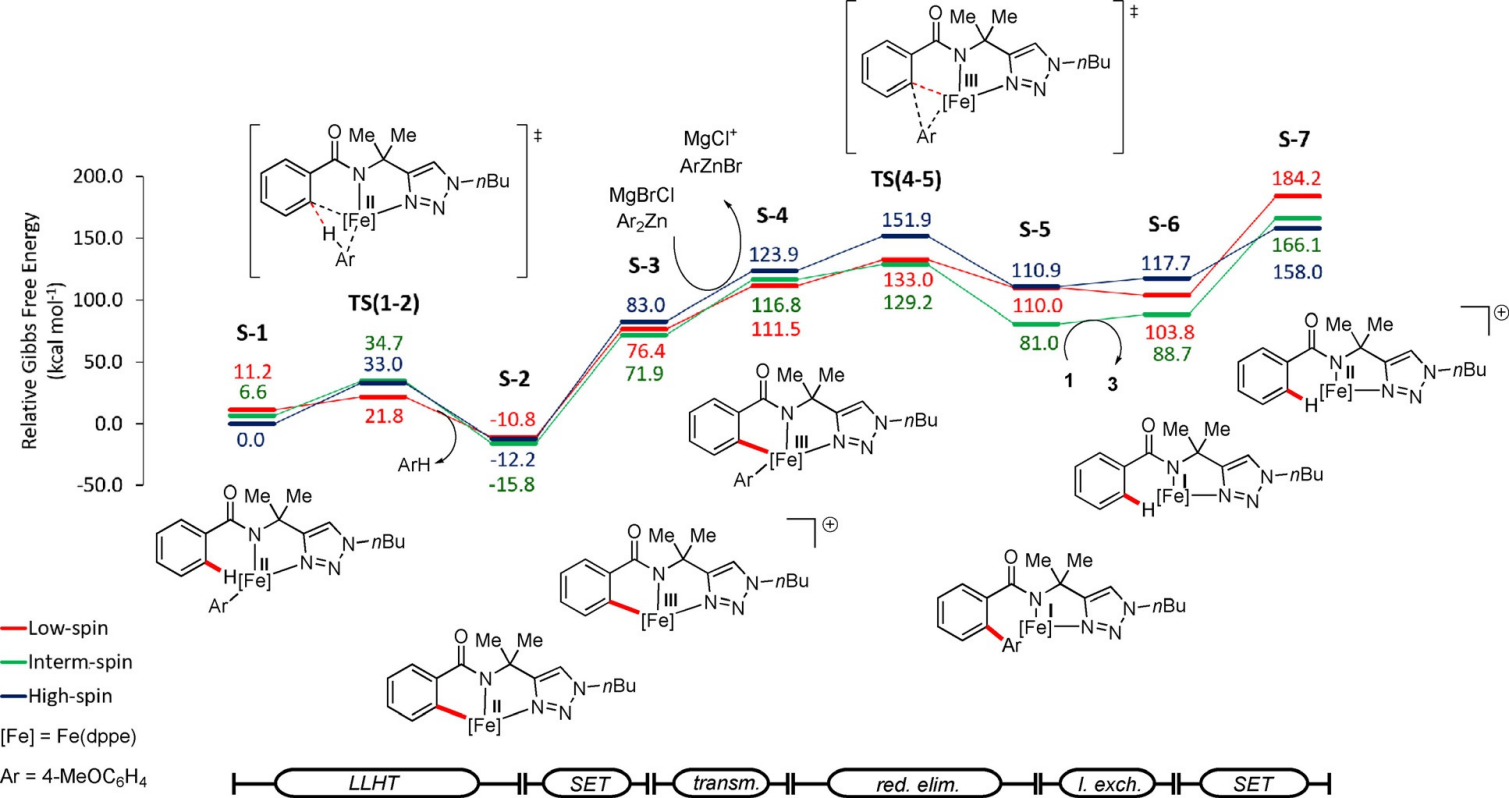
Computed energy profile for the iron‐catalyzed C−H arylation, where oxidation by single‐electron‐transfer occurs after C−H activation followed by transmetalation.

Subsequently, we explored a possible transmetalation prior to the SET (Figure 4), which leads to a considerable decrease of the energy barrier associated with this process. The oxidation potential associated to the iron(II)/iron(III) was found to be comparably low (Table S4). Next, we assessed the influence of the Lewis acidic species in solution on this oxidation‐induced reductive elimination (Figure 5), which significantly lowered the transmetalation activation energy by the formation of a bimetallic iron(II) complex (S‐3′′). This bimetallic intermediate is in good agreement with our recent findings on stoichiometric transformations.19a

**Figure 4:**
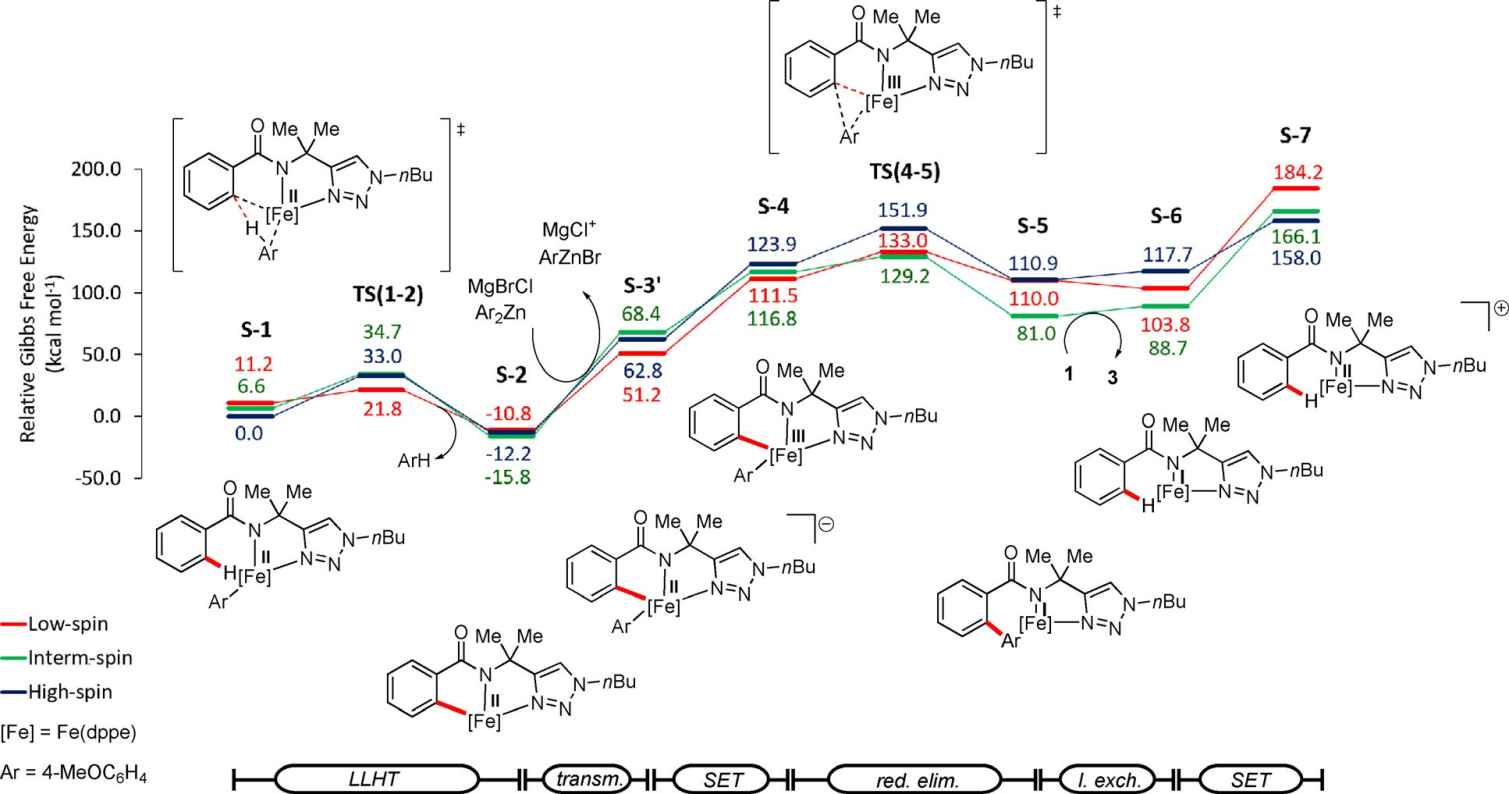
Computed energy profile for the iron‐catalyzed C−H arylation, where oxidation by single‐electron‐transfer occurs after transmetalation.

**Figure 5:**
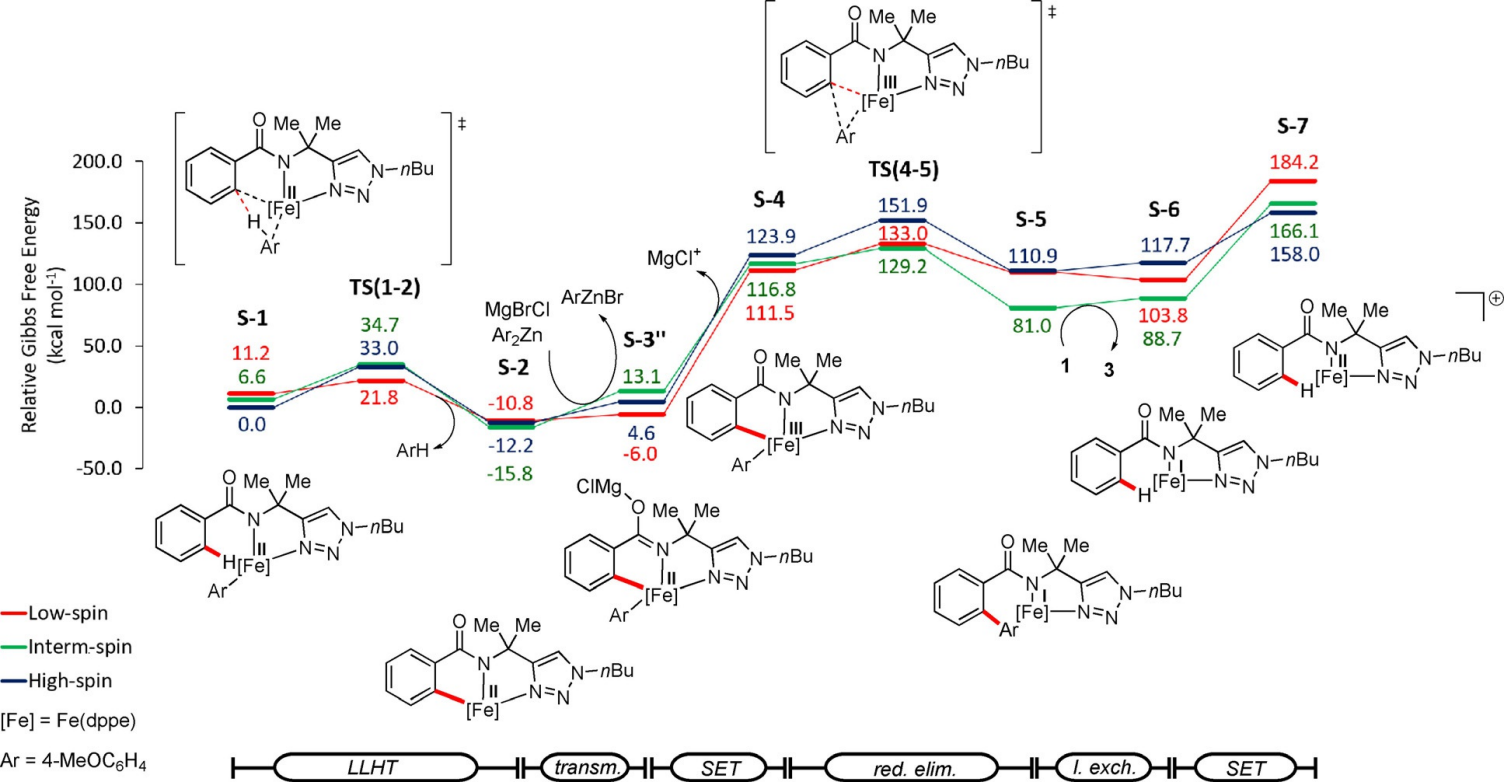
Computed energy profile for the iron‐catalyzed C−H arylation, where oxidation by single‐electron‐transfer occurs after transmetalation with assistance of Lewis acidic magnesium.

This bimetallic intermediate S‐3′′ leads via anodic oxidation to the aryl‐iron(III) complex (S‐4). The calculated half‐wave oxidation potential associated with this process is of 0.01 V vs. ferrocene, which is in excellent agreement with the experimentally observed one (vide supra).

Finally, the generality of the metallaelectrocatalysis strategy was reflected by the merger of electrosynthesis with environmentally benign manganese catalysis. Indeed, unprecedented electrochemical manganese‐catalyzed C−H activation was realized, indicating the broad nature of our strategy beyond iron catalysis, featuring cost‐effective, non‐toxic MnCl_2_ as the catalyst (Scheme 6). It is particularly noteworthy that the manganese‐catalyzed electrochemical C−H arylation did not require any zinc additives. These findings clearly show that this approach is not limited to cathodic zinc reduction manifolds.

**Scheme 6:**
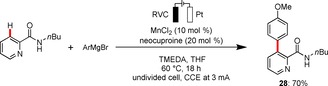
Manganaelectro‐catalyzed C−H activation.

In summary, toxic and cost‐intensive dihalide oxidants were for the first time replaced by electrocatalysis, allowing for versatile iron‐catalyzed C−H activations. The unprecedented ferraelectrocatalytic C−H arylation enabled direct arylations with ample scope, even efficiently occurring at room temperature. Our strategy set the stage for avoiding chemical oxidants in low‐valent metal‐catalyzed C−H activation, featuring non‐toxic, Earth‐abundant iron Fe(acac)_3_ and user‐friendly MnCl_2_ catalysts. Detailed analyses by experiment, spectroscopy and computation unravelled key insights into the role of additives within an iron(II/III/I) manifold, which should prove invaluable for the future design of iron‐ and manganese‐catalyzed electrochemical strong bond activations. Detailed mechanistic studies on 3d metallaelectro‐catalyzed C−H activation are currently ongoing in our laboratories and will be reported in due course.

## Conflict of interest

The authors declare no conflict of interest.

## Supporting information

As a service to our authors and readers, this journal provides supporting information supplied by the authors. Such materials are peer reviewed and may be re‐organized for online delivery, but are not copy‐edited or typeset. Technical support issues arising from supporting information (other than missing files) should be addressed to the authors.

Supplementary
