# Supplementary material for: Iron‐Electrocatalyzed C−H Arylations: Mechanistic Insights into Oxidation‐Induced Reductive Elimination for Ferraelectrocatalysis
Source: Chemistry. 2019 Dec 9;25(71):16382–9. doi: 10.1002/chem.201904018 (PMC6972497; doi:10.1002/chem.201904018)
Supplement: Supplementary file 1 — Supplementary [file CHEM-25-16382-s001.pdf]

CHEMISTRY
A European Journal

Supporting Information

Iron-Electrocatalyzed C–H Arylations: Mechanistic Insights into Oxidation-Induced Reductive Elimination for Ferraelectrocatalysis

Cuiju Zhu,[a] Maximilian Stangier,[a] João C. A. Oliveira,[a] Leonardo Massignan,[a] and Lutz Ackermann*[a, b]

chem_201904018_sm_miscellaneous_information.pdf

Table of content

General Remarks	S-3
Optimization of the Reaction Conditions	S-4
General procedure A: Ferraelectrocatalyzed C–H Arylation	S-6
General procedure B: Manganaelectrocatalyzed C–H Arylation	S-6
Oxidants in Iron-catalyzed C–H Activation	S-7
Characterization Data of Products **3-26**	S-9
Comparision of Electrochemical Oxidation versus Chemical Oxidation	S-27
Kinetic Studies by ^{19}F-NMR	S-28
Intermolecular Competition Experiment	S-31
Kinetic Isotope Effect Studies	S-32
Cyclic Voltammetry	S-34
SEM Analysis	S-37
Plausible Catalytic Cycle	S-38
Gram-Scale Synthesis of **3**	S-39
Computational Studies	S-40
References	S-96
^{1}H-, ^{13}C- and ^{19}F-NMR spectra	S-98

General Remarks

Catalytic reactions were carried out in undivided electrochemical cells (10 mL) using pre-dried glassware. THF was dried using a solvent purification system (SPS) from MBraun. All starting materials **1**,[1] [D_5]-**1a**[2] were prepared according to previously described methods. The Grignard reagents were prepared from the corresponding bromides and magnesium in anhydrous THF and titrated prior to their use according to reported procedures.[3] Other chemicals were obtained from commercial sources and were used without further purification. Platinum electrodes (10 mm × 15 mm × 0.25 mm, 99.9%, obtained from ChemPur) and RVC electrodes (10 mm × 15 mm × 6 mm, SIGRACELL®GFA 6 EA, obtained from SGL Carbon) were connected using stainless steel adapters. Electrocatalysis was conducted using an AXIOMET AX-3003P potentiostat in constant current mode (CCE). Cyclic Voltammetry studies were performed using a Metrohm Autolab PGSTAT204 workstation and Nova 2.0 software. Yields refer to isolated compounds, estimated to be >95% pure as determined by ^{1}H-NMR and GC analysis. Chromatography: Merck silica gel 60 (40–63 µm). NMR: Spectra were recorded on a Varian Mercury 300, Varian Inova 500 or Bruker Avance III 300, Bruker Avance III HD 400 and Bruker Avance III HD 500 in the solvent indicated; chemical shifts (δ) are given in ppm relative to the residual solvent peak. All IR spectra were recorded on a Bruker FT-IR Alpha device. MS: EI-MS-was recorded on Jeol AccuTOF at 70 eV and ESI-MS was recorded on Bruker micrOTOF and maXis. M. p.: Stuart melting point apparatus SMP3, Barloworld Scientific, values are uncorrected. Scanning Electron Microscopy (SEM) images were obtained on an Carl Zeiss EVO LS15 high resolution field emission scanning electron microscope. A layer of carbon was coated on the surface of samples before SEM measurements. The SEM images were captured with an electron beam acceleration voltage of 10 kV. Energy-dispersive X-ray spectroscopy (EDS) spectra were acquired on Oxford instruments X-Max 51-XMX1004.

Optimization of the Reaction Conditions

Table S1: Optimization of the Ferraelectrocatalyzed C–H arylation.[a]

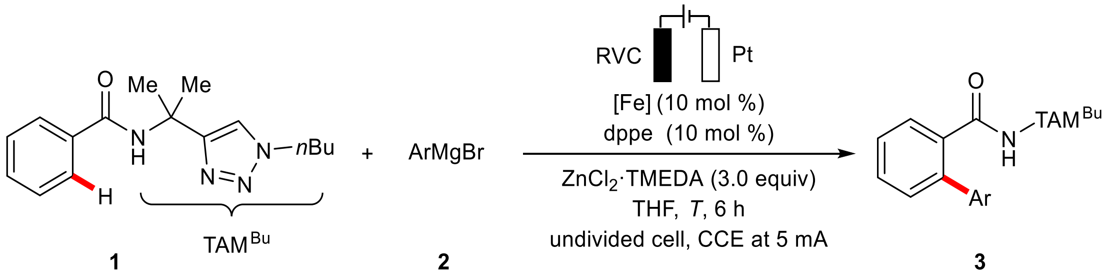

Entry	[Fe]	*T* [°C]	FE [%]	Yield [%]
1	$Fe(acac)_3$	60	34	75[b]
2	$Fe(acac)_3$	60	41	90
3	$Fe(acac)_3$	40	43	95
4	$Fe(acac)_3$	23	33	74
5	$FeCl_3$	40	36	80
6	$FeCl_2$	40	32	72
7	$Fe(acac)_3$	40	5	10[c]
8	$Fe(acac)_3$	40	38	86[d]
9	$Fe(acac)_3$	40	13	28[e]
10	---	40	---	---
11	$Fe(acac)_3$	40	---	---[f]
12	$Fe(acac)_3$	40	34	76[g]
13	$Fe(acac)_3$	40	---	---[h]
14	$Fe(acac)_3$	40	---	---[i]
15	$Fe(acac)_3$	40	33	73[j]
16	$Fe(acac)_3$	40	---	---[k]
17	$Fe(acac)_3$	40	25	56[l]
18	$Fe(acac)_3$	40	40	87[m]
19	$Fe(acac)_3$	40	---	---[n]
20	$Fe(acac)_3$	40	33	73%[o]
21	$Fe(acac)_3$	40	---	---[p]
22	$Fe(acac)_3$	40	30	66%[q]
23	$Fe(acac)_3$	40	27	60%[r]
24	$Fe(acac)_3$	23	34	75[s]
25	$Fe(acac)_3$	40	41	90%[t]

[a] Reaction conditions: **1a** (0.25 mmol), **2a** (1.75 mmol), [Fe] (10 mol %), dppe (10 mol %), $ZnCl_2$·TMEDA (0.75 mmol), THF (5.0 mL), 6 h, constant current

electrolysis (CCE) at 5 mA, undivided cell, RVC as anode (10 mm × 15 mm × 6 mm), Pt-plate as cathode (10 mm × 15 mm × 0.25 mm), isolated yield. [b] RVC as cathode. [c] Without electricity, under N_2. [d] under N_2. [e] Without electricity, under air. [f] TMEDA instead of $ZnCl_2$·TMEDA. [g] $ZnBr_2$·TMEDA instead of $ZnCl_2$·TMEDA. [h] $MnCl_2$·TMEDA instead of $ZnCl_2$·TMEDA. [i] Cp_2Fe instead of $ZnCl_2$·TMEDA. [j] 2-MeTHF instead of THF. [k] **2** (0.50 mmol). [l] **2** (1.0 mmol). [m] 5 h. [n] divided cell. [o] Ni foam as cathode. [p] Mg-plate as anode and cathode. [q] $Fe(acac)_3$ (5 mol %). [r] constant potential electrolysis at 2 V. [s] With IKA® ElectraSyn. Ar = 4-$MeOC_6H_4$, TMEDA = N^1,N^1,N^2,N^2-*tetra*-methylethane-1,2-diamine. [t] $Fe(acac)_3$ (0.25 mmol), 10 min, without electricity.

Table S2: Optimization of the Manganaelectrocatalyzed C–H arylation.[a]

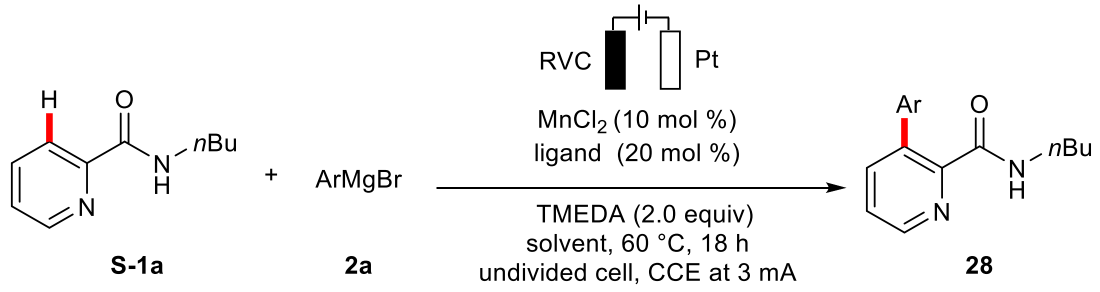

Entry	[TM]	ligand	solvent	Yield [%]
1	$MnCl_2$	2,2'-dipyridine	THF	40
2	$MnCl_2$	1,10-phenanthroline	THF	61
3	$MnCl_2$	neocuproine	THF	70
4	$MnCl_2$	---	THF	34
5	---	neocuproine	THF	---
6	$MnCl_2$	neocuproine	THF	--- [b]
7	$MnCl_2$	neocuproine	THF	67[c]
8	$MnCl_2$	neocuproine	THF	10[d]
9	$MnCl_2$	neocuproine	THF/NMP (4:1)	---
10	$MnCl_2$	neocuproine	GVL	---
11	$Fe(acac)_3$	neocuproine	THF	---
12	(DME)$NiCl_2$	neocuproine	THF	---
13	$RuCl_3$·$(H_2O)_n$	neocuproine	THF	---
14	$PdCl_2$	neocuproine	THF	---

[a] Reaction conditions: Undivided cell, **S-1a** (0.25 mmol), **2a** (1.00 mmol), [TM] (10 mol %), ligand (20 mol %), TMEDA (0.50 mmol), solvent (5.0 mL), 60 °C, 3 mA, 18 h, RVC electrode (1.0×1.5 cm), Pt-plate electrode (1.0×1.5 cm), yield of isolated products. [b] Without TMEDA. [c] 2.5 mA. [d] Without electricity. Ar = 4-$MeOC_6H_4$, TMEDA = N^1,N^1,N^2,N^2-*tetra*-methylethane-1,2-diamine.

General procedure A: Ferraelectrocatalyzed C–H Arylation

The electrocatalysis was carried out in an undivided cell with a RVC anode (10 mm × 15 mm × 6 mm) and a platinum cathode (10 mm × 15 mm × 0.25 mm). A solution of ArMgBr (0.88 mL, 7.0 equiv, 2.0 M in THF) was slowly added to a mixture of amide **1** (0.25 mmol, 1.00 equiv), $Fe(acac)_3$ (8.8 mg, 10 mol %), dppe (10.0 mg, 10 mol %) and $ZnCl_2$·TMEDA (189 mg, 3.00 equiv) were placed in a 10 mL cell and dissolved in THF (5 mL). Electrolysis was performed at 40 °C with a constant current of 5 mA maintained for 6 h (4.50 F/mol). At ambient temperature, a saturated aqueous NH_4Cl solution (10 mL) was added and the RVC anode was washed with EtOAc (3 × 2 mL) in an ultrasonic bath. The combined phases were extracted with EtOAc (3 × 10 mL) and then dried over Na_2SO_4. Evaporation of the solvents and purification by column chromatography on silica gel (*n*-hexane/EtOAc) yielded the desired products **3**-**25**.

General procedure B: Manganaelectrocatalyzed C–H Arylation

The electrocatalysis was carried out in an undivided cell with a RVC anode (10 mm × 15 mm × 6 mm) and a platinum cathode (10 mm × 15 mm × 0.25 mm). A solution of ArMgBr (0.5 mL, 4.0 equiv, 2.0 M in THF) was slowly added to a mixture of amide **S-1a** (0.25 mmol, 1.00 equiv), $MnCl_2$ (3.1 mg, 10 mol %), neocuproine (10.4 mg, 20 mol %) and TMEDA (74 µL, 2.0 equiv) were placed in a 10 mL cell and dissolved in THF (5 mL). Electrolysis was performed at 60 °C with a constant current of 3 mA maintained for 18 h. At ambient temperature, a saturated aqueous NH_4Cl solution (10 mL) was added and the RVC anode was washed with EtOAc (3 × 2 mL) in an ultrasonic bath. The combined phases were extracted with EtOAc (3 × 10 mL) and then dried over Na_2SO_4. Evaporation of the solvents and purification by column chromatography on silica gel (*n*-hexane/EtOAc) yielded the desired product **26**.

Oxidants in Iron-catalyzed C–H Activation

Table S3: Literature summary: An analysis of literature reports shows that DCIB is dominant in organometallic iron-catalyzed C–H activation chemistry.

Title	Oxidant	Reference
Iron-Catalyzed Direct Arylation through Directed C−H Bond Activation.	DCIB[a]	*J. Am. Chem. Soc.* **2008,** *130*, 5858-5859
Iron-Catalyzed Chemoselective *ortho* Arylation of Aryl Imines by Directed C−H Bond Activation	DCIB	*Angew. Chem. Int. Ed.* **2009,** *48*, 2925-2928.
Iron-Catalyzed Stereospecific Activation of Olefinic C–H Bonds with Grignard Reagent for Synthesis of Substituted Olefins.	DCIB	*J. Am. Chem. Soc.* **2011,** *133*, 7672-7675.
Iron-Catalyzed C−H Bond Activation for the *ortho*-Arylation of Aryl Pyridines and Imines with Grignard Reagents.	DCIB	*Chem. Asian J.* **2011,** *6*, 3059-3065.
Iron-Catalyzed C(sp^2)–H Bond Functionalization with Organoboron Compounds.	DCIB	*J. Am. Chem. Soc.* **2014,** *136*, 14349-14352.
Iron-Catalyzed *ortho* Monoarylation of Benzamide Derivatives.	DCIB	*Asian J. Org. Chem.* **2012,** *1*, 142-145.
Iron-Catalyzed Nitrogen-Directed Coupling of Arene and Aryl Bromides Mediated by Metallic Magnesium.	DCIB	*Adv. Synth. Catal* **2012,** *354*, 593-596.
Iron-Catalyzed C(sp^2) −H and C(sp^3)−H Arylation by Triazole Assistance.	DCIB	*Angew. Chem. Int. Ed.* **2014,** *53*, 3868-3871.
Iron-Catalyzed Arylation of Heterocycles via Directed C–H Bond Activation.	DCIB	*Org. Lett.* **2014,** *16*, 868-871.
Iron-Catalyzed Directed Alkylation of Alkenes and Arenes with Alkylzinc Halides.	DCIB	*Adv. Synth. Catal* **2015,** *357*, 2175-2179.
Iron-Catalyzed Directed C(sp^2)–H and C(sp^3)–H Functionalization with Trimethylaluminum.	2,3-DCB[b]	*J. Am. Chem. Soc.* **2015,** *137*, 7660-7663.

Iron-Catalyzed *Ortho* C–H Methylation of Aromatics Bearing a Simple Carbonyl Group with Methylaluminum and Tridentate Phosphine Ligand.	2,3-DCB	*J. Am. Chem. Soc.* **2016,** *138,* 10132-10135.
Iron-Catalyzed C(sp^2)–H and C(sp^3)–H Methylations of Amides and Anilides.	DCIB	*Chem. Eur. J.* **2015,** *21,* 8812-8815.
β-Arylation of Carboxamides via Iron-Catalyzed C(sp^3)–H Bond Activation.	DCIB	*J. Am. Chem. Soc.* **2013,** *135,* 6030-6032.
Iron/Zinc-Co-catalyzed Directed Arylation and Alkenylation of C(sp^3)–H Bonds with Organoborates.	DCIB	*ACS Catal.* **2017,** *7,* 89-92.
Phenanthrene Synthesis by Iron-Catalyzed [4 + 2] Benzannulation between Alkyne and Biaryl or 2-Alkenylphenyl Grignard Reagent.	DCIB	*J. Am. Chem. Soc.* **2011,** *133,* 6557-6559.
Synthesis of Polysubstituted Naphthalenes by Iron-Catalyzed [2+2+2] Annulation of Grignard Reagents with Alkynes.	DCIB	*Synlett* **2012,** *23,* 2381-2384.
Iron-catalyzed C-H/N-H activation by triazole guidance: versatile alkyne annulation.	DCIB	*Chem. Commun.* **2017,** *53,* 6460-6463.
Tri-Substituted Triazole-Enabled C–H Activation of Benzyl and Aryl Amines by Iron Catalysis.	2,3-DCB	*Org. Lett.* **2017,** *19,* 3795-3798.

[a] The price for DCIB is 4086 USD/mol based on 5g Package (List Price Ambeed, Inc. USA). [b] The price for 2,3-DCB is 1258 USD/mol based on 10g Package (List Price Ambeed, Inc. USA).

Characterization Data of Products 3-26

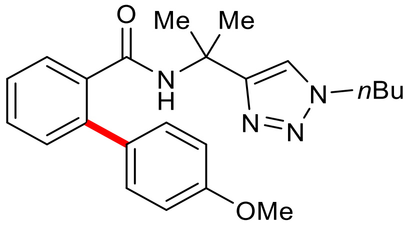

3

***N*-[2-(1-*n*-Butyl-1*H*-1,2,3-triazol-4-yl)propan-2-yl]-4'-methoxy-[1,1'-biphenyl]-2--carboxamide (3)**

The general procedure was followed using **1a** (71.6 mg, 0.25 mmol) and **2a** (0.88 mL, 1.75 mmol, 2.0 M in THF). Purification by column chromatography on silica gel (*n*-hexane/EtOAc: 3:1→1:1) yielded **3** (93.2 mg, 95%) as a white solid. **M. p.** = 112–113 °C. **^{1}H-NMR** (400 MHz, $CDCl_3$) δ = 7.63 (dd, J = 7.5, 1.5 Hz, 1H), 7.42 (ddd, J = 7.5, 7.5, 1.5 Hz, 1H), 7.38–7.28 (m, 5H), 6.91 (d, J = 8.8 Hz, 2H), 5.89 (s_{br}, 1H), 4.27 (t, J = 7.3 Hz, 2H), 3.84 (s, 3H), 1.93–1.79 (m, 2H), 1.60 (s, 6H), 1.41–1.28 (m, 2H), 0.95 (t, J = 7.4 Hz, 3H). **^{13}C-NMR** (100 MHz, $CDCl_3$) δ = 168.5 (C_q), 159.3 (C_q), 152.7 (C_q), 139.3 (C_q), 136.2 (C_q), 132.7 (C_q), 130.2 (CH), 130.1 (CH), 129.9 (CH), 128.6 (CH), 127.1 (CH), 120.4 (CH), 113.9 (CH), 55.4 (CH_3), 51.6 (C_q), 50.0 (CH_2), 32.2 (CH_2), 27.5 (CH_3), 19.8 (CH_2), 13.5 (CH_3). **IR** (ATR): 3243, 3142, 2954, 1664, 1515, 1300, 1242, 831 cm^{-1}. **MS** (ESI) *m/z* (relative intensity): 807 (12) $[2M+Na]^+$, 415 (52) $[M+Na]^+$, 393 (34) $[M+H]^+$. **HR-MS** (ESI) *m/z* calcd for $C_{23}H_{29}N_4O_2$ $[M+H]^+$ 393.2285, found 393.2284. The analytical data are in accordance to those reported in the literature.[4]

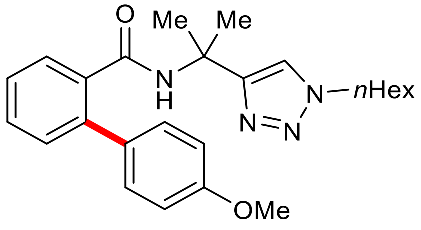

4

***N*-[2-(1-*n*-Hexyl-1*H*-1,2,3-triazol-4-yl)propan-2-yl]-4'-methoxy-[1,1'-biphenyl]-2--carboxamide (4)**

The general procedure A was followed using **1b** (78.6 mg, 0.25 mmol) and **2a** (0.88 mL, 1.75 mmol, 2.0 M in THF). Purification by column chromatography on silica gel (*n*-hexane/EtOAc: 3:1→1:1) yielded **4** (103.0 mg, 98%) as a white solid. **M. p.** = 86–87 °C. **^{1}H-NMR** (400 MHz, $CDCl_3$) δ = 7.61 (dd, *J* = 7.5, 1.5 Hz, 1H), 7.41 (ddd, *J* = 7.5, 7.5, 1.5 Hz, 1H), 7.36–7.28 (m, 5H), 6.90 (d, *J* = 8.8 Hz, 2H), 5.91 (s_{br}, 1H), 4.28 (t, *J* = 7.3 Hz, 2H), 3.83 (s, 3H), 1.95–1.80 (m, 2H), 1.59 (s, 6H), 1.37–1.27 (m, 6H), 0.87 (t, *J* = 7.0 Hz, 3H). **^{13}C-NMR** (100 MHz, $CDCl_3$) δ = 168.5 (C_q), 159.3 (C_q), 152.7 (C_q), 139.2 (C_q), 136.2 (C_q), 132.6 (C_q), 130.1 (CH), 130.0 (CH), 129.8 (CH), 128.5 (CH), 127.1 (CH), 120.4 (CH), 113.9 (CH), 55.3 (CH_3), 51.6 (C_q), 50.2 (CH_2), 31.1 (CH_2), 30.2 (CH_2), 27.4 (CH_3), 26.1 (CH_2), 22.4 (CH_2), 13.9 (CH_3). **IR** (ATR): 3293, 2927, 2858, 1637, 1544, 1514, 1240, 831 cm^{-1}. **MS** (ESI) *m/z* (relative intensity): 863 (18) $[2M+Na]^+$, 443 (60) $[M+Na]^+$, 421 (100) $[M+H]^+$. **HR-MS** (ESI) *m/z* calcd for $C_{25}H_{33}N_4O_2$ $[M+H]^+$ 421.2598, found 421.2599.

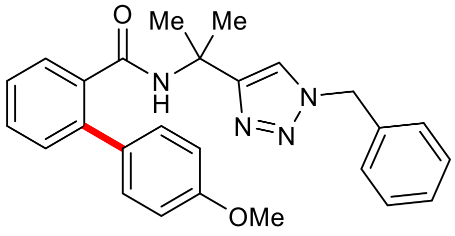

5

N-[2-(1-Benzyl-1*H*-1,2,3-triazol-4-yl)propan-2-yl]-4'-methoxy-[1,1'-biphenyl]-2-carboxamide (5)

The general procedure A was followed using **1c** (80.1 mg, 0.25 mmol) and **2a** (0.88 mL, 1.75 mmol, 2.0 M in THF). Purification by column chromatography on silica gel (*n*-hexane/EtOAc: 3:1→1:1) yielded **5** (90.6 mg, 85%) as a white solid. **M. p.** = 136–137 °C. **^{1}H-NMR** (400 MHz, $CDCl_3$) δ = 7.60 (dd, *J* = 7.5, 1.5 Hz, 1H), 7.40 (ddd, *J* = 7.5, 7.5, 1.5 Hz, 1H), 7.39–7.19 (m, 10H), 6.85 (d, *J* = 8.7 Hz, 2H), 5.87 (s_{br}, 1H), 5.45 (s, 2H), 3.79 (s, 3H), 1.56 (s, 6H). **^{13}C-NMR** (100 MHz, $CDCl_3$) δ = 168.5 (C_q), 159.2 (C_q), 153.2 (C_q), 139.2 (C_q), 136.1 (C_q), 134.8 (C_q), 132.6 (C_q), 130.1 (CH), 130.0 (CH), 129.8 (CH), 129.0 (CH), 128.5 (CH), 127.9 (CH), 127.1 (CH), 120.5 (CH), 113.9 (CH), 55.3 (CH_3), 53.9 (C_q), 51.6 (CH_2), 27.4 (CH_3). **IR** (ATR): 3300, 1639, 1516, 1251,

1180, 836, 732, 578 cm^{-1}. **MS** (ESI) *m/z* (relative intensity): 875 (26) [2M+Na]$^+$, 449 (65) [M+Na]$^+$, 427 (100) [M+H]$^+$. **HR-MS** (ESI) *m/z* calcd for $C_{26}H_{27}N_4O_2$ [M+H]$^+$ 427.2129, found 427.2127.

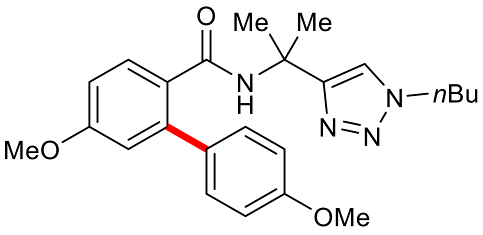

6

_N_-[2-(1-_n_-Butyl-1_H_-1,2,3-triazol-4-yl)propan-2-yl]-4',5-dimethoxy-[1,1'-biphenyl]-2-carboxamide (6)

The general procedure A was followed using **1d** (79.1 mg, 0.25 mmol) and **2a** (0.88 mL, 1.75 mmol, 2.0 M in THF). Purification by column chromatography on silica gel (*n*-hexane/EtOAc: 3:1→1:1) yielded **6** (101.4 mg, 96%) as a white solid. **M. p.** = 91–92 °C. **^{1}H-NMR** (400 MHz, $CDCl_3$) δ = 7.59 (d, J = 8.6 Hz, 1H), 7.32 (s, 1H), 7.25 (d, J = 8.7 Hz, 2H), 6.87 (d, J = 8.7 Hz, 2H), 6.82 (dd, J = 8.6, 2.6 Hz, 1H), 6.74 (d, J = 2.6 Hz, 1H), 5.79 (s_{br}, 1H), 4.22 (t, J = 7.3 Hz, 2H), 3.79 (s, 3H), 3.77 (s, 3H) 1.89–1.74 (m, 2H), 1.52 (s, 6H), 1.34–1.23 (m, 2H), 0.90 (t, J = 7.4 Hz, 3H). **^{13}C-NMR** (100 MHz, $CDCl_3$) δ = 167.8 (C_q), 160.4 (C_q), 159.3 (C_q), 152.7 (C_q), 141.1 (C_q), 132.6 (C_q), 130.6 (CH), 129.9 (CH), 128.5 (C_q), 120.3 (CH), 115.4 (CH), 113.8 (CH), 112.4 (CH), 55.3 (CH_3), 55.2 (CH_3), 51.3 (C_q), 49.8 (CH_2), 32.1 (CH_2), 27.4 (CH_3), 19.6 (CH_2), 13.3 (CH_3). **IR** (ATR): 3301, 2961, 1627, 1600, 1317, 1031, 838 cm^{-1}. **MS** (ESI) *m/z* (relative intensity): 867 (13) [2M+Na]$^+$, 445 (52) [M+Na]$^+$, 423 (100) [M+H]$^+$. **HR-MS** (ESI) *m/z* calcd for $C_{24}H_{31}N_4O_3$ [M+H]$^+$ 423.2391, found 423.2389.

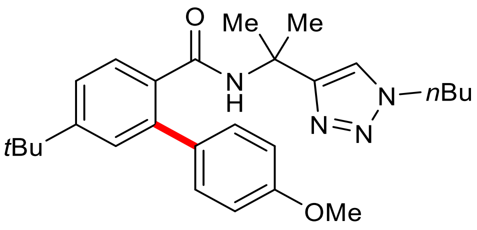

7

5-(*tert*-Butyl)-*N*-[2-(1-*n*-butyl-1*H*-1,2,3-triazol-4-yl)propan-2-yl]-4'-methoxy-[1,1'-biphenyl]-2-carboxamide (7)

The general procedure A was followed using **1e** (85.6 mg, 0.25 mmol) and **2a** (0.88 mL, 1.75 mmol, 2.0 M in THF). Purification by column chromatography on silica gel (*n*-hexane/EtOAc: 3:1→1:1) yielded **7** (109.9 mg, 98%) as a white solid. **M. p.** = 100–101 °C. **^{1}H-NMR** (400 MHz, $CDCl_3$) δ = 7.58 (d, J = 8.2 Hz, 1H), 7.37 (dd, J = 8.2, 2.0 Hz, 1H), 7.34–7.27 (m, 4H), 6.92 (d, J = 8.8 Hz, 2H), 5.86 (s_{br}, 1H), 4.26 (t, J = 7.3 Hz, 2H), 3.83 (s, 3H), 1.89–1.77 (m, 2H), 1.58 (s, 6H), 1.32 (s, 11H), 0.94 (t, J = 7.4 Hz, 3H). **^{13}C-NMR** (100 MHz, $CDCl_3$) δ = 168.4 (C_q), 159.2 (C_q), 153.1 (C_q), 152.7 (C_q), 138.9 (C_q), 133.3 (C_q), 133.2 (C_q), 130.1 (CH), 128.4 (CH), 127.2 (CH), 124.2 (CH), 120.4 (CH), 113.8 (CH), 55.3 (CH_3), 51.5 (C_q), 49.8 (CH_2), 34.7 (C_q), 32.1 (CH_2), 31.1 (CH_3), 27.4 (CH_3), 19.7 (CH_2), 13.4 (CH_3). **IR** (ATR): 3281, 2961, 1634, 1514, 1240, 1044, 834 cm^{-1}. **MS** (ESI) *m/z* (relative intensity): 919 (10) $[2M+Na]^+$, 471 (30) $[M+Na]^+$, 449 (100) $[M+H]^+$. **HR-MS** (ESI) *m/z* calcd for $C_{27}H_{37}N_4O_2$ $[M+H]^+$ 449.2911, found 449.2907.

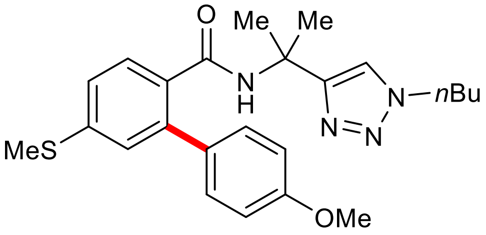

8

***N*-[2-(1-*n*-Butyl-1*H*-1,2,3-triazol-4-yl)propan-2-yl]-4'-methoxy-5-(methylthio)-[1,1'-biphenyl]-2-carboxamide (8)**

The general procedure A was followed using **1f** (83.1 mg, 0.25 mmol) and **2a** (0.88 mL, 1.75 mmol, 2.0 M in THF). Purification by column chromatography on silica gel (*n*-hexane/EtOAc: 3:1→1:1) yielded **8** (102.0 mg, 93%) as a white solid. **M. p.** = 94–95 °C. **^{1}H-NMR** (400 MHz, $CDCl_3$) δ = 7.55 (d, J = 8.1 Hz, 1H), 7.31 (s, 1H), 7.25 (d, J = 8.7 Hz, 2H), 7.16 (dd, J = 8.2, 2.0 Hz, 1H), 7.08 (d, J = 1.9 Hz, 1H), 6.88 (d, J = 8.7 Hz, 2H), 5.85 (s_{br}, 1H), 4.23 (t, J = 7.3 Hz, 2H), 3.80 (s, 3H), 2.45 (s, 3H), 1.90–

1.75 (m, 2H), 1.54 (s, 6H), 1.35–1.26 (m, 2H), 0.91 (t, J = 7.4 Hz, 3H). **^{13}C-NMR** (100 MHz, $CDCl_3$) δ = 167.8 (C_q), 159.4 (C_q), 152.6 (C_q), 141.2 (C_q), 139.8 (C_q), 132.5 (C_q), 132.2 (C_q), 130.0 (CH), 129.2 (CH), 127.2 (CH), 124.3 (CH), 120.4 (CH), 113.9 (CH), 55.3 (CH_3), 51.5 (C_q), 49.9 (CH_2), 32.1 (CH_2), 27.4 (CH_3), 19.7 (CH_2), 15.2 (CH_3), 13.4 (CH_3). **IR** (ATR): 3274, 2869, 1633, 1513, 1239, 1025, 831 cm^{-1}. **MS** (ESI) *m/z* (relative intensity): 899 (10) [2M+Na]$^+$, 461 (35) [M+Na]$^+$, 439 (100) [M+H]$^+$. **HR-MS** (ESI) *m/z* calcd for $C_{24}H_{31}N_4O_2S$ [M+H]$^+$ 439.2162, found 439.2159.

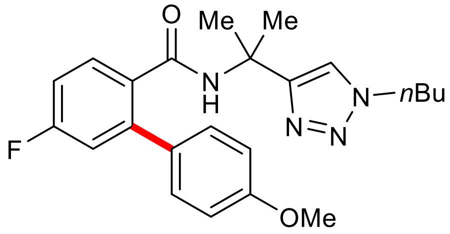

9

_N_-[2-(1-_n_-Butyl-1_H_-1,2,3-triazol-4-yl)propan-2-yl]-5-fluoro-4'-methoxy-[1,1'-biphenyl]-2-carboxamide (9)

The general procedure A was followed using **1g** (76.1 mg, 0.25 mmol) and **2a** (0.88 mL, 1.75 mmol, 2.0 M in THF). Purification by column chromatography on silica gel (*n*-hexane/EtOAc: 3:1→1:1) yielded **9** (95.4 mg, 93%) as a white solid. **M. p.** = 109–110 °C. **^{1}H-NMR** (400 MHz, $CDCl_3$) δ = 7.58 (dd, J = 8.5, 6.1 Hz, 1H), 7.32 (s, 1H), 7.25 (d, J = 8.8 Hz, 2H), 7.04–6.92 (m, 2H), 6.87 (d, J = 8.8 Hz, 2H), 5.92 (s_{br}, 1H), 4.24 (t, J = 7.4 Hz, 2H), 3.79 (s, 3H), 1.91–1.73 (m, 2H), 1.55 (s, 6H), 1.41–1.21 (m, 2H), 0.91 (t, J = 7.4 Hz, 3H). **^{13}C-NMR** (100 MHz, $CDCl_3$) δ = 167.5 (C_q), 163.0 (d, $^1J_{C-F}$ = 250.3 Hz, C_q), 159. 6 (C_q), 152.6 (C_q), 141.8 (d, $^3J_{C-F}$ = 8.3 Hz, C_q), 132.3 (d, $^4J_{C-F}$ = 3.4 Hz, C_q), 131.4 (d, $^4J_{C-F}$ = 1.8 Hz, C_q), 130.8 (d, $^3J_{C-F}$ = 9.0 Hz, CH), 129.9 (CH), 120.3 (CH), 116.9 (d, $^2J_{C-F}$ = 21.9 Hz, CH), 114.0 (CH), 113.9 (d, $^2J_{C-F}$ = 21.3 Hz, CH), 55.3 (CH_3), 51.6 (C_q), 49.9 (CH_2), 32.1 (CH_2), 27.3 (CH_3), 19.7 (CH_2), 13.4 (CH_3). **^{19}F-NMR** (376 MHz, $CDCl_3$) δ = –110.69 (ddd, J = 8.7, 8.7, 5.8 Hz). **IR** (ATR): 3274, 2963, 2873, 1630, 1608, 1517, 1026, 835 cm^{-1}. **MS** (ESI) *m/z* (relative intensity):

843 (40) [2M+Na]$^+$, 433 (75) [M+Na]$^+$, 411 (100) [M+H]$^+$. **HR-MS** (ESI) *m/z* calcd for $C_{23}H_{28}FN_4O_2$ [M+H]$^+$ 411.2191, found 411.2188.

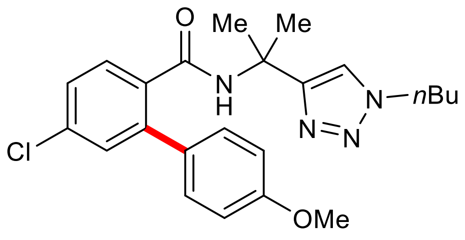

10

***N*-[2-(1-*n*-Butyl-1*H*-1,2,3-triazol-4-yl)propan-2-yl]-5-chloro-4'-methoxy-[1,1'-biphenyl]-2-carboxamide (10)**

The general procedure A was followed using **1h** (80.2 mg, 0.25 mmol) and **2a** (0.88 mL, 1.75 mmol, 2.0 M in THF). Purification by column chromatography on silica gel (*n*-hexane/EtOAc: 3:1→1:1) yielded **10** (55.5 mg, 52%) as a white solid. **M. p.** = 106–107 °C. **^{1}H-NMR** (400 MHz, $CDCl_3$) δ = 7.53 (d, J = 8.8 Hz, 1H), 7.32 (s, 1H), 7.29–7.23 (m, 4H), 6.88 (d, J = 8.8 Hz, 2H), 5.94 (s_{br}, 1H), 4.24 (t, J = 7.3 Hz, 2H), 3.80 (s, 3H), 1.87–1.78 (m, 2H), 1.55 (s, 6H), 1.37–1.22 (m, 2H), 0.92 (t, J = 7.4 Hz, 3H). **^{13}C-NMR** (100 MHz, $CDCl_3$) δ = 167.4 (C_q), 159.6 (C_q), 152.5 (C_q), 141.0 (C_q), 135.6 (C_q), 134.5 (C_q), 131.2 (C_q), 130.1 (CH), 130.0 (CH), 129.9 (CH), 127.1 (CH), 120.3 (CH), 114.0 (CH), 55.3 (CH_3), 51.6 (C_q), 49.9 (CH_2), 32.1 (CH_2), 27.4 (CH_3), 19.7 (CH_2), 13.4 (CH_3). **IR** (ATR): 3272, 2958, 2089, 1631, 1549, 1257, 1023, 837 cm^{-1}. **MS** (ESI) *m/z* (relative intensity): 875 (10) [2M+Na]$^+$ (^{35}Cl), 449 (90) [M+Na]$^+$ (^{35}Cl), 427 (100) [M+H]$^+$ (^{35}Cl). **HR-MS** (ESI) *m/z* calcd for $C_{27}H_{28}{}^{35}ClN_4O_2$ [M+H]$^+$ 427.1895, found 427.1890.

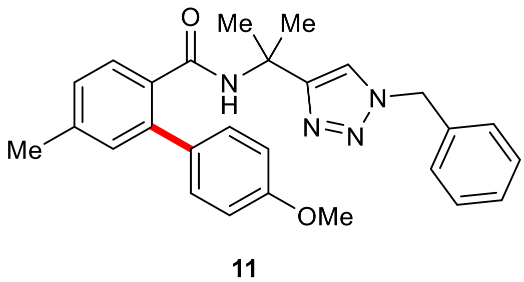

11

***N*-[2-(1-Benzyl-1*H*-1,2,3-triazol-4-yl)propan-2-yl]-4'-methoxy-5-methyl- [1,1'-**

biphenyl]-2-carboxamide (11)

The general procedure A was followed using **1i** (83.6 mg, 0.25 mmol) and **2a** (0.88 mL, 1.75 mmol, 2.0 M in THF). Purification by column chromatography on silica gel (*n*-hexane/EtOAc: 3:1→1:1) yielded **11** (87.0 mg, 79%) as a white solid. **M. p.** = 89–90 °C. **^{1}H-NMR** (300 MHz, $CDCl_3$) δ = 7.51 (d, J = 7.8 Hz, 1H), 7.39–7.28 (m, 4H), 7.26–7.19 (m, 4H), 7.13 (dd, J = 7.8, 1.8 Hz, 1H), 7.07 (d, J = 1.8 Hz, 1H), 6.83 (d, J = 8.7 Hz, 2H), 5.79 (s_{br}, 1H), 5.43 (s, 2H), 3.78 (s, 3H), 2.35 (s, 3H), 1.53 (s, 6H). **^{13}C-NMR** (125 MHz, $CDCl_3$) δ = 168.2 (C_q), 159.1 (C_q), 153.1 (C_q), 139.9 (C_q), 139.1 (C_q), 134.7 (C_q), 133.2 (C_q), 132.7 (C_q), 130.7 (CH), 129.9 (CH), 128.9 (CH), 128.7 (CH), 128.4 (CH), 127.8 (CH), 127.7 (CH), 120.5 (CH), 113.8 (CH), 55.3 (CH_3), 53.9 (CH_2), 51.5 (C_q), 27.5 (CH_3), 21.3 (CH_3). **IR** (ATR): 3272, 2954, 1628, 1514, 1244, 1030, 582 cm^{-1}. **MS** (ESI) *m/z* (relative intensity): 903 (10) [2M+Na]$^{+}$, 463 (45) [M+Na]$^{+}$, 441 (100) [M+H]$^{+}$. **HR-MS** (ESI) *m/z* calcd for $C_{27}H_{29}N_4O_2$ [M+H]$^{+}$ 441.2285, found 441.2287.

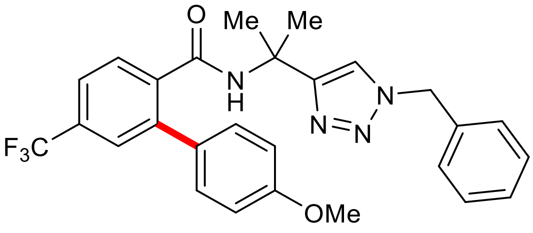

12

***N*-[2-(1-Benzyl-1*H*-1,2,3-triazol-4-yl)propan-2-yl]-4'-methoxy-5-(trifluoromethyl)-[1,1'-biphenyl]-2-carboxamide (12)**

The general procedure A was followed using **1j** (97.1 mg, 0.25 mmol) and **2a** (0.88 mL, 1.75 mmol, 2.0 M in THF). Purification by column chromatography on silica gel (*n*-hexane/EtOAc: 3:1→1:1) yielded **12** (66.8 mg, 54%) as a white solid. **M. p.** = 100–101 °C. **^{1}H-NMR** (400 MHz, $CDCl_3$) δ = 7.68 (dd, J = 7.9, 0.8 Hz, 1H), 7.59–7.52 (m, 2H), 7.39–7.30 (m, 3H), 7.31–7.26 (m, 3H), 7.24–7.19 (m, 2H), 6.86 (d, J = 8.7 Hz, 2H), 6.00 (s_{br}, 1H), 5.44 (s, 2H), 3.79 (s, 3H), 1.56 (s, 6H). **^{13}C-NMR** (125 MHz, $CDCl_3$) δ = 167.3 (C_q), 159.7 (C_q), 153.0 (C_q), 140.0 (C_q), 139.3 (C_q), 134.7 (C_q), 131.8 (q,

$^2J_{C–F}$ = 32.6 Hz, C_q), 131.0 (C_q), 130.0 (CH), 129.1 (CH), 129.0 (CH), 128.7 (CH), 128.0 (CH), 127.0 (q, $^3J_{C–F}$ = 3.8 Hz, CH), 123.8 (q, $^3J_{C–F}$ = 3.7 Hz, CH), 123.7 (q, $^1J_{C–F}$ = 272.5 Hz, C_q), 120.4 (CH), 114.1 (CH), 55.4 (CH_3), 54.0 (CH_2), 51.8 (C_q), 27.3 (CH_3). **^{19}F-NMR** (376 MHz, $CDCl_3$) δ = –62.80 (s). **IR** (ATR): 3270, 2935, 1635, 1518, 1257, 1116, 837 cm^{-1}. **MS** (ESI) *m/z* (relative intensity): 1011 (10) [2M+Na]$^+$, 517 (60) [M+Na]$^+$, 495 (100) [M+H]$^+$. **HR-MS** (ESI) *m/z* calcd for $C_{27}H_{26}F_3N_4O_2$ [M+H]$^+$ 495.2002, found 495.2002.

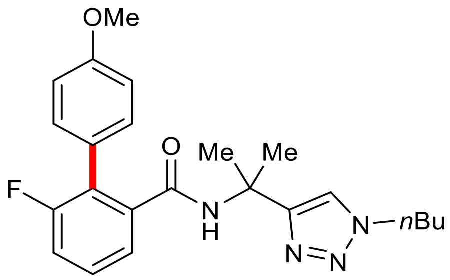

13

N-[2-(1-n-Butyl-1H-1,2,3-triazol-4-yl)propan-2-yl]-4-fluoro-4'-methoxy-[1,1'-biphenyl]-2-carboxamide (13)

The general procedure A was followed using **1k** (76.1 mg, 0.25 mmol) and **2a** (0.88 mL, 1.75 mmol, 2.0 M in THF). Purification by column chromatography on silica gel (*n*-hexane/EtOAc: 3:1→1:1) yielded **13** (54.4 mg, 53%) as a white solid. **M. p.** = 116–117 °C. **^{1}H-NMR** (400 MHz, $CDCl_3$) δ = 7.41 (dd, *J* = 7.6, 1.3 Hz, 1H), 7.32–7.20 (m, 4H), 7.15 (ddd, *J* = 9.4, 8.2, 1.3 Hz, 1H), 6.91 (d, *J* = 8.8 Hz, 2H), 5.90 (s_{br}, 1H), 4.24 (t, *J* = 7.3 Hz, 2H), 3.81 (s, 3H), 1.91–1.73 (m, 2H), 1.52 (s, 6H), 1.37–1.27 (m, 2H), 0.93 (t, *J* = 7.4 Hz, 3H). **^{13}C-NMR** (100 MHz, $CDCl_3$) δ = 166.9 (d, $^4J_{C–F}$ = 3.2 Hz, C_q), 159.7 (d, $^1J_{C–F}$ = 246.5 Hz, C_q), 159.6 (C_q), 152.6 (C_q), 138.8 (d, $^3J_{C–F}$ = 1.8 Hz, C_q), 131.0 (d, $^4J_{C–F}$ = 1.5 Hz, CH), 128.8 (d, $^3J_{C–F}$ = 8.6 Hz, CH), 126.9 (d, $^2J_{C–F}$ = 17.1 Hz, C_q), 125.3 (C_q), 124.1 (d, $^4J_{C–F}$ = 3.6 Hz, CH), 120.3 (CH), 117.2 (d, $^2J_{C–F}$ = 23.5 Hz, CH), 113.9 (CH), 55.3 (CH_3), 51.7 (C_q), 49.9 (CH_2), 32.2 (CH_2), 27.4 (CH_3), 19.7 (CH_2), 13.4 (CH_3). **^{19}F-NMR** (376 MHz, $CDCl_3$) δ = –115.10 (dd, *J* = 9.4, 5.1 Hz). **IR** (ATR): 3285, 2954, 1638, 1454, 1240, 838, 568 cm^{-1}. **MS** (ESI) *m/z* (relative intensity): 843 (12) [2M+Na]$^+$, 433 (96) [M+Na]$^+$, 411 (100) [M+H]$^+$. **HR-MS** (ESI) *m/z* calcd for $C_{23}H_{28}FN_4O_2$ [M+H]$^+$ 411.2191, found 411.2192.

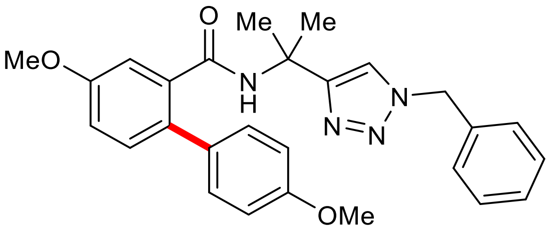

14

***N*-[2-(1-Benzyl-1*H*-1,2,3-triazol-4-yl)propan-2-yl]-4,4'-dimethoxy-[1,1'-biphenyl]-2-carboxamide (14)**

The general procedure A was followed using **1l** (87.6 mg, 0.25 mmol) and **2a** (0.88 mL, 1.75 mmol, 2.0 M in THF). Purification by column chromatography on silica gel (*n*-hexane/EtOAc: 3:1→1:1) yielded **14** (77.6 mg, 68%) as a white solid. **M. p.** = 120–121 °C. **^{1}H-NMR** (300 MHz, $CDCl_3$) δ = 7.42–7.30 (m, 3H), 7.30 (s, 1H), 7.30–7.13 (m, 6H), 6.97 (dd, J = 8.5, 2.8 Hz, 1H), 6.84 (d, J = 8.7 Hz, 2H), 5.81 (s_{br}, 1H), 5.46 (s, 2H), 3.84 (s, 3H), 3.80 (s, 3H), 1.54 (s, 6H). **^{13}C-NMR** (100 MHz, $CDCl_3$) δ = 167.9 (C_q), 158.9 (C_q), 158.6 (C_q), 153.1 (C_q), 136.8 (C_q), 134.7 (C_q), 132.3 (C_q), 131.7 (C_q), 131.4 (CH), 130.1 (CH), 129.0 (CH), 128.5 (CH), 127.9 (CH), 120.5 (CH), 116.5 (CH), 113.9 (CH), 113.0 (CH), 55.5 (CH_3), 55.4 (CH_3), 54.0 (CH_2), 51.6 (C_q), 27.4 (CH_3). **IR** (ATR): 3290, 1634, 1548, 1465, 1271, 1045 cm^{-1}. **MS** (ESI) *m/z* (relative intensity): 935 (10) $[2M+Na]^+$, 479 (100) $[M+Na]^+$, 457 (100) $[M+H]^+$. **HR-MS** (ESI) *m/z* calcd for $C_{27}H_{29}N_4O_3$ $[M+H]^+$ 457.2234, found 457.2236.

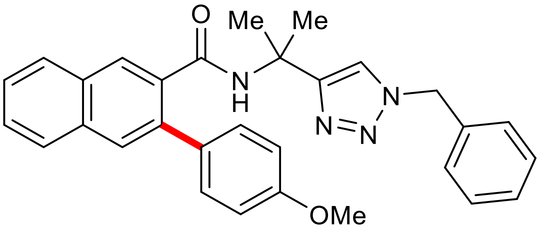

15

***N*-[2-(1-Benzyl-1*H*-1,2,3-triazol-4-yl)propan-2-yl]-3-(4-methoxyphenyl)-2-naphthamide (15)**

The general procedure A was followed using **1m** (92.6 mg, 0.25 mmol) and **2a** (0.88 mL,

1.75 mmol, 2.0 M in THF). Purification by column chromatography on silica gel (*n*-hexane/EtOAc: 3:1→1:1) yielded **15** (85.8 mg, 72%) as a white solid. **M. p.** = 161–162 °C. **^{1}H-NMR** (400 MHz, $CDCl_3$) δ = 8.12 (s, 1H), 7.86–7.83 (dd, J = 7.8, 2.1 Hz, 1H), 7.81–7.78 (dd, J = 7.8, 2.1 Hz, 1H), 7.73 (s, 1H), 7.53–7.44 (m, 2H), 7.38–7.31 (m, 6H), 7.26–7.21 (m, 2H), 6.87 (d, J = 8.8 Hz, 2H), 6.07 (s_{br}, 1H), 5.45 (s, 2H), 3.80 (s, 3H), 1.61 (s, 6H). **^{13}C-NMR** (100 MHz, $CDCl_3$) δ = 168.3 (C_q), 159.2 (C_q), 153.2 (C_q), 136.6 (C_q), 134.8 (C_q), 134.5 (C_q), 133.7 (C_q), 132.7 (C_q), 131.7 (C_q), 130.2 (CH), 129.1 (CH), 129.0 (CH), 128.7 (CH), 128.6 (CH), 128.2 (CH), 128.0 (CH), 127.6 (CH), 127.4 (CH), 126.4 (CH), 120.6 (CH), 113.9 (CH), 55.3 (CH_3), 54.0 (CH_2), 51.6 (C_q), 27.5 (CH_3). **IR** (ATR): 3269, 2024, 1966, 1634, 1543, 1455, 1241, 720 cm^{-1}. **MS** (ESI) *m/z* (relative intensity): 975 (12) $[2M+Na]^+$, 499 (70) $[M+Na]^+$, 477 (100) $[M+H]^+$. **HR-MS** (ESI) *m/z* calcd for $C_{30}H_{29}N_4O_2$ $[M+H]^+$ 477.2285, found 477.2285. The analytical data are in accordance to those reported in the literature.[5]

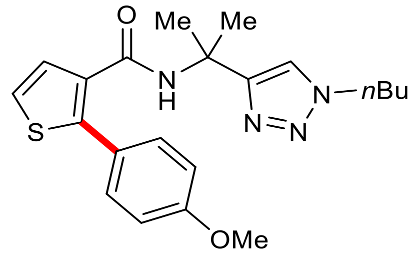

16

N-[2-(1-n-Butyl-1H-1,2,3-triazol-4-yl)propan-2-yl]-2-(4-methoxyphenyl)thiophene-3-carboxamide (16)

The general procedure A was followed using **1n** (73.1 mg, 0.25 mmol) **2a** (0.88 mL, 1.75 mmol, 2.0 M in THF). Purification by column chromatography on silica gel (*n*-hexane/EtOAc: 3:1→1:1) yielded **16** (77.7 mg, 78%) as a white solid. **M. p.** = 98–99 °C. **^{1}H-NMR** (400 MHz, $CDCl_3$) δ = 7.43–7.35 (m, 3H), 7.32 (d, J = 5.3 Hz, 1H), 7.14 (d, J = 5.3 Hz, 1H), 6.91 (d, J = 8.8 Hz, 2H), 6.00 (s_{br}, 1H), 4.24 (t, J = 7.3 Hz, 2H), 3.81 (s, 3H), 1.83 (m, 2H), 1.59 (s, 6H), 1.39–1.17 (m, 2H), 0.91 (t, J = 7.4 Hz, 3H). **^{13}C-NMR** (100 MHz, $CDCl_3$) δ = 163.2 (C_q), 160.2 (C_q), 152.6 (C_q), 143.7 (C_q), 133.8 (C_q), 131.0 (CH), 129.2 (CH), 124.8 (C_q), 123.7 (CH), 120.5 (CH), 114.2 (CH), 55.3 (CH_3), 51.3 (C_q), 49.9 (CH_2), 32.1 (CH_2), 27.6 (CH_3), 19.7 (CH_2), 13.4

(CH_3). **IR** (ATR): 3278, 2957, 2142, 1656, 1634, 1291, 1178, 829 cm^{-1}. **MS** (ESI) *m/z* (relative intensity): 819 (12) $[2M+Na]^+$, 421 (60) $[M+Na]^+$, 399 (100) $[M+H]^+$. **HR-MS** (ESI) *m/z* calcd for $C_{21}H_{27}N_4O_2S$ $[M+H]^+$ 399.1849, found 399.1851.

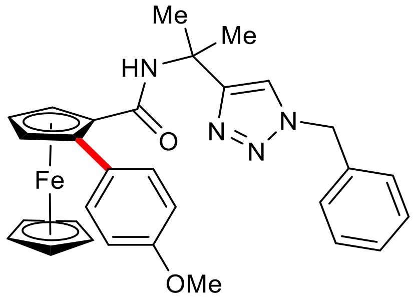

17

***N*-[2-(1-Benzyl-1*H*-1,2,3-triazol-4-yl)-propan-2-yl]-2-(4-methoxyphenyl)-ferrocenoylamide (17)**

The general procedure A was followed using **1o** (107.1 mg, 0.25 mmol) **2a** (0.88 mL, 1.75 mmol, 2.0 M in THF). Purification by column chromatography on silica gel (*n*-hexane/EtOAc: 3:1→1:1) yielded **17** (93.5 mg, 70%) as a white solid. **M. p.** = 137–138 °C. **^{1}H-NMR** (300 MHz, $CDCl_3$) δ = 7.49 (d, J = 8.7 Hz, 2H), 7.41 (s, 1H), 7.33 (m, 3H), 7.25 (dd, J = 5.6, 2.2 Hz, 2H), 6.86 (d, J = 8.7 Hz, 2H), 6.15 (s_{br}, 1H), 5.47 (s, 2H), 4.79 (dd, J = 2.5, 1.6 Hz, 1H), 4.36 (dd, J = 2.5, 1.6 Hz, 1H), 4.31 (dd, J = 2.5, 2.5 Hz, 1H), 4.15 (s, 5H), 3.81 (s, 3H), 1.63 (s, 3H), 1.57 (s, 3H). **^{13}C-NMR** (125 MHz, $CDCl_3$) δ = 168.9 (C_q), 158.9 (C_q), 153.2 (C_q), 134.8 (C_q), 131.6 (CH), 128.9 (CH), 128.5 (CH), 128.2 (C_q), 127.9 (CH), 120.6 (CH), 113.5 (CH), 88.3 (C_q), 76.3 (C_q), 72.6 (CH), 70.8 (CH), 70.2 (CH), 68.4 (CH), 55.4 (CH_3), 54.0 (C_q), 51.1 (CH_2), 28.2 (CH_3), 27.7 (CH_3). **IR** (ATR): 3337, 2956, 1638, 1517, 1294, 1048, 822 cm^{-1}. **MS** (ESI) *m/z* (relative intensity): 1091 (5) $[2M+Na]^+$, 557 (75) $[M+Na]^+$, 535 (100) $[M+H]^+$. **HR-MS** (ESI) *m/z* calcd for $C_{30}H_{31}FeN_4O_2$ $[M+H]^+$ 535.1791, found 535.1794. The analytical data are in accordance to those reported in the literature.[6]

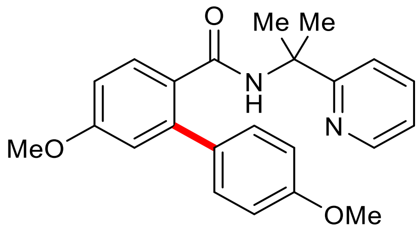

18

4',5-Dimethoxy-*N*-[2-(pyridin-2-yl)propan-2-yl]-[1,1'-biphenyl]-2-carboxamide (18)

The general procedure A was followed using **1p** (67.6 mg, 0.25 mmol) **2a** (0.88 mL, 1.75 mmol, 2.0 M in THF). Purification by column chromatography on silica gel (*n*-hexane/EtOAc: 3:1→1:1) yielded **18** (61.2 mg, 65%) as a white solid. **M. p.** = 88–89 °C. **^{1}H-NMR** (300 MHz, $CDCl_3$) δ = 8.30 (dd, J = 4.9, 1.8 Hz, 1H), 7.70 (d, J = 8.5, 1H), 7.60 (ddd, J = 8.1, 7.5, 1.8 Hz, 1H), 7.36 (d, J = 8.8 Hz, 2H), 7.29–7.18 (m, 2H), 7.07 (dd, J = 7.5, 4.9 Hz, 1H), 6.95–6.82 (m, 3H), 6.81 (d, J = 2.6 Hz, 1H), 3.83 (s, 3H), 3.74 (s, 3H), 1.59 (s, 6H). **^{13}C-NMR** (100 MHz, $CDCl_3$) δ = 167.8 (C_q), 164.2 (C_q), 160.3 (C_q), 159.2 (C_q), 147.3 (CH), 141.2 (C_q), 136.7 (CH), 132.9 (C_q), 130.7 (CH), 130.1 (CH), 129.3 (C_q), 121.4 (CH), 119.1 (CH), 115.4 (CH), 113.7 (CH), 112.4 (CH), 56.9 (C_q), 55.4 (CH_3), 55.3 (CH_3), 27.1 (CH_3). **IR** (ATR): 3290, 2959, 2956, 2108, 1990, 1637, 1540, 831 cm^{-1}. **MS** (ESI) *m/z* (relative intensity): 775 (10) [2M+Na]$^+$, 399 (14) [M+Na]$^+$, 377 (100) [M+H]$^+$. **HR-MS** (ESI) *m/z* calcd for $C_{23}H_{25}N_2O_3$ [M+H]$^+$ 377.1860, found 377.1861. The analytical data are in accordance to those reported in the literature.[7]

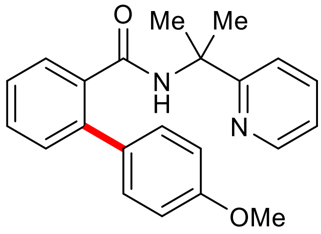

19

4'-Methoxy-*N*-[2-(pyridin-2-yl)propan-2-yl]-[1,1'-biphenyl]-2-carboxamide (19)

The general procedure A was followed using **1q** (60.1 mg, 0.25 mmol) **2a** (0.88 mL, 1.75 mmol, 2.0 M in THF). Purification by column chromatography on silica gel (*n*-hexane/EtOAc: 3:1→1:1) yielded **19** (50.2 mg, 58%) as a white solid. **M. p.** = 106–

107 °C. **^{1}H-NMR** (300 MHz, $CDCl_3$) δ = 8.31 (dd, J = 4.9, 1.8 Hz, 1H), 7.69 (dd, J = 7.4, 1.8 Hz, 1H), 7.61 (ddd, J = 8.1, 7.4, 1.8 Hz, 1H), 7.49–7.30 (m, 6H), 7.27–7.21 (m, 1H), 7.09 (ddd, J = 7.4, 4.9 Hz, 1H), 6.86 (d, J = 8.6 Hz, 2H), 3.75 (s, 3H), 1.62 (s, 6H). **^{13}C-NMR** (125 MHz, $CDCl_3$) δ = 168.3 (C_q), 164.1 (C_q), 159.0 (C_q), 147.3 (CH), 139.3 (C_q), 137.0 (C_q), 136.7 (CH), 132.9 (C_q), 130.2 (CH), 130.1 (CH), 129.5 (CH), 128.6 (CH), 127.0 (CH), 121.5 (CH), 119.1 (CH), 113.7 (CH), 57.0 (C_q), 55.3 (CH_3), 27.1 (CH_3). **IR** (ATR): 3293, 2988, 1640, 1542, 1474, 1240, 835 cm^{-1}. **MS** (ESI) *m/z* (relative intensity): 715 (5) $[2M+Na]^+$, 369 (4) $[M+Na]^+$, 347 (100) $[M+H]^+$. **HR-MS** (ESI) *m/z* calcd for $C_{22}H_{23}N_2O_2$ $[M+H]^+$ 347.1754, found 347.1756. The analytical data are in accordance to those reported in the literature.[7]

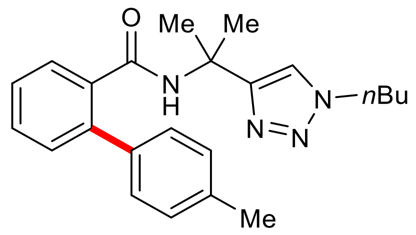

20

N-[2-(1-*n*-Butyl-1*H*-1,2,3-triazol-4-yl)propan-2-yl]-4'-methyl-[1,1'-biphenyl]-2-carboxamide (20)

The general procedure A was followed using **1a** (76.1 mg, 0.25 mmol) and **2b** (0.83 mL, 1.75 mmol, 2.1 M in THF). Purification by column chromatography on silica gel (*n*-hexane/EtOAc: 3:1→1:1) yielded **20** (102.0 mg, 77%) as a white solid. **M. p.** = 106–107 °C. **^{1}H-NMR** (400 MHz, $CDCl_3$) δ = 7.61 (dd, J = 7.5, 1.5 Hz, 1H), 7.40 (ddd, J = 7.5, 7.5, 1.5 Hz, 1H), 7.35–7.22 (m, 5H), 7.16 (d, J = 7.6 Hz, 2H), 5.86 (s_{br}, 1H), 4.24 (t, J = 7.3 Hz, 2H), 2.36 (s, 3H), 1.92–1.76 (m, 2H), 1.55 (s, 6H), 1.87–1.79 (m, 2H), 0.92 (t, J = 7.4 Hz, 3H). **^{13}C-NMR** (100 MHz, $CDCl_3$) δ = 168.4 (C_q), 152.7 (C_q), 139.6 (C_q), 137.4 (C_q), 137.3 (C_q), 136.2 (C_q), 130.1 (CH), 129.8 (CH), 129.1 (CH), 128.8 (CH), 128.5 (CH), 127.2 (CH), 120.4 (CH), 51.6 (C_q), 49.9 (CH_2), 32.2 (CH_2), 27.3 (CH_3), 21.1 (CH_3), 19.7 (CH_2), 13.4 (CH_3). **IR** (ATR): 3262, 3146, 2959, 1668, 1532, 1469, 1306, 756 cm^{-1}. **MS** (ESI) *m/z* (relative intensity): 775 (16) $[2M+Na]^+$, 399 (60) $[M+Na]^+$, 377 (100) $[M+H]^+$. **HR-MS** (ESI) *m/z* calcd for $C_{23}H_{29}N_4O$ $[M+H]^+$

377.2336, found 377.2332.

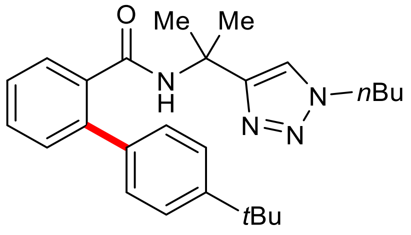

21

4'-(*tert*-Butyl)-*N*-[2-(1-*n*-butyl-1*H*-1,2,3-triazol-4-yl)propan-2-yl]-[1,1'-biphenyl]-2-carboxamide (21)

The general procedure A was followed using **1a** (76.1 mg, 0.25 mmol) and **2c** (0.88 mL, 1.75 mmol, 2.0 M in THF). Purification by column chromatography on silica gel (*n*-hexane/EtOAc: 3:1→1:1) yielded **21** (102.0 mg, 76%) as a white solid. **M. p.** = 136–137 °C. **^{1}H-NMR** (400 MHz, $CDCl_3$) δ = 7.66 (dd, J = 7.6, 1.5 Hz, 1H), 7.46–7.38 (m, 4H), 7.38–7.29 (m, 4H), 5.76 (s_{br}, 1H), 4.26 (t, J = 7.3 Hz, 2H), 1.93–1.73 (m, 2H), 1.49 (s, 6H), 1.38–1.28 (m, 11H), 0.94 (t, J = 7.4 Hz, 3H). **^{13}C-NMR** (100 MHz, $CDCl_3$) δ = 168.2 (C_q), 152.6 (C_q), 150.7 (C_q), 139.5 (C_q), 137.3 (C_q), 136.1 (C_q), 130.0 (CH), 129.9 (CH), 128.7 (CH), 128.6 (CH), 127.3 (CH), 125.4 (CH), 120.4 (CH), 51.4 (C_q), 49.9 (CH_2), 34.5 (C_q), 32.2 (CH_2), 31.3 (CH_3), 27.2 (CH_3), 19.7 (CH_2), 13.4 (CH_3). **IR** (ATR): 3314, 2963, 2867, 1635, 1541, 1318, 1046, 581 cm^{-1}. **MS** (ESI) *m/z* (relative intensity): 859 (20) $[2M+Na]^+$, 441 (52) $[M+Na]^+$, 419 (100) $[M+H]^+$. **HR-MS** (ESI) *m/z* calcd for $C_{26}H_{35}N_4O$ $[M+H]^+$ 419.2805, found 419.2801.

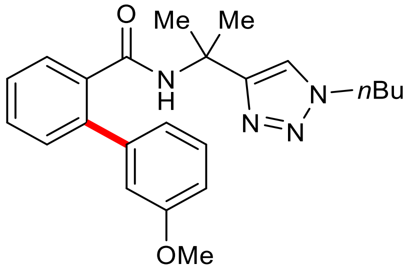

22

***N*-[2-(1-Butyl-1*H*-1,2,3-triazol-4-yl)propan-2-yl]-3'-methoxy-[1,1'-biphenyl]-2-carboxamide (22)**

The general procedure A was followed using **1a** (76.1 mg, 0.25 mmol) and **2d** (0.88 mL, 1.75 mmol, 2.0 M in THF). Purification by column chromatography on silica gel (*n*-hexane/EtOAc: 3:1→1:1) yielded **22** (102.0 mg, 98%) as a white solid. **M. p.** = 111–112 °C. **^{1}H-NMR** (300 MHz, $CDCl_3$) δ = 7.62 (dd, J = 7.3, 1.7 Hz, 1H), 7.46–7.33 (m, 2H), 7.33–7.23 (m, 3H), 6.99–6.81 (m, 3H), 5.85 (s_{br}, 1H), 4.24 (t, J = 7.3 Hz, 2H), 3.77 (s, 3H), 1.91–1.71 (m, 2H), 1.54 (s, 6H), 1.39–1.14 (m, 2H), 0.93 (t, J = 7.4 Hz, 3H). **^{13}C-NMR** (100 Hz, $CDCl_3$) δ = 168.0 (C_q), 159.4 (C_q), 152.6 (C_q), 141.7 (C_q), 139.4 (C_q), 136.2 (C_q), 129.8 (CH), 129.7 (CH), 129.4 (CH), 128.5 (CH), 127.5 (CH), 121.1 (CH), 120.2 (CH), 114.1 (CH), 113.5 (CH), 55.3 (CH_3), 51.6 (C_q), 49.9 (CH_2), 32.2 (CH_2), 27.4 (CH_3), 19.8 (CH_2), 13.5 (CH_3). **IR** (ATR): 2960, 2873, 1653, 1511, 1467, 1210, 759 cm^{-1}. **MS** (ESI) *m/z* (relative intensity): 807 (20) [2M+Na]$^+$, 415 (50) [M+Na]$^+$, 393 (100) [M+H]$^+$. **HR-MS** (ESI) *m/z* calcd for $C_{23}H_{29}N_4O_2$ [M+H]$^+$ 393.2285, found 393.2282.

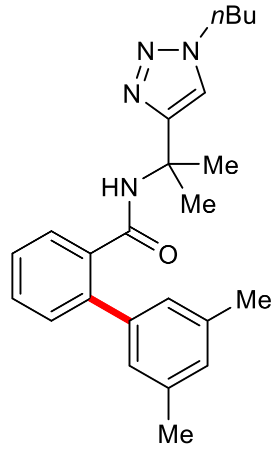

23

***N*-[2-(1-*n*-Butyl-1*H*-1,2,3-triazol-4-yl)propan-2-yl]-3',5'-dimethyl-[1,1'-biphenyl]-2-carboxamide (23)**

The general procedure A was followed using **1a** (76.1 mg, 0.25 mmol) and **2e** (0.76 mL, 1.75 mmol, 2.3 M in THF). Purification by column chromatography on silica gel (*n*-hexane/EtOAc: 3:1→1:1) yielded **23** (102.0 mg, 84%) as a white solid. **M. p.** = 112–113 °C. **^{1}H-NMR** (400 MHz, $CDCl_3$) δ = 7.65 (dd, J = 7.4, 1.5 Hz, 1H), 7.41 (ddd, J = 7.4, 7.4, 1.5 Hz, 1H), 7.37–7.28 (m, 3H), 7.01 (d, J = 0.7 Hz, 2H), 7.00 (s, 1H), 5.86

(s_{br}, 1H), 4.26 (t, *J* = 7.3 Hz, 2H), 2.32 (s, 6H), 1.99–1.75 (m, 2H), 1.53 (s, 6H), 1.40–1.21 (m, 2H), 0.94 (t, *J* = 7.4 Hz, 3H). **^{13}C-NMR** (100 MHz, $CDCl_3$) δ = 168.2 (C_q), 152.7 (C_q), 140.3 (C_q), 139.9 (C_q), 138.0 (C_q), 136.0 (C_q), 130.0 (CH), 129.8 (CH), 129.1 (CH), 128.6 (CH), 127.3 (CH), 126.7 (CH), 120.3 (CH), 51.5 (C_q), 49.9 (CH_2), 32.2 (CH_2), 27.3 (CH_3), 21.2 (CH_3), 19.7 (CH_2), 13.4 (CH_3). **IR** (ATR): 3243, 2951, 2975, 1659, 1525, 1300, 1194, 1056 cm^{-1}. **MS** (ESI) *m/z* (relative intensity): 803 (15) $[2M+Na]^+$, 413 (28) $[M+Na]^+$, 391 (100) $[M+H]^+$. **HR-MS** (ESI) *m/z* calcd for $C_{24}H_{31}N_4O$ $[M+H]^+$ 391.2492, found 391.2492.

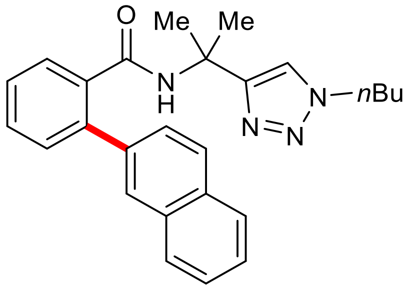

24

***N*-[2-(1-*n*-Butyl-1*H*-1,2,3-triazol-4-yl)propan-2-yl]-2-(naphthalen-2-yl)benzamide (24)**

The general procedure A was followed using **1a** (76.1 mg, 0.25 mmol) and **2f** (0.80 mL, 1.75 mmol, 2.2 M in THF). Purification by column chromatography on silica gel (*n*-hexane/EtOAc: 3:1→1:1) yielded **24** (102.0 mg, 88%) as a white solid. **M. p.** = 140–141 °C. **^{1}H-NMR** (400 MHz, $CDCl_3$) δ = 7.89–7.77 (m, 4H), 7.65 (dd, *J* = 7.6, 1.5 Hz, 1H), 7.51–7.43 (m, 4H), 7.4–7.35 (m, 2H), 6.97 (s, 1H), 5.94 (s_{br}, 1H), 4.0 (t, *J* = 7.3 Hz, 2H), 1.71–1.60 (m, 2H), 1.50 (s, 6H), 1.33–1.14 (m, 2H), 0.88 (t, *J* = 7.4 Hz, 3H). **^{13}C-NMR** (100 MHz, $CDCl_3$) δ = 168.3 (C_q), 152.6 (C_q), 139.5 (C_q), 137.9 (C_q), 136.5 (C_q), 133.1 (C_q), 132.4 (C_q), 130.4 (CH), 129.9 (CH), 128.5 (CH), 128.0 (CH), 127.9 (CH), 127.5 (CH), 127.5 (CH), 127.5 (CH), 126.3 (CH), 126.1 (CH), 120.0 (CH), 51.7 (C_q), 49.7 (CH_2), 32.0 (CH_2), 27.1 (CH_3), 19.6 (CH_2), 13.4 (CH_3). **IR** (ATR): 3247, 3055, 2966, 1634, 1558, 1322, 1051, 754 cm^{-1}. **MS** (ESI) *m/z* (relative intensity): 847 (15) $[2M+Na]^+$, 435 (52) $[M+Na]^+$, 413 (100) $[M+H]^+$. **HR-MS** (ESI) *m/z* calcd for $C_{26}H_{29}N_4O$ $[M+H]^+$ 413.2336, found 413.2333.

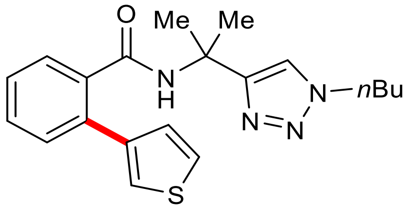

25

***N*-[2-(1-*n*-Butyl-1*H*-1,2,3-triazol-4-yl)propan-2-yl]-2-(thiophen-3-yl)benzamide (25)**

The general procedure A was followed using **1a** (76.1 mg, 0.25 mmol) and **2g** (0.88 mL, 1.75 mmol, 2.0 M in THF). Purification by column chromatography on silica gel (*n*-hexane/EtOAc: 3:1→1:1) yielded **25** (102.0 mg, 63%) as a white solid. **M. p.** = 116–117 °C. **^{1}H-NMR** (400 MHz, $CDCl_3$) δ = 7.63 (dd, J = 6.6, 1.6 Hz, 1H), 7.51–7.31 (m, 6H), 7.16 (dd, J = 4.6, 1.6 Hz, 1H), 6.06 (s_{br}, 1H), 4.32 (t, J = 7.3 Hz, 2H), 2.01–1.80 (m, 2H), 1.68 (s, 6H), 1.36 (dt, J = 14.7, 7.4 Hz, 2H), 0.98 (t, J = 7.4 Hz, 3H). **^{13}C-NMR** (100 MHz, $CDCl_3$) δ = 168.4 (C_q), 152.6 (C_q), 140.5 (C_q), 136.3 (C_q), 134.0 (C_q), 129.8 (CH), 129.7 (CH), 128.7 (CH), 128.3 (CH), 127.5 (CH), 125.6 (CH), 123.0 (CH), 120.4 (CH), 51.6 (C_q), 49.9 (CH_2), 32.1 (CH_2), 27.4 (CH_3), 19.7 (CH_2), 13.4 (CH_3). **IR** (ATR): 3297, 3149, 2929, 2874, 1633, 1543, 1195, 751, 627 cm^{-1}. **MS** (ESI) *m/z* (relative intensity): 759 (22) $[2M+Na]^+$, 391 (52) $[M+Na]^+$, 369 (100) $[M+H]^+$. **HR-MS** (ESI) *m/z* calcd for $C_{20}H_{25}N_4OS$ $[M+H]^+$ 369.1744, found 369.1742.

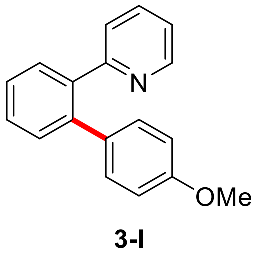

3-I

2-(4'-Methoxy-[1,1'-biphenyl]-2-yl)pyridine (3-I)

The general procedure A was followed using 2-phenylpyridine (38.8 mg, 0.25 mmol) and **2a** (0.88 mL, 1.75 mmol, 2.0 M in THF). Purification by column chromatography on silica gel (*n*-hexane/EtOAc: 20:1→10:1) yielded **3-I** (18.3 mg, 28%) as a colorless

oil. The analytical data are in accordance to those reported in the literature.[8]

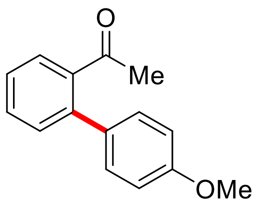

3-II

1-(4'-Methoxy-[1,1'-biphenyl]-2-yl)ethan-1-one (3-II)

The general procedure A was followed using *N*-(4-methoxyphenyl)-1-phenylethan-1-imine (56.3 mg, 0.25 mmol) and **2a** (0.88 mL, 1.75 mmol, 2.0 M in THF). Purification by column chromatography on silica gel (*n*-hexane/EtOAc: 20:1→10:1) yielded **3-II** (17.0 mg, 30%) as a colorless oil. The analytical data are in accordance to those reported in the literature.[9]

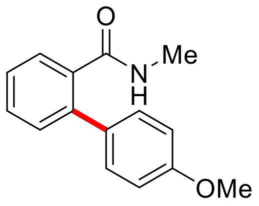

3-III

4'-Methoxy-*N*-methyl-[1,1'-biphenyl]-2-carboxamide (3-III)

The general procedure A was followed using *N*-methylbenzamide (33.8 mg, 0.25 mmol) and **2a** (0.88 mL, 1.75 mmol, 2.0 m in THF). Purification by column chromatography on silica gel (*n*-hexane/EtOAc: 20:1→10:1) yielded **3-III** (15.1 mg, 25%) as a colorless oil. The analytical data are in accordance to those reported in the literature.[10]

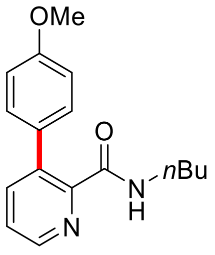

26

***N*-*n*-Butyl-3-(4-methoxyphenyl)picolinamide (26)**

The general procedure B was followed using **S-1a** (44.5 mg, 0.25 mmol) and **2a** (0.50 mL, 1.0 mmol, 2.0 M in THF). Purification by column chromatography on silica gel (*n*-hexane/EtOAc: 2:1→1:1) yielded **26** (49.8 mg, 70%) as a white solid. **M. p.** = 96–97 °C. **^{1}H-NMR** (300 MHz, $CDCl_3$) δ = 8.48 (dd, J = 4.6, 1.7 Hz, 1H), 7.66–7.62 (m, 2H), 7.38 (dd, J = 7.8, 4.6 Hz, 1H), 7.25 (d, J = 8.8 Hz , 2H), 6.92 (d, J = 8.8 Hz, 2H), 3.81 (s, 3H), 3.34 (td, J = 7.2, 6.0 Hz, 2H), 1.60–1.44 (m, 2H), 1.42–1.23 (m, 2H), 0.91 (t, J = 7.2 Hz, 3H). **^{13}C-NMR** (75 MHz, $CDCl_3$) δ = 165.2 (C_q), 159.0 (C_q), 148.2 (C_q), 146.4 (CH), 139.9 (CH), 137.7 (C_q), 131.6 (C_q), 129.5 (CH), 124.9 (CH), 113.4 (CH), 55.2 (CH_3), 39.2 (CH_2), 31.7 (CH_2), 20.2 (CH_2), 13.8 (CH_3). **IR** (ATR): 3330, 2324, 2181, 2032, 514, 478, 455, 432 cm^{-1}. **MS** (EI) *m/z* (relative intensity): 284 (55) $[M]^+$, 242 (20), 227 (70), 213 (50), 185 (100), 169 (32). **HR-MS** (ESI) *m/z* calcd for $C_{17}H_{21}N_2O_2$ $[M+H]^+$ 285.1598, found 285.1597. The analytical data are in accordance to those reported in the literature.[4]

Comparision of Electrochemical Oxidation versus Chemical Oxidation

General procedure for chemical oxidation C–H arylation: A solution of ArMgBr (2.10 mL, 7.0 equiv, 1.0 M in THF) was slowly added to a mixture of amide **1** (0.3 mmol) and $ZnBr_2$•TMEDA (308 mg, 3.00 equiv) under N_2. The resulting mixture was stirred at ambient temperature for 10 min, then a solution of $FeCl_3$ (4.9 mg, 10 mol %) and dppe (11.9 mg, 10 mol %) in THF (1.5 mL) was added. The reaction mixture was stirred at ambient temperature for 10 min and then DCIB (76.2 mg, 2.0 equiv) was added. The mixture was stirred at 55 °C (t = 0 min). At ambient temperature, saturated aqueous NH_4Cl solution (10 mL) was added, the reaction mixture was extracted with EtOAc (3 × 10 mL) and dried over Na_2SO_4. Evaporation of the solvents and purification by column chromatography on silica gel (*n*-Hexane/EtOAc 3:1→1:1) the desired product **9**.

Kinetic Studies by ^{19}F-NMR

General procedure for chemical oxidation C–H arylation: A solution of ArMgBr (2.10 mL, 7.0 equiv, 1.0 M in THF) was slowly added to a mixture of amide **1** (0.3 mmol) and $ZnBr_2$•TMEDA (308 mg, 3.00 equiv) under N_2. The resulting mixture was stirred at ambient temperature for 10 min, then a solution of $FeCl_3$ (4.9 mg, 10 mol %) and dppe (11.9 mg, 10 mol %) in THF (1.5 mL) was added. The reaction mixture was stirred at ambient temperature for 10 min and then DCIB (76.2 mg, 2.0 equiv) was added. The mixture was stirred at 55 °C ($t = 0$ min). Aliquots (100 µL) were removed via a syringe periodically every 5 and 10 min. The conversion was determined by ^{19}F-NMR using *n*-nonyl fluoride (54 µL, 0.3 mmol) as the internal standard.

General procedure for electrochemical oxidation C–H arylation: The electrocatalysis was carried out in an undivided cell with a RVC anode (10 mm × 15 mm × 6 mm) and a platinum cathode (10 mm × 15 mm × 0.25 mm). A solution of ArMgBr (0.88 mL, 7.0 equiv, 2.0 M in THF) was slowly added to a mixture of amide **1** (0.25 mmol, 1.00 equiv), $Fe(acac)_3$ (8.8 mg, 10 mol %), dppe (10.0 mg, 10 mol %) and $ZnCl_2$·TMEDA (189 mg, 3.00 equiv) were placed in a 10 mL cell and dissolved in THF (5 mL). Electrolysis was performed at 40 °C with a constant current of 5 mA ($t = 0$ min). Aliquots (300 µL) were removed periodically every 5 and 10 min. The conversion was determined by ^{19}F-NMR using *n*-nonyl fluoride (45 µL, 0.25 mmol) as the internal standard.

Each reaction was performed three times, the measured conversions were averaged and the error corresponds to the standard deviation.

Chemical oxidant

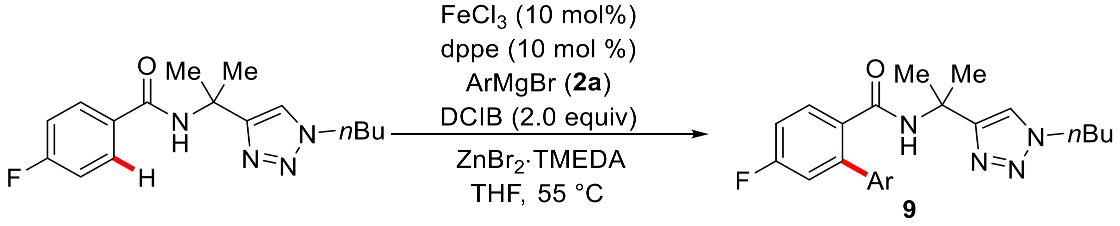

Electrochemical reaction

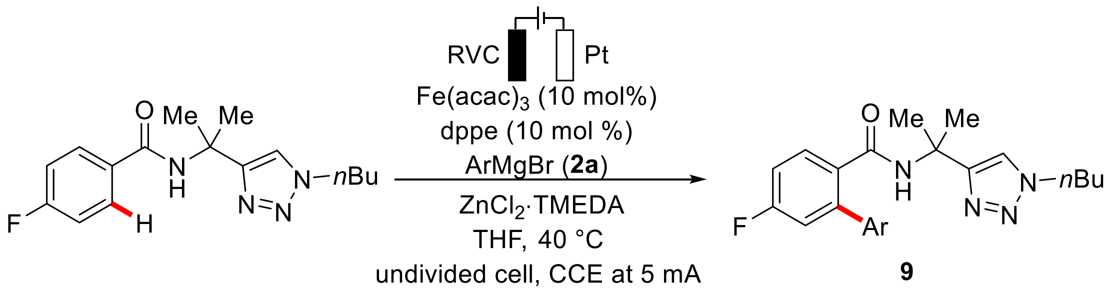

NMR conv. (%) / Reaction \ *t* (min)		5	10	20	30	40	50	60
Chemical oxidation	1st	3	4	11	12	19	21	25
	2nd	2	3	11	14	18	20	25
	3rd	4	5	8	10	16	20	24
Electrochemical oxidation	1st	10	12	23	35	40	45	59
	2nd	15	17	22	30	39	45	56
	3rd	14	16	25	30	37	41	51

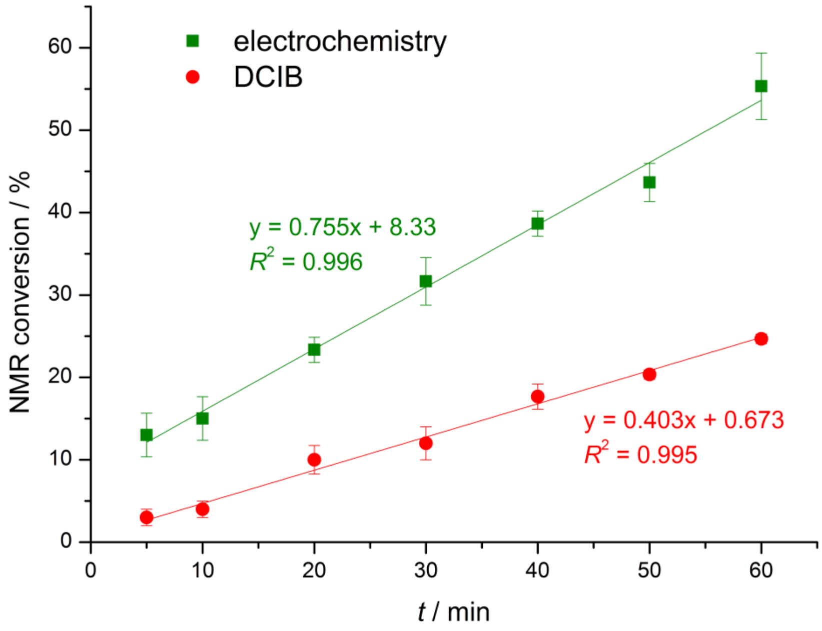

Figure S1. Comparision of electrochemical *versus* chemical oxidation reaction of **9**.

Chemical oxidant

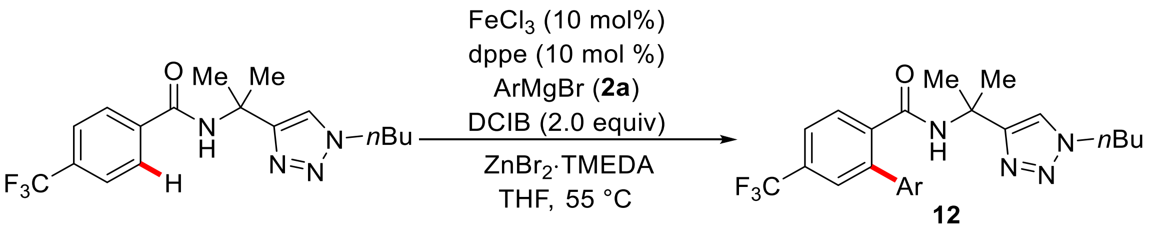

Electrochemical reaction

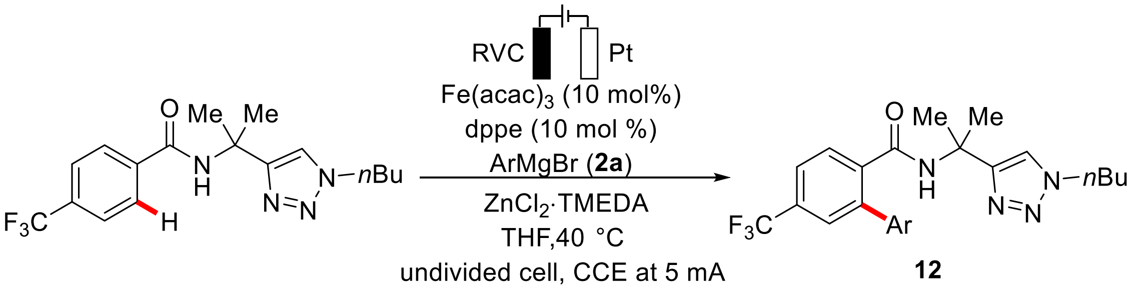

Reaction \ NMR conv. (%) \ *t* (min)		5	10	20	30	40	50	60
Chemical oxidation	1st	5	8	12	13	16	16	16
	2nd	5	8	12	14	18	18	19
	3rd	6	6	13	16	20	21	22
Electrochemical oxidation	1st	11	18	21	24	32	37	43
	2nd	14	22	30	32	37	39	41
	3rd	16	25	22	29	33	39	40

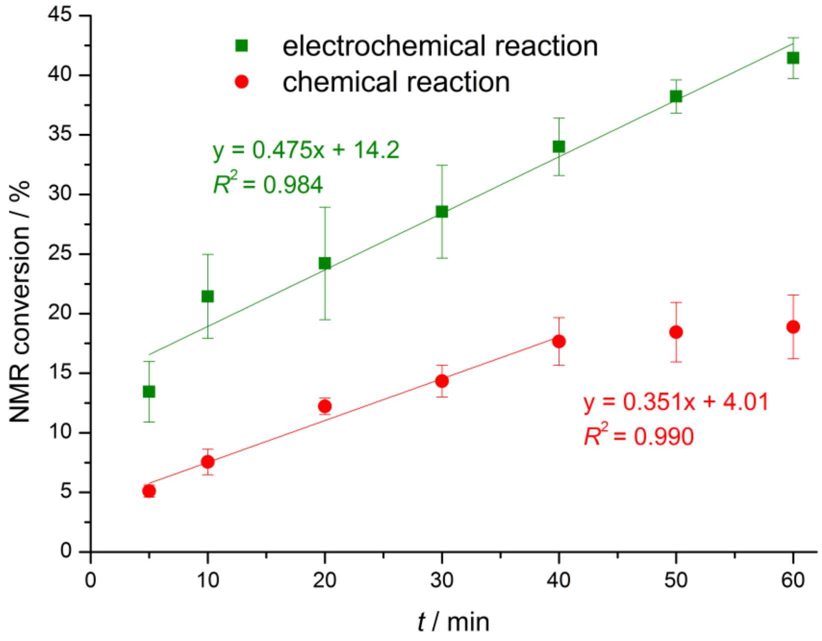

Figure S2. Comparision of electrochemical *versus* chemical oxidation reaction rate of **12**.

Intermolecular Competition Experiment

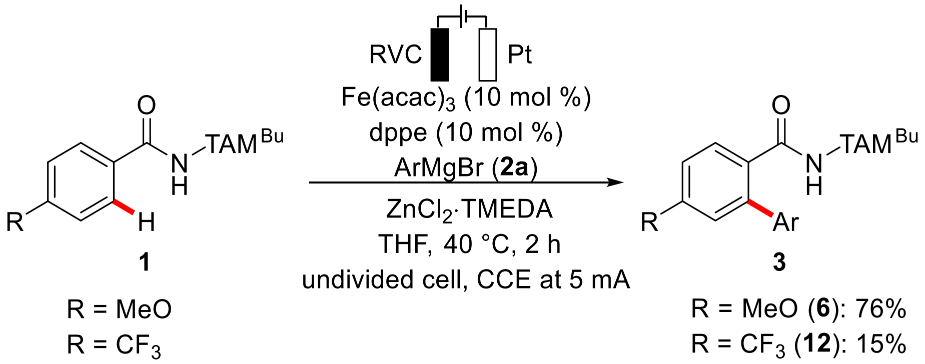

A solution of ArMgBr (0.88 mL, 7.0 equiv, 2.0 M in THF) was slowly added to a mixture of amide **1d** (79.1 mg, 0.25 mmol) and **1j** (88.6 mg, 0.25 mmol), $Fe(acac)_3$ (8.8 mg, 10 mol %), dppe (10.0 mg, 10 mol %) and $ZnCl_2$·TMEDA (189 mg, 3.00 equiv) were placed in a 10 mL cell and dissolved in THF (5 mL). Electrolysis was performed at 40 °C with a constant current of 5 mA maintained for 2 h. At ambient temperature, a saturated aqueous NH_4Cl solution (10 mL) was added and the RVC anode was washed with EtOAc (3 × 2 mL) in an ultrasonic bath. The combined phases were extracted with EtOAc (3 × 10 mL) and then dried over Na_2SO_4. Evaporation of

the solvents and purification by column chromatography on silica gel (*n*-hexane/EtOAc 3:1→1:1) yielded **6** (80.3 mg, 76%) and **12** (17.3 mg, 15%).

Kinetic Isotope Effect Studies

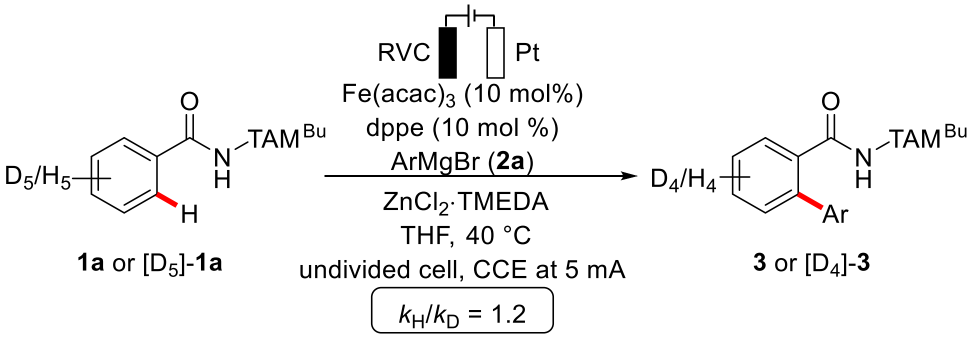

Five parallel independent reactions of **1a** or [D_5] **-1a** with **2a** were performed to determine the KIE. A solution of ArMgBr (0.88 mL, 7.0 equiv, 2.0 M in THF) was slowly added to a mixture of amide **1a** (76.1 mg, 0.25 mmol) and [D_5]-**1a** (72.9 mg, 0.25 mmol), $Fe(acac)_3$ (8.8 mg, 10 mol %), dppe (10.0 mg, 10 mol %) and $ZnCl_2$·TMEDA (189 mg, 3.00 equiv) were placed in a 10 mL cell and dissolved in THF (5 mL). Electrolysis was performed at 40 °C with a constant current of 5 mA. At ambient temperature, a saturated aqueous NH_4Cl solution (10 mL) was added and the RVC anode was washed with EtOAc (3 × 2 mL) in an ultrasonic bath. The combined phases were extracted with EtOAc (3 × 10 mL) and then dried over Na_2SO_4. Evaporation of the solvents and purification by column chromatography on silica gel (*n*-hexane/EtOAc 3:1→1:1), Then ^{1}H-NMR conversions were obtained by the use of CH_2Br_2 (17 μL, 0.25 mmol) as the standard.

t (min)	15	30	45	60	75	90
**3** (%)	9	19	25	32	38	45
[D_4]-**3** (%)	5	12	19	24	28	36

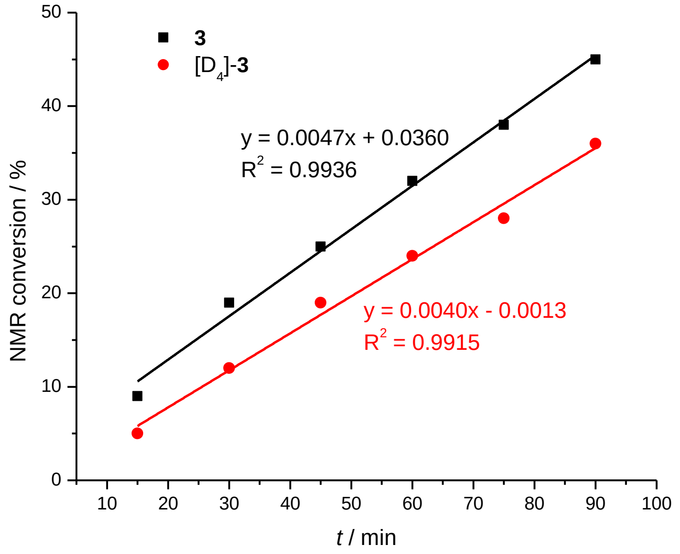

Figure S3. Kinetic profile with substrates **1a** or [D_5]-**1a**.

Cyclic Voltammetry

The cyclic voltammetry was carried out with a Metrohm Autolab PGSTAT204 workstation and following analysis was performed with Nova 2.0 software. A glassy-carbon electrode (3 mm diameter, disc-electrode) was used as the working electrode, a Pt wire was employed as the auxiliary electrode and a Ag/Ag^+ electrode was used as a quasi-reference electrode with ferrocene as an internal standard. The measurements were carried out at a scan rate of 100 mV s^{-1}. Arrows in cyclic voltammograms indicate scanning direction and the starting potential.

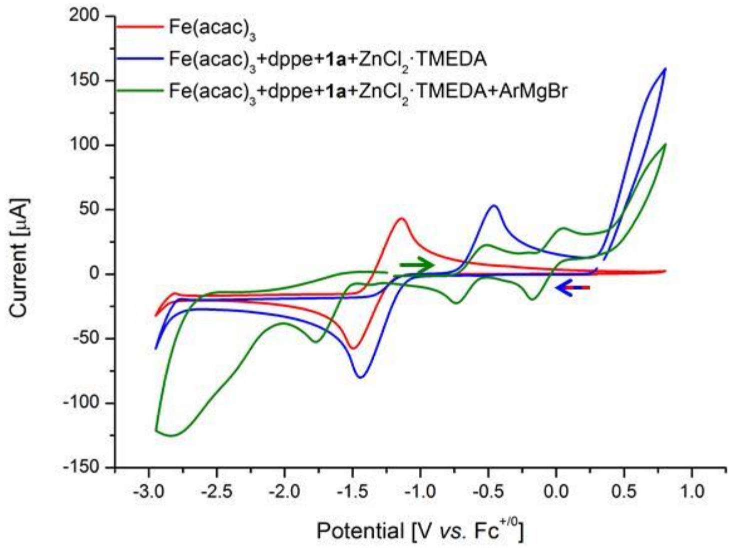

Figure S4. Cyclic voltammograms of reactants at 100 mV $s^{-1}$with $n$$Bu_4NPF_6$ (0.1 M in THF); concentrations of substrates 5 mM (Grignard 20 mM). $Fe(acac)_3$ (red), $Fe(acac)_3$ + dppe + **1** + $ZnCl_2$·TMEDA (blue), $Fe(acac)_3$ + dppe + **1** + $ZnCl_2$·TMEDA + 4-$MeOC_6H_4MgBr$ (green).

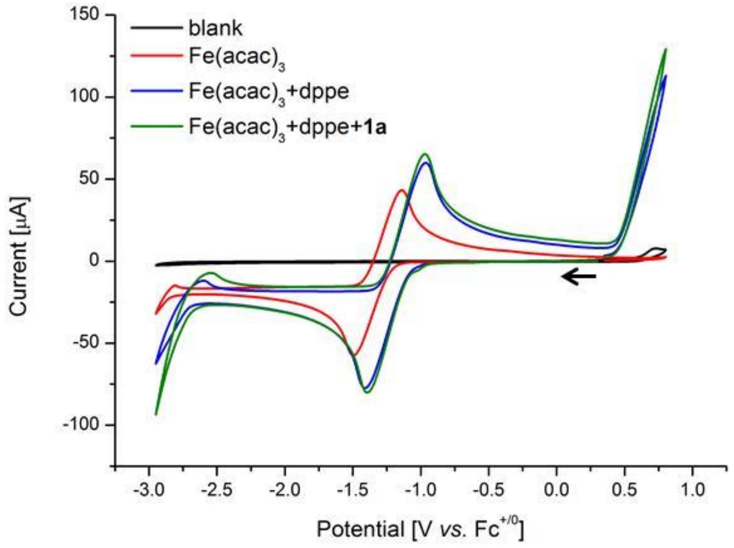

Figure S5. Cyclic voltammograms of reactants at 100 mV s^{-1} with nBu$_4$NPF$_6$ (0.1 M in THF); concentrations of substrates 5 mM. Fe(acac)$_3$ (red), Fe(acac)$_3$ + dppe (blue), Fe(acac)$_3$ + dppe + **1** (green).

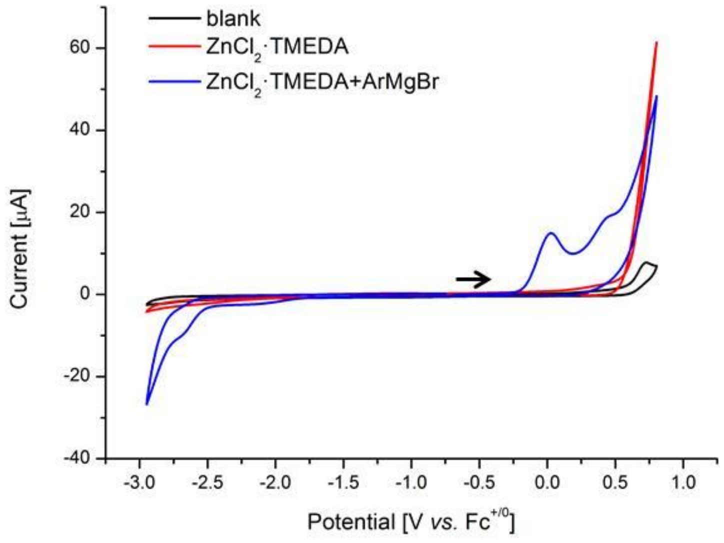

Figure S6. Cyclic voltammograms of reactants at 100 mV s^{-1} with nBu$_4$NPF$_6$ (0.1 M in THF); concentrations of substrates 5 mM (Grignard 20 mM). blank (black), ZnCl$_2$·TMEDA (red), ZnCl$_2$·TMEDA + 4-MeOC$_6$H$_4$MgBr (blue).

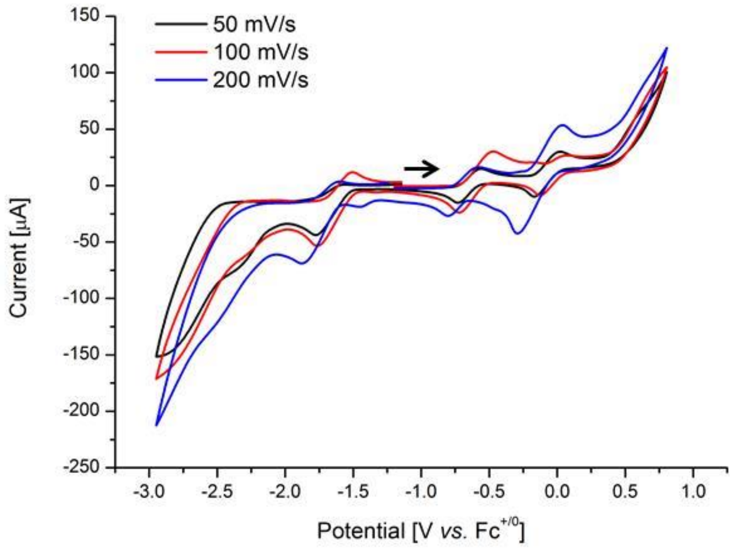

Figure S7. Cyclic voltammograms of reactants at 100 mV s^{-1} with *n*Bu_4NPF_6 (0.1 M in THF); concentrations of substrates 5 mM (Grignard 20 mM). Fe(acac)$_3$ + dppe + **1** + $ZnCl_2$·TMEDA + 4-$MeOC_6H_4MgBr$. 50 mV s^{-1} (black), 100 mV s^{-1} (red), 200 mV s^{-1} (blue).

SEM Analysis

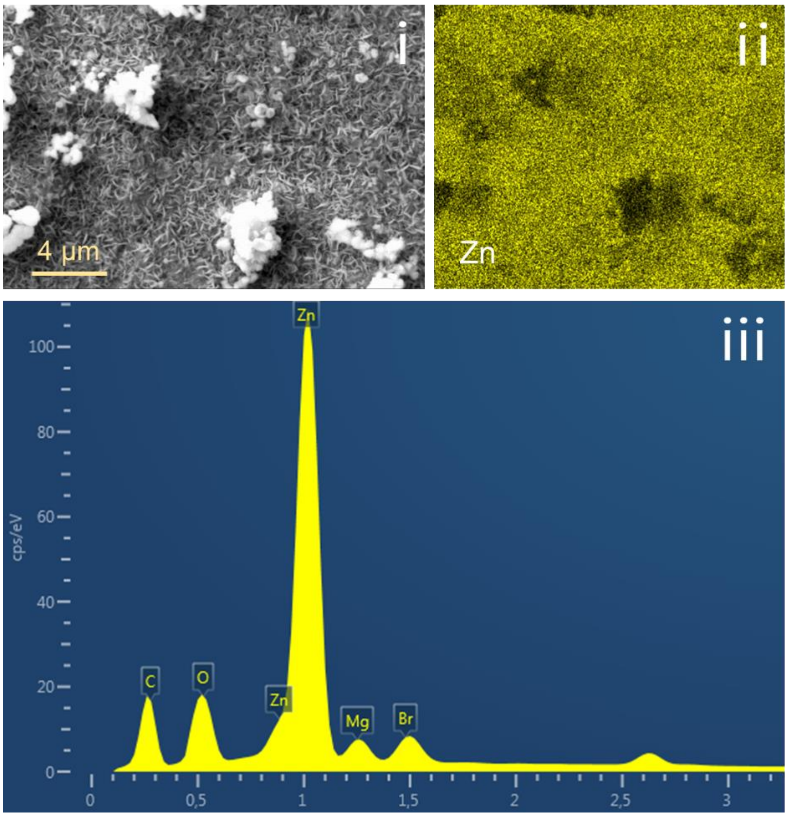

Figure S8. SEM studies on the post-catalysis cathode materials. i) SEM image of deposition. ii) SEM-EDS mapping revealing the location of zinc. iii) Elemental distribution.

Plausible Catalytic Cycle

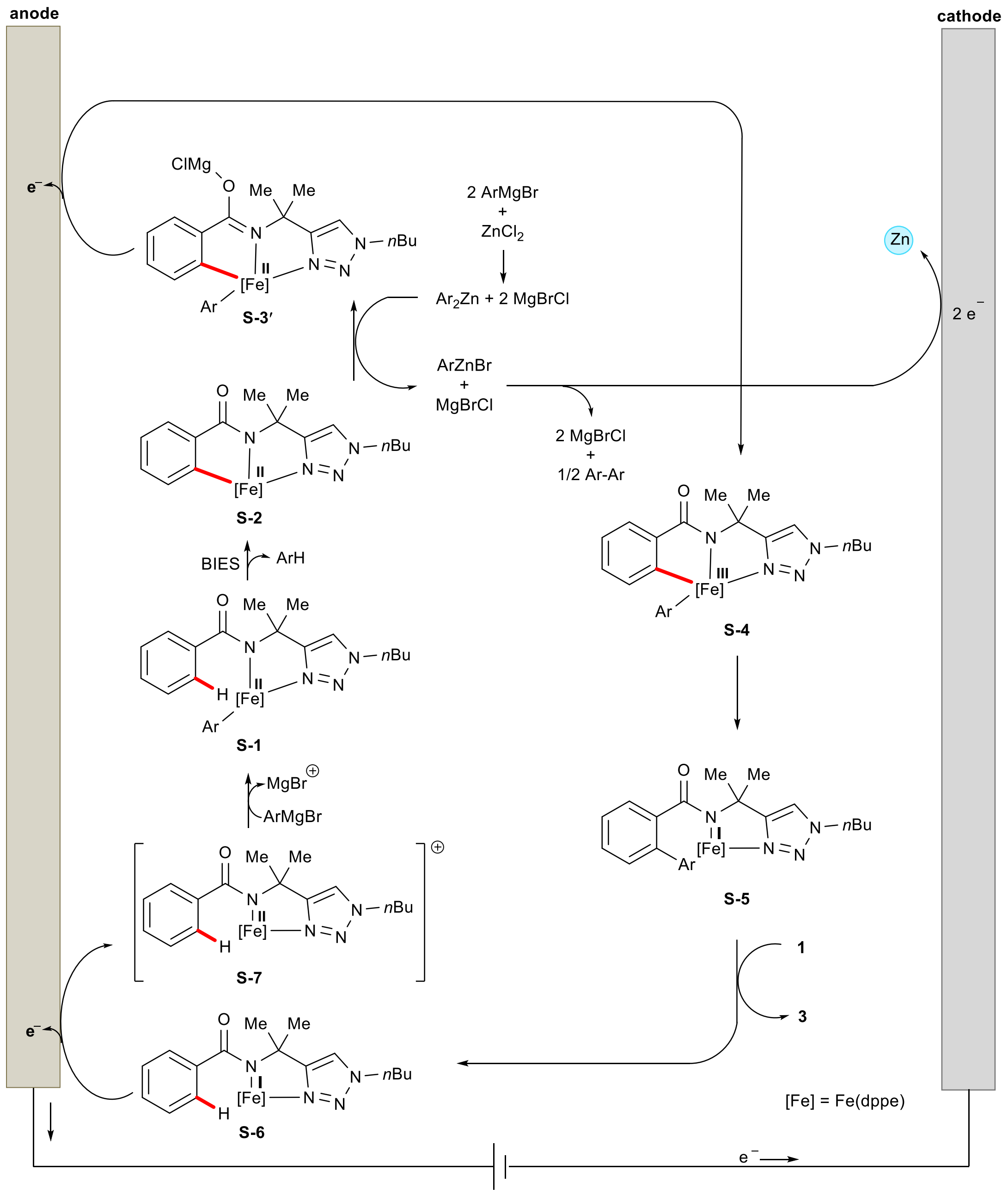

Figure S9. The plausible catalytic cycle.

Gram-Scale Synthesis of 3

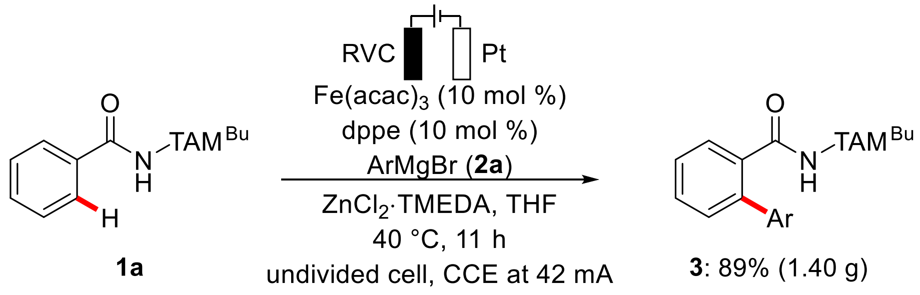

Benzamide **1a** (1.15 g, 4.00 mmol, 1.00 equiv), Fe(acac)$_3$ (141 mg, 10 mol %), dppe (160 mg, 10 mol %), $ZnCl_2$·TMEDA (3.02 g, 3.00 equiv) and **2a** (14.0 mL, 7.0 equiv, 2.0 M in THF) were placed in an undivided cell (100 mL) with a RVC anode (25 mm × 50 mm × 6 mm) and a platinum cathode (25 mm × 50 mm × 0.25 mm) and dissolved in THF (50 mL). Electrocatalysis was performed at 40 °C with a constant current of 41.7 mA maintained for 11.6 h (4.51 F/mol). At ambient temperature, saturated aqueous NH_4Cl (10 mL) was added, and the RVC anode was washed with EtOAc (3 × 10 mL) in an ultrasonic bath. The combined phases were extracted with EtOAc (3 × 30 mL) and then dried over Na_2SO_4. Evaporation of the solvents and purification by column chromatography (*n*-hexane/EtOAc = 3:1→1:1) yielded **3** (1.40 g, 89%) as a white solid.

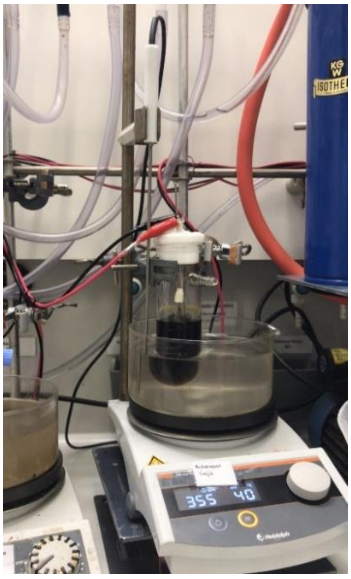

Figure S10. Electrolysis setup for gram-scale.

Computational Studies

All structures were optimized with the meta-GGA functional TPSS[11] in combination with Grimme's D3 dispersion corrections with the Becke-Johnson damping scheme (D3BJ).[12] All atoms were described with a def2-SVP basis set.[13] Analytical frequency calculations were carried out at the same level of theory to identify the nature of the stationary points either as intermediates (no imaginary frequencies) or transition states (only one imaginary frequency), and to provide thermal as well as non-thermal corrections to the free energy in gas-phase at 313.15 K and 1 atm. The electronic energy was then refined through PW6B95[14] single-point calculations on the optimized geometries in combination with dispersion corrections with a def2-TZVP basis set.[13] Solvent effects were included implicitly through the use of the Solvation Model Based on Density (SMD)[15] with a dielectric constant of ε = 7.4257, which corresponds to tetrahydrofuran (THF), the solvent of choice used in the experimental work. Unless otherwise stated, the energies herein provided are based on gas-phase Gibbs free energies with def2-SVP basis set for which the electronic energies were improved at the PW6B95-D3BJ/def2-TZVP+SMD(THF) level of theory. Open-shell systems were calculated under the unrestricted formalism.

All calculations were performed using Gaussian 16, Revision A.03 package.[16] 3D structure images were created with PyMOL version 1.8.x.[17]

The oxidation potential was calculated as follows:[18]

$$E_{1/2}^{\mathrm{o,calc}} = -\frac{\Delta G_{1/2}^{\mathrm{o}}}{n_e F} - E_{1/2}^{\mathrm{o,SHE}} - E^{\mathrm{o,SCE}} - \Delta E^{\mathrm{o,Fc}} \quad (1)$$

With $\Delta G_{1/2}^{\mathrm{o}}$ defined as:

$$\Delta G_{1/2}^{\mathrm{o}} = G_{313.15}^{n} - G_{313.15}^{ox} \quad (2)$$

Where $G_{313.15}^{n}$ and $G_{313.15}^{ox}$ is the Gibbs free energy from the neutral and oxidized species, respectively.

In Eq. 1, n_e is the number of electrons transferred during the oxidation process, F is the Faraday constant (23.061 kcal mol^{-1}V^{-1}), $E_{1/2}^{\mathrm{o,SHE}}$ is the absolute value of the standard hydrogen electrode (4.281 V), and $E^{\mathrm{o,SCE}}$ is the saturated calomel electrode

relative to SHE in sat. KCl (0.244 V). The final term in equation 1, $\Delta E^{\mathrm{o,Fc}}$, is the oxidation potential of ferrocene relative to SCE in THF (0.560 V)[19] in the presence of nBu$_4$NPF$_6$ which is the same electrolyte used in the cyclic voltammetry experiments.

Table S4. Calculated electronic energies at PW6B95-D3BJ/def2-TZVP+SMD(THF) level of theory and Gibbs free energies with dispersion corrections for all structures in the present work (all in Hartree).[a]

**Structure**	**Electronic energy**	**Total Gibbs Free Energy**
^{1}S-1	-4219.652135	-4218.857158
^{3}**S-1**	-4219.652939	-4218.864472
^{5}**S-1**	-4219.654767	-4218.874944
^{1}**TS(1-2)**	-4219.631509	-4218.840215
^{3}**TS(1-2)**	-4219.602748	-4218.819675
^{5}**TS(1-2)**	-4219.603176	-4218.822325
^{1}**S-2**	-3872.308870	-3871.637897
^{3}**S-2**	-3872.311773	-3871.645912
^{5}**S-2**	-3872.302284	-3871.640110
^{2}**S-3**	-3872.167978	-3871.498882
^{4}**S-3**	-3872.174247	-3871.506083
^{6}**S-3**	-3872.151810	-3871.488391
^{1}**S-3'**	-4219.142075	-4218.361625
^{3}**S-3'**	-4219.107662	-4218.334299
^{5}**S-3'**	-4219.110468	-4218.343101
^{1}**S-3''**	-4880.051488	-4879.271198
^{3}**S-3''**	-4880.016666	-4879.240776
^{5}**S-3''**	-4880.023588	-4879.254285
^{1}**S-3'''**	-6460.007264	-6459.228085
^{3}**S-3'''**	-6459.981183	-6459.204797
^{5}**S-3'''**	-6459.963853	-6459.194677
^{1}**S-4**	-4219.047759	-4218.265579
^{3}**S-4**	-4219.032593	-4218.257115
^{5}**S-4**	-4219.015479	-4218.245779
^{1}**TS(4-5)**	-4219.014021	-4218.231238
^{3}**TS(4-5)**	-4219.011069	-4218.237355
^{5}**TS(4-5)**	-4218.973023	-4218.201155
^{2}**S-5**	-4219.052313	-4218.268017
^{4}**S-5**	-4219.089601	-4218.314127
^{6}**S-5**	-4219.040583	-4218.266551
^{2}**S-6**	-3872.901959	-3872.219720
^{4}**S-6**	-3872.916868	-3872.243779
^{6}**S-6**	-3872.863414	-3872.197529
^{1}**S-7**	-3872.777975	-3872.091662
^{3}**S-7**	-3872.797899	-3872.120479
^{5}**S-7**	-3872.804834	-3872.133290
**4-MeOC$_6$H$_5$**	-347.352535	-347.254275

**Zn(4-MeOC$_6$H$_4$)$_2$**	-2473.820718	-2473.631085
**Zn(4-MeOC$_6$H$_4$)Br**	-4702.416521	-4702.336843
**MgBrCl**	-3236.320019	-3236.346682
**ZnCl$_2$**	-2701.586383	-2701.611850
**MgCl$^+$**	-660.796068	-660.818351
**ZnCl$^+$**	-2240.782608	-2240.806278
**Substrate 1**	-918.718564	-918.416049
**Product 3**	-1264.880563	-1264.474156

[a] Indices correspond to the spin state of the respective complexes.

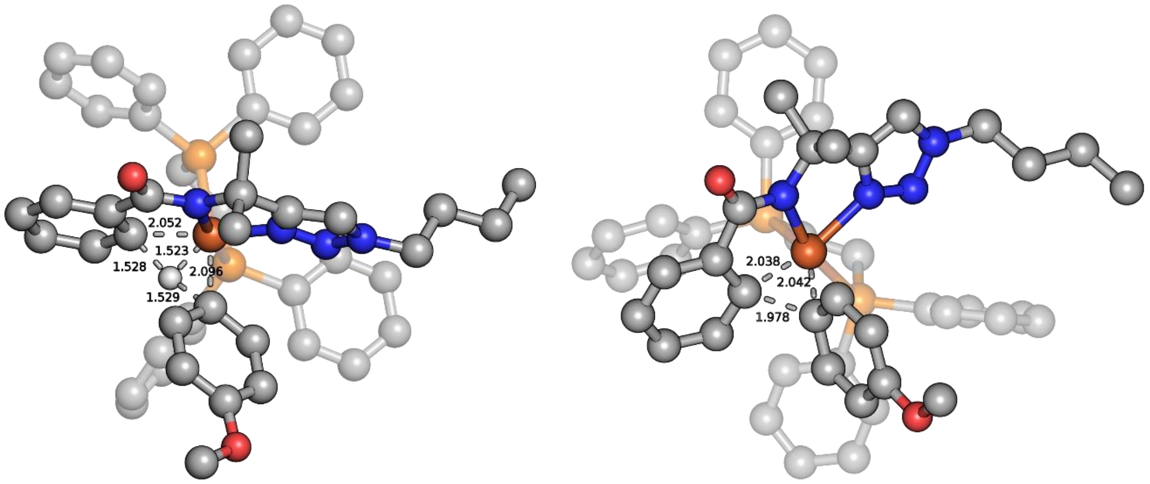

^{1}TS(1-2) **^{3}TS(4-5)**

Figure S11. Computed structure for the most stable transition state involved in C–H activation ^{1}TS(1-2) and reductive elimination ^{3}TS(4-5). Non-participating hydrogens were omitted for clarity. Key distances are reported in Å.

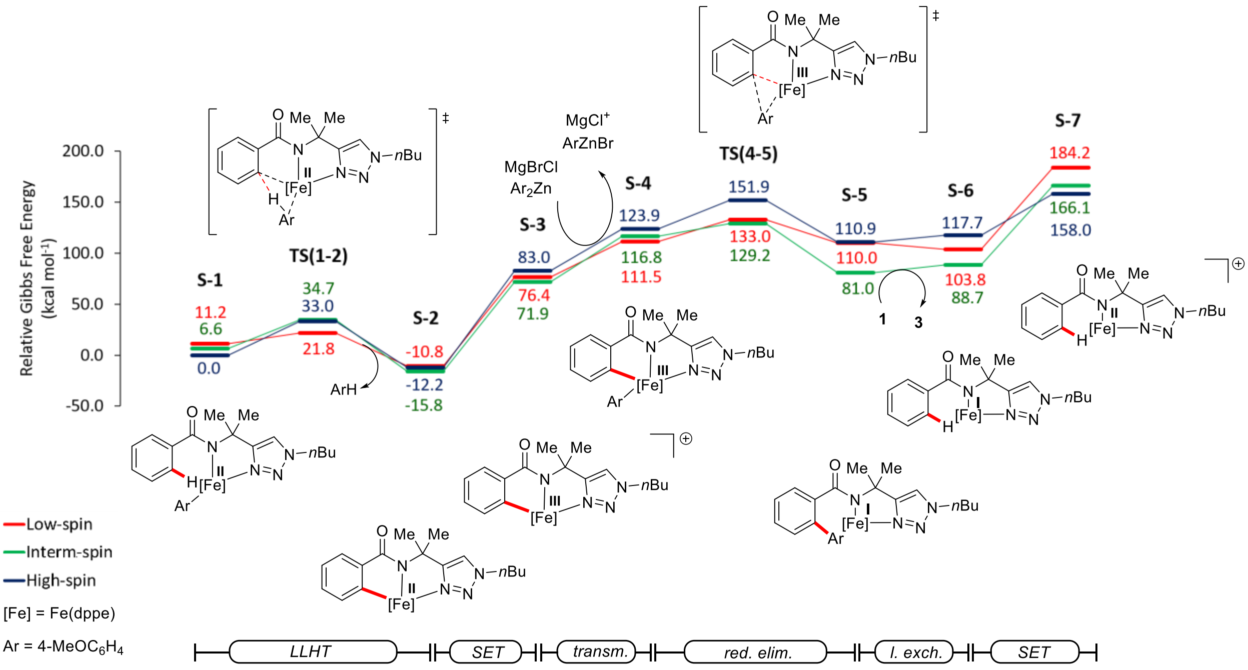

Figure S12. Computed reaction profile for iron-catalyzed C–H arylation, where oxidation by single-electron-transfer occurs after C–H activation followed by transmetalation. transm. = transmetalation. l. exch. = ligand exchange.

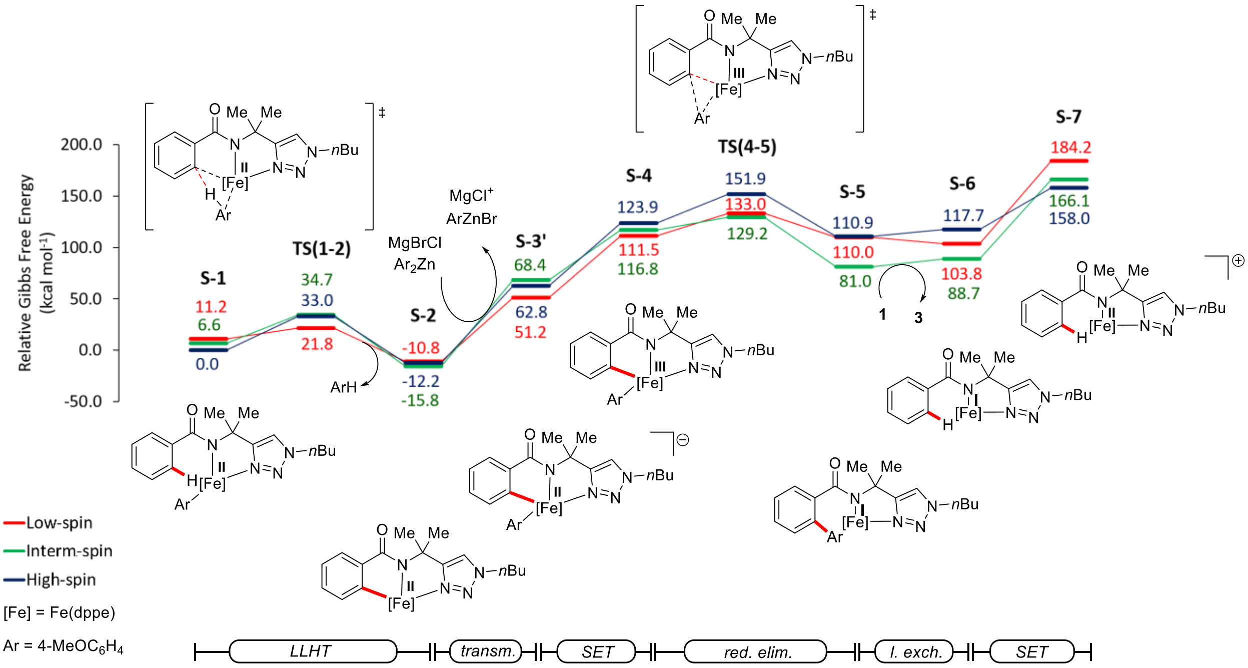

Figure S13. Computed reaction profile for iron-catalyzed C–H arylation, where oxidation by single-electron-transfer occurs after transmetalation. transm. = transmetalation. l. exch. = ligand exchange.

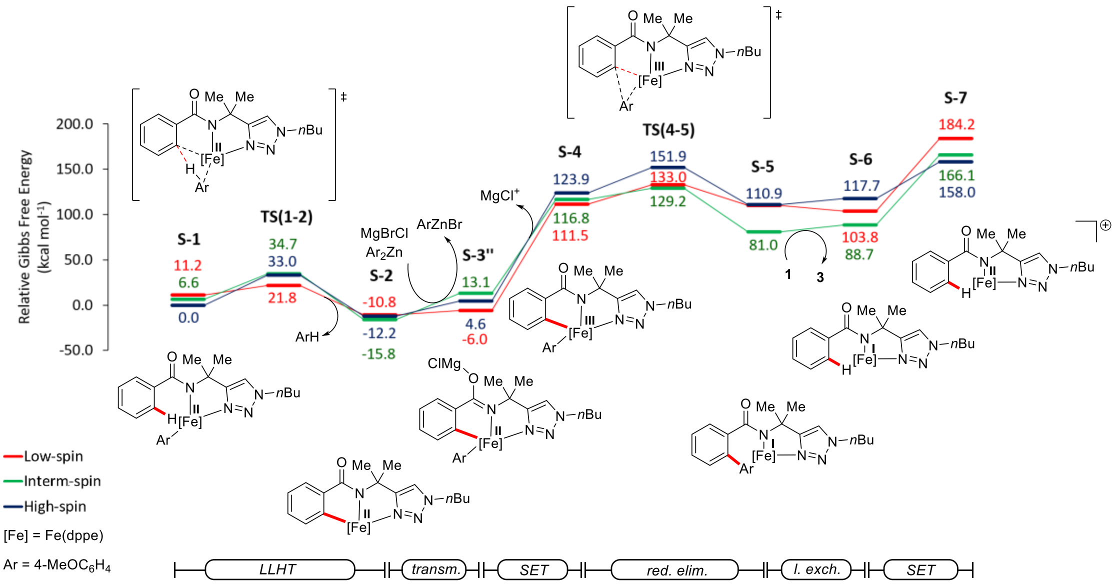

Figure S14. Computed reaction profile for iron-catalyzed C–H arylation, where oxidation by single-electron-transfer occurs after transmetalation with assistance of magnesium. transm. = transmetalation. l. exch. = ligand exchange.

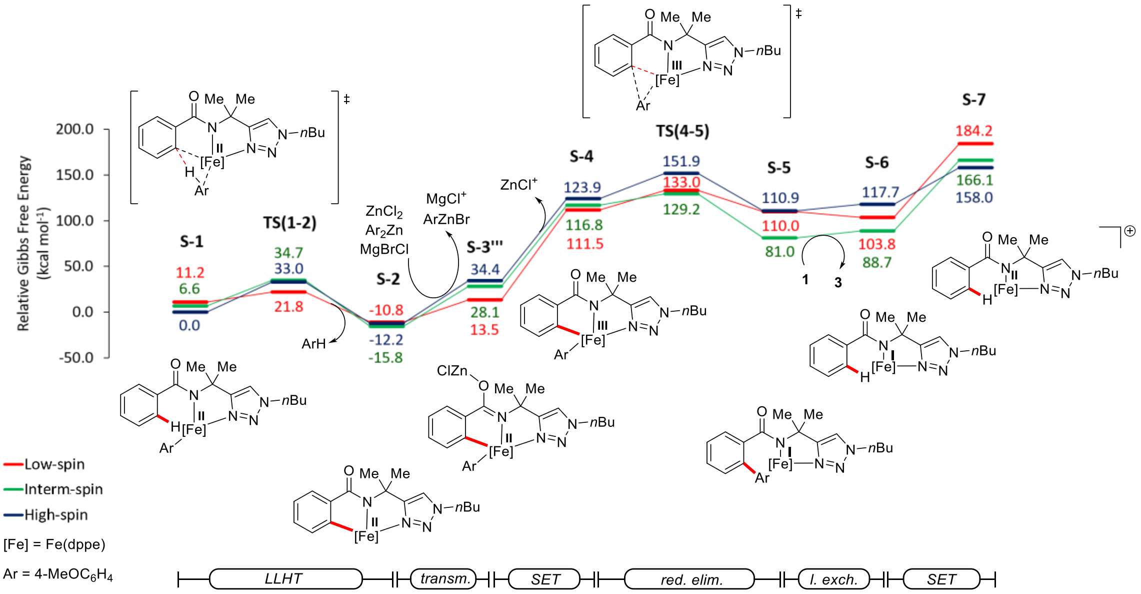

Figure S15. Computed reaction profile for iron-catalyzed C–H arylation, where oxidation through single electron transfer occurs after transmetalation with assistance of zinc. transm. = transmetalation. l. exch. = ligand exchange.

Table S5. Calculated oxidation potentials for the most stable Fe^{2+}/Fe^{3+} complexes.[a]

Redox pair	$E_{1/2}^{ox.}$ (V *vs.* Ferrocene)
^{3}S-2 → ^{3}S-3 + 1e^{-}	-1.28
^{3}S-3' → ^{3}S-4 + 1e^{-}	-2.47
**^{3}S-3'' → ^{3}S-4 + 1e^{-}**	**0.01**
^{3}S-3''' → ^{3}S-4 + 1e^{-}	-0.83

[a] Indices correspond to the spin state of the respective complexes.

Cartesian coordinates of the optimized structures

[1]S-1

Lowest frequency = 18.0886 cm^{-1}

Charge = 0, Multiplicity = 1

110

P	1.358944	-1.568910	-0.762291
P	-0.560307	0.611340	-1.744288
C	1.267181	-1.283113	-2.612537
H	2.008443	-0.500410	-2.854260
H	1.522403	-2.200573	-3.170261
C	-0.149007	-0.791203	-2.920830
H	-0.873833	-1.597040	-2.716277
H	-0.283972	-0.507434	-3.977662
C	2.467247	1.146959	-0.197735
C	3.236964	0.791946	0.935989
C	3.116168	1.534407	-1.387030
C	4.636926	0.833189	0.864377
C	4.514889	1.543107	-1.453749
H	2.527961	1.859042	-2.248631
C	5.277016	1.201058	-0.323675
H	5.190785	0.543770	1.763000
H	5.010425	1.838759	-2.384986
H	6.371032	1.214271	-0.376428
C	2.582830	0.270354	2.181330
N	1.295419	-0.077997	1.956343
C	0.488836	-0.613803	3.075835
C	-0.858851	-0.874626	2.460324
C	-2.091382	-1.296258	2.928240
N	-1.019415	-0.631426	1.118288
N	-2.255778	-0.872846	0.736504
N	-2.914028	-1.277924	1.834662
H	-2.442845	-1.579454	3.917902
O	3.226267	0.139697	3.248181
Fe	0.400654	0.241486	0.231299
C	3.122421	-1.979903	-0.457957
C	3.490119	-2.386932	0.842606
C	4.122612	-1.858879	-1.440461
C	4.825597	-2.659573	1.153674
H	2.726301	-2.473766	1.617262
C	5.460717	-2.139650	-1.127851
H	3.869361	-1.538455	-2.454280
C	5.816439	-2.535764	0.167978
H	5.091823	-2.951512	2.174416
H	6.228623	-2.037419	-1.902173
H	6.864098	-2.742550	0.411320
C	0.575614	-3.247832	-0.651180
C	1.332302	-4.437950	-0.615318
C	-0.832137	-3.343705	-0.657619
C	0.696694	-5.687013	-0.587383
H	2.424994	-4.387301	-0.610460
C	-1.464745	-4.594746	-0.643697
H	-1.435966	-2.432917	-0.650869
C	-0.703118	-5.771124	-0.605308
H	1.301510	-6.599925	-0.556628
H	-2.558532	-4.648388	-0.656096
H	-1.197100	-6.748573	-0.587154
C	-4.339884	-1.587149	1.724624
H	-4.700219	-1.801084	2.743653
H	-4.849437	-0.677044	1.362858
C	-4.601551	-2.760017	0.774873
C	-6.096908	-3.013218	0.547179
H	-4.112828	-3.667383	1.175830
H	-4.113289	-2.530105	-0.188279
H	-6.588887	-3.236821	1.513796
H	-6.565844	-2.084064	0.169220
C	-2.386478	0.797184	-2.009850
C	-2.982601	2.073923	-2.001209
C	-3.219486	-0.332134	-2.140822
C	-4.372785	2.215508	-2.110407
H	-2.358516	2.965871	-1.899788
C	-4.607578	-0.190753	-2.262401
H	-2.789397	-1.337552	-2.135164
C	-5.191391	1.084886	-2.240973
H	-4.815071	3.217285	-2.094876
H	-5.235479	-1.082126	-2.373642
H	-6.277206	1.196535	-2.332228
C	0.069510	2.114072	-2.632980
C	0.454531	2.102951	-3.988725
C	0.217142	3.310805	-1.898573
C	0.989046	3.251589	-4.589732
H	0.349977	1.195227	-4.589465

C	0.738851	4.460705	-2.505272
H	-0.057011	3.331274	-0.839344
C	1.134123	4.434132	-3.850125
H	1.292761	3.219931	-5.641777
H	0.848128	5.376026	-1.914265
H	1.555270	5.329025	-4.320534
C	-6.354351	-4.156830	-0.440535
H	-5.916786	-5.104111	-0.076601
H	-7.434865	-4.320748	-0.593498
H	-5.902761	-3.937517	-1.425351
C	1.068874	-1.945785	3.615303
H	2.104297	-1.768998	3.944489
H	0.467850	-2.310093	4.467883
H	1.048834	-2.718826	2.827882
C	0.354874	0.422797	4.218774
H	-0.274837	0.019408	5.033160
H	1.361048	0.641614	4.607647
H	-0.099798	1.348495	3.833041
C	-0.396906	1.925868	1.025366
C	-1.785619	2.199157	1.035995
C	0.416113	2.896150	1.652996
C	-2.326898	3.360016	1.600929
H	-2.482836	1.484699	0.585045
C	-0.099142	4.074483	2.224949
H	1.500656	2.749137	1.716883
C	-1.483972	4.315708	2.197260
H	-3.406123	3.548581	1.587518
H	0.589802	4.783909	2.692936
O	-2.095298	5.429468	2.720143
C	-1.272990	6.401012	3.341826
H	-0.536911	6.830503	2.632868
H	-1.947534	7.198103	3.690490
H	-0.725043	5.981017	4.208804
H	1.403709	1.558617	-0.088143

^{3}S-1

Lowest frequency = 7.3496 cm^{-1}

Charge = 0, Multiplicity = 3

110

P	1.588339	-1.443139	-0.855469
P	-0.413602	0.652722	-1.829189
C	1.652294	-0.987159	-2.663372
H	2.332293	-0.120599	-2.753700
H	2.049285	-1.820085	-3.269452
C	0.225463	-0.598804	-3.066212
H	-0.442917	-1.474582	-2.994670
H	0.155235	-0.226100	-4.102210
C	2.720398	1.488053	0.176805
C	3.229405	0.820209	1.307592
C	3.579803	2.039070	-0.786190
C	4.625533	0.770817	1.483225
C	4.967853	1.941283	-0.619489
H	3.156674	2.564221	-1.648501
C	5.487705	1.317066	0.527001
H	5.002023	0.278001	2.383869
H	5.643116	2.367247	-1.370198
H	6.571815	1.248435	0.669036
C	2.379584	0.146583	2.362991
N	1.105958	-0.187041	2.003661
C	0.241276	-0.771174	3.065453
C	-1.106331	-1.025051	2.431392
C	-2.363033	-1.300747	2.947616
N	-1.237739	-1.009249	1.069122
N	-2.481068	-1.261179	0.713909
N	-3.172499	-1.438848	1.851917
H	-2.740870	-1.390903	3.963551
O	2.899281	-0.088466	3.476946
Fe	0.279617	0.111210	0.249714
C	3.322873	-1.806888	-0.389805
C	3.550261	-2.401217	0.870287
C	4.422946	-1.496308	-1.209477
C	4.851305	-2.684192	1.296123
H	2.702953	-2.630792	1.521514
C	5.726261	-1.785664	-0.780339
H	4.273892	-1.020385	-2.182379
C	5.943930	-2.379256	0.469778
H	5.011317	-3.130452	2.282823
H	6.574999	-1.535364	-1.425595
H	6.963912	-2.595735	0.804721
C	0.883212	-3.155114	-0.970383
C	1.691879	-4.300054	-1.122730
C	-0.517252	-3.304540	-0.966635
C	1.105859	-5.565318	-1.265741
H	2.781928	-4.201437	-1.124997
C	-1.100955	-4.570003	-1.116103

H	-1.146016	-2.423734	-0.821503
C	-0.290673	-5.704732	-1.262818
H	1.744894	-6.447895	-1.379196
H	-2.192433	-4.666247	-1.105463
H	-0.743661	-6.696232	-1.370883
C	-4.622455	-1.639764	1.786553
H	-4.953837	-1.914079	2.801236
H	-5.089831	-0.672942	1.525330
C	-5.001452	-2.712814	0.761931
C	-6.520165	-2.872988	0.615999
H	-4.539303	-3.673808	1.056982
H	-4.557701	-2.430333	-0.208425
H	-6.961146	-3.152973	1.592643
H	-6.963118	-1.894327	0.346996
C	-2.185089	0.806562	-2.329148
C	-2.782501	2.043864	-2.636871
C	-2.988441	-0.352502	-2.310518
C	-4.154032	2.119101	-2.918423
H	-2.175428	2.953448	-2.651526
C	-4.354660	-0.277105	-2.606519
H	-2.549086	-1.317196	-2.042537
C	-4.944182	0.961076	-2.905622
H	-4.605120	3.089836	-3.150594
H	-4.962955	-1.188588	-2.597963
H	-6.014805	1.021954	-3.128823
C	0.337467	2.232928	-2.453429
C	1.104921	2.290129	-3.635733
C	0.200008	3.412902	-1.689152
C	1.730763	3.481136	-4.031610
H	1.230073	1.403763	-4.262978
C	0.818645	4.603444	-2.090951
H	-0.380688	3.393193	-0.764419
C	1.593651	4.642521	-3.259078
H	2.329013	3.496837	-4.949105
H	0.699091	5.501862	-1.476408
H	2.087485	5.570707	-3.565531
C	-6.902470	-3.915820	-0.440440
H	-6.496152	-4.910845	-0.183621
H	-7.998019	-4.011510	-0.532588
H	-6.502378	-3.638137	-1.432699
C	0.794839	-2.140947	3.543011
H	1.808623	-1.995572	3.944147
H	0.141447	-2.566612	4.325450
H	0.820687	-2.847164	2.694898
C	0.062018	0.204433	4.252658
H	-0.612334	-0.233860	5.011191
H	1.043438	0.395227	4.710182
H	-0.370645	1.151653	3.892285
C	-0.659572	1.768093	0.928025
C	-2.057242	1.939751	0.770064
C	-0.001012	2.766065	1.680198
C	-2.749385	3.035021	1.297553
H	-2.633833	1.191368	0.214662
C	-0.668406	3.880352	2.218262
H	1.076158	2.691060	1.866448
C	-2.055166	4.023157	2.021699
H	-3.829230	3.151965	1.154969
H	-0.100375	4.618884	2.791641
O	-2.804835	5.068381	2.496169
C	-2.139843	6.081729	3.232241
H	-1.360278	6.583247	2.625126
H	-2.911640	6.815482	3.510363
H	-1.670054	5.678802	4.151103
H	1.639278	1.657882	0.058924

^{5}S-1

Lowest frequency = 4.9427 cm^{-1}

Charge = 0, Multiplicity = 5

110

P	1.810040	-1.270271	-0.896774
P	-0.384749	0.817116	-1.937996
C	1.874274	-0.645842	-2.655382
H	2.474188	0.282768	-2.633508
H	2.378988	-1.376522	-3.311783
C	0.446184	-0.349610	-3.134293
H	-0.159568	-1.273260	-3.136519
H	0.426704	0.046937	-4.164509
C	2.491417	1.769374	0.485919
C	3.100245	1.009140	1.505177
C	3.266078	2.434993	-0.478078
C	4.506305	0.990355	1.575779
C	4.664341	2.360992	-0.423869
H	2.772254	3.031707	-1.250478
C	5.282568	1.649775	0.617993
H	4.959977	0.429069	2.397117

H	5.270102	2.876036	-1.177803	C	-2.121977	0.826958	-2.531912
H	6.375304	1.602167	0.676364	C	-2.776203	1.996708	-2.966651
C	2.350862	0.191589	2.539085	C	-2.855747	-0.377487	-2.451408
N	1.115786	-0.237059	2.162477	C	-4.133191	1.959660	-3.315768
C	0.347786	-1.037738	3.146115	H	-2.223817	2.939358	-3.024649
C	-0.965872	-1.393245	2.480587	C	-4.206134	-0.412311	-2.814675
C	-2.141307	-1.967643	2.942640	H	-2.374696	-1.286124	-2.077199
N	-1.161033	-1.140475	1.150868	C	-4.852969	0.758156	-3.243731
N	-2.363751	-1.511501	0.763591	H	-4.629884	2.877870	-3.647463
N	-2.966230	-2.015504	1.850313	H	-4.760023	-1.355839	-2.750884
H	-2.456159	-2.318513	3.922938	H	-5.913101	0.733525	-3.517343
O	2.930507	-0.069919	3.615986	C	0.270507	2.467091	-2.418706
Fe	0.151625	0.274580	0.435583	C	1.107791	2.671304	-3.535383
C	3.562355	-1.551269	-0.460595	C	-0.018888	3.566082	-1.577639
C	3.831057	-2.175154	0.778178	C	1.651184	3.936524	-3.798288
C	4.641906	-1.134380	-1.262776	H	1.348199	1.844079	-4.209503
C	5.147735	-2.392117	1.193506	C	0.517510	4.830641	-1.850848
H	3.001089	-2.476961	1.422813	H	-0.649353	3.417442	-0.695964
C	5.961456	-1.350099	-0.839813	C	1.359684	5.020621	-2.956826
H	4.464256	-0.634013	-2.218395	H	2.305002	4.074176	-4.666328
C	6.219134	-1.980445	0.384511	H	0.284345	5.667130	-1.183789
H	5.336420	-2.867330	2.161510	H	1.789238	6.006954	-3.161439
H	6.791547	-1.017562	-1.472355	C	-6.266975	-4.721108	-0.812467
H	7.251079	-2.142566	0.713288	H	-5.657519	-5.641028	-0.753347
C	1.186724	-2.988304	-1.154918	H	-7.325086	-5.021775	-0.900422
C	2.037349	-4.091767	-1.373309	H	-5.986495	-4.193991	-1.742577
C	-0.208545	-3.190598	-1.154022	C	1.071320	-2.364317	3.499328
C	1.497433	-5.366855	-1.589834	H	2.061233	-2.127773	3.916684
H	3.123039	-3.950781	-1.368604	H	0.485051	-2.939827	4.238292
C	-0.745698	-4.464617	-1.381570	H	1.182598	-2.983033	2.591988
H	-0.869573	-2.344884	-0.948421	C	0.050975	-0.209014	4.420299
C	0.106523	-5.557309	-1.597571	H	-0.570022	-0.787951	5.128505
H	2.167710	-6.217468	-1.755292	H	1.004791	0.050987	4.902557
H	-1.832680	-4.602126	-1.376246	H	-0.485804	0.712911	4.141857
H	-0.309617	-6.556393	-1.766279	C	-1.049760	1.850693	1.061409
C	-4.359579	-2.462382	1.757011	C	-2.425904	1.908350	0.722250
H	-4.596801	-2.966995	2.707547	C	-0.604407	2.827435	1.979346
H	-5.000652	-1.566112	1.677151	C	-3.293262	2.875771	1.241371
C	-4.577593	-3.390709	0.558468	H	-2.846178	1.169234	0.028135
C	-6.040741	-3.828645	0.413175	C	-1.449561	3.816592	2.515945
H	-3.920939	-4.274863	0.664292	H	0.444435	2.831829	2.306121
H	-4.250553	-2.858128	-0.351786	C	-2.805869	3.844531	2.140801
H	-6.361299	-4.363917	1.328233	H	-4.353157	2.906715	0.965180
H	-6.683707	-2.930029	0.340668	H	-1.044695	4.547641	3.222520

O	-3.719710	4.761174	2.596421
C	-3.269726	5.752198	3.504389
H	-2.477832	6.386742	3.059042
H	-4.146303	6.375943	3.737309
H	-2.880629	5.304523	4.440355
H	1.402439	1.944594	0.481100

[1]TS(1-2)

Lowest frequency = -762.5951 cm^{-1}

Charge = 0, Multiplicity = 1

110

P	-1.230618	-1.692691	0.859998
P	0.563018	0.598027	1.805553
C	-1.151022	-1.418713	2.705854
H	-1.968905	-0.720846	2.950230
H	-1.314811	-2.363936	3.251475
C	0.198914	-0.786443	3.033579
H	0.999672	-1.536211	2.921925
H	0.240771	-0.414204	4.070293
C	-2.427445	0.797940	0.419724
C	-3.344798	0.521857	-0.633702
C	-2.967499	1.275745	1.633651
C	-4.723890	0.710577	-0.477576
C	-4.352166	1.455115	1.795573
H	-2.307129	1.525289	2.470229
C	-5.234789	1.178275	0.739974
H	-5.366953	0.461051	-1.328557
H	-4.738378	1.822387	2.753756
H	-6.313153	1.320874	0.871452
C	-2.802880	-0.043572	-1.910595
N	-1.472790	-0.267979	-1.792801
C	-0.696051	-0.719444	-2.965374
C	0.725366	-0.733994	-2.475224
C	1.961405	-0.956066	-3.059959
N	0.967511	-0.419427	-1.162270
N	2.258854	-0.429188	-0.906245
N	2.868559	-0.750636	-2.056683
H	2.259562	-1.213949	-4.073743
O	-3.520197	-0.273021	-2.910541
Fe	-0.536230	0.207036	-0.113028
C	-2.963652	-2.236447	0.588293
C	-3.312626	-2.769436	-0.668786
C	-3.975289	-2.041252	1.548422
C	-4.644267	-3.069856	-0.972157
H	-2.540879	-2.918490	-1.424553
C	-5.306958	-2.354397	1.246919
H	-3.740757	-1.618472	2.527731
C	-5.647100	-2.858942	-0.015520
H	-4.897853	-3.448852	-1.966927
H	-6.083846	-2.183080	1.999440
H	-6.692329	-3.081521	-0.255193
C	-0.288475	-3.277606	0.688921
C	-0.900929	-4.542388	0.807251
C	1.104113	-3.212185	0.480922
C	-0.134299	-5.712532	0.719712
H	-1.982141	-4.608777	0.964353
C	1.870187	-4.384115	0.403566
H	1.583979	-2.236980	0.367495
C	1.252577	-5.637562	0.519537
H	-0.623498	-6.688607	0.809647
H	2.951655	-4.316278	0.243070
H	1.848336	-6.554166	0.450765
C	4.323862	-0.898839	-2.054423
H	4.653163	-0.905661	-3.106044
H	4.737392	-0.000998	-1.565656
C	4.763316	-2.165006	-1.310313
C	6.286288	-2.268597	-1.164907
H	4.363924	-3.053513	-1.834725
H	4.296303	-2.142352	-0.310138
H	6.757989	-2.271983	-2.166899
H	6.659406	-1.362318	-0.649766
C	2.423768	0.671728	1.924065
C	3.075129	1.919234	1.832061
C	3.217107	-0.487600	2.028282
C	4.474400	2.000940	1.817791
H	2.483054	2.837594	1.773055
C	4.615409	-0.406075	2.035604
H	2.750303	-1.474382	2.092734
C	5.252012	0.838271	1.917880
H	4.956837	2.981284	1.740097
H	5.210432	-1.321171	2.132158
H	6.345565	0.902155	1.919264
C	0.202737	2.141489	2.780194
C	0.581820	2.238387	4.135651
C	-0.391626	3.257813	2.162511

C	0.346137	3.411692	4.862434
H	1.084399	1.397748	4.626257
C	-0.619574	4.436345	2.888334
H	-0.673518	3.212367	1.107666
C	-0.258441	4.514933	4.239612
H	0.642033	3.467633	5.915661
H	-1.084388	5.293176	2.389638
H	-0.441462	5.433851	4.806890
C	6.717873	-3.516640	-0.386605
H	6.379500	-4.439029	-0.892448
H	7.815673	-3.569222	-0.287083
H	6.285698	-3.516723	0.630777
C	-1.104949	-2.143576	-3.416983
H	-2.180404	-2.137582	-3.653303
H	-0.534047	-2.445033	-4.313918
H	-0.896151	-2.873965	-2.616577
C	-0.823358	0.270837	-4.151128
H	-0.222635	-0.079477	-5.010738
H	-1.883271	0.328995	-4.441969
H	-0.470851	1.269148	-3.848736
C	-0.151259	2.072025	-0.988474
C	1.180903	2.546216	-0.955390
C	-1.063256	2.808661	-1.769896
C	1.585670	3.668520	-1.682002
H	1.939758	2.002635	-0.385172
C	-0.682381	3.950071	-2.497425
H	-2.111090	2.493738	-1.820740
C	0.653785	4.384899	-2.459745
H	2.625870	4.010933	-1.663831
H	-1.435010	4.480260	-3.087377
O	1.137985	5.475960	-3.132230
C	0.230743	6.217111	-3.932185
H	-0.591910	6.645598	-3.326380
H	0.815086	7.034417	-4.381358
H	-0.206813	5.593996	-4.736908
H	-1.172701	1.582391	0.038606

^{3}TS(1-2)

Lowest frequency = -1161.9018 cm^{-1}

Charge = 0, Multiplicity = 3

110

P	-1.404261	-1.520519	0.965900
P	0.896148	0.556657	1.855085
C	-1.159843	-1.149417	2.773166
H	-1.805985	-0.285218	3.006295
H	-1.480566	-2.006179	3.392017
C	0.312745	-0.804583	3.013120
H	0.938007	-1.692299	2.816785
H	0.487739	-0.508318	4.061216
C	-2.459461	1.082227	0.269111
C	-3.434979	0.798680	-0.732155
C	-2.915501	1.643398	1.482988
C	-4.794101	1.060166	-0.515887
C	-4.279581	1.898352	1.699078
H	-2.194230	1.903084	2.266007
C	-5.222322	1.611881	0.699194
H	-5.494218	0.798573	-1.316806
H	-4.603582	2.331174	2.652841
H	-6.285709	1.810913	0.871777
C	-2.989400	0.131446	-2.002219
N	-1.665112	-0.186681	-1.922248
C	-0.955356	-0.759487	-3.078124
C	0.484925	-0.843181	-2.618340
C	1.686044	-1.194446	-3.216215
N	0.795118	-0.455683	-1.338583
N	2.088732	-0.540646	-1.112885
N	2.636481	-0.984372	-2.254167
H	1.937064	-1.545887	-4.214748
O	-3.763585	-0.097569	-2.952891
Fe	-0.654992	0.330122	-0.301667
C	-3.182104	-1.918905	0.809223
C	-3.629028	-2.488734	-0.401803
C	-4.128864	-1.572252	1.791996
C	-4.992914	-2.691135	-0.631622
H	-2.905288	-2.746776	-1.177925
C	-5.494839	-1.782541	1.560373
H	-3.811727	-1.116667	2.733290
C	-5.931177	-2.335089	0.348720
H	-5.322816	-3.107585	-1.588431
H	-6.221447	-1.496457	2.328093
H	-7.000863	-2.481212	0.165134
C	-0.596155	-3.173625	0.817721
C	-1.278304	-4.378306	1.087365
C	0.765539	-3.224604	0.454623
C	-0.608284	-5.605861	0.999228

H	-2.337923	-4.351501	1.361669
C	1.435638	-4.454084	0.378897
H	1.295582	-2.296063	0.224998
C	0.750030	-5.647408	0.647723
H	-1.149379	-6.535170	1.208207
H	2.494204	-4.478881	0.099033
H	1.270428	-6.608794	0.579680
C	4.075369	-1.253094	-2.277981
H	4.387101	-1.279111	-3.334798
H	4.564811	-0.394941	-1.789335
C	4.421657	-2.556089	-1.548532
C	5.933561	-2.789857	-1.442578
H	3.935584	-3.404605	-2.066401
H	3.985010	-2.497668	-0.536147
H	6.380084	-2.811921	-2.455893
H	6.391931	-1.929163	-0.918815
C	2.726420	0.363725	1.971169
C	3.552055	1.510750	1.886133
C	3.351448	-0.904925	1.958861
C	4.942361	1.391798	1.784628
H	3.096382	2.506700	1.904619
C	4.744240	-1.019947	1.870745
H	2.751353	-1.817851	2.017843
C	5.550499	0.124861	1.773105
H	5.557291	2.296689	1.725057
H	5.204294	-2.014632	1.884493
H	6.639499	0.032147	1.703388
C	0.662635	2.070277	2.898138
C	1.113276	2.113792	4.234902
C	0.064592	3.217749	2.344036
C	0.949611	3.271743	5.004678
H	1.613529	1.241804	4.671035
C	-0.097343	4.378949	3.115702
H	-0.262941	3.207327	1.300862
C	0.339226	4.406850	4.446551
H	1.304561	3.291719	6.040899
H	-0.564662	5.262639	2.668715
H	0.211969	5.312855	5.048944
C	6.276173	-4.086336	-0.700088
H	5.853079	-4.966409	-1.217550
H	7.367734	-4.230617	-0.625718
H	5.866290	-4.074244	0.326231
C	-1.478386	-2.171978	-3.434871
H	-2.554687	-2.099609	-3.656412
H	-0.949061	-2.575786	-4.316922
H	-1.316144	-2.862313	-2.589436
C	-1.045299	0.180498	-4.307097
H	-0.464761	-0.228420	-5.154267
H	-2.102553	0.276598	-4.598012
H	-0.646980	1.174556	-4.045878
C	-0.035647	2.203533	-0.961527
C	1.326701	2.553931	-0.805902
C	-0.800055	2.993006	-1.844105
C	1.894681	3.625584	-1.498136
H	1.975910	1.953824	-0.160047
C	-0.249017	4.074058	-2.552151
H	-1.862083	2.763432	-1.991294
C	1.109992	4.396492	-2.379427
H	2.953617	3.879239	-1.381739
H	-0.887045	4.651927	-3.226503
O	1.752204	5.423756	-3.017950
C	1.000453	6.219269	-3.920593
H	0.161702	6.733110	-3.411101
H	1.697926	6.970818	-4.320341
H	0.594219	5.615418	-4.755869
H	-1.207709	1.763927	-0.029198

^{5}TS(1-2)

Lowest frequency = -1254.0099 cm^{-1}

Charge = 0, Multiplicity = 5

110

P	-2.341877	0.662340	1.040877
P	0.308075	2.106661	-0.177114
C	-2.326684	2.488549	0.689454
H	-2.604392	2.617146	-0.372410
H	-3.065469	3.017622	1.317221
C	-0.900108	2.993977	0.937801
H	-0.585074	2.768760	1.972556
H	-0.810475	4.086347	0.810347
C	-1.746156	-0.261623	-1.987861
C	-2.555703	-1.416494	-1.783287
C	-2.260447	0.741825	-2.837965
C	-3.794969	-1.561536	-2.432183
C	-3.509599	0.610264	-3.464937
H	-1.666961	1.639497	-3.040300

C	-4.274646	-0.551887	-3.272591
H	-4.367359	-2.473999	-2.235496
H	-3.880255	1.411341	-4.116443
H	-5.246893	-0.660943	-3.766337
C	-2.146828	-2.504053	-0.817601
N	-1.038193	-2.171665	-0.095046
C	-0.413370	-3.160601	0.804467
C	0.911423	-2.520666	1.158310
C	2.200430	-3.004888	1.274829
N	0.988298	-1.154691	1.298695
N	2.237076	-0.781025	1.507011
N	2.979503	-1.897976	1.486854
H	2.622364	-4.003384	1.192174
O	-2.808211	-3.558002	-0.713372
Fe	-0.368448	-0.292445	-0.171827
C	-4.027383	0.104473	0.628836
C	-4.409611	-1.186834	1.052740
C	-4.910375	0.856954	-0.168885
C	-5.650626	-1.712821	0.684273
H	-3.720382	-1.787432	1.652872
C	-6.156550	0.326955	-0.529196
H	-4.626599	1.849145	-0.529382
C	-6.528682	-0.956489	-0.107512
H	-5.921854	-2.725075	0.999595
H	-6.832676	0.917719	-1.156180
H	-7.497459	-1.372292	-0.403979
C	-2.315127	0.635742	2.881808
C	-3.457712	0.902627	3.663428
C	-1.087042	0.365374	3.517938
C	-3.365841	0.910020	5.061084
H	-4.418461	1.094671	3.173153
C	-0.998439	0.380513	4.917759
H	-0.215127	0.116079	2.902409
C	-2.136274	0.653396	5.690044
H	-4.257132	1.115310	5.664033
H	-0.040673	0.165913	5.404150
H	-2.069979	0.658225	6.783385
C	4.448045	-1.840684	1.508542
H	4.800119	-2.495979	2.325507
H	4.799424	-2.269038	0.552504
C	4.978660	-0.419513	1.680623
C	6.511772	-0.384919	1.607258
H	4.629059	-0.007899	2.643798
H	4.543947	0.227044	0.899942
H	6.936796	-1.040501	2.392759
H	6.838529	-0.813883	0.639853
C	1.905024	2.741445	0.493651
C	2.811573	3.500299	-0.272895
C	2.244206	2.407530	1.822227
C	4.024839	3.929291	0.284892
H	2.564235	3.762918	-1.306188
C	3.445835	2.855761	2.381067
H	1.570357	1.784443	2.418002
C	4.341854	3.617486	1.614597
H	4.719195	4.520843	-0.321709
H	3.689786	2.598756	3.417509
H	5.285159	3.961379	2.051287
C	0.145393	3.010904	-1.776696
C	-0.635254	4.174556	-1.929940
C	0.776863	2.468050	-2.917830
C	-0.784953	4.775506	-3.187827
H	-1.141125	4.622938	-1.070275
C	0.642756	3.082021	-4.169218
H	1.350708	1.541593	-2.832911
C	-0.144292	4.234156	-4.311156
H	-1.404429	5.673393	-3.287256
H	1.139424	2.642030	-5.040207
H	-0.263693	4.704176	-5.293006
C	7.078949	1.030945	1.755598
H	6.789179	1.475329	2.724910
H	8.181331	1.028499	1.699174
H	6.695829	1.694341	0.960457
C	-1.280080	-3.354155	2.073871
H	-2.254630	-3.764093	1.763590
H	-0.799786	-4.050015	2.784842
H	-1.432006	-2.384288	2.576536
C	-0.149954	-4.521415	0.121078
H	0.351585	-5.210791	0.825198
H	-1.108122	-4.955795	-0.196650
H	0.495011	-4.382161	-0.761945
C	1.101818	-0.834401	-1.867101
C	2.378343	-0.302608	-1.568981
C	1.075788	-2.119086	-2.445650
C	3.554898	-1.029118	-1.787239
H	2.483124	0.687597	-1.108569
C	2.238332	-2.870966	-2.674486
H	0.106995	-2.560334	-2.710963
C	3.489262	-2.330725	-2.316565

H	4.537196	-0.609125	-1.545312
H	2.158882	-3.870072	-3.112735
O	4.686324	-2.993505	-2.430481
C	4.670552	-4.306397	-2.970908
H	4.280164	-4.315554	-4.006916
H	5.716290	-4.649056	-2.972943
H	4.059739	-4.994453	-2.353626
H	-0.277886	-0.349197	-1.972710

^{1}S-2

Lowest frequency = 15.2139 cm^{-1}

Charge = 0, Multiplicity = 1

94

P	0.969806	-0.848219	-1.209317
P	-0.235280	1.761574	-0.777148
C	1.227269	0.276972	-2.692775
H	2.276425	0.605759	-2.650511
H	1.078149	-0.291424	-3.625746
C	0.276078	1.479663	-2.586177
H	-0.640891	1.313804	-3.173655
H	0.744778	2.402046	-2.968416
C	2.526837	0.983607	0.196664
C	3.474349	0.360576	1.055738
C	3.062361	1.952323	-0.684437
C	4.846693	0.633385	1.020619
C	4.439323	2.242516	-0.733945
H	2.409585	2.516923	-1.361061
C	5.343026	1.575817	0.107972
H	5.499557	0.093436	1.715954
H	4.804231	2.998943	-1.439587
H	6.414624	1.799515	0.060623
C	2.917803	-0.610588	2.041099
N	1.540505	-0.603433	1.977333
C	0.777258	-1.473903	2.894181
C	-0.653220	-1.135751	2.580711
C	-1.885811	-1.427563	3.140342
N	-0.893557	-0.334149	1.487762
N	-2.190997	-0.110470	1.353735
N	-2.791978	-0.772150	2.350751
H	-2.182676	-2.014788	4.006636
O	3.608229	-1.289210	2.818298
Fe	0.687172	0.305642	0.538772
C	2.435644	-1.964594	-1.218821
C	2.287232	-3.317571	-0.851451
C	3.728971	-1.464471	-1.468760
C	3.404894	-4.153697	-0.742019
H	1.288702	-3.721100	-0.654953
C	4.844728	-2.305309	-1.366011
H	3.876738	-0.409832	-1.718642
C	4.687938	-3.648988	-0.999822
H	3.272698	-5.201069	-0.450111
H	5.842600	-1.896893	-1.555821
H	5.563607	-4.300321	-0.908042
C	-0.345285	-1.984918	-1.865390
C	-0.087280	-2.794349	-2.993469
C	-1.627098	-2.008539	-1.290090
C	-1.091975	-3.609152	-3.528965
H	0.910933	-2.795490	-3.445697
C	-2.634141	-2.825975	-1.827805
H	-1.842625	-1.362585	-0.438155
C	-2.369703	-3.627980	-2.945383
H	-0.877917	-4.233968	-4.402967
H	-3.630005	-2.828478	-1.371903
H	-3.154738	-4.266743	-3.364522
C	-4.255431	-0.742513	2.419389
H	-4.546253	-1.242757	3.357186
H	-4.561265	0.315899	2.478582
C	-4.880232	-1.411078	1.190214
C	-6.406888	-1.277340	1.149256
H	-4.586146	-2.477577	1.169918
H	-4.443213	-0.933387	0.296193
H	-6.851503	-1.748440	2.047676
H	-6.674290	-0.204020	1.196694
C	-2.082316	1.863754	-0.902296
C	-2.813488	1.022223	-1.761913
C	-2.790216	2.687300	-0.003423
C	-4.214745	1.002314	-1.725717
H	-2.294852	0.357730	-2.459407
C	-4.189766	2.666999	0.035807
H	-2.237482	3.344962	0.675755
C	-4.909086	1.822137	-0.824187
H	-4.763362	0.342197	-2.406831
H	-4.722596	3.318225	0.737855
H	-6.004300	1.809553	-0.798272
C	0.145256	3.542751	-0.453478
C	-0.257960	4.566517	-1.333131

C	0.838521	3.875492	0.723940
C	0.039471	5.904200	-1.041900
H	-0.817230	4.317280	-2.242388
C	1.128393	5.215273	1.019094
H	1.166988	3.071841	1.392961
C	0.732797	6.229505	0.135375
H	-0.273298	6.696064	-1.731379
H	1.673201	5.465014	1.935701
H	0.964563	7.275900	0.361947
C	-7.010452	-1.899149	-0.115265
H	-6.778520	-2.977831	-0.178681
H	-8.108280	-1.788207	-0.134400
H	-6.603898	-1.415993	-1.022051
C	1.038880	-2.970927	2.597303
H	2.110025	-3.177591	2.743841
H	0.440546	-3.615324	3.266411
H	0.766950	-3.191496	1.552856
C	1.075419	-1.142514	4.376551
H	0.436248	-1.748991	5.043989
H	2.133630	-1.357298	4.584360
H	0.878271	-0.074141	4.567771

^{3}S-2

Lowest frequency = 11.5735 cm^{-1}

Charge = 0, Multiplicity = 3

94

P	1.033294	-0.850454	-1.330551
P	-0.340443	1.813748	-0.710490
C	1.141314	0.430823	-2.685020
H	2.116695	0.922285	-2.541107
H	1.129355	-0.046181	-3.680130
C	0.006883	1.453892	-2.532945
H	-0.920901	1.050076	-2.969443
H	0.237400	2.391882	-3.066341
C	2.494843	0.929148	0.392465
C	3.395372	0.158087	1.181809
C	3.089133	1.879851	-0.470533
C	4.788073	0.289205	1.092563
C	4.483389	2.030996	-0.560259
H	2.465576	2.541466	-1.084846
C	5.339747	1.227011	0.211303
H	5.408916	-0.354586	1.725668
H	4.903442	2.784115	-1.238357
H	6.426566	1.339881	0.126802
C	2.782248	-0.805242	2.140848
N	1.415395	-0.754490	2.032445
C	0.585350	-1.538165	2.964799
C	-0.827071	-1.230347	2.549305
C	-2.081733	-1.628713	2.981627
N	-1.029267	-0.390305	1.478463
N	-2.319204	-0.260030	1.226260
N	-2.957550	-1.000741	2.137791
H	-2.411907	-2.278133	3.789395
O	3.431894	-1.521662	2.926695
Fe	0.622425	0.291132	0.626029
C	2.640021	-1.721872	-1.366183
C	2.791567	-2.797989	-0.465628
C	3.768952	-1.253246	-2.064873
C	4.043657	-3.384311	-0.259523
H	1.926454	-3.149663	0.103950
C	5.022386	-1.845272	-1.858725
H	3.686267	-0.407933	-2.752958
C	5.165187	-2.905067	-0.953065
H	4.148242	-4.198112	0.464941
H	5.895057	-1.460403	-2.396872
H	6.150623	-3.349545	-0.778243
C	-0.164843	-2.074430	-2.009708
C	0.209406	-3.152964	-2.836719
C	-1.524945	-1.895567	-1.681895
C	-0.764849	-4.025821	-3.339816
H	1.266252	-3.306454	-3.080436
C	-2.498071	-2.761938	-2.200022
H	-1.810308	-1.082028	-1.005926
C	-2.119988	-3.828779	-3.028941
H	-0.466212	-4.862597	-3.980907
H	-3.552733	-2.610214	-1.946002
H	-2.878465	-4.511735	-3.426788
C	-4.421549	-1.060147	2.087071
H	-4.749761	-1.662659	2.949294
H	-4.798781	-0.031094	2.215048
C	-4.906252	-1.646254	0.757519
C	-6.431844	-1.608951	0.611544
H	-4.533800	-2.683351	0.661065
H	-4.441783	-1.056916	-0.051798
H	-6.902873	-2.188674	1.429332

H	-6.779138	-0.564440	0.731654
C	-2.182250	2.006800	-0.661999
C	-3.066627	1.355328	-1.541067
C	-2.723004	2.739164	0.416073
C	-4.453985	1.426663	-1.345059
H	-2.687359	0.769461	-2.383214
C	-4.105751	2.806197	0.615916
H	-2.048314	3.251530	1.110551
C	-4.979698	2.145023	-0.262774
H	-5.125420	0.918086	-2.045944
H	-4.504893	3.381111	1.458896
H	-6.063330	2.201446	-0.111889
C	0.127862	3.589268	-0.475869
C	-0.268790	4.593250	-1.381267
C	0.849075	3.945416	0.678270
C	0.066959	5.931981	-1.142240
H	-0.855582	4.330837	-2.269079
C	1.178129	5.286940	0.920241
H	1.165849	3.155535	1.369080
C	0.791348	6.280090	0.009446
H	-0.241816	6.707161	-1.852086
H	1.745547	5.553508	1.818183
H	1.052571	7.327523	0.195344
C	-6.899724	-2.151832	-0.743618
H	-6.584813	-3.201948	-0.882365
H	-7.998773	-2.113562	-0.835566
H	-6.469390	-1.561973	-1.573245
C	0.834600	-3.061667	2.818537
H	1.899654	-3.259225	3.012256
H	0.213486	-3.629803	3.534659
H	0.575039	-3.387949	1.797285
C	0.820191	-1.085266	4.427180
H	0.158538	-1.635161	5.121045
H	1.871452	-1.280995	4.687869
H	0.618507	-0.004886	4.519213

[5]S-2

Lowest frequency = 13.5369 cm^{-1}

Charge = 0, Multiplicity = 5

94

P	1.083312	-0.644024	-1.324827
P	-0.588725	1.933374	-0.584134
C	1.245175	0.817317	-2.472771
H	2.152385	1.345702	-2.133570
H	1.412870	0.495179	-3.515466
C	0.006974	1.721906	-2.362733
H	-0.831694	1.288838	-2.933127
H	0.209352	2.721483	-2.784736
C	2.807563	1.191940	0.630405
C	3.686799	0.265911	1.272854
C	3.418983	2.271129	-0.048090
C	5.082776	0.414457	1.229379
C	4.816117	2.423187	-0.099936
H	2.798014	3.024085	-0.554105
C	5.653070	1.490809	0.537209
H	5.695103	-0.339558	1.736457
H	5.254026	3.273260	-0.638008
H	6.741977	1.608542	0.494897
C	3.109810	-0.925207	1.996078
N	1.738436	-0.927149	1.964796
C	0.972101	-1.966997	2.650858
C	-0.472499	-1.530304	2.494374
C	-1.689753	-2.036111	2.928002
N	-0.756948	-0.381299	1.799132
N	-2.050521	-0.145442	1.782714
N	-2.623558	-1.143785	2.471990
H	-1.960009	-2.914247	3.510731
O	3.826500	-1.783601	2.543880
Fe	0.938956	0.454099	0.769538
C	2.545977	-1.679898	-1.688783
C	2.421639	-3.072920	-1.868186
C	3.831963	-1.098994	-1.660358
C	3.563358	-3.870146	-2.021093
H	1.428430	-3.533799	-1.888134
C	4.967814	-1.902206	-1.818076
H	3.950994	-0.024059	-1.494980
C	4.838892	-3.287386	-1.995668
H	3.454825	-4.951543	-2.157888
H	5.958968	-1.438488	-1.783875
H	5.730914	-3.912732	-2.107860
C	-0.321704	-1.591902	-2.052104
C	-0.561200	-1.669838	-3.439900
C	-1.235086	-2.198036	-1.166813
C	-1.701422	-2.322265	-3.928130
H	0.145385	-1.217563	-4.143800

C	-2.360063	-2.875484	-1.657051
H	-1.063128	-2.116938	-0.089889
C	-2.602182	-2.928070	-3.037265
H	-1.884874	-2.364979	-5.007208
H	-3.057645	-3.349206	-0.959638
H	-3.490700	-3.441235	-3.420495
C	-4.085939	-1.212018	2.523744
H	-4.364246	-1.748259	3.446003
H	-4.439564	-0.171646	2.600982
C	-4.660998	-1.883559	1.271828
C	-6.192833	-1.838599	1.223330
H	-4.310295	-2.932611	1.229715
H	-4.245717	-1.362898	0.391120
H	-6.611931	-2.343129	2.115884
H	-6.520953	-0.783104	1.279213
C	-2.411964	1.782997	-0.740604
C	-2.994556	0.796970	-1.566765
C	-3.254516	2.531829	0.111640
C	-4.379887	0.590453	-1.564007
H	-2.370162	0.173974	-2.213929
C	-4.637220	2.318966	0.115046
H	-2.820088	3.285891	0.776037
C	-5.210282	1.349142	-0.724929
H	-4.808076	-0.170549	-2.225546
H	-5.274165	2.919369	0.774256
H	-6.293873	1.189761	-0.728050
C	-0.344674	3.725952	-0.250856
C	-0.979414	4.729390	-1.013230
C	0.522378	4.094694	0.794262
C	-0.745239	6.080447	-0.730597
H	-1.666583	4.448646	-1.819546
C	0.757151	5.449236	1.073524
H	1.025380	3.310048	1.373140
C	0.124497	6.441621	0.312298
H	-1.241558	6.855906	-1.324224
H	1.438012	5.725843	1.885285
H	0.307543	7.499574	0.529234
C	-6.755779	-2.483296	-0.048306
H	-6.464530	-3.547150	-0.118766
H	-7.858025	-2.433265	-0.070708
H	-6.373448	-1.971742	-0.949675
C	1.191297	-3.344500	1.976308
H	2.256311	-3.608059	2.069446
H	0.571855	-4.127255	2.451263
H	0.934110	-3.283503	0.905482
C	1.314244	-2.039591	4.160631
H	0.684493	-2.793573	4.667369
H	2.375633	-2.308523	4.267768
H	1.140950	-1.056931	4.630795

[2]S-3

Lowest frequency = 11.8712 cm^{-1}

Charge = 1, Multiplicity = 2

94

P	1.337075	-0.603227	-1.222002
P	-0.525636	1.712629	-0.848504
C	1.416816	0.647187	-2.594138
H	2.307963	1.262375	-2.398268
H	1.561263	0.123417	-3.554020
C	0.143579	1.501544	-2.591584
H	-0.651952	1.013224	-3.176539
H	0.319531	2.492035	-3.042407
C	2.399485	1.159123	0.471096
C	3.347245	0.450700	1.256683
C	2.847656	2.323905	-0.187177
C	4.677651	0.866960	1.384860
C	4.178588	2.758259	-0.050262
H	2.167691	2.924348	-0.801262
C	5.097523	2.029082	0.723752
H	5.356048	0.273715	2.007013
H	4.499519	3.673513	-0.559944
H	6.133483	2.372037	0.811145
C	2.828893	-0.748803	1.967380
N	1.439081	-0.784270	1.846589
C	0.658010	-1.714742	2.696143
C	-0.767011	-1.309057	2.450559
C	-2.006767	-1.685936	2.942062
N	-1.001067	-0.316108	1.530851
N	-2.293421	-0.053407	1.436573
N	-2.898033	-0.878487	2.288390
H	-2.317852	-2.423291	3.678952
O	3.508099	-1.544768	2.618546
Fe	0.615165	0.348077	0.601340
C	2.958025	-1.439127	-1.213477
C	3.034242	-2.776099	-0.773886

C	4.138368	-0.751942	-1.560286
C	4.275069	-3.416765	-0.685392
H	2.122719	-3.315156	-0.500993
C	5.374713	-1.403130	-1.483074
H	4.104199	0.298214	-1.863773
C	5.446104	-2.732460	-1.041809
H	4.327212	-4.451875	-0.334214
H	6.286714	-0.862680	-1.754602
H	6.416257	-3.234149	-0.970249
C	0.200553	-1.898511	-1.871605
C	0.560796	-2.623312	-3.028640
C	-1.029921	-2.160947	-1.244998
C	-0.309234	-3.588554	-3.548739
H	1.526851	-2.443373	-3.512900
C	-1.896315	-3.133271	-1.766292
H	-1.312131	-1.587971	-0.360751
C	-1.538086	-3.846164	-2.917893
H	-0.025407	-4.147452	-4.446175
H	-2.853210	-3.327975	-1.270981
H	-2.212217	-4.605930	-3.326161
C	-4.369116	-0.902861	2.315546
H	-4.671210	-1.325599	3.286677
H	-4.697505	0.147159	2.268956
C	-4.927446	-1.703741	1.133021
C	-6.457538	-1.629781	1.045347
H	-4.599528	-2.757259	1.219096
H	-4.481880	-1.294601	0.208451
H	-6.904453	-2.027326	1.976550
H	-6.761526	-0.567202	0.983790
C	-2.343039	1.559099	-1.086893
C	-2.878959	0.546470	-1.906521
C	-3.218930	2.403983	-0.377224
C	-4.266375	0.386958	-2.020077
H	-2.222269	-0.131765	-2.459317
C	-4.605007	2.240125	-0.491109
H	-2.816454	3.191088	0.267350
C	-5.133407	1.231699	-1.311295
H	-4.668505	-0.397977	-2.669019
H	-5.275596	2.910174	0.056924
H	-6.217415	1.110329	-1.405808
C	-0.332113	3.471575	-0.378851
C	-0.614261	4.518382	-1.279126
C	0.070163	3.771914	0.937199
C	-0.476917	5.849882	-0.867043
H	-0.952513	4.297906	-2.297477
C	0.198214	5.105080	1.347925
H	0.299488	2.959680	1.637059
C	-0.070587	6.143476	0.444544
H	-0.691760	6.661227	-1.569783
H	0.515756	5.331935	2.370387
H	0.035653	7.185604	0.762295
C	-7.009476	-2.394490	-0.162846
H	-6.743777	-3.465623	-0.113125
H	-8.108855	-2.322395	-0.210751
H	-6.601551	-1.989944	-1.106865
C	0.880837	-3.182932	2.257843
H	1.949370	-3.424338	2.361182
H	0.289224	-3.867276	2.889959
H	0.569595	-3.314490	1.208336
C	0.990446	-1.524648	4.197894
H	0.332123	-2.163505	4.811256
H	2.038402	-1.802328	4.379057
H	0.837096	-0.472279	4.488803

^{4}S-3

Lowest frequency = 18.3731 cm^{-1}

Charge = 1, Multiplicity = 4

94

P	0.969414	-0.930458	-1.367877
P	-0.112552	1.963668	-0.704351
C	1.376075	0.400930	-2.594579
H	2.400267	0.716162	-2.338567
H	1.401774	0.000885	-3.622404
C	0.400395	1.584734	-2.478040
H	-0.527142	1.383281	-3.038476
H	0.847136	2.498443	-2.904349
C	2.676421	0.967002	0.468577
C	3.571741	0.109550	1.163459
C	3.255113	1.983502	-0.325655
C	4.964337	0.241897	1.081003
C	4.649356	2.120980	-0.423200
H	2.629900	2.694497	-0.876634
C	5.506874	1.253936	0.279336
H	5.593036	-0.459251	1.640250
H	5.071469	2.917182	-1.046254

H	6.592128	1.375589	0.199994
C	2.952143	-0.971725	1.978707
N	1.567174	-0.871427	1.917387
C	0.725494	-1.777006	2.736114
C	-0.675591	-1.278429	2.492933
C	-1.938349	-1.605632	2.960865
N	-0.850494	-0.228207	1.624484
N	-2.121523	0.112712	1.536055
N	-2.777812	-0.713282	2.345783
H	-2.296869	-2.356660	3.661517
O	3.576075	-1.828474	2.608732
Fe	0.817870	0.352086	0.709332
C	2.293323	-2.171112	-1.498200
C	1.987271	-3.547725	-1.504652
C	3.641311	-1.754006	-1.479263
C	3.019997	-4.493364	-1.491126
H	0.944093	-3.878951	-1.528373
C	4.665936	-2.707599	-1.468603
H	3.896491	-0.690295	-1.464127
C	4.359070	-4.076188	-1.469293
H	2.776624	-5.560567	-1.500320
H	5.708792	-2.376116	-1.451902
H	5.163737	-4.817897	-1.454084
C	-0.554865	-1.729458	-1.989821
C	-0.893904	-1.740574	-3.358735
C	-1.433950	-2.317631	-1.057250
C	-2.100518	-2.314109	-3.781693
H	-0.218995	-1.303253	-4.100781
C	-2.628003	-2.910562	-1.486824
H	-1.181356	-2.304614	0.006926
C	-2.969126	-2.898970	-2.847201
H	-2.358645	-2.312053	-4.845451
H	-3.298002	-3.375672	-0.757671
H	-3.908675	-3.350769	-3.181195
C	-4.247254	-0.640337	2.383726
H	-4.569483	-1.000903	3.373466
H	-4.499372	0.427584	2.294441
C	-4.869880	-1.447123	1.239023
C	-6.390252	-1.264662	1.145650
H	-4.623441	-2.517854	1.371610
H	-4.395776	-1.116137	0.298011
H	-6.864568	-1.589390	2.091331
H	-6.616072	-0.186357	1.039308
C	-1.946935	2.021774	-0.788733
C	-2.667592	1.066345	-1.531968
C	-2.649130	2.949677	0.005460
C	-4.067320	1.048571	-1.490906
H	-2.147679	0.320090	-2.138813
C	-4.048860	2.923597	0.049223
H	-2.101470	3.694637	0.591109
C	-4.762061	1.973489	-0.697262
H	-4.613858	0.309271	-2.085515
H	-4.584486	3.656191	0.661879
H	-5.856723	1.962833	-0.671414
C	0.330854	3.704825	-0.363719
C	0.033095	4.728516	-1.286535
C	0.929115	4.025422	0.869941
C	0.348245	6.057621	-0.978990
H	-0.455826	4.491711	-2.237948
C	1.233790	5.358612	1.176271
H	1.172766	3.225597	1.578389
C	0.947590	6.372819	0.251318
H	0.120890	6.850831	-1.698294
H	1.701892	5.603044	2.134907
H	1.191082	7.413439	0.488286
C	-6.995698	-2.037411	-0.031650
H	-6.810449	-3.122287	0.065289
H	-8.086547	-1.886403	-0.088469
H	-6.557072	-1.705322	-0.990313
C	0.871713	-3.240725	2.250851
H	1.908970	-3.567913	2.414781
H	0.185129	-3.902281	2.806824
H	0.644990	-3.309475	1.174246
C	1.059320	-1.653120	4.243519
H	0.366907	-2.276037	4.835738
H	2.090619	-1.991786	4.416989
H	0.960493	-0.604206	4.568565

^{6}S-3

Lowest frequency = 15.4874 cm^{-1}

Charge = 1, Multiplicity = 6

94

P	-1.447875	-0.375216	1.375422
P	0.906746	1.698574	0.698683
C	-1.247436	1.129642	2.446204

H	-1.896994	1.908972	2.009207
H	-1.593932	0.922135	3.473812
C	0.223242	1.569955	2.437618
H	0.837686	0.825688	2.970999
H	0.351859	2.535084	2.956265
C	-2.531511	1.233891	-1.059665
C	-3.437193	0.219068	-1.492920
C	-3.073163	2.455327	-0.606396
C	-4.823769	0.424393	-1.479155
C	-4.463023	2.658721	-0.588095
H	-2.412404	3.263496	-0.270242
C	-5.336279	1.647380	-1.025935
H	-5.477274	-0.384656	-1.820752
H	-4.868324	3.614842	-0.238157
H	-6.417856	1.817250	-1.012604
C	-2.881801	-1.098342	-1.956054
N	-1.501709	-1.139714	-1.831999
C	-0.701200	-2.223182	-2.417178
C	0.722428	-1.709608	-2.311080
C	1.970124	-2.234141	-2.614645
N	0.947983	-0.446323	-1.826735
N	2.234812	-0.163255	-1.810092
N	2.855510	-1.240746	-2.293931
H	2.291112	-3.190836	-3.021370
O	-3.582598	-2.022391	-2.382204
Fe	-0.738328	0.376844	-0.837250
C	-3.166272	-0.925280	1.527880
C	-3.500164	-2.206592	1.039144
C	-4.185768	-0.053518	1.960303
C	-4.838524	-2.607801	0.984456
H	-2.713792	-2.880172	0.685651
C	-5.522761	-0.466867	1.908483
H	-3.948243	0.950792	2.321197
C	-5.851399	-1.738384	1.417108
H	-5.091347	-3.596550	0.590332
H	-6.311318	0.213393	2.245018
H	-6.898929	-2.051875	1.367893
C	-0.384995	-1.656687	2.126888
C	-0.768848	-2.376288	3.277002
C	0.865089	-1.912957	1.526538
C	0.098296	-3.332301	3.821319
H	-1.746663	-2.194528	3.735615
C	1.732330	-2.864352	2.080636
H	1.153963	-1.362445	0.623917

C	1.348064	-3.574619	3.227290
H	-0.201772	-3.892886	4.712399
H	2.703404	-3.053694	1.611581
H	2.019524	-4.324472	3.657699
C	4.325385	-1.285218	-2.288399
H	4.643508	-1.853943	-3.177086
H	4.658882	-0.242220	-2.398268
C	4.860618	-1.902205	-0.990734
C	6.393468	-1.872518	-0.921891
H	4.498101	-2.944795	-0.906752
H	4.437107	-1.337714	-0.140361
H	6.814139	-2.404307	-1.796772
H	6.735002	-0.823015	-1.002276
C	2.719165	1.527264	0.923970
C	3.269928	0.631113	1.863279
C	3.584889	2.245451	0.071757
C	4.659950	0.480139	1.966628
H	2.625298	0.045952	2.525732
C	4.971454	2.084451	0.174115
H	3.170591	2.936307	-0.669095
C	5.514887	1.205427	1.124484
H	5.073196	-0.203960	2.714930
H	5.631959	2.657961	-0.484502
H	6.599944	1.090414	1.211800
C	0.684602	3.449972	0.213285
C	1.066936	4.514690	1.055921
C	0.147264	3.728674	-1.058462
C	0.894813	5.837619	0.632756
H	1.512261	4.311407	2.036057
C	-0.020716	5.055136	-1.480814
H	-0.150891	2.903280	-1.717827
C	0.349304	6.108480	-0.633509
H	1.191283	6.661705	1.289526
H	-0.443079	5.262429	-2.468940
H	0.216635	7.145018	-0.959616
C	6.934576	-2.494825	0.369794
H	6.634955	-3.554498	0.458389
H	8.036229	-2.453268	0.399778
H	6.550474	-1.961172	1.257415
C	-0.870675	-3.533979	-1.612275
H	-1.923886	-3.848688	-1.673293
H	-0.232140	-4.332830	-2.027486
H	-0.593443	-3.372168	-0.557280
C	-1.043542	-2.444902	-3.912777

H	-0.359600	-3.194522	-4.346531
H	-2.080296	-2.799223	-4.003560
H	-0.933497	-1.499953	-4.469813

[1]S-3'

Lowest frequency = 12.9129 cm^{-1}

Charge = -1, Multiplicity = 1

109

P	0.573080	-1.969385	-0.930675
P	-0.333589	0.793524	-1.679893
C	0.619473	-1.617433	-2.773085
H	1.646932	-1.279664	-2.983944
H	0.416520	-2.532518	-3.357217
C	-0.373860	-0.496562	-3.080488
H	-1.397153	-0.904670	-3.129267
H	-0.163533	-0.017551	-4.052149
C	2.443700	-0.034338	-0.474109
C	3.324449	-0.554325	0.526885
C	3.072300	0.306880	-1.697185
C	4.702788	-0.713119	0.329385
C	4.454768	0.149760	-1.910923
H	2.480883	0.719774	-2.524303
C	5.281713	-0.363607	-0.898545
H	5.288457	-1.122202	1.161463
H	4.886444	0.437538	-2.879430
H	6.358376	-0.489418	-1.068820
C	2.713454	-0.926619	1.838821
N	1.371426	-0.760512	1.780505
C	0.565830	-0.883502	3.004835
C	-0.833490	-0.553348	2.559273
C	-2.061662	-0.479571	3.190290
N	-1.036313	-0.190958	1.245168
N	-2.307548	0.101499	1.038106
N	-2.938713	-0.072099	2.219559
H	-2.371088	-0.661906	4.217425
O	3.381738	-1.328327	2.827087
Fe	0.540215	-0.027718	0.134592
C	2.024651	-3.075786	-0.681366
C	2.156206	-3.694181	0.578864
C	3.086505	-3.182569	-1.599983
C	3.328799	-4.373605	0.925098
H	1.348847	-3.600153	1.308016
C	4.255117	-3.875395	-1.258481
H	3.027610	-2.689662	-2.573315
C	4.385055	-4.463465	0.007531
H	3.426621	-4.806340	1.926162
H	5.080427	-3.927036	-1.977450
H	5.313215	-4.976746	0.283781
C	-0.821114	-3.204662	-0.876318
C	-0.647777	-4.583852	-1.116598
C	-2.120641	-2.721248	-0.621461
C	-1.747222	-5.453916	-1.109767
H	0.358289	-4.973586	-1.305856
C	-3.221751	-3.590614	-0.625420
H	-2.257959	-1.657884	-0.404056
C	-3.039158	-4.960123	-0.868574
H	-1.595219	-6.523841	-1.296276
H	-4.225244	-3.196316	-0.428623
H	-3.898001	-5.641519	-0.865079
C	-4.372373	0.175993	2.278334
H	-4.675962	0.103052	3.336121
H	-4.548965	1.212379	1.938510
C	-5.152008	-0.804861	1.394443
C	-6.642620	-0.464055	1.290861
H	-5.014617	-1.832063	1.781975
H	-4.694977	-0.773271	0.390557
H	-7.100775	-0.461408	2.300247
H	-6.746910	0.566704	0.899889
C	-2.115693	1.376362	-1.720530
C	-2.401329	2.716478	-1.381933
C	-3.202645	0.511372	-1.955259
C	-3.722891	3.164290	-1.253326
H	-1.576263	3.414246	-1.209943
C	-4.525884	0.961208	-1.850831
H	-3.026430	-0.537751	-2.210058
C	-4.794885	2.288738	-1.485314
H	-3.914377	4.207921	-0.977600
H	-5.351395	0.268330	-2.053967
H	-5.829434	2.640686	-1.396238
C	0.371221	2.283093	-2.567357
C	-0.125594	2.675679	-3.827772
C	1.386671	3.048856	-1.969307
C	0.391720	3.800652	-4.483275
H	-0.940597	2.107508	-4.291968
C	1.898140	4.181750	-2.620447

H	1.764940	2.751469	-0.988149
C	1.407629	4.558694	-3.878152
H	-0.003656	4.092043	-5.463788
H	2.686207	4.768398	-2.135320
H	1.810337	5.442810	-4.386590
C	-7.405217	-1.434964	0.381835
H	-7.338712	-2.471302	0.760866
H	-8.474795	-1.168933	0.308819
H	-6.983647	-1.428363	-0.639666
C	0.607433	-2.317977	3.590359
H	1.661831	-2.578423	3.773695
H	0.036443	-2.372504	4.537098
H	0.160907	-3.034493	2.879362
C	1.024229	0.140555	4.076789
H	0.399467	0.059622	4.987478
H	2.075289	-0.075534	4.325517
H	0.946358	1.161202	3.670828
C	0.760943	1.811750	0.951228
C	-0.350935	2.655111	1.204698
C	2.003345	2.331552	1.383808
C	-0.243079	3.919644	1.801573
H	-1.356923	2.313514	0.933861
C	2.145551	3.596510	1.986548
H	2.912616	1.736853	1.249867
C	1.015450	4.405803	2.191601
H	-1.126504	4.546405	1.975073
H	3.143047	3.929234	2.292953
O	1.042701	5.667828	2.765758
C	2.299546	6.164343	3.167236
H	3.004732	6.253410	2.314390
H	2.119622	7.166416	3.590998
H	2.774137	5.524350	3.940172

^{3}S-3'

Lowest frequency = 14.9616 cm^{-1}

Charge = -1, Multiplicity = 3

109

P	0.811151	-1.869966	-0.995446
P	-0.607548	0.782090	-1.724659
C	0.787300	-1.429100	-2.805208
H	1.720747	-0.871039	-2.990798
H	0.782052	-2.337210	-3.434977
C	-0.432085	-0.543841	-3.062547
H	-1.349841	-1.154679	-3.036683
H	-0.382123	-0.060247	-4.053924
C	2.546134	0.146423	-0.342521
C	3.399284	-0.411620	0.660544
C	3.195865	0.598918	-1.515375
C	4.790992	-0.494808	0.509598
C	4.591876	0.523082	-1.673455
H	2.610540	1.040763	-2.331176
C	5.399113	-0.024804	-0.662295
H	5.365768	-0.939006	1.330782
H	5.051426	0.900553	-2.597012
H	6.486635	-0.086673	-0.792280
C	2.753278	-0.912286	1.914892
N	1.400902	-0.777715	1.832529
C	0.566695	-0.986021	3.021431
C	-0.826275	-0.607186	2.574821
C	-2.070280	-0.588711	3.185469
N	-1.004727	-0.111879	1.306512
N	-2.266595	0.201243	1.097608
N	-2.919853	-0.074250	2.240656
H	-2.406696	-0.865753	4.182506
O	3.402872	-1.360320	2.890446
Fe	0.648843	0.091496	0.194885
C	2.325654	-2.888187	-0.804190
C	2.482192	-3.617596	0.393562
C	3.402501	-2.832777	-1.711207
C	3.690828	-4.255914	0.687496
H	1.662077	-3.648241	1.115402
C	4.609610	-3.479477	-1.417964
H	3.318360	-2.250734	-2.632189
C	4.761604	-4.186435	-0.216572
H	3.804170	-4.785353	1.639317
H	5.444789	-3.406463	-2.123453
H	5.716291	-4.668969	0.021542
C	-0.512100	-3.164010	-0.924881
C	-0.292761	-4.506324	-1.311094
C	-1.804675	-2.781822	-0.507093
C	-1.338596	-5.436763	-1.277749
H	0.707447	-4.816962	-1.632218
C	-2.852232	-3.713179	-0.483112
H	-1.982268	-1.746031	-0.204187
C	-2.624140	-5.045332	-0.865157

H	-1.151689	-6.474888	-1.577647
H	-3.849627	-3.396969	-0.158739
H	-3.441346	-5.775313	-0.839854
C	-4.371526	0.072903	2.253981
H	-4.687566	0.187877	3.304913
H	-4.596759	1.004901	1.709072
C	-5.067741	-1.111027	1.572914
C	-6.581824	-0.912061	1.442783
H	-4.845000	-2.040860	2.130760
H	-4.623073	-1.214094	0.567621
H	-7.031949	-0.778803	2.447177
H	-6.766899	0.027618	0.888325
C	-2.415365	1.118137	-1.833739
C	-2.899597	2.416378	-1.496251
C	-3.384994	0.078084	-1.942866
C	-4.253181	2.655468	-1.270286
H	-2.186236	3.244045	-1.413200
C	-4.743194	0.324440	-1.722597
H	-3.077214	-0.938248	-2.207759
C	-5.200965	1.608101	-1.367797
H	-4.585213	3.672708	-1.027582
H	-5.461197	-0.497257	-1.843317
H	-6.267733	1.798439	-1.204193
C	0.030413	2.286856	-2.606791
C	-0.531974	2.654914	-3.849784
C	1.060976	3.075468	-2.066671
C	-0.071962	3.787762	-4.533962
H	-1.354475	2.060591	-4.264473
C	1.519122	4.211676	-2.751372
H	1.492055	2.797766	-1.101276
C	0.957184	4.570181	-3.985682
H	-0.520718	4.063568	-5.495802
H	2.316566	4.818727	-2.308662
H	1.316661	5.458040	-4.519457
C	-7.267949	-2.077061	0.720450
H	-7.115214	-3.028945	1.261880
H	-8.356076	-1.911696	0.626583
H	-6.855733	-2.203587	-0.296854
C	0.591906	-2.461694	3.489855
H	1.637517	-2.733315	3.704368
H	-0.024243	-2.601341	4.398739
H	0.192165	-3.116243	2.696129
C	0.999600	-0.039518	4.172293
H	0.336447	-0.158371	5.050246
H	2.035956	-0.286293	4.451216
H	0.955034	1.004730	3.822031
C	0.774106	1.969331	0.933861
C	-0.375474	2.796456	0.997206
C	1.948160	2.518374	1.492346
C	-0.359775	4.082919	1.549850
H	-1.328072	2.420245	0.605123
C	1.995150	3.806531	2.059376
H	2.873733	1.932089	1.487434
C	0.834456	4.599613	2.082579
H	-1.261746	4.705416	1.579951
H	2.939650	4.174587	2.473584
O	0.770811	5.878296	2.605939
C	1.957214	6.418098	3.146886
H	2.763611	6.493169	2.388345
H	1.704345	7.429539	3.505072
H	2.337875	5.816495	3.998113

[5]S-3'

Lowest frequency = 10.1089 cm^{-1}

Charge = -1, Multiplicity = 5

109

P	2.060869	1.318309	0.662065
P	-0.614757	0.028761	1.909676
C	1.928768	1.094207	2.510190
H	2.320194	0.082737	2.714435
H	2.551473	1.833897	3.045892
C	0.460046	1.190239	2.931850
H	0.087154	2.216214	2.769118
H	0.336098	0.960946	4.005301
C	2.182101	-1.599933	0.349060
C	3.067943	-1.730594	-0.760627
C	2.619866	-2.202900	1.551083
C	4.297149	-2.404356	-0.674503
C	3.846246	-2.886526	1.652873
H	1.991695	-2.152427	2.451566
C	4.693452	-2.985393	0.537220
H	4.920672	-2.447075	-1.575297
H	4.142846	-3.338892	2.609494
H	5.655668	-3.507322	0.616383
C	2.676547	-1.116472	-2.081765

N	1.464034	-0.513620	-2.005240	C	-4.715452	0.473130	2.282795
C	0.848734	0.058797	-3.207869	H	-3.287444	-1.147262	2.301280
C	-0.489692	0.585717	-2.734777	C	-3.794535	2.708335	2.144295
C	-1.709833	0.807010	-3.345488	H	-1.653856	2.859895	2.004925
N	-0.643016	0.905278	-1.407551	C	-4.915993	1.864117	2.232354
N	-1.869882	1.328224	-1.168934	H	-5.575692	-0.203418	2.352298
N	-2.532134	1.271370	-2.346789	H	-3.927423	3.796438	2.107126
H	-2.063422	0.658086	-4.363394	H	-5.927932	2.283361	2.262258
O	3.418052	-1.195294	-3.092419	C	-0.769907	-1.449314	3.016037
Fe	0.456906	-0.553567	-0.230502	C	-1.132375	-1.318374	4.373583
C	3.850039	1.134300	0.324100	C	-0.527223	-2.731333	2.492440
C	4.299319	1.448976	-0.977015	C	-1.235107	-2.449169	5.194303
C	4.753208	0.541380	1.228909	H	-1.355685	-0.326124	4.783090
C	5.612620	1.170654	-1.365272	C	-0.640475	-3.863986	3.312502
H	3.597684	1.874402	-1.699823	H	-0.253692	-2.824431	1.436346
C	6.069425	0.264685	0.836786	C	-0.988649	-3.726313	4.663918
H	4.423708	0.256231	2.231101	H	-1.514727	-2.335560	6.248433
C	6.503668	0.574367	-0.459292	H	-0.451505	-4.856135	2.888135
H	5.930252	1.390205	-2.389908	H	-1.071908	-4.611740	5.305160
H	6.751049	-0.219939	1.544679	C	-6.336068	4.133594	-0.732825
H	7.526565	0.333318	-0.769930	H	-6.004261	5.028077	-1.291813
C	1.804894	3.145163	0.513108	H	-7.433383	4.193417	-0.619320
C	2.833272	4.106283	0.628551	H	-5.886424	4.183559	0.274359
C	0.492739	3.585950	0.227918	C	1.686803	1.237877	-3.769177
C	2.551141	5.471003	0.481900	H	2.695186	0.861423	-4.003899
H	3.857907	3.776388	0.832684	H	1.220459	1.659944	-4.679340
C	0.211078	4.952855	0.097391	H	1.754299	2.036884	-3.010752
H	-0.300286	2.845737	0.080387	C	0.631634	-1.020874	-4.294111
C	1.238474	5.901196	0.223293	H	0.132084	-0.594733	-5.185812
H	3.359573	6.206141	0.576096	H	1.614817	-1.423574	-4.582845
H	-0.814236	5.273697	-0.121255	H	0.008493	-1.833475	-3.884771
H	1.021697	6.969701	0.109713	C	-1.017494	-1.949709	-0.694423
C	-3.980122	1.457888	-2.353553	C	-2.413448	-1.726069	-0.636691
H	-4.298169	1.495545	-3.409326	C	-0.616862	-3.181963	-1.255536
H	-4.444418	0.565739	-1.891333	C	-3.352593	-2.655117	-1.105732
C	-4.392700	2.723378	-1.596790	H	-2.787076	-0.780010	-0.222037
C	-5.914132	2.846000	-1.449504	C	-1.534101	-4.137677	-1.735980
H	-3.983267	3.608244	-2.121387	H	0.454656	-3.412653	-1.331563
H	-3.923550	2.691608	-0.598623	C	-2.913088	-3.870870	-1.660495
H	-6.395840	2.801797	-2.447378	H	-4.431905	-2.465125	-1.050629
H	-6.288523	1.971226	-0.883778	H	-1.163395	-5.073842	-2.167117
C	-2.282770	0.777396	2.172308	O	-3.901091	-4.733405	-2.101867
C	-3.423277	-0.061687	2.257391	C	-3.489230	-5.960077	-2.665399
C	-2.502050	2.175049	2.101182	H	-2.918566	-6.579265	-1.942708

H	-4.409290	-6.496058	-2.950799
H	-2.860232	-5.813283	-3.567698

[1]S-3''

Lowest frequency = 16.4674 cm^{-1}

Charge = 0, Multiplicity = 1

111

P	0.672641	-1.482116	-1.201572
P	-1.136815	0.836333	-1.687000
C	0.607514	-0.996470	-3.010616
H	1.526041	-0.418051	-3.197990
H	0.623979	-1.884698	-3.664072
C	-0.627240	-0.121482	-3.259041
H	-1.487969	-0.734475	-3.571761
H	-0.440140	0.609614	-4.062719
C	1.780695	1.084786	-0.787862
C	2.908287	0.923639	0.082371
C	2.075731	1.770062	-1.997464
C	4.181115	1.481497	-0.184094
C	3.354600	2.263180	-2.309476
H	1.281383	1.944062	-2.732821
C	4.418857	2.144131	-1.398163
H	4.956295	1.534323	0.605216
H	3.513937	2.781586	-3.262318
H	5.395723	2.590304	-1.607938
C	2.659881	0.097680	1.279279
N	1.384951	-0.156587	1.470585
C	0.869811	-0.796675	2.698638
C	-0.624817	-0.808877	2.493345
C	-1.706876	-1.146554	3.285848
N	-1.128370	-0.427058	1.271177
N	-2.443388	-0.503115	1.269945
N	-2.801889	-0.935789	2.492341
H	-1.788502	-1.492172	4.314206
O	3.671637	-0.369611	1.986519
Fe	0.157508	0.310202	-0.003782
C	2.401044	-2.155996	-1.040491
C	2.651978	-3.265296	-0.204448
C	3.509167	-1.496087	-1.616592
C	3.956341	-3.697276	0.071581
H	1.808022	-3.802370	0.237986
C	4.822886	-1.939199	-1.365962
H	3.362087	-0.621927	-2.257343
C	5.056016	-3.040272	-0.509119
H	4.118006	-4.554722	0.732694
H	5.668036	-1.440854	-1.856222
H	6.080016	-3.376879	-0.318111
C	-0.247313	-3.101379	-1.210187
C	0.075104	-4.121747	-2.130982
C	-1.290984	-3.323576	-0.293197
C	-0.634180	-5.329160	-2.137586
H	0.897049	-3.975604	-2.841249
C	-2.002277	-4.533642	-0.298370
H	-1.559525	-2.532570	0.406694
C	-1.677183	-5.537491	-1.220165
H	-0.373010	-6.111171	-2.858910
H	-2.816093	-4.686690	0.418340
H	-2.232789	-6.481400	-1.226504
C	-4.214683	-1.218619	2.738956
H	-4.361582	-1.246176	3.831101
H	-4.784098	-0.367614	2.330522
C	-4.649617	-2.526065	2.065873
C	-6.159019	-2.775919	2.165283
H	-4.090854	-3.369771	2.514707
H	-4.353441	-2.464572	1.003819
H	-6.462281	-2.825914	3.229365
H	-6.693735	-1.909534	1.730781
C	-2.926657	0.356840	-1.574049
C	-3.903834	1.288665	-1.171614
C	-3.308331	-0.988415	-1.743196
C	-5.222021	0.879297	-0.927205
H	-3.630430	2.340906	-1.047509
C	-4.628496	-1.395709	-1.515637
H	-2.568400	-1.737663	-2.040155
C	-5.590250	-0.464102	-1.095580
H	-5.968085	1.617969	-0.613510
H	-4.903751	-2.445936	-1.664822
H	-6.623674	-0.780536	-0.915352
C	-1.315105	2.553824	-2.369780
C	-2.139691	2.769353	-3.495502
C	-0.623416	3.643113	-1.811004
C	-2.270126	4.049101	-4.048708
H	-2.696268	1.932044	-3.932334
C	-0.757388	4.924773	-2.365675
H	0.010703	3.484326	-0.935745

C	-1.578702	5.131930	-3.481843
H	-2.916117	4.203063	-4.920152
H	-0.217304	5.763564	-1.913931
H	-1.684508	6.134740	-3.910246
C	-6.588638	-4.057853	1.442929
H	-6.087639	-4.944699	1.872071
H	-7.678179	-4.216810	1.517911
H	-6.325539	-4.011857	0.370580
C	1.371025	-2.249988	2.863533
H	2.470588	-2.265483	2.900450
H	0.967650	-2.684133	3.795412
H	1.021967	-2.863874	2.018415
C	1.238467	0.069013	3.924855
H	0.782162	-0.346717	4.840915
H	2.333208	0.084413	4.045837
H	0.873470	1.097252	3.771312
C	-0.250420	2.065409	0.925977
C	-1.583855	2.477890	1.170319
C	0.744594	2.938717	1.411609
C	-1.907278	3.678448	1.812167
H	-2.413162	1.835617	0.854374
C	0.451325	4.152051	2.066087
H	1.803189	2.695311	1.273466
C	-0.886221	4.535696	2.260658
H	-2.949572	3.975229	1.974638
H	1.275928	4.783627	2.411143
O	-1.287913	5.701178	2.872102
C	-0.280087	6.585708	3.323813
H	0.362050	6.937495	2.491093
H	-0.801431	7.447894	3.767975
H	0.366786	6.116134	4.092299
Mg	5.201970	-0.674999	0.831356
Cl	7.439192	-0.749973	0.940862

^{3}S-3''

Lowest frequency = 14.5919 cm^{-1}

Charge = 0, Multiplicity = 3

111

P	0.883734	-1.437082	-1.304196
P	-1.269260	0.759612	-1.698425
C	0.666035	-0.849664	-3.056351
H	1.451650	-0.095026	-3.221142
H	0.828266	-1.676882	-3.767776
C	-0.728480	-0.233610	-3.221520
H	-1.478545	-1.026970	-3.373995
H	-0.770263	0.430207	-4.100674
C	1.733270	1.200235	-0.779490
C	2.898307	0.929343	0.001681
C	1.937677	1.974600	-1.944295
C	4.167404	1.462902	-0.316828
C	3.204211	2.480264	-2.288608
H	1.096779	2.213574	-2.604305
C	4.321630	2.242092	-1.468504
H	5.026312	1.335465	0.361598
H	3.314727	3.085190	-3.196236
H	5.298522	2.667987	-1.718632
C	2.686042	0.033197	1.143298
N	1.394157	-0.171163	1.403396
C	0.923351	-0.840334	2.631537
C	-0.577872	-0.857698	2.483354
C	-1.623619	-1.254692	3.298997
N	-1.138791	-0.422734	1.307174
N	-2.452422	-0.522434	1.349823
N	-2.752089	-1.018926	2.559100
H	-1.660665	-1.657066	4.308917
O	3.679889	-0.495339	1.797785
Fe	0.137972	0.421078	0.032769
C	2.574299	-2.058114	-1.184400
C	2.863532	-3.191565	-0.334050
C	3.688128	-1.376153	-1.763784
C	4.164121	-3.567770	-0.046932
H	2.035493	-3.775172	0.078696
C	5.001572	-1.763289	-1.498496
H	3.519791	-0.521809	-2.427904
C	5.287553	-2.842768	-0.570125
H	4.342131	-4.442904	0.588826
H	5.826775	-1.252888	-2.008541
H	6.302705	-3.245859	-0.489426
C	-0.073869	-3.024499	-1.310029
C	0.208365	-4.017004	-2.273023
C	-1.077335	-3.270265	-0.356196
C	-0.511147	-5.217962	-2.288977
H	1.010459	-3.852106	-3.000872
C	-1.793448	-4.477788	-0.366292
H	-1.306843	-2.503583	0.383291

C	-1.515393	-5.451090	-1.334552
H	-0.283895	-5.978879	-3.043387
H	-2.571717	-4.653096	0.384093
H	-2.074422	-6.392955	-1.345772
C	-4.146743	-1.359012	2.843197
H	-4.274346	-1.356054	3.938096
H	-4.757164	-0.547818	2.414829
C	-4.531376	-2.707131	2.221494
C	-6.025444	-3.021724	2.363494
H	-3.925184	-3.509224	2.684424
H	-4.259959	-2.668070	1.151713
H	-6.302167	-3.048558	3.435489
H	-6.608944	-2.195709	1.913303
C	-3.023292	0.209198	-1.499903
C	-4.007147	1.127378	-1.078842
C	-3.376778	-1.147753	-1.630111
C	-5.305247	0.693732	-0.781261
H	-3.754390	2.187736	-0.983334
C	-4.679325	-1.578273	-1.347653
H	-2.632726	-1.887384	-1.938582
C	-5.647413	-0.661169	-0.912073
H	-6.056785	1.421452	-0.455892
H	-4.934974	-2.636860	-1.468435
H	-6.666423	-0.997319	-0.691053
C	-1.547540	2.447484	-2.413816
C	-2.388331	2.581189	-3.540587
C	-0.952346	3.596213	-1.863297
C	-2.623828	3.839519	-4.106607
H	-2.875949	1.697014	-3.967062
C	-1.192777	4.856486	-2.431414
H	-0.304730	3.501914	-0.988768
C	-2.025709	4.982071	-3.550667
H	-3.279030	3.929701	-4.979800
H	-0.724081	5.741703	-1.989114
H	-2.212791	5.967641	-3.990643
C	-6.411057	-4.346240	1.695263
H	-5.860706	-5.193826	2.142438
H	-7.490195	-4.550811	1.801665
H	-6.174561	-4.326399	0.615927
C	1.425258	-2.299319	2.750414
H	2.523922	-2.314708	2.772086
H	1.030899	-2.757536	3.674758
H	1.074612	-2.889814	1.889665
C	1.333572	-0.001828	3.865042
H	0.925037	-0.444560	4.790838
H	2.432555	0.021021	3.935353
H	0.954359	1.027179	3.756288
C	-0.272964	2.159263	0.936237
C	-1.613329	2.525154	1.216455
C	0.717660	3.065043	1.371365
C	-1.950603	3.718033	1.860992
H	-2.428526	1.850430	0.931053
C	0.408502	4.269650	2.026741
H	1.774721	2.844151	1.189270
C	-0.936772	4.608107	2.267095
H	-2.993383	3.987049	2.061549
H	1.220879	4.933390	2.337216
O	-1.351484	5.759019	2.882619
C	-0.360449	6.681270	3.307464
H	0.241984	7.053704	2.455700
H	-0.901633	7.522714	3.765937
H	0.320985	6.232328	4.056509
Mg	5.311547	-1.001948	0.841353
Cl	7.396478	-0.280658	1.287681

[5]S-3''

Lowest frequency = 11.1990 cm^{-1}

Charge = 0, Multiplicity = 5

111

P	-1.256080	-1.301894	1.215174
P	1.412125	0.451755	1.764860
C	-0.912913	-0.667270	2.937523
H	-1.396309	0.321951	3.008860
H	-1.364514	-1.335715	3.691943
C	0.602503	-0.551442	3.139027
H	1.055301	-1.557356	3.140722
H	0.836705	-0.081808	4.109558
C	-1.764996	1.547971	0.476223
C	-2.865530	1.352096	-0.406537
C	-1.994158	2.422369	1.562448
C	-4.085068	2.057741	-0.255250
C	-3.237425	3.048109	1.783445
H	-1.181649	2.633322	2.269811
C	-4.280718	2.892723	0.857109
H	-4.846315	2.065951	-1.057041

H	-3.380453	3.687297	2.663017	C	3.092759	-0.295031	1.643850
H	-5.223905	3.435748	0.976626	C	4.172435	0.525725	1.246573
C	-2.723606	0.310524	-1.464331	C	3.316778	-1.681108	1.774507
N	-1.484277	0.005845	-1.769110	C	5.431207	-0.026753	0.983014
C	-1.117179	-0.986651	-2.783209	H	4.020763	1.604951	1.142152
C	0.387715	-1.118541	-2.643821	C	4.581676	-2.229569	1.525244
C	1.389870	-1.640557	-3.445324	H	2.502628	-2.349935	2.067513
N	0.986946	-0.719069	-1.476724	C	5.644385	-1.407933	1.120323
N	2.282107	-0.963098	-1.506021	H	6.254315	0.628477	0.677086
N	2.535697	-1.512869	-2.704622	H	4.734514	-3.307370	1.651528
H	1.390136	-2.066204	-4.446586	H	6.632232	-1.838348	0.923151
O	-3.813292	-0.234255	-1.956876	C	1.808191	2.039275	2.624427
Fe	-0.042753	0.661103	-0.260944	C	2.550308	2.040345	3.825323
C	-3.050261	-1.605212	1.202247	C	1.386033	3.262678	2.075450
C	-3.597981	-2.713287	0.490610	C	2.848489	3.245672	4.471461
C	-3.958466	-0.650325	1.739671	H	2.910362	1.094286	4.246162
C	-4.975591	-2.857350	0.318774	C	1.689592	4.469918	2.723634
H	-2.926252	-3.465469	0.067887	H	0.838671	3.264371	1.128684
C	-5.344134	-0.803919	1.587028	C	2.416251	4.464195	3.921310
H	-3.580446	0.230563	2.267400	H	3.425188	3.236768	5.402877
C	-5.877582	-1.908216	0.862367	H	1.359275	5.415409	2.280740
H	-5.364499	-3.718771	-0.234441	H	2.654324	5.407080	4.425693
H	-6.021457	-0.070096	2.037963	C	6.162458	-4.757223	-1.459838
H	-6.959222	-2.034643	0.763207	H	5.563907	-5.648062	-1.723686
C	-0.574120	-3.017277	1.343566	H	7.227378	-5.015061	-1.591869
C	-1.100885	-3.985102	2.226137	H	5.994735	-4.546689	-0.388443
C	0.549779	-3.345551	0.561338	C	-1.739000	-2.380281	-2.511396
C	-0.513256	-5.252912	2.319124	H	-2.836018	-2.310375	-2.543377
H	-1.978945	-3.744569	2.835942	H	-1.390672	-3.108669	-3.264952
C	1.139391	-4.615673	0.657644	H	-1.424634	-2.736269	-1.516697
H	0.967927	-2.591625	-0.109461	C	-1.499727	-0.477496	-4.189367
C	0.608742	-5.570679	1.534779	H	-1.159058	-1.180990	-4.970490
H	-0.929196	-5.997021	3.007125	H	-2.595406	-0.382763	-4.252300
H	2.017489	-4.852326	0.046881	H	-1.041331	0.508733	-4.368349
H	1.065893	-6.563132	1.610932	C	0.893327	2.280896	-1.049900
C	3.902675	-1.947122	-2.998281	C	2.290505	2.285802	-1.285734
H	3.953705	-2.162195	-4.077930	C	0.198163	3.467316	-1.368856
H	4.566925	-1.093660	-2.782636	C	2.959915	3.406799	-1.786005
C	4.295787	-3.162514	-2.149865	H	2.877060	1.381066	-1.079669
C	5.769305	-3.551557	-2.320841	C	0.845750	4.606260	-1.881378
H	3.640334	-4.014942	-2.413252	H	-0.885548	3.521230	-1.207825
H	4.097357	-2.910417	-1.093123	C	2.238694	4.580661	-2.082766
H	5.978674	-3.770080	-3.386393	H	4.041993	3.403375	-1.959035
H	6.401588	-2.683522	-2.051854	H	0.259528	5.500080	-2.115717

O	2.973569	5.634393	-2.562391
C	2.288831	6.836091	-2.873529
H	1.787382	7.263657	-1.982666
H	3.054074	7.540996	-3.232747
H	1.531744	6.682454	-3.667762
Mg	-5.417194	-0.143431	-0.854401
Cl	-7.552828	0.417557	-1.276853

^{1}S-3'''

Lowest frequency = 11.8692 cm^{-1}

Charge = 0, Multiplicity = 1

111

P	-0.490648	-1.467331	1.352983
P	1.536292	0.751627	1.627823
C	-0.073165	-1.014289	3.119783
H	-0.816418	-0.267056	3.440565
H	-0.147323	-1.895061	3.780855
C	1.325354	-0.396317	3.125423
H	2.085212	-1.190253	3.040193
H	1.524965	0.149684	4.062103
C	-1.502299	1.059866	0.904524
C	-2.667891	0.926327	0.073799
C	-1.751937	1.708550	2.141269
C	-3.925765	1.479383	0.402727
C	-3.018703	2.194329	2.514907
H	-0.930315	1.864200	2.849208
C	-4.117531	2.100149	1.646136
H	-4.722892	1.574613	-0.360603
H	-3.138745	2.688342	3.486621
H	-5.085321	2.538555	1.908466
C	-2.466404	0.170834	-1.167415
N	-1.217476	-0.191012	-1.364220
C	-0.761817	-0.792697	-2.640221
C	0.718734	-0.980618	-2.435974
C	1.755489	-1.436446	-3.231194
N	1.265042	-0.634611	-1.224009
N	2.564950	-0.848331	-1.229274
N	2.870363	-1.333114	-2.446053
H	1.794201	-1.798287	-4.256494
O	-3.489111	-0.134418	-1.951858
Fe	0.080170	0.232809	0.053728
C	-2.300438	-1.836450	1.419843
C	-2.887514	-2.503936	0.326694
C	-3.149119	-1.299388	2.412633
C	-4.285940	-2.599753	0.205635
H	-2.250013	-2.942025	-0.444320
C	-4.540388	-1.390420	2.302984
H	-2.723938	-0.773292	3.269474
C	-5.123748	-2.026283	1.192782
H	-4.728826	-3.158813	-0.628659
H	-5.176779	-0.949413	3.075813
H	-6.211330	-2.098187	1.096298
C	0.218228	-3.177741	1.243249
C	-0.447225	-4.318152	1.739833
C	1.506375	-3.326944	0.691513
C	0.161781	-5.578939	1.680573
H	-1.447805	-4.218542	2.174262
C	2.118163	-4.588400	0.642525
H	2.024477	-2.449106	0.296437
C	1.446783	-5.717280	1.132967
H	-0.367263	-6.456970	2.067241
H	3.121157	-4.688636	0.213940
H	1.921594	-6.703406	1.089174
C	4.261516	-1.691686	-2.719972
H	4.341120	-1.888391	-3.801371
H	4.878202	-0.808769	-2.480316
C	4.708164	-2.896881	-1.884718
C	6.205156	-3.196844	-2.025886
H	4.107978	-3.781171	-2.171272
H	4.474289	-2.673153	-0.829268
H	6.448363	-3.401789	-3.086798
H	6.779152	-2.292791	-1.744883
C	3.371186	0.586015	1.339378
C	4.100662	1.706085	0.890175
C	4.046397	-0.643775	1.462237
C	5.457006	1.592570	0.555394
H	3.601497	2.675179	0.798023
C	5.407423	-0.754458	1.149781
H	3.510711	-1.538805	1.792080
C	6.117488	0.361798	0.683197
H	6.001186	2.475442	0.202547
H	5.914833	-1.718436	1.270056
H	7.181175	0.276054	0.434959
C	1.569975	2.434959	2.419338
C	2.336600	2.657271	3.582999

C	0.875052	3.513643	1.844803
C	2.389922	3.927513	4.169992
H	2.916200	1.837694	4.023085
C	0.935481	4.787440	2.429370
H	0.295383	3.349412	0.933627
C	1.686801	4.997527	3.593276
H	2.988569	4.085183	5.073965
H	0.390802	5.616814	1.965700
H	1.732082	5.992734	4.049178
C	6.649931	-4.378126	-1.156098
H	6.109712	-5.302129	-1.431494
H	7.730605	-4.573832	-1.264437
H	6.447644	-4.179296	-0.087791
C	-1.420945	-2.161900	-2.927764
H	-2.514934	-2.050216	-2.959553
H	-1.069313	-2.547842	-3.900651
H	-1.139225	-2.892795	-2.151540
C	-1.017530	0.201918	-3.797752
H	-0.598490	-0.194690	-4.739615
H	-2.102230	0.349094	-3.920129
H	-0.541865	1.167758	-3.565311
C	0.509745	1.942785	-0.956044
C	1.817581	2.228405	-1.419809
C	-0.470910	2.891003	-1.320159
C	2.135613	3.375253	-2.157744
H	2.632564	1.528812	-1.205128
C	-0.184441	4.050847	-2.066448
H	-1.512365	2.747153	-1.013695
C	1.132943	4.305228	-2.484648
H	3.160835	3.570042	-2.492532
H	-0.997501	4.742317	-2.308702
O	1.528971	5.408044	-3.205759
C	0.538556	6.360320	-3.544756
H	0.064628	6.799649	-2.643769
H	1.053171	7.155061	-4.106858
H	-0.255823	5.920237	-4.180921
Cl	-7.190222	-0.547717	-1.127737
Zn	-5.044343	-0.464349	-0.902274

^{3}S-3'''

Lowest frequency = 16.5542 cm^{-1}

Charge = 0, Multiplicity = 3

111

P	-0.725568	-1.342844	1.426400
P	1.629241	0.681801	1.650510
C	-0.213063	-0.845570	3.144686
H	-0.847139	0.013228	3.417450
H	-0.404814	-1.666098	3.856966
C	1.261812	-0.444374	3.118258
H	1.887940	-1.343898	2.997528
H	1.569473	0.047493	4.055937
C	-1.465749	1.223465	0.837356
C	-2.650593	1.030762	0.061113
C	-1.620163	1.970104	2.025438
C	-3.898574	1.566691	0.433640
C	-2.860781	2.512481	2.403496
H	-0.761053	2.159563	2.677043
C	-4.004446	2.309945	1.613786
H	-4.780273	1.438924	-0.211058
H	-2.932400	3.098567	3.327398
H	-4.969126	2.733479	1.910951
C	-2.481546	0.218235	-1.146650
N	-1.203583	-0.129492	-1.359627
C	-0.784009	-0.786178	-2.614573
C	0.696979	-0.992172	-2.442277
C	1.709165	-1.491964	-3.246761
N	1.279590	-0.635564	-1.255346
N	2.572989	-0.881045	-1.276193
N	2.839732	-1.393730	-2.482668
H	1.721643	-1.881351	-4.262292
O	-3.465016	-0.102428	-1.924622
Fe	0.086397	0.344780	0.003704
C	-2.530240	-1.611888	1.519776
C	-3.139612	-2.286838	0.466531
C	-3.320173	-1.054840	2.570352
C	-4.590856	-2.343753	0.331289
H	-2.522605	-2.736134	-0.317506
C	-4.727888	-1.242265	2.547729
H	-2.860719	-0.501209	3.390538
C	-5.356942	-1.898098	1.506486
H	-4.991279	-3.217029	-0.211261
H	-5.324829	-0.856881	3.382087
H	-6.445218	-2.020323	1.503404
C	-0.046686	-3.057684	1.308468
C	-0.749332	-4.170601	1.817472

C	1.226432	-3.253364	0.739025
C	-0.183007	-5.451216	1.754246
H	-1.742008	-4.027943	2.256190
C	1.793777	-4.535459	0.684683
H	1.769096	-2.397354	0.331564
C	1.088417	-5.637172	1.189075
H	-0.737918	-6.309043	2.149368
H	2.785026	-4.672702	0.239027
H	1.525759	-6.640262	1.140287
C	4.210669	-1.828323	-2.764320
H	4.290349	-1.973734	-3.853563
H	4.874125	-0.998140	-2.471236
C	4.565218	-3.100345	-1.985878
C	6.039031	-3.495714	-2.138170
H	3.907542	-3.926353	-2.316364
H	4.340341	-2.911322	-0.921564
H	6.272214	-3.666321	-3.207211
H	6.672488	-2.647513	-1.813903
C	3.424738	0.317936	1.347232
C	4.253022	1.351302	0.861559
C	3.977285	-0.968011	1.502537
C	5.584536	1.095339	0.509214
H	3.852585	2.364242	0.759018
C	5.315263	-1.218918	1.171320
H	3.366372	-1.796000	1.872251
C	6.121375	-0.192181	0.658873
H	6.208403	1.911247	0.128594
H	5.728573	-2.223456	1.314849
H	7.166453	-0.389472	0.396271
C	1.825561	2.353768	2.426931
C	2.622567	2.495382	3.583071
C	1.244934	3.497446	1.850796
C	2.817702	3.755094	4.161374
H	3.112438	1.619887	4.024073
C	1.447771	4.759460	2.428703
H	0.637408	3.395253	0.948781
C	2.228779	4.891714	3.584154
H	3.436478	3.851165	5.060056
H	0.988783	5.640052	1.967428
H	2.384272	5.877983	4.034607
C	6.396756	-4.744944	-1.325087
H	5.795844	-5.615129	-1.645512
H	7.461866	-5.008507	-1.441726
H	6.203912	-4.584141	-0.248725
C	-1.455975	-2.168172	-2.813493
H	-2.547771	-2.043882	-2.845315
H	-1.112200	-2.621533	-3.760027
H	-1.183421	-2.844661	-1.986272
C	-1.054631	0.150288	-3.817187
H	-0.678852	-0.302096	-4.752267
H	-2.139645	0.313805	-3.906534
H	-0.552447	1.117296	-3.653111
C	0.570843	2.031228	-0.956142
C	1.903342	2.261982	-1.378761
C	-0.370708	3.016654	-1.325424
C	2.282091	3.400097	-2.097013
H	2.679277	1.522872	-1.153777
C	-0.019115	4.167753	-2.050097
H	-1.420073	2.903380	-1.036160
C	1.319969	4.371046	-2.435271
H	3.319655	3.559482	-2.410205
H	-0.795566	4.895332	-2.303764
O	1.773033	5.458620	-3.131917
C	0.830253	6.453797	-3.500170
H	0.350606	6.909385	-2.611777
H	1.396304	7.225064	-4.043913
H	0.041535	6.043731	-4.160772
Cl	-7.036199	0.133222	-1.358903
Zn	-5.082462	-0.799821	-1.013456

^{5}S-3'''

Lowest frequency = 13.2282 cm^{-1}

Charge = 0, Multiplicity = 5

111

P	0.843921	-1.024069	-1.479303
P	-1.945825	0.634531	-1.596346
C	0.188579	-0.374686	-3.099518
H	0.591883	0.647501	-3.209452
H	0.574038	-0.990931	-3.930729
C	-1.342164	-0.355022	-3.070876
H	-1.726581	-1.384513	-2.970646
H	-1.757429	0.065919	-4.002603
C	1.586084	1.507305	-0.405671
C	2.768001	1.167118	0.312474
C	1.692449	2.481517	-1.413984

C	3.995941	1.799578	0.038426
C	2.922064	3.106634	-1.695655
H	0.809038	2.777306	-1.991104
C	4.075026	2.765430	-0.974619
H	4.889479	1.587982	0.644869
H	2.975449	3.862235	-2.488269
H	5.030993	3.254619	-1.186657
C	2.655128	0.099932	1.337378
N	1.402980	-0.277032	1.581763
C	1.047271	-1.276387	2.592716
C	-0.458237	-1.393270	2.466204
C	-1.442572	-2.053615	3.186306
N	-1.093829	-0.770563	1.425352
N	-2.389358	-1.001454	1.453029
N	-2.609452	-1.771831	2.528406
H	-1.413591	-2.667434	4.083962
O	3.704295	-0.389577	1.920475
Fe	0.005039	0.513733	0.256632
C	2.646874	-1.130605	-1.736913
C	3.384443	-1.970203	-0.906584
C	3.315714	-0.276778	-2.667813
C	4.834899	-1.925685	-0.878798
H	2.866981	-2.639211	-0.212203
C	4.730661	-0.315430	-2.751253
H	2.753852	0.399218	-3.314067
C	5.484993	-1.136168	-1.931296
H	5.351095	-2.842609	-0.549093
H	5.233157	0.319592	-3.489458
H	6.577668	-1.141888	-1.998052
C	0.286199	-2.781886	-1.534099
C	0.990551	-3.776522	-2.246060
C	-0.907333	-3.130449	-0.870227
C	0.509442	-5.092622	-2.281252
H	1.917983	-3.515449	-2.765440
C	-1.392596	-4.445367	-0.918743
H	-1.453769	-2.363515	-0.315405
C	-0.681452	-5.430443	-1.619602
H	1.066430	-5.858530	-2.831766
H	-2.322642	-4.700715	-0.399635
H	-1.052822	-6.460420	-1.650281
C	-3.964099	-2.275891	2.764636
H	-4.056143	-2.487596	3.842469
H	-4.649889	-1.452706	2.508437
C	-4.264494	-3.514048	1.911469
C	-5.720006	-3.979499	2.042042
H	-3.573259	-4.330321	2.195292
H	-4.051213	-3.255516	0.859295
H	-5.946940	-4.200759	3.103197
H	-6.388178	-3.147627	1.747698
C	-3.663356	0.017755	-1.352801
C	-4.630247	0.898998	-0.817705
C	-4.019413	-1.333747	-1.545623
C	-5.907178	0.438009	-0.477043
H	-4.377261	1.954052	-0.669740
C	-5.304175	-1.788083	-1.218592
H	-3.296015	-2.046391	-1.952450
C	-6.251858	-0.909061	-0.673706
H	-6.641651	1.138770	-0.065274
H	-5.566686	-2.836747	-1.397081
H	-7.255285	-1.266644	-0.419003
C	-2.272540	2.292972	-2.341461
C	-3.098193	2.417993	-3.480027
C	-1.719550	3.450116	-1.763265
C	-3.349364	3.676629	-4.037817
H	-3.559020	1.527032	-3.921858
C	-1.973930	4.711057	-2.324978
H	-1.104205	3.361627	-0.863844
C	-2.784193	4.826743	-3.461594
H	-3.991432	3.762851	-4.921131
H	-1.537655	5.602824	-1.863038
H	-2.983293	5.811511	-3.897983
C	-6.022202	-5.211943	1.182340
H	-5.387724	-6.068061	1.474891
H	-7.076190	-5.522726	1.283121
H	-5.831415	-5.005082	0.113714
C	1.674686	-2.664629	2.306498
H	2.771258	-2.575718	2.315690
H	1.361962	-3.394042	3.074574
H	1.341048	-3.033359	1.322260
C	1.431262	-0.777974	4.005708
H	1.093757	-1.491314	4.779025
H	2.525993	-0.675762	4.063880
H	0.966644	0.203765	4.193622
C	-0.745129	2.137786	1.139520
C	-2.133931	2.189616	1.421367
C	0.026346	3.242043	1.559043
C	-2.724139	3.282776	2.059452
H	-2.778378	1.347481	1.142741

C	-0.544138	4.348396	2.212550
H	1.104753	3.257216	1.370958
C	-1.930244	4.378190	2.456064
H	-3.799551	3.314693	2.265207
H	0.099776	5.177456	2.520161
O	-2.589227	5.410090	3.066470
C	-1.829035	6.533472	3.485049
H	-1.328024	7.027713	2.629747
H	-2.544553	7.233461	3.942072
H	-1.063124	6.251253	4.233537
Cl	7.258540	0.143915	1.234758
Zn	5.283169	-0.686976	0.777728

[2]S-4

Lowest frequency = 15.7924 cm^{-1}

Charge = 0, Multiplicity = 2

109

P	0.764061	-1.940593	-0.981472
P	-0.408058	0.827119	-1.687510
C	0.791149	-1.474911	-2.783106
H	1.765728	-0.992945	-2.962647
H	0.713022	-2.372567	-3.420413
C	-0.351067	-0.490795	-3.038052
H	-1.314452	-1.026289	-3.011146
H	-0.266731	-0.013189	-4.028233
C	2.474239	0.109133	-0.343147
C	3.344054	-0.426697	0.649512
C	3.100830	0.556774	-1.528015
C	4.735325	-0.503991	0.489697
C	4.494772	0.484528	-1.702124
H	2.509370	0.989734	-2.342442
C	5.320445	-0.045828	-0.696349
H	5.326848	-0.931552	1.306850
H	4.939057	0.853181	-2.635128
H	6.405016	-0.100221	-0.842175
C	2.695298	-0.906396	1.895442
N	1.327720	-0.764571	1.798301
C	0.497512	-0.973628	3.004757
C	-0.899555	-0.653840	2.553498
C	-2.146332	-0.643226	3.159349
N	-1.084938	-0.225785	1.265462
N	-2.354412	0.045195	1.040522
N	-3.001572	-0.200659	2.188419
H	-2.480358	-0.889272	4.164882
O	3.305125	-1.351985	2.885552
Fe	0.582541	0.019719	0.231972
C	2.275849	-2.941192	-0.731017
C	2.404588	-3.651926	0.479513
C	3.357862	-2.917801	-1.631140
C	3.601023	-4.303310	0.796302
H	1.574679	-3.666382	1.190190
C	4.550982	-3.579512	-1.315045
H	3.291742	-2.356072	-2.565849
C	4.679395	-4.264883	-0.099036
H	3.696095	-4.823530	1.754511
H	5.391188	-3.539097	-2.015965
H	5.621641	-4.762246	0.154130
C	-0.570774	-3.222007	-0.954144
C	-0.325936	-4.569133	-1.292916
C	-1.884289	-2.824084	-0.636849
C	-1.377677	-5.494633	-1.318858
H	0.693023	-4.891281	-1.531653
C	-2.936692	-3.750987	-0.672603
H	-2.077071	-1.785999	-0.354128
C	-2.685645	-5.087962	-1.011539
H	-1.175373	-6.538837	-1.581121
H	-3.953790	-3.427755	-0.426187
H	-3.505394	-5.814185	-1.032344
C	-4.455821	-0.032006	2.215818
H	-4.767736	-0.058951	3.272280
H	-4.670748	0.971839	1.813218
C	-5.162096	-1.106202	1.381367
C	-6.671927	-0.867172	1.261349
H	-4.962049	-2.100970	1.822323
H	-4.705238	-1.098069	0.376431
H	-7.130606	-0.854211	2.269282
H	-6.841984	0.138342	0.830139
C	-2.207785	1.288851	-1.708186
C	-2.569641	2.618174	-1.405337
C	-3.232952	0.349824	-1.932153
C	-3.917035	2.986610	-1.297480
H	-1.790161	3.370264	-1.251683
C	-4.580555	0.723181	-1.846736
H	-2.993016	-0.691599	-2.163379
C	-4.929553	2.040202	-1.514527

H	-4.174896	4.023105	-1.054451
H	-5.361354	-0.020910	-2.040089
H	-5.983304	2.331189	-1.443149
C	0.254453	2.331706	-2.546773
C	-0.242132	2.676207	-3.822322
C	1.202264	3.168145	-1.932229
C	0.216466	3.825156	-4.477211
H	-1.008777	2.055089	-4.299435
C	1.654645	4.323017	-2.587760
H	1.579919	2.913921	-0.939628
C	1.168458	4.652371	-3.859552
H	-0.175660	4.080023	-5.467788
H	2.392252	4.964342	-2.094148
H	1.524434	5.554044	-4.369750
C	-7.364335	-1.922842	0.391934
H	-7.231758	-2.935877	0.813076
H	-8.447817	-1.729801	0.310955
H	-6.944522	-1.929107	-0.630541
C	0.560785	-2.437308	3.506060
H	1.607780	-2.687666	3.732104
H	-0.053268	-2.556710	4.417018
H	0.169440	-3.121992	2.734299
C	0.909688	0.014956	4.126606
H	0.243628	-0.098557	5.001030
H	1.946732	-0.201542	4.424108
H	0.843513	1.048968	3.751961
C	0.704199	1.877394	0.986871
C	-0.462207	2.658732	1.172409
C	1.908993	2.459673	1.437670
C	-0.435533	3.938554	1.737855
H	-1.436812	2.258180	0.875144
C	1.963806	3.738259	2.018331
H	2.850720	1.912043	1.335727
C	0.785056	4.494017	2.163129
H	-1.351568	4.526210	1.863958
H	2.929588	4.130394	2.350610
O	0.727366	5.753366	2.702326
C	1.938611	6.338301	3.151053
H	2.667902	6.453568	2.324862
H	1.672927	7.332235	3.542248
H	2.407328	5.740857	3.957762

^{4}S-4

Lowest frequency = 13.9934 cm^{-1}

Charge = 0, Multiplicity = 4

109

P	1.153306	-1.514265	-1.174200
P	-0.989847	0.865207	-1.639741
C	0.892547	-0.808058	-2.880676
H	1.594890	0.039105	-2.976255
H	1.146654	-1.561759	-3.647001
C	-0.558097	-0.331365	-3.022298
H	-1.239562	-1.196281	-2.950324
H	-0.728095	0.145298	-4.003116
C	2.620554	0.686663	-0.065339
C	3.584658	0.095716	0.792845
C	3.085891	1.470833	-1.137817
C	4.958736	0.281773	0.587042
C	4.465094	1.664949	-1.340311
H	2.379382	1.957142	-1.820410
C	5.404507	1.070421	-0.483020
H	5.652876	-0.208335	1.278513
H	4.804381	2.288637	-2.176534
H	6.476630	1.220624	-0.651605
C	3.084999	-0.753417	1.925320
N	1.719046	-0.831954	1.899719
C	0.981481	-1.501389	2.977030
C	-0.472704	-1.271600	2.625439
C	-1.684278	-1.594844	3.220440
N	-0.779378	-0.566198	1.493262
N	-2.080129	-0.435168	1.348425
N	-2.636005	-1.050262	2.402121
H	-1.938014	-2.132521	4.131416
O	3.839727	-1.300680	2.751715
Fe	0.786942	0.211770	0.449487
C	2.853681	-2.184278	-1.206590
C	3.240094	-3.066913	-0.177057
C	3.822294	-1.730941	-2.122380
C	4.572525	-3.473571	-0.054844
H	2.500440	-3.409994	0.550247
C	5.154571	-2.145539	-1.999461
H	3.552909	-1.028250	-2.914647
C	5.534397	-3.010472	-0.963987
H	4.861691	-4.134555	0.767999
H	5.902017	-1.772162	-2.707040

H	6.580942	-3.315470	-0.859566
C	0.102141	-3.030486	-1.247489
C	0.563059	-4.240434	-1.806635
C	-1.221775	-2.957230	-0.768919
C	-0.286169	-5.352207	-1.885477
H	1.592114	-4.310117	-2.174143
C	-2.072610	-4.068538	-0.860474
H	-1.582912	-2.024966	-0.325368
C	-1.605905	-5.268770	-1.414939
H	0.083859	-6.288014	-2.318221
H	-3.100289	-3.997317	-0.489315
H	-2.267200	-6.139641	-1.477962
C	-4.096433	-1.136263	2.474955
H	-4.363444	-1.301851	3.531417
H	-4.483911	-0.152434	2.165170
C	-4.646599	-2.242349	1.567826
C	-6.179544	-2.282824	1.550056
H	-4.240276	-3.217973	1.895697
H	-4.269892	-2.055897	0.546847
H	-6.561167	-2.422789	2.580323
H	-6.558857	-1.301060	1.207710
C	-2.829370	0.814723	-1.605744
C	-3.522940	1.962572	-1.156359
C	-3.569701	-0.361130	-1.853091
C	-4.907087	1.929592	-0.954069
H	-2.968418	2.887559	-0.965476
C	-4.958128	-0.386625	-1.661290
H	-3.069103	-1.270218	-2.199323
C	-5.634114	0.753470	-1.202614
H	-5.423335	2.831841	-0.608156
H	-5.515352	-1.304616	-1.879052
H	-6.719000	0.730842	-1.053931
C	-0.697622	2.498524	-2.454406
C	-1.354427	2.830146	-3.659706
C	0.175509	3.432946	-1.868046
C	-1.128643	4.068248	-4.271907
H	-2.057232	2.120587	-4.111605
C	0.400518	4.673299	-2.485461
H	0.664572	3.193164	-0.919957
C	-0.247049	4.992165	-3.685789
H	-1.644390	4.316514	-5.206011
H	1.081552	5.390725	-2.015889
H	-0.072960	5.961843	-4.164830
C	-6.726544	-3.391119	0.643443
H	-6.387065	-4.386972	0.981470
H	-7.830038	-3.395367	0.635154
H	-6.381138	-3.256575	-0.397522
C	1.272534	-3.021322	3.028278
H	2.353918	-3.163146	3.178677
H	0.720500	-3.500684	3.857154
H	0.961270	-3.498561	2.083242
C	1.285601	-0.837880	4.344774
H	0.674373	-1.291638	5.146209
H	2.353536	-0.976917	4.571743
H	1.064167	0.240741	4.288108
C	0.460525	2.054162	1.148975
C	-0.870970	2.531131	1.255928
C	1.473667	2.918528	1.615167
C	-1.174880	3.796725	1.763959
H	-1.704014	1.893686	0.936823
C	1.192008	4.193513	2.138402
H	2.521611	2.605708	1.566033
C	-0.139097	4.644710	2.203575
H	-2.208262	4.154185	1.831276
H	2.017554	4.822237	2.484909
O	-0.522483	5.872729	2.675575
C	0.490351	6.757921	3.124976
H	1.202474	7.009014	2.314175
H	-0.025703	7.673367	3.452106
H	1.056703	6.332786	3.976871

^{6}S-4

Lowest frequency = 11.5647 cm^{-1}

Charge = 0, Multiplicity = 6

109

P	-1.687008	-1.390933	0.946966
P	0.876631	0.460910	1.814410
C	-1.438627	-0.858316	2.719927
H	-1.933814	0.123210	2.823289
H	-1.920131	-1.569055	3.414824
C	0.064047	-0.736407	3.008649
H	0.549241	-1.719887	2.883165
H	0.247799	-0.408522	4.046581
C	-2.423461	1.409012	0.147976
C	-3.352876	1.187567	-0.906239

C	-2.879233	2.136068	1.266781	C	3.991373	-3.402606	-1.422029
C	-4.671905	1.663131	-0.838635	C	5.442784	-3.881692	-1.297627
C	-4.200161	2.611220	1.343459	H	3.356216	-4.201217	-1.849920
H	-2.201976	2.347637	2.104481	H	3.590086	-3.170525	-0.419957
C	-5.098944	2.372497	0.291071	H	5.857477	-4.085507	-2.304127
H	-5.340056	1.456300	-1.681454	H	6.054382	-3.064165	-0.869380
H	-4.529045	3.170020	2.228444	C	2.658140	0.017964	1.907976
H	-6.129912	2.738683	0.355159	C	3.622426	1.033788	1.725165
C	-2.915990	0.420010	-2.127746	C	3.096143	-1.318241	2.017628
N	-1.610116	0.016204	-2.044838	C	4.984277	0.717519	1.647707
C	-0.976164	-0.699558	-3.159993	H	3.300815	2.076293	1.636066
C	0.414482	-1.043706	-2.665008	C	4.460669	-1.628901	1.953738
C	1.544618	-1.588319	-3.261749	H	2.374012	-2.129660	2.146856
N	0.745495	-0.804848	-1.362063	C	5.410868	-0.615078	1.760105
N	1.987916	-1.151636	-1.114039	H	5.716406	1.519496	1.503961
N	2.482051	-1.629505	-2.265082	H	4.782759	-2.670985	2.057970
H	1.756865	-1.926741	-4.273565	H	6.477073	-0.860415	1.707984
O	-3.686416	0.203014	-3.082579	C	0.833720	2.054828	2.735602
Fe	-0.584610	0.668436	-0.437394	C	1.331343	2.152405	4.052723
C	-3.498995	-1.525034	0.750150	C	0.315710	3.200724	2.105526
C	-3.982148	-2.116649	-0.436380	C	1.295046	3.376006	4.731010
C	-4.415254	-0.937425	1.643528	H	1.763291	1.271916	4.542172
C	-5.349360	-2.110594	-0.728216	C	0.282370	4.426205	2.788155
H	-3.278323	-2.556649	-1.148104	H	-0.044789	3.129122	1.074947
C	-5.784985	-0.935220	1.348221	C	0.767523	4.515070	4.099287
H	-4.065762	-0.453164	2.558835	H	1.683848	3.444723	5.752794
C	-6.255280	-1.515760	0.162522	H	-0.122541	5.310862	2.285922
H	-5.703817	-2.550513	-1.665645	H	0.742216	5.472261	4.631371
H	-6.484966	-0.460164	2.043504	C	5.573384	-5.133769	-0.422993
H	-7.324576	-1.495181	-0.072859	H	4.994269	-5.975947	-0.843098
C	-1.141801	-3.156128	1.033208	H	6.625202	-5.456423	-0.337304
C	-1.995324	-4.207224	1.429105	H	5.193517	-4.944331	0.597498
C	0.194373	-3.453192	0.690736	C	-1.711770	-2.018657	-3.509411
C	-1.516246	-5.522707	1.491627	H	-2.752639	-1.784473	-3.777552
H	-3.038295	-3.990661	1.683726	H	-1.213118	-2.524703	-4.355664
C	0.674021	-4.768988	0.766805	H	-1.694851	-2.699196	-2.641229
H	0.854579	-2.649926	0.350249	C	-0.877717	0.224367	-4.398766
C	-0.180484	-5.806971	1.165548	H	-0.345000	-0.278424	-5.226657
H	-2.188738	-6.331043	1.799354	H	-1.897691	0.479741	-4.724991
H	1.715132	-4.983529	0.500693	H	-0.338433	1.147185	-4.128082
H	0.189501	-6.836714	1.215930	C	0.628940	2.197588	-1.099098
C	3.856433	-2.138610	-2.278689	C	2.043579	2.169526	-1.129007
H	4.125460	-2.323067	-3.331013	C	0.013363	3.381689	-1.562637
H	4.504569	-1.335800	-1.889042	C	2.804071	3.257212	-1.569054

H	2.572774	1.265124	-0.801148
C	0.752682	4.489633	-2.014250
H	-1.082355	3.458025	-1.568508
C	2.159552	4.430314	-2.011580
H	3.899422	3.228406	-1.583474
H	0.227371	5.383571	-2.363782
O	2.979617	5.449394	-2.420731
C	2.375498	6.648183	-2.878451
H	1.760802	7.123413	-2.088642
H	3.201432	7.322579	-3.151170
H	1.738522	6.471629	-3.767449

^{2}TS(4-5)

Lowest frequency = -318.0510 cm^{-1}

Charge = 0, Multiplicity = 2

109

P	-1.765852	-0.952625	1.121488
P	0.678018	0.802773	1.394685
C	-1.053119	-0.807956	2.865718
H	-1.609397	-0.026295	3.406439
H	-1.212330	-1.755316	3.407798
C	0.430942	-0.431024	2.789081
H	1.055384	-1.296850	2.511495
H	0.792752	-0.027362	3.750167
C	-1.788019	1.331925	-1.030144
C	-2.495649	0.834357	-2.166849
C	-2.425718	2.386674	-0.322782
C	-3.675481	1.427623	-2.633170
C	-3.628181	2.948138	-0.759019
H	-1.957881	2.801241	0.572964
C	-4.248288	2.501166	-1.941419
H	-4.140983	0.986319	-3.522165
H	-4.081624	3.756374	-0.173338
H	-5.174440	2.967464	-2.294136
C	-2.000782	-0.430101	-2.771993
N	-1.013035	-0.965430	-1.999555
C	-0.354001	-2.205512	-2.458081
C	0.728639	-2.435296	-1.445211
C	1.700551	-3.405490	-1.264206
N	0.892379	-1.519813	-0.433407
N	1.888741	-1.880509	0.361659
N	2.380783	-3.019807	-0.142321
H	1.949785	-4.308481	-1.816867
O	-2.474305	-0.924677	-3.819457
Fe	-0.371312	0.008003	-0.399125
C	-3.494624	-0.356451	1.238798
C	-4.265816	-0.443502	0.058711
C	-4.056777	0.258786	2.373008
C	-5.562687	0.079015	0.016757
H	-3.839389	-0.906907	-0.834669
C	-5.355416	0.786869	2.326193
H	-3.492499	0.329257	3.307954
C	-6.109354	0.701830	1.148130
H	-6.136857	0.016905	-0.912561
H	-5.777165	1.264714	3.217187
H	-7.119406	1.123328	1.110219
C	-1.971074	-2.792302	1.028038
C	-3.189178	-3.430550	0.724090
C	-0.827759	-3.592661	1.243869
C	-3.255262	-4.827013	0.615493
H	-4.094529	-2.836742	0.573459
C	-0.896450	-4.986778	1.136461
H	0.126274	-3.120695	1.497692
C	-2.110062	-5.611144	0.812421
H	-4.211937	-5.302835	0.374748
H	0.004252	-5.587755	1.304963
H	-2.164073	-6.700973	0.720267
C	3.551105	-3.627177	0.496827
H	3.287805	-3.833692	1.548822
H	3.724363	-4.591933	-0.006952
C	4.778125	-2.712552	0.415950
C	5.994093	-3.305093	1.139917
H	4.511226	-1.734450	0.853260
H	5.018705	-2.528407	-0.647900
H	5.727026	-3.500696	2.196919
H	6.246341	-4.289827	0.699270
C	2.518146	0.921710	1.264827
C	3.098353	1.074415	-0.008275
C	3.355309	0.848233	2.396006
C	4.489213	1.175064	-0.145443
H	2.455139	1.108901	-0.891976
C	4.747677	0.938581	2.256140
H	2.922110	0.734002	3.395173
C	5.317385	1.110639	0.984623
H	4.923821	1.306777	-1.142077

H	5.388694	0.883164	3.143013
H	6.404391	1.192354	0.876859
C	0.273225	2.376522	2.297776
C	1.224495	3.391718	2.524006
C	-1.047526	2.577724	2.751630
C	0.866021	4.566326	3.200435
H	2.246869	3.269677	2.155545
C	-1.401802	3.746936	3.437586
H	-1.819251	1.833543	2.537521
C	-0.444885	4.746432	3.664840
H	1.617275	5.347362	3.360378
H	-2.434112	3.880872	3.778242
H	-0.722075	5.665776	4.191401
C	7.219497	-2.385841	1.084363
H	7.000992	-1.412230	1.556592
H	8.080832	-2.835549	1.607680
H	7.521914	-2.189517	0.039860
C	-1.334695	-3.403362	-2.489600
H	-2.130714	-3.171346	-3.213260
H	-0.812285	-4.327278	-2.798443
H	-1.777272	-3.562380	-1.496130
C	0.302527	-2.036697	-3.857188
H	0.759448	-2.989227	-4.181388
H	-0.478317	-1.740408	-4.573794
H	1.092754	-1.268101	-3.827209
C	0.033549	1.740387	-1.370805
C	0.419279	2.996779	-0.819201
C	0.621129	1.425641	-2.624801
C	1.391042	3.805075	-1.395067
H	-0.059616	3.357296	0.091810
C	1.606324	2.232008	-3.220677
H	0.319927	0.519976	-3.147473
C	2.021019	3.420953	-2.598318
H	1.681168	4.750894	-0.926969
H	2.034207	1.911571	-4.174879
O	2.985659	4.265412	-3.078253
C	3.610388	3.924978	-4.304817
H	2.879359	3.875739	-5.135476
H	4.341255	4.721886	-4.509159
H	4.138246	2.952580	-4.236909

[4]TS(4-5)

Lowest frequency = -325.0757 cm^{-1}

Charge = 0, Multiplicity = 4

109

P	-1.812073	0.927121	-1.241907
P	0.759133	-0.936154	-1.515810
C	-1.049590	0.661712	-2.938411
H	-1.568015	-0.187011	-3.413146
H	-1.236043	1.551509	-3.563951
C	0.451723	0.349943	-2.837781
H	1.027135	1.231124	-2.503724
H	0.844354	0.026946	-3.817722
C	-1.852102	-1.335240	1.248313
C	-2.547656	-0.723527	2.334434
C	-2.505223	-2.408406	0.594538
C	-3.765596	-1.238201	2.800822
C	-3.744172	-2.888320	1.034435
H	-2.025084	-2.901829	-0.255503
C	-4.368874	-2.331045	2.164557
H	-4.235183	-0.721286	3.645933
H	-4.218279	-3.718073	0.497238
H	-5.323147	-2.730655	2.524588
C	-2.041677	0.578035	2.883732
N	-1.068441	1.095269	2.079095
C	-0.404620	2.347003	2.467252
C	0.696366	2.520137	1.445135
C	1.639821	3.512015	1.216060
N	0.919797	1.546827	0.506725
N	1.918991	1.882351	-0.288174
N	2.362121	3.072198	0.141088
H	1.845649	4.460776	1.706684
O	-2.514969	1.094939	3.917600
Fe	-0.425995	-0.068526	0.531345
C	-3.531889	0.317999	-1.359972
C	-4.298359	0.395083	-0.176064
C	-4.091811	-0.296036	-2.497122
C	-5.595016	-0.127195	-0.134359
H	-3.864132	0.844572	0.721640
C	-5.387869	-0.830335	-2.446847
H	-3.527722	-0.360391	-3.432715
C	-6.141381	-0.747680	-1.267922
H	-6.167131	-0.070073	0.796336
H	-5.809905	-1.308559	-3.337525
H	-7.150526	-1.171386	-1.230499

C	-1.990635	2.762692	-1.209066
C	-3.210130	3.419174	-0.952655
C	-0.824812	3.541056	-1.380451
C	-3.258384	4.817328	-0.859714
H	-4.126986	2.836478	-0.825713
C	-0.878383	4.937106	-1.294413
H	0.133599	3.049585	-1.574860
C	-2.094968	5.582103	-1.025592
H	-4.214935	5.310390	-0.656150
H	0.037323	5.523413	-1.430190
H	-2.136103	6.673497	-0.946871
C	3.522911	3.677308	-0.519218
H	3.273991	3.797413	-1.587891
H	3.651267	4.681545	-0.083953
C	4.780282	2.817534	-0.350340
C	5.987828	3.399965	-1.096456
H	4.557292	1.800774	-0.719050
H	5.006954	2.722422	0.728305
H	5.736578	3.499014	-2.170611
H	6.191144	4.426267	-0.732044
C	2.594495	-0.985477	-1.392286
C	3.165445	-1.169266	-0.116742
C	3.442217	-0.815957	-2.505654
C	4.557572	-1.203920	0.037151
H	2.516388	-1.279807	0.756908
C	4.835296	-0.840387	-2.347557
H	3.016591	-0.673945	-3.504616
C	5.395459	-1.042316	-1.076197
H	4.985739	-1.357846	1.033255
H	5.485093	-0.710690	-3.220175
H	6.483711	-1.073167	-0.955087
C	0.342804	-2.502673	-2.387335
C	1.297197	-3.492949	-2.701489
C	-1.021399	-2.767950	-2.651417
C	0.897800	-4.704007	-3.284153
H	2.354502	-3.317291	-2.480975
C	-1.413835	-3.971785	-3.247977
H	-1.786944	-2.043859	-2.353395
C	-0.454766	-4.946388	-3.566464
H	1.651046	-5.464562	-3.517576
H	-2.475662	-4.155553	-3.444450
H	-0.762055	-5.894518	-4.020035
C	7.247060	2.539469	-0.943264
H	7.076608	1.521873	-1.335956
H	8.100461	2.978538	-1.488141
H	7.536179	2.443711	0.118929
C	-1.381527	3.546634	2.412151
H	-2.185120	3.364770	3.142394
H	-0.865541	4.491954	2.662538
H	-1.816459	3.637020	1.405179
C	0.245960	2.260481	3.875399
H	0.748251	3.211649	4.130189
H	-0.543015	2.049458	4.612731
H	0.998261	1.453751	3.901339
C	0.053526	-1.790430	1.517950
C	0.450639	-3.021354	0.929585
C	0.670097	-1.450858	2.745231
C	1.449138	-3.816969	1.482779
H	-0.022654	-3.363904	0.006093
C	1.683633	-2.241820	3.314774
H	0.359107	-0.543883	3.268922
C	2.092757	-3.426410	2.677288
H	1.757072	-4.750431	1.001455
H	2.134766	-1.921023	4.257973
O	3.077998	-4.260077	3.134169
C	3.729815	-3.914483	4.345275
H	3.019262	-3.872757	5.193895
H	4.472527	-4.705060	4.530474
H	4.247266	-2.937480	4.265370

^{6}TS(4-5)

Lowest frequency = -310.2108 cm^{-1}

Charge = 0, Multiplicity = 6

109

P	0.793066	-1.784420	-1.107549
P	-0.978181	0.999673	-1.699153
C	0.562596	-1.094850	-2.826340
H	1.401825	-0.390599	-2.971155
H	0.651296	-1.897691	-3.577394
C	-0.790864	-0.379887	-2.951177
H	-1.603024	-1.102964	-2.763309
H	-0.933212	0.029734	-3.967366
C	2.660455	0.568272	-0.082041
C	3.433923	-0.371360	0.717573
C	3.315505	1.127982	-1.226282

C	4.791014	-0.607183	0.425066
C	4.645332	0.832689	-1.523320
H	2.777252	1.846051	-1.853739
C	5.407350	-0.014986	-0.678508
H	5.327035	-1.303041	1.080177
H	5.104872	1.279011	-2.411153
H	6.461119	-0.213793	-0.901566
C	2.789444	-1.132560	1.815056
N	1.423586	-0.899727	1.883677
C	0.615946	-1.533342	2.933763
C	-0.798461	-1.068837	2.661754
C	-2.017666	-1.265671	3.288307
N	-1.042302	-0.290158	1.564376
N	-2.328084	0.016432	1.466731
N	-2.926659	-0.576001	2.519373
H	-2.308406	-1.816380	4.180095
O	3.415864	-1.901519	2.575391
Fe	0.636838	0.283119	0.464029
C	2.374741	-2.687133	-1.212182
C	2.683628	-3.634401	-0.208457
C	3.348320	-2.382952	-2.185330
C	3.933435	-4.257684	-0.183488
H	1.953180	-3.858458	0.569489
C	4.600084	-3.009694	-2.152273
H	3.150983	-1.634681	-2.956777
C	4.898344	-3.948590	-1.155343
H	4.161077	-4.971831	0.614201
H	5.350881	-2.751343	-2.907706
H	5.883146	-4.428558	-1.127756
C	-0.504998	-3.086770	-1.055856
C	-0.274027	-4.435509	-1.405825
C	-1.800346	-2.697583	-0.643303
C	-1.318485	-5.368976	-1.347759
H	0.724483	-4.750841	-1.725116
C	-2.844591	-3.633514	-0.610566
H	-1.989689	-1.660614	-0.346664
C	-2.607210	-4.970428	-0.954283
H	-1.124448	-6.412651	-1.622294
H	-3.845421	-3.311758	-0.304755
H	-3.418603	-5.704771	-0.915471
C	-4.372409	-0.438251	2.664348
H	-4.643762	-0.855512	3.647709
H	-4.603653	0.641679	2.669523
C	-5.123732	-1.133945	1.522956

C	-6.635360	-0.881916	1.566711
H	-4.915360	-2.219446	1.567326
H	-4.713923	-0.760238	0.568690
H	-7.046393	-1.227708	2.535634
H	-6.817928	0.209146	1.523204
C	-2.780504	1.233287	-1.505353
C	-3.219192	2.088910	-0.461457
C	-3.752961	0.589379	-2.304307
C	-4.583089	2.311968	-0.250547
H	-2.480584	2.558580	0.196102
C	-5.116568	0.796782	-2.073484
H	-3.446776	-0.073946	-3.120857
C	-5.542452	1.666405	-1.052249
H	-4.902632	2.984074	0.553512
H	-5.854631	0.293440	-2.705879
H	-6.610813	1.835036	-0.881367
C	-0.483350	2.480933	-2.689847
C	-1.141009	3.719297	-2.531905
C	0.637224	2.414624	-3.542715
C	-0.676517	4.860597	-3.201572
H	-2.022998	3.790355	-1.891167
C	1.094688	3.556834	-4.215924
H	1.168596	1.469412	-3.684544
C	0.444695	4.786562	-4.040839
H	-1.199593	5.813397	-3.066342
H	1.964962	3.482550	-4.875854
H	0.807537	5.680830	-4.557912
C	-7.375467	-1.570735	0.414753
H	-7.234889	-2.666399	0.450070
H	-8.459799	-1.368286	0.454557
H	-6.996973	-1.215668	-0.560209
C	0.651013	-3.083268	2.853228
H	1.691927	-3.417553	2.968566
H	0.028552	-3.523651	3.651089
H	0.253664	-3.414747	1.879772
C	1.068102	-1.052850	4.335802
H	0.434280	-1.495833	5.124697
H	2.113378	-1.360076	4.490368
H	0.993100	0.046018	4.394815
C	1.843969	1.973437	0.879273
C	1.313157	3.102636	0.196700
C	2.221972	2.152651	2.233648
C	1.171048	4.343320	0.834899
H	1.047842	3.032242	-0.860341

C	2.085854	3.392036	2.877578
H	2.674926	1.316087	2.773147
C	1.568999	4.503293	2.176646
H	0.779784	5.208296	0.290651
H	2.410259	3.483433	3.918375
O	1.433535	5.756664	2.705643
C	1.855501	5.976612	4.045773
H	2.936023	5.767655	4.168650
H	1.662195	7.039653	4.255744
H	1.284443	5.350796	4.759627

^{2}S-5

Lowest frequency = 12.9481 cm^{-1}

Charge = 0, Multiplicity = 2

109

P	-1.611535	0.856887	-1.205258
P	0.578605	-1.158555	-1.286197
C	-1.275032	0.113554	-2.900230
H	-1.907606	-0.778981	-3.036396
H	-1.528897	0.837953	-3.692606
C	0.198668	-0.291976	-2.917700
H	0.860880	0.590649	-2.966625
H	0.427885	-0.944623	-3.777399
C	-2.021250	-1.739765	1.669651
C	-2.812411	-0.595181	2.012778
C	-2.732792	-2.920414	1.319791
C	-4.214915	-0.705958	2.048916
C	-4.125896	-2.982946	1.283549
H	-2.177101	-3.835347	1.108983
C	-4.884796	-1.867630	1.664868
H	-4.758919	0.180496	2.382713
H	-4.614481	-3.921273	0.997704
H	-5.978891	-1.906149	1.672356
C	-2.303823	0.787797	2.368523
N	-1.201647	1.170985	1.692649
C	-0.519769	2.424759	2.096484
C	0.772911	2.410909	1.325763
C	1.827628	3.297127	1.196690
N	1.080285	1.309777	0.557690
N	2.251892	1.474332	-0.038493
N	2.710028	2.682523	0.351698
H	2.010612	4.285511	1.613006
O	-2.943418	1.479803	3.196184
Fe	-0.335559	-0.019959	0.362911
C	-3.439018	0.796082	-1.013050
C	-4.058228	1.680736	-0.102386
C	-4.238022	-0.160843	-1.670386
C	-5.440831	1.635840	0.108340
H	-3.453732	2.404606	0.448866
C	-5.619681	-0.216012	-1.442864
H	-3.789433	-0.870285	-2.371816
C	-6.228179	0.688317	-0.561494
H	-5.897789	2.333514	0.817797
H	-6.222578	-0.968186	-1.962919
H	-7.308896	0.647240	-0.389102
C	-1.326675	2.640462	-1.668555
C	-2.358357	3.551797	-1.972082
C	0.009851	3.072668	-1.796335
C	-2.059569	4.868188	-2.351482
H	-3.403064	3.235360	-1.911223
C	0.306747	4.385403	-2.185327
H	0.823439	2.373969	-1.589084
C	-0.727150	5.293850	-2.452142
H	-2.876401	5.563729	-2.573441
H	1.352925	4.699516	-2.274679
H	-0.497524	6.324152	-2.744437
C	4.000873	3.146915	-0.156442
H	3.904811	3.306828	-1.245997
H	4.196972	4.124729	0.313232
C	5.116427	2.138783	0.137787
C	6.464312	2.549020	-0.468081
H	4.804119	1.159196	-0.264071
H	5.210168	2.018392	1.234149
H	6.340766	2.698605	-1.558427
H	6.779337	3.528007	-0.056111
C	2.398332	-1.446404	-1.464333
C	3.199345	-1.352679	-0.314289
C	3.013846	-1.737720	-2.699087
C	4.579646	-1.581871	-0.380889
H	2.723271	-1.080142	0.628718
C	4.398952	-1.945517	-2.773178
H	2.413735	-1.811456	-3.611870
C	5.183808	-1.877039	-1.611959
H	5.184716	-1.525682	0.529677
H	4.864688	-2.169179	-3.739296
H	6.263877	-2.052614	-1.668181

C	-0.125114	-2.830217	-1.702643
C	0.642148	-3.954646	-2.062776
C	-1.525751	-2.965534	-1.611914
C	0.017599	-5.179324	-2.341995
H	1.732625	-3.879908	-2.107999
C	-2.150660	-4.183310	-1.909805
H	-2.121803	-2.122604	-1.247020
C	-1.379245	-5.296135	-2.276120
H	0.627846	-6.049157	-2.609431
H	-3.239729	-4.265564	-1.826526
H	-1.862388	-6.254258	-2.495844
C	7.558845	1.504916	-0.217984
H	7.277935	0.530699	-0.656356
H	8.519892	1.815156	-0.663231
H	7.721845	1.350942	0.864285
C	-1.358611	3.676531	1.746776
H	-2.312555	3.610978	2.292605
H	-0.828898	4.598477	2.048564
H	-1.547224	3.725279	0.663756
C	-0.181094	2.442341	3.613577
H	0.310625	3.395845	3.877180
H	-1.112168	2.335313	4.189635
H	0.509789	1.621614	3.868317
C	-0.528147	-1.850247	1.805809
C	0.157374	-3.026039	1.339650
C	0.232164	-1.017432	2.677624
C	1.465162	-3.316920	1.682056
H	-0.360041	-3.729886	0.688533
C	1.569486	-1.302465	3.025175
H	-0.262740	-0.210024	3.209340
C	2.196102	-2.448885	2.530647
H	1.962996	-4.207222	1.288054
H	2.084073	-0.620511	3.706955
O	3.493010	-2.801858	2.776808
C	4.265459	-1.940034	3.599116
H	3.840624	-1.861905	4.618407
H	5.268884	-2.387682	3.654407
H	4.337334	-0.924138	3.162005

[4]S-5

Lowest frequency = 11.0184 cm^{-1}

Charge = 0, Multiplicity = 4

109

P	-2.163055	1.301771	-0.816278
P	0.272330	-0.385189	-1.547988
C	-1.653068	1.310108	-2.631437
H	-2.248259	0.508338	-3.104477
H	-1.931051	2.258663	-3.125565
C	-0.155454	1.003241	-2.749012
H	0.459289	1.859820	-2.419942
H	0.120981	0.736442	-3.784661
C	-1.287445	-2.908997	1.395966
C	-1.844910	-1.934056	2.273525
C	-2.178539	-3.742152	0.679075
C	-3.240840	-1.880845	2.455225
C	-3.564775	-3.651732	0.840323
H	-1.766830	-4.507558	0.014222
C	-4.101868	-2.726318	1.748658
H	-3.641831	-1.152146	3.167084
H	-4.222431	-4.323879	0.278187
H	-5.184359	-2.660756	1.901931
C	-1.011880	-0.985199	3.121872
N	-0.345460	-0.065773	2.390641
C	0.490621	0.925811	3.109132
C	1.125927	1.770338	2.028943
C	2.146101	2.710210	2.013663
N	0.673742	1.671251	0.741679
N	1.337043	2.478802	-0.059310
N	2.229620	3.115592	0.707958
H	2.795470	3.112621	2.788042
O	-1.033846	-1.113709	4.364001
Fe	-0.694195	0.155565	0.445996
C	-3.978706	1.020031	-0.962622
C	-4.469959	-0.246576	-0.598054
C	-4.871010	1.993503	-1.454730
C	-5.831998	-0.546989	-0.738812
H	-3.780020	-0.988278	-0.181592
C	-6.233573	1.694616	-1.587610
H	-4.496766	2.987635	-1.724411
C	-6.715180	0.423180	-1.234425
H	-6.201886	-1.536425	-0.449150
H	-6.924402	2.455672	-1.967205
H	-7.781249	0.193771	-1.339113
C	-2.078012	3.069279	-0.307080
C	-2.673869	3.419750	0.924682

C	-1.310398	4.033819	-0.986874
C	-2.514510	4.704462	1.455165
H	-3.259194	2.670223	1.469943
C	-1.145285	5.319938	-0.450139
H	-0.823917	3.783017	-1.934050
C	-1.743940	5.659665	0.770758
H	-2.986692	4.960853	2.409802
H	-0.544807	6.059677	-0.991887
H	-1.612779	6.663428	1.189079
C	3.213579	3.994119	0.072857
H	2.663499	4.617224	-0.651358
H	3.618189	4.654223	0.857515
C	4.317595	3.186338	-0.619426
C	5.329133	4.075284	-1.353497
H	3.840931	2.483275	-1.325446
H	4.834018	2.564038	0.134749
H	4.794299	4.701097	-2.094481
H	5.794127	4.779629	-0.635763
C	2.116380	-0.390742	-1.589346
C	2.763854	-0.646904	-0.364500
C	2.894205	-0.116535	-2.732084
C	4.162264	-0.647962	-0.286283
H	2.149792	-0.838783	0.524042
C	4.295008	-0.121071	-2.653197
H	2.408640	0.099083	-3.690011
C	4.930701	-0.389807	-1.430818
H	4.651147	-0.852257	0.671648
H	4.892533	0.084834	-3.548294
H	6.024552	-0.391268	-1.372237
C	-0.229921	-1.869677	-2.520610
C	0.639835	-2.641837	-3.315285
C	-1.575977	-2.269605	-2.388769
C	0.167250	-3.791153	-3.964631
H	1.693735	-2.360095	-3.404105
C	-2.050064	-3.408030	-3.052462
H	-2.235611	-1.699656	-1.725618
C	-1.177221	-4.175521	-3.838807
H	0.854377	-4.393937	-4.568724
H	-3.097286	-3.706524	-2.934755
H	-1.540131	-5.076387	-4.345080
C	6.421313	3.264440	-2.060611
H	5.982327	2.580803	-2.808342
H	7.138284	3.924438	-2.578708
H	6.987132	2.647394	-1.339428
C	-0.388266	1.836779	4.004130
H	-0.884031	1.209930	4.761126
H	0.225124	2.607938	4.504700
H	-1.149855	2.338971	3.384305
C	1.605488	0.247898	3.939789
H	2.245157	1.007886	4.425139
H	1.140433	-0.381691	4.712804
H	2.233257	-0.380437	3.286088
C	0.176438	-3.136415	1.266862
C	0.735628	-3.604808	0.054649
C	1.049666	-2.975997	2.361902
C	2.098854	-3.861775	-0.073504
H	0.091211	-3.755830	-0.813306
C	2.423032	-3.234149	2.250771
H	0.648661	-2.672518	3.332670
C	2.958425	-3.671221	1.024837
H	2.523930	-4.196353	-1.023864
H	3.058523	-3.102104	3.130280
O	4.284626	-3.926333	0.805432
C	5.180267	-3.784918	1.896232
H	4.922891	-4.471077	2.726283
H	6.177813	-4.039275	1.507799
H	5.192770	-2.746784	2.283631

[6]S-5

Lowest frequency = 13.2851 cm^{-1}

Charge = 0, Multiplicity = 6

109

P	-1.770111	1.103835	-1.191633
P	0.704458	-1.031849	-1.431832
C	-0.982928	0.734511	-2.865071
H	-1.604076	-0.060957	-3.312259
H	-1.083735	1.627243	-3.507665
C	0.475576	0.281910	-2.757863
H	1.136790	1.105823	-2.438033
H	0.839333	-0.104976	-3.726245
C	-1.893575	-1.961240	1.754147
C	-2.559117	-0.902647	2.493821
C	-2.748051	-2.938187	1.143807
C	-3.955525	-0.942329	2.668896
C	-4.127951	-2.931068	1.305065

H	-2.292341	-3.760217	0.585801
C	-4.752581	-1.935208	2.094160
H	-4.397804	-0.140738	3.269295
H	-4.725468	-3.722324	0.838352
H	-5.836709	-1.936593	2.244843
C	-1.881005	0.314162	3.063203
N	-0.883755	0.816931	2.264379
C	-0.106809	1.979381	2.736306
C	0.904796	2.246648	1.645728
C	1.912857	3.187609	1.485999
N	0.926701	1.459698	0.529028
N	1.868995	1.852446	-0.307082
N	2.474948	2.898929	0.272556
H	2.262662	4.007586	2.109066
O	-2.267037	0.816597	4.135554
Fe	-0.559002	-0.078775	0.535038
C	-3.542934	0.812003	-1.520265
C	-4.176669	-0.242980	-0.839298
C	-4.262969	1.556695	-2.482635
C	-5.514825	-0.557159	-1.113658
H	-3.618576	-0.812268	-0.090316
C	-5.599931	1.241165	-2.750291
H	-3.777355	2.386907	-3.007635
C	-6.227599	0.183564	-2.069294
H	-5.995386	-1.375015	-0.568133
H	-6.157839	1.821552	-3.493475
H	-7.274118	-0.058782	-2.283549
C	-1.649524	2.926290	-1.058732
C	-2.641584	3.640194	-0.350130
C	-0.510757	3.623329	-1.522813
C	-2.505848	5.014886	-0.126891
H	-3.524557	3.110333	0.023175
C	-0.371758	4.996076	-1.280223
H	0.272900	3.091693	-2.069961
C	-1.366686	5.699561	-0.584186
H	-3.290463	5.554419	0.414190
H	0.516069	5.522720	-1.648989
H	-1.258229	6.773626	-0.401021
C	3.605106	3.530604	-0.413785
H	3.228091	3.961207	-1.358727
H	3.943790	4.361557	0.225810
C	4.728146	2.522951	-0.683622
C	5.894879	3.134168	-1.469296
H	4.302283	1.667316	-1.236330
H	5.086489	2.120109	0.281954
H	5.512657	3.555103	-2.419951
H	6.319714	3.987340	-0.904414
C	2.516203	-1.207849	-1.356860
C	3.135642	-1.027728	-0.100059
C	3.321220	-1.465100	-2.489889
C	4.524832	-1.129354	0.024603
H	2.513572	-0.809930	0.772945
C	4.712362	-1.564359	-2.359746
H	2.860668	-1.583392	-3.476957
C	5.318370	-1.401638	-1.101223
H	4.991901	-0.994120	1.005549
H	5.328162	-1.766586	-3.243101
H	6.405962	-1.482743	-1.002158
C	-0.001514	-2.532048	-2.193708
C	0.760561	-3.643702	-2.612408
C	-1.416945	-2.627678	-2.218376
C	0.126255	-4.809775	-3.057968
H	1.852990	-3.604777	-2.563235
C	-2.044235	-3.798303	-2.660482
H	-2.028322	-1.801569	-1.839151
C	-1.278093	-4.893622	-3.087068
H	0.732434	-5.664686	-3.376952
H	-3.138201	-3.853512	-2.657147
H	-1.768307	-5.810572	-3.430135
C	6.996976	2.111453	-1.769298
H	6.599514	1.267192	-2.359609
H	7.824782	2.569943	-2.337305
H	7.415581	1.694166	-0.835720
C	-0.999749	3.231903	2.916358
H	-1.748869	3.022567	3.693810
H	-0.386533	4.102145	3.213381
H	-1.506142	3.467698	1.968308
C	0.646118	1.657646	4.052764
H	1.232978	2.533744	4.382815
H	-0.089901	1.401825	4.830030
H	1.334194	0.810312	3.898647
C	-0.436697	-2.229952	1.811563
C	0.194439	-3.153066	0.907272
C	0.375495	-1.794036	2.910335
C	1.511216	-3.568474	1.065523
H	-0.377326	-3.567917	0.076119
C	1.698634	-2.207224	3.070164
H	-0.070866	-1.176558	3.691124

C	2.287811	-3.097800	2.144050
H	1.965277	-4.262541	0.352576
H	2.253328	-1.858141	3.945585
O	3.577467	-3.558327	2.216191
C	4.369066	-3.143903	3.314932
H	3.934425	-3.471576	4.279805
H	5.353588	-3.616710	3.179878
H	4.493953	-2.042420	3.338618

^{2}S-6

Lowest frequency = 13.1355 cm^{-1}

Charge = 0, Multiplicity = 2

95

P	1.134769	-0.681869	-1.174602
P	-0.478580	1.760301	-0.640351
C	1.180307	0.599328	-2.546144
H	2.050290	1.246979	-2.338756
H	1.311582	0.131587	-3.537461
C	-0.122556	1.396716	-2.457383
H	-0.953969	0.771480	-2.824303
H	-0.101201	2.308515	-3.076867
C	2.644800	1.182430	0.717720
C	3.360637	0.161630	1.386877
C	3.319196	2.067287	-0.148059
C	4.739401	0.026985	1.169919
C	4.691990	1.907678	-0.371663
H	2.770883	2.887816	-0.622352
C	5.404387	0.890569	0.291567
H	5.256862	-0.784471	1.690986
H	5.215204	2.590467	-1.050513
H	6.478175	0.770407	0.112743
C	2.637884	-0.821030	2.253105
N	1.284781	-0.781949	2.060084
C	0.442782	-1.700059	2.862508
C	-0.962524	-1.382628	2.432395
C	-2.225101	-1.802219	2.813080
N	-1.137257	-0.500059	1.388225
N	-2.423526	-0.357421	1.111216
N	-3.083425	-1.143854	1.973025
H	-2.573133	-2.485266	3.585045
O	3.255295	-1.584738	3.025595
Fe	0.456969	0.283604	0.662761
C	2.776525	-1.496125	-1.286484
C	3.033696	-2.563973	-0.400315
C	3.814270	-1.046007	-2.125305
C	4.294443	-3.167042	-0.353995
H	2.242717	-2.908781	0.270094
C	5.077363	-1.653190	-2.079488
H	3.648530	-0.213807	-2.814621
C	5.322495	-2.710647	-1.193186
H	4.478665	-3.980852	0.354733
H	5.876176	-1.289173	-2.734844
H	6.313863	-3.174501	-1.151449
C	0.014457	-1.954774	-1.919748
C	0.479073	-3.014385	-2.724886
C	-1.371253	-1.825454	-1.690759
C	-0.426718	-3.920848	-3.293813
H	1.553674	-3.129354	-2.901901
C	-2.275286	-2.724745	-2.273052
H	-1.733008	-1.023593	-1.039472
C	-1.805398	-3.776300	-3.074192
H	-0.053982	-4.743493	-3.914221
H	-3.349384	-2.608420	-2.091385
H	-2.510200	-4.485065	-3.522606
C	-4.544087	-1.198494	1.884398
H	-4.893256	-1.849956	2.701830
H	-4.927046	-0.178569	2.063108
C	-5.000764	-1.704569	0.512179
C	-6.521332	-1.635757	0.329506
H	-4.640090	-2.740484	0.368920
H	-4.506580	-1.079523	-0.251523
H	-7.022249	-2.246839	1.105805
H	-6.855236	-0.592606	0.492141
C	-2.314565	2.030259	-0.618839
C	-2.858944	2.734803	0.474168
C	-3.196028	1.442409	-1.544901
C	-4.244092	2.833228	0.647129
H	-2.188304	3.199948	1.204752
C	-4.584765	1.544542	-1.376031
H	-2.811462	0.881387	-2.401242
C	-5.115211	2.232325	-0.275850
H	-4.646668	3.384111	1.504451
H	-5.254456	1.083371	-2.110698
H	-6.200043	2.310902	-0.144745
C	0.090585	3.520470	-0.415727

C	0.130509	4.468946	-1.456176
C	0.490687	3.919447	0.875577
C	0.580670	5.774492	-1.215564
H	-0.197412	4.195676	-2.464205
C	0.930260	5.227224	1.121112
H	0.460295	3.187422	1.691884
C	0.984237	6.156524	0.072821
H	0.613260	6.497774	-2.037769
H	1.240410	5.516310	2.130903
H	1.338596	7.176163	0.257986
C	-6.963102	-2.103944	-1.061733
H	-6.663519	-3.152046	-1.243284
H	-8.058574	-2.040257	-1.179749
H	-6.500199	-1.483469	-1.850455
C	0.742372	-3.184425	2.533196
H	1.803176	-3.385672	2.747191
H	0.108975	-3.853970	3.142357
H	0.527884	-3.379003	1.468494
C	0.602171	-1.427723	4.377586
H	-0.082153	-2.070274	4.961146
H	1.642039	-1.636154	4.669161
H	0.368167	-0.371393	4.592467
H	1.651466	1.539657	1.138839

^{4}S-6

Lowest frequency = 14.2204 cm^{-1}

Charge = 0, Multiplicity = 4

95

P	1.303306	-1.098825	-1.097818
P	-0.344161	1.447556	-1.177291
C	1.475848	-0.070839	-2.655542
H	2.327812	0.611326	-2.485537
H	1.690103	-0.698843	-3.538362
C	0.166934	0.708466	-2.830666
H	-0.632411	0.006802	-3.123103
H	0.236450	1.466884	-3.628072
C	2.753710	1.564141	0.649564
C	3.208751	0.659930	1.631861
C	3.572809	1.921238	-0.435297
C	4.507319	0.132871	1.519408
C	4.846928	1.350796	-0.559969
H	3.212402	2.655502	-1.164436
C	5.314110	0.460868	0.423218
H	4.856515	-0.543614	2.304937
H	5.485241	1.611589	-1.411440
H	6.311628	0.019166	0.329334
C	2.339521	0.240883	2.797885
N	1.018040	0.196134	2.475673
C	0.059980	-0.250231	3.507111
C	-1.282783	-0.285621	2.810809
C	-2.596707	-0.355681	3.248768
N	-1.329058	-0.312903	1.439511
N	-2.571839	-0.401158	1.012791
N	-3.346590	-0.426366	2.103218
H	-3.047138	-0.357605	4.238991
O	2.869913	-0.062547	3.885701
Fe	0.469108	0.208980	0.555823
C	2.934259	-1.916023	-0.899861
C	3.143232	-2.610292	0.310202
C	3.979806	-1.849693	-1.839893
C	4.363051	-3.246197	0.564170
H	2.344864	-2.627785	1.061258
C	5.207203	-2.476461	-1.577927
H	3.845718	-1.303325	-2.778240
C	5.399851	-3.178256	-0.379875
H	4.512435	-3.778249	1.509567
H	6.016795	-2.412997	-2.313277
H	6.360204	-3.664348	-0.177059
C	0.216022	-2.474237	-1.684827
C	0.694602	-3.589351	-2.400080
C	-1.162577	-2.363101	-1.415687
C	-0.199253	-4.570828	-2.850797
H	1.767885	-3.690778	-2.596621
C	-2.054374	-3.342123	-1.875411
H	-1.524044	-1.508456	-0.833760
C	-1.574442	-4.447359	-2.593979
H	0.178405	-5.437789	-3.404231
H	-3.124310	-3.243564	-1.659636
H	-2.268301	-5.217863	-2.947674
C	-4.806205	-0.428123	1.945586
H	-5.234686	-0.650365	2.936560
H	-5.114802	0.591637	1.653799
C	-5.257865	-1.440775	0.890764
C	-6.768173	-1.377016	0.631682
H	-4.964017	-2.457683	1.212273

H	-4.708744	-1.224980	-0.041560
H	-7.319842	-1.595678	1.566980
H	-7.040827	-0.342510	0.346345
C	-2.179464	1.627000	-1.329854
C	-2.837659	2.571719	-0.513416
C	-2.968206	0.740987	-2.091667
C	-4.235391	2.630045	-0.462685
H	-2.249126	3.270234	0.090100
C	-4.367915	0.802158	-2.044674
H	-2.499292	-0.019940	-2.721627
C	-5.010253	1.744298	-1.229205
H	-4.722477	3.378704	0.172321
H	-4.958541	0.107381	-2.652438
H	-6.104072	1.794686	-1.197258
C	0.212271	3.217289	-1.267958
C	0.579554	3.890905	-2.449275
C	0.322810	3.905494	-0.039733
C	1.046561	5.212233	-2.402638
H	0.500179	3.389898	-3.419074
C	0.772902	5.231000	0.006713
H	0.062794	3.380408	0.888736
C	1.143472	5.886713	-1.176365
H	1.332850	5.718783	-3.330954
H	0.850561	5.745679	0.970315
H	1.511797	6.917494	-1.143130
C	-7.213347	-2.345137	-0.470108
H	-6.975013	-3.390454	-0.202553
H	-8.300780	-2.281026	-0.647394
H	-6.700155	-2.119007	-1.422453
C	0.380712	-1.693517	3.984695
H	1.384231	-1.699407	4.436059
H	-0.366359	-2.037644	4.722385
H	0.362901	-2.378446	3.119264
C	0.023719	0.727111	4.702254
H	-0.727735	0.414350	5.450649
H	1.018494	0.740801	5.172805
H	-0.224773	1.742589	4.349522
H	1.792086	2.072717	0.795189

^{6}S-6

Lowest frequency = 6.7412 cm^{-1}

Charge = 0, Multiplicity = 6

95

P	-1.203141	-0.954868	1.304967
P	0.259604	1.912725	0.848287
C	-1.465591	0.440169	2.511482
H	-2.313668	1.039489	2.135312
H	-1.720017	0.045297	3.511022
C	-0.180208	1.277799	2.556781
H	0.674410	0.661753	2.890924
H	-0.260470	2.125157	3.258833
C	-2.728812	1.107755	-1.014458
C	-3.110036	0.006953	-1.813215
C	-3.643724	1.698396	-0.124591
C	-4.429051	-0.478766	-1.719150
C	-4.936679	1.177034	-0.009777
H	-3.343545	2.582164	0.447593
C	-5.330514	0.087439	-0.815882
H	-4.712396	-1.316496	-2.362811
H	-5.649833	1.629688	0.687730
H	-6.344502	-0.316847	-0.731138
C	-2.159031	-0.665806	-2.756208
N	-0.833489	-0.564637	-2.394463
C	0.184660	-1.200858	-3.275578
C	1.525325	-0.980276	-2.603239
C	2.832624	-1.007523	-3.049043
N	1.571089	-0.795341	-1.241202
N	2.834222	-0.703328	-0.808103
N	3.605837	-0.841090	-1.915947
H	3.270904	-1.138076	-4.035741
O	-2.590290	-1.286985	-3.744794
Fe	-0.197637	-0.002725	-0.634742
C	-2.804093	-1.800784	1.127529
C	-2.906279	-2.770021	0.104699
C	-3.920771	-1.540152	1.947735
C	-4.097623	-3.476468	-0.080929
H	-2.048762	-2.954764	-0.551608
C	-5.114324	-2.247608	1.752533
H	-3.866551	-0.780270	2.732908
C	-5.206285	-3.215805	0.741723
H	-4.167819	-4.219253	-0.882190
H	-5.979992	-2.034133	2.388706
H	-6.142559	-3.763242	0.590571
C	-0.161213	-2.114323	2.286041
C	-0.693453	-2.896359	3.332849

C	1.204219	-2.237038	1.952452
C	0.133960	-3.768001	4.049547
H	-1.759876	-2.826860	3.576884
C	2.029886	-3.107760	2.678229
H	1.617818	-1.664344	1.115568
C	1.499806	-3.872771	3.726778
H	-0.284662	-4.370883	4.862827
H	3.089371	-3.192892	2.412164
H	2.143208	-4.559541	4.287095
C	5.042391	-0.611763	-1.801261
H	5.480328	-0.802323	-2.794823
H	5.203951	0.455582	-1.554863
C	5.684651	-1.500696	-0.730222
C	7.168256	-1.175181	-0.514794
H	5.560920	-2.561872	-1.017616
H	5.125817	-1.360004	0.212168
H	7.718497	-1.302054	-1.467837
H	7.267286	-0.105817	-0.243577
C	2.003491	2.462137	1.074088
C	2.374348	3.809193	0.881089
C	3.003533	1.500357	1.334469
C	3.720291	4.188355	0.965597
H	1.601114	4.555322	0.668417
C	4.346946	1.886056	1.420394
H	2.735930	0.448063	1.465835
C	4.711303	3.229591	1.235352
H	3.997032	5.239028	0.825167
H	5.113325	1.131952	1.633144
H	5.763230	3.528549	1.297917
C	-0.720147	3.398089	0.559245
C	-1.418431	4.092108	1.587281
C	-0.876027	3.849727	-0.785033
C	-2.251279	5.168815	1.277163
H	-1.310239	3.784413	2.632235
C	-1.709838	4.927238	-1.082086
H	-0.333536	3.340862	-1.591192
C	-2.411683	5.595064	-0.057782
H	-2.782155	5.686865	2.083757
H	-1.819165	5.252093	-2.122488
H	-3.070811	6.435727	-0.295164
C	7.814310	-2.042237	0.571633
H	7.758434	-3.114634	0.310926
H	8.878070	-1.783719	0.712264
H	7.302145	-1.907844	1.541707
C	-0.040598	-2.735681	-3.359281
H	-1.031946	-2.933504	-3.792021
H	0.741748	-3.201767	-3.983414
H	0.016519	-3.169130	-2.346022
C	0.181556	-0.559016	-4.678845
H	0.972417	-1.003490	-5.309609
H	-0.795544	-0.733316	-5.152332
H	0.360327	0.526326	-4.596777
H	-1.759623	1.594101	-1.189487

[1]S-7

Lowest frequency = 15.3194 cm^{-1}

Charge = 0, Multiplicity = 1

95

P	-0.968584	-0.809995	1.187980
P	0.475230	1.755785	0.718915
C	-0.993245	0.385924	2.624656
H	-1.906740	0.994945	2.518136
H	-1.035151	-0.157285	3.583602
C	0.262876	1.257204	2.511709
H	1.147433	0.660174	2.786474
H	0.231384	2.124000	3.190757
C	-2.722391	1.181078	-0.476812
C	-3.403367	0.229182	-1.258923
C	-3.385050	1.902090	0.529561
C	-4.758220	-0.021593	-1.005259
C	-4.737154	1.635606	0.782726
H	-2.855981	2.688313	1.077280
C	-5.417873	0.672169	0.018614
H	-5.267995	-0.774626	-1.613124
H	-5.267137	2.191541	1.562626
H	-6.473589	0.465868	0.220832
C	-2.674873	-0.566519	-2.303459
N	-1.304910	-0.556041	-2.127058
C	-0.468450	-1.377451	-3.045771
C	0.916185	-1.202151	-2.490745
C	2.200081	-1.548765	-2.876286
N	1.039770	-0.559477	-1.278635
N	2.303825	-0.495626	-0.904797
N	3.004762	-1.093468	-1.867081
H	2.594155	-2.059097	-3.752509

O	-3.285210	-1.188292	-3.179332
Fe	-0.520404	0.275099	-0.622091
C	-2.592220	-1.651842	1.206734
C	-2.847529	-2.619643	0.212579
C	-3.584542	-1.366410	2.162582
C	-4.070674	-3.295770	0.179547
H	-2.086219	-2.835427	-0.541137
C	-4.810902	-2.047396	2.125645
H	-3.413507	-0.618540	2.941351
C	-5.056097	-3.010025	1.137928
H	-4.258014	-4.039518	-0.601099
H	-5.576737	-1.820180	2.874109
H	-6.015522	-3.536381	1.111869
C	0.208611	-2.104632	1.752693
C	-0.245967	-3.346699	2.242962
C	1.590747	-1.820497	1.770624
C	0.668253	-4.282518	2.745948
H	-1.314275	-3.580735	2.238564
C	2.497034	-2.755277	2.287252
H	1.965661	-0.881763	1.354959
C	2.038894	-3.988438	2.774248
H	0.304205	-5.243708	3.122822
H	3.566075	-2.520287	2.304213
H	2.748844	-4.719867	3.173594
C	4.476092	-1.105436	-1.779517
H	4.829975	-1.796788	-2.560616
H	4.828126	-0.089014	-2.028403
C	4.958829	-1.517446	-0.388465
C	6.484684	-1.432234	-0.252357
H	4.608746	-2.543650	-0.172567
H	4.484281	-0.848479	0.349860
H	6.965262	-2.098656	-0.993949
H	6.812399	-0.404097	-0.500086
C	2.272319	2.097303	0.511010
C	2.688992	2.623903	-0.728883
C	3.241528	1.776415	1.478025
C	4.048376	2.813458	-0.999804
H	1.945704	2.881944	-1.491123
C	4.604645	1.966310	1.204425
H	2.951159	1.375380	2.453088
C	5.012289	2.478114	-0.034765
H	4.357453	3.229160	-1.964541
H	5.349000	1.717244	1.967937
H	6.076323	2.628703	-0.244044
C	-0.262163	3.443185	0.577924
C	-0.442605	4.306379	1.675894
C	-0.656233	3.879058	-0.705099
C	-1.019423	5.571167	1.494311
H	-0.130816	4.004225	2.680169
C	-1.223668	5.146408	-0.886599
H	-0.521612	3.218880	-1.571377
C	-1.412672	5.992487	0.215536
H	-1.158377	6.231192	2.356591
H	-1.525107	5.469271	-1.888039
H	-1.864528	6.979908	0.078393
C	6.960417	-1.797617	1.158130
H	6.667567	-2.829540	1.422968
H	8.057872	-1.727654	1.240874
H	6.520216	-1.118956	1.911495
C	-0.832612	-2.883435	-2.999562
H	-1.884049	-3.017240	-3.292281
H	-0.187245	-3.446251	-3.695091
H	-0.669696	-3.283475	-1.984681
C	-0.551614	-0.829782	-4.489280
H	0.139304	-1.382079	-5.149502
H	-1.578973	-0.950275	-4.863032
H	-0.281456	0.239142	-4.504094
H	-1.737883	1.607703	-0.820724

^{3}S-7

Lowest frequency = 12.3695 cm^{-1}

Charge = 0, Multiplicity = 3

95

P	1.657380	-0.518009	-1.196099
P	-0.732441	1.387106	-1.040024
C	1.595778	0.836305	-2.473080
H	2.149029	1.698144	-2.059007
H	2.081672	0.521302	-3.412469
C	0.117296	1.178060	-2.689527
H	-0.399790	0.341376	-3.191258
H	-0.027828	2.070553	-3.320441
C	2.303317	1.579729	1.157216
C	3.072265	0.524567	1.691890
C	2.921647	2.685831	0.557085
C	4.473216	0.616423	1.667680

C	4.323420	2.751144	0.511308
H	2.312156	3.504582	0.162279
C	5.095374	1.723974	1.079552
H	5.050035	-0.206851	2.098671
H	4.814114	3.616766	0.054134
H	6.187928	1.785040	1.053930
C	2.431980	-0.701558	2.279472
N	1.089061	-0.829360	1.981336
C	0.250428	-1.752721	2.795650
C	-1.155502	-1.286947	2.495432
C	-2.428772	-1.582462	2.957187
N	-1.311636	-0.272150	1.580590
N	-2.579575	0.078662	1.459832
N	-3.253713	-0.710578	2.297110
H	-2.804065	-2.302029	3.681782
O	3.080233	-1.498840	2.968293
Fe	0.271332	0.033307	0.519292
C	3.396533	-1.019218	-1.037181
C	3.676226	-2.171439	-0.272677
C	4.451528	-0.277251	-1.599981
C	5.000711	-2.570020	-0.068358
H	2.856602	-2.746169	0.169995
C	5.776077	-0.689073	-1.399189
H	4.252292	0.626536	-2.182155
C	6.051970	-1.828826	-0.630990
H	5.212495	-3.455668	0.538173
H	6.595000	-0.111477	-1.839639
H	7.088530	-2.141500	-0.469611
C	0.858732	-1.928966	-2.062167
C	1.442654	-2.532032	-3.193847
C	-0.375415	-2.405613	-1.580465
C	0.786539	-3.587025	-3.840177
H	2.415409	-2.184503	-3.559281
C	-1.032010	-3.461105	-2.229672
H	-0.820983	-1.941486	-0.689674
C	-0.450517	-4.050086	-3.361364
H	1.243128	-4.055568	-4.717875
H	-1.990899	-3.826653	-1.847945
H	-0.956363	-4.877878	-3.868534
C	-4.722737	-0.630237	2.311928
H	-5.060521	-1.026333	3.282712
H	-4.974309	0.440859	2.263494
C	-5.335556	-1.392348	1.130994
C	-6.856137	-1.204185	1.040954
H	-5.088691	-2.466978	1.224917
H	-4.858834	-1.026617	0.203969
H	-7.328797	-1.527997	1.988109
H	-7.077984	-0.124667	0.936207
C	-2.515044	1.273740	-1.455093
C	-3.422652	2.290695	-1.107259
C	-2.989779	0.102982	-2.081868
C	-4.787717	2.139526	-1.388561
H	-3.064492	3.201016	-0.617244
C	-4.352998	-0.038849	-2.368598
H	-2.297798	-0.703450	-2.348479
C	-5.255817	0.978093	-2.019613
H	-5.486642	2.938645	-1.120891
H	-4.711215	-0.946273	-2.865811
H	-6.321469	0.866488	-2.244217
C	-0.453156	3.120971	-0.523153
C	0.007484	4.122731	-1.398588
C	-0.688152	3.438391	0.832776
C	0.241113	5.420330	-0.920809
H	0.190697	3.901378	-2.454057
C	-0.461890	4.737613	1.302349
H	-1.048139	2.660681	1.515795
C	0.009846	5.728734	0.427656
H	0.604766	6.192262	-1.606450
H	-0.645436	4.974987	2.354908
H	0.197247	6.741749	0.797329
C	-7.472505	-1.973804	-0.132617
H	-7.296852	-3.059922	-0.033906
H	-8.562178	-1.812126	-0.185036
H	-7.034420	-1.648553	-1.093242
C	0.470361	-3.214632	2.342305
H	1.517454	-3.487020	2.546631
H	-0.195988	-3.904046	2.889269
H	0.273703	-3.312051	1.261335
C	0.496978	-1.600115	4.316410
H	-0.228745	-2.217972	4.873000
H	1.516357	-1.923750	4.566818
H	0.368141	-0.546860	4.616741
H	1.205446	1.616331	1.330781

^{5}**S-7**

Lowest frequency = 8.4976 cm^{-1}

Charge = 0, Multiplicity = 5

95

P	-1.016822	-0.902270	1.366631
P	-0.124501	2.171128	0.607511
C	-1.583565	0.545797	2.394304
H	-2.527667	0.911560	1.952488
H	-1.779836	0.226321	3.432578
C	-0.495394	1.630563	2.356063
H	0.457485	1.235673	2.753281
H	-0.757044	2.505200	2.975494
C	-2.882820	0.557470	-1.196470
C	-3.029802	-0.729118	-1.749639
C	-3.908449	1.127686	-0.424953
C	-4.234552	-1.426777	-1.547999
C	-5.087656	0.405951	-0.197556
H	-3.798051	2.144526	-0.037335
C	-5.250320	-0.869393	-0.765543
H	-4.343133	-2.410684	-2.011990
H	-5.891608	0.846601	0.401130
H	-6.176903	-1.427306	-0.598696
C	-1.952978	-1.393961	-2.564178
N	-0.669054	-1.002320	-2.251508
C	0.447240	-1.626993	-3.014794
C	1.728836	-1.068891	-2.430677
C	3.060771	-1.146707	-2.814788
N	1.720079	-0.401805	-1.233633
N	2.941165	-0.068148	-0.859931
N	3.753021	-0.514675	-1.819153
H	3.554392	-1.577816	-3.683029
O	-2.255953	-2.256766	-3.399496
Fe	-0.199360	0.030443	-0.668863
C	-2.400595	-2.082892	1.309547
C	-2.239756	-3.212860	0.479659
C	-3.593334	-1.916919	2.037297
C	-3.253838	-4.171703	0.393511
H	-1.321920	-3.332186	-0.105669
C	-4.607573	-2.881406	1.944690
H	-3.741846	-1.041054	2.675276
C	-4.439361	-4.007338	1.127569
H	-3.123512	-5.042962	-0.255870
H	-5.533272	-2.748836	2.513812
H	-5.234557	-4.756351	1.057723
C	0.239385	-1.695007	2.443933
C	-0.129027	-2.461081	3.568161
C	1.602406	-1.501291	2.146453
C	0.860644	-3.013792	4.390505
H	-1.187894	-2.634013	3.790434
C	2.589022	-2.050812	2.977644
H	1.888212	-0.933903	1.255079
C	2.218989	-2.806317	4.099130
H	0.572604	-3.612792	5.260428
H	3.647321	-1.896231	2.741656
H	2.988069	-3.242376	4.744740
C	5.207260	-0.356867	-1.660483
H	5.650481	-0.447044	-2.664764
H	5.373769	0.670110	-1.298134
C	5.789639	-1.390586	-0.689105
C	7.301224	-1.210879	-0.488225
H	5.574965	-2.406778	-1.069856
H	5.263411	-1.293762	0.278858
H	7.813443	-1.298526	-1.465610
H	7.500141	-0.183476	-0.126709
C	1.434488	3.118236	0.772385
C	1.509567	4.491734	0.469491
C	2.593329	2.431855	1.191679
C	2.730555	5.169366	0.593477
H	0.615814	5.032551	0.143617
C	3.806391	3.117810	1.321520
H	2.554667	1.360337	1.408823
C	3.878402	4.486742	1.020658
H	2.781213	6.237847	0.360848
H	4.699376	2.579560	1.656241
H	4.828388	5.021423	1.120582
C	-1.392549	3.410063	0.164853
C	-2.222964	4.039284	1.112769
C	-1.538831	3.727514	-1.202657
C	-3.191630	4.962916	0.695309
H	-2.124012	3.813465	2.178545
C	-2.502339	4.655850	-1.614444
H	-0.895348	3.242671	-1.946678
C	-3.334600	5.269725	-0.665955
H	-3.836040	5.444524	1.437563
H	-2.610037	4.893164	-2.677328
H	-4.095575	5.987302	-0.988253
C	7.889161	-2.228502	0.495765
H	7.733474	-3.262655	0.140311
H	8.973752	-2.074836	0.622619

H	7.416389	-2.139294	1.490455
C	0.482002	-3.164736	-2.796487
H	-0.455576	-3.604647	-3.164507
H	1.334662	-3.606765	-3.340011
H	0.600922	-3.385594	-1.721794
C	0.359192	-1.274816	-4.516807
H	1.224576	-1.690643	-5.061991
H	-0.563907	-1.701534	-4.934373
H	0.348557	-0.180038	-4.648724
H	-2.003428	1.164142	-1.461057

4-MeOC_6H_5

Lowest frequency = 99.2291 cm^{-1}

Charge = 0, Multiplicity = 1

16

C	-6.624290	2.553149	-0.018983
C	-5.215359	2.492675	-0.037462
C	-4.465301	3.670263	-0.006336
C	-5.101373	4.923267	0.043510
C	-6.499819	4.978022	0.061673
C	-7.269921	3.803551	0.030886
H	-4.738271	1.508839	-0.076340
H	-3.371557	3.610461	-0.021116
H	-4.510279	5.844121	0.067797
H	-7.010488	5.946538	0.100423
H	-8.360533	3.873459	0.045972
O	-7.272761	1.351080	-0.052123
C	-8.692553	1.352658	-0.035512
H	-9.109207	1.881664	-0.914836
H	-8.998658	0.296493	-0.067704
H	-9.088193	1.820652	0.887040

Zn(4-MeOC_6H_4)$_2$

Lowest frequency = 15.6854 cm^{-1}

Charge = 0, Multiplicity = 1

31

C	-6.622542	2.556349	-0.040374
C	-5.214626	2.491456	-0.058095
C	-4.461175	3.666400	-0.016495
C	-5.062971	4.949392	0.044089
C	-6.474468	4.977915	0.060178
C	-7.256257	3.811491	0.019215
H	-4.739991	1.506128	-0.104795
H	-3.367845	3.570911	-0.032209
H	-7.006301	5.937254	0.106218
H	-8.346725	3.892010	0.034062
O	-7.278457	1.359212	-0.083802
C	-8.698477	1.370064	-0.068268
H	-9.110718	1.909174	-0.943400
H	-9.011481	0.316197	-0.109352
H	-9.091576	1.833276	0.857676
Zn	-4.017575	6.575459	0.104306
C	-2.980692	8.206543	0.175063
C	-2.575373	8.771214	1.403988
C	-2.586755	8.902533	-0.996414
C	-1.823762	9.955453	1.482322
H	-2.845571	8.284711	2.350349
C	-1.839736	10.081267	-0.946791
H	-2.867813	8.519178	-1.985744
C	-1.451835	10.617759	0.297592
H	-1.536726	10.348469	2.461572
H	-1.540856	10.610529	-1.857218
O	-0.723998	11.772520	0.251810
C	-0.307339	12.353428	1.478903
H	0.350305	11.669528	2.050065
H	0.255229	13.260119	1.211027
H	-1.172588	12.632423	2.111377

Zn(4-MeOC_6H_4)Br

Lowest frequency = 34.6898 cm^{-1}

Charge = 0, Multiplicity = 1

17

C	-6.615547	2.565472	-0.018664
C	-5.206753	2.507267	-0.037052
C	-4.457536	3.684300	-0.005878
C	-5.074352	4.958860	0.044518
C	-6.484196	4.989728	0.061992
C	-7.256762	3.817884	0.031267
H	-4.726853	1.524496	-0.075961

H	-3.364423	3.597129	-0.021571
H	-7.018365	5.947010	0.100669
H	-8.347316	3.892712	0.046510
O	-7.263355	1.366983	-0.051746
C	-8.684996	1.366641	-0.035195
H	-9.100389	1.895043	-0.914895
H	-8.988363	0.309964	-0.067486
H	-9.079389	1.833929	0.887696
Zn	-4.042706	6.576952	0.087354
Br	-2.837887	8.485126	0.138006

MgBrCl

Lowest frequency = 106.3090 cm^{-1}

Charge = 0, Multiplicity = 1

3

Mg	-0.061943	0.370880	0.145325
Cl	2.143335	0.370880	0.145325
Br	-2.415584	0.370880	0.145325

$ZnCl_2$

Lowest frequency = 106.3090 cm^{-1}

Charge = 0, Multiplicity = 1

4

Zn	-0.061397	0.370880	0.145325
Cl	-2.165223	0.370880	0.145325
Cl	2.042429	0.370880	0.145325

$MgCl^+$

Lowest frequency = 522.0987 cm^{-1}

Charge = 1, Multiplicity = 1

2

Mg	-0.229802	0.370880	0.145325
Cl	-2.392993	0.370880	0.145325

$ZnCl^+$

Lowest frequency = 439.1911 cm^{-1}

Charge = 1, Multiplicity = 1

2

Zn	-0.271191	0.370880	0.145325
Cl	-2.351604	0.370880	0.145325

Substrate 1

Lowest frequency = 12.4839 cm^{-1}

Charge = 0, Multiplicity = 1

43

C	-4.713471	-1.049121	-1.361237
C	-3.395382	-0.560697	-1.291094
C	-5.648197	-0.713161	-0.375930
C	-3.032650	0.290131	-0.228215
C	-5.278083	0.126469	0.686488
H	-6.669839	-1.103736	-0.435551
C	-3.970757	0.630534	0.755458
H	-2.023537	0.712988	-0.169084
H	-6.009261	0.392962	1.457379
H	-3.681029	1.296824	1.574966
C	-2.455678	-0.969705	-2.399315
N	-1.133129	-0.736501	-2.163171
C	-0.045383	-1.023021	-3.109118
C	1.221423	-0.569375	-2.407144
C	2.542675	-0.558579	-2.835603
N	1.195410	-0.065414	-1.136133
N	2.408355	0.253749	-0.754248
N	3.235047	-0.035351	-1.780532
H	3.028886	-0.857476	-3.762076
O	-2.881288	-1.484155	-3.441558
C	4.679105	0.138785	-1.622225
H	5.089379	0.453836	-2.596624
H	4.808161	0.967910	-0.908688
C	5.369093	-1.133246	-1.113339
C	6.877806	-0.937453	-0.909681
H	5.189008	-1.956384	-1.830885
H	4.891124	-1.430408	-0.161839
H	7.338702	-0.620377	-1.865766

H	7.043011	-0.105153	-0.198398
C	7.577567	-2.200626	-0.395401
H	7.456108	-3.039617	-1.104244
H	8.658911	-2.029726	-0.256334
H	7.157454	-2.519608	0.575344
C	0.027607	-2.540471	-3.407762
H	-0.921360	-2.862985	-3.863526
H	0.858629	-2.755134	-4.102350
H	0.192495	-3.102862	-2.473658
C	-0.244920	-0.221261	-4.416940
H	0.584691	-0.420257	-5.117633
H	-1.194916	-0.522627	-4.884479
H	-0.273501	0.859025	-4.197980
H	-4.973021	-1.691079	-2.208578
H	-0.816128	-0.391016	-1.253374

Product 3

Lowest frequency = 13.7793 cm^{-1}

Charge = 0, Multiplicity = 1

57

C	-5.077694	0.737372	-0.570296
C	-3.762561	0.359195	-0.242516
C	-5.948835	-0.154425	-1.203127
C	-3.327272	-0.969460	-0.503687
C	-5.510110	-1.452150	-1.506426
H	-6.964888	0.160768	-1.463098
C	-4.217015	-1.852622	-1.150277
H	-6.181699	-2.160322	-2.003719
H	-3.888151	-2.879351	-1.344397
C	-2.896585	1.443589	0.367606
N	-1.587114	1.399889	-0.005143
C	-0.478217	2.208494	0.539650
C	0.772084	1.481843	0.080808
C	1.939471	1.142943	0.751984
N	0.855377	0.968249	-1.184717
N	1.993480	0.335982	-1.332902
N	2.663040	0.435664	-0.165120
H	2.297364	1.325667	1.762471
O	-3.378653	2.307119	1.109933
C	3.985252	-0.177367	-0.031464
H	4.234677	-0.171219	1.042336
H	3.886871	-1.228352	-0.348095
C	5.050406	0.556016	-0.853001
C	6.428933	-0.109869	-0.745835
H	5.108774	1.607720	-0.513768
H	4.717552	0.578918	-1.906360
H	6.740372	-0.142404	0.317027
H	6.348688	-1.164389	-1.074222
C	7.502050	0.606385	-1.573534
H	7.626402	1.653513	-1.243060
H	8.480617	0.104355	-1.481272
H	7.229819	0.624332	-2.644112
C	-0.536258	3.634793	-0.055958
H	-1.494160	4.102203	0.225307
H	0.296647	4.249830	0.326888
H	-0.463987	3.582720	-1.154547
C	-0.528319	2.254434	2.079054
H	0.311853	2.859171	2.462496
H	-1.477044	2.705270	2.404123
H	-0.455736	1.235352	2.494291
H	-5.388219	1.757461	-0.323248
H	-1.300072	0.715696	-0.707089
C	-1.985639	-1.465507	-0.080731
C	-1.071175	-1.996194	-1.015671
C	-1.577520	-1.373336	1.263440
C	0.216244	-2.377149	-0.631999
H	-1.359902	-2.066875	-2.069452
C	-0.294160	-1.760029	1.666190
H	-2.274827	-0.974334	2.007416
C	0.617949	-2.245528	0.709210
H	0.938703	-2.746809	-1.364177
H	-0.013782	-1.667575	2.718340
O	1.911269	-2.602722	0.992245
C	2.345461	-2.518974	2.341031
H	1.749125	-3.177065	3.001645
H	3.394075	-2.852052	2.345926
H	2.287575	-1.480614	2.723388

References

[1] Q. Gu, H. H. Al Mamari, K. Graczyk, E. Diers, L. Ackermann, *Angew. Chem. Int. Ed.* **2014**, *53*, 3868-3871.

[2] P. Gandeepan, P. Rajamalli, C. H. Cheng, *Chem. Eur. J.* **2015**, *21*, 9198-9203.

[3] a) A. Krasovskiy, P. Knochel, *Synthesis* **2006**, 890-891; b) K. Adams, A. K. Ball, J. Birkett, L. Brown, B. Chappell, D. M. Gill, P. T. Lo, N. J. Patmore, C. R. Rice, J. Ryan, *Nat. Chem.* **2017**, *9*, 396-401.

[4] C. Zhu, J. C. Oliveira, Z. Shen, H. Huang, L. Ackermann, *ACS Catal.* **2018**, *8*, 4402-4407.

[5] D. Santrač, S. Cella, W. Wang, L. Ackermann, *Eur. J. Org. Chem.* **2016**, 5429-5436.

[6] D. Schmiel, H. Butenschön, *Organometallics* **2017**, *36*, 4979-4989.

[7] S. Zhao, B. Liu, B.-B. Zhan, W.-D. Zhang, B.-F. Shi, *Org. Lett.* **2016**, *18*, 4586-4589.

[8] L. Su, D.-D. Guo, B. Li, S.-H. Guo, G.-F. Pan, Y.-R. Gao, Y.-Q. Wang, *ChemCatChem* **2017**, *9*, 2001-2008.

[9] P. Gandeepan, K. Parthasarathy, C.-H. Cheng, *J. Am. Chem. Soc.* **2010**, *132*, 8569-8571.

[10] N. Yoshikai, S. Asako, T. Yamakawa, L. Ilies, E. Nakamura, *Chem. Asian J.* **2011**, *6*, 3059-3065.

[11] J. Tao, J. P. Perdew, V. N. Staroverov, G. E. Scuseria, *Phys. Rev. Lett.* **2003**, *91*, 146401.

[12] a) S. Grimme, J. Antony, S. Ehrlich, H. Krieg, *J. Chem. Phys.* **2010**, *132*, 154104; b) S. Grimme, S. Ehrlich, L. Goerigk, *J. Comput. Chem.* **2011**, *32*, 1456-1465.

[13] a) A. Schäfer, H. Horn, R. Ahlrichs, *J. Chem. Phys.* **1992**, *97*, 2571-2577; b) A. Schäfer, C. Huber, R. Ahlrichs, *J. Chem. Phys.* **1994**, *100*, 5829-5835; c) F. Weigend, R. Ahlrichs, *Phys. Chem. Chem. Phys.* **2005**, *7*, 3297-3305; d) F. Weigend, *Phys. Chem. Chem. Phys.* **2006**, *8*, 1057-1065.

[14] Y. Zhao, D. G. Truhlar, *J. Phys. Chem. A* **2005**, *109*, 5656-5667.

[15] A. V. Marenich, C. J. Cramer, D. G. Truhlar, *J. Phys. Chem. B* **2009**, *113*, 6378-

6396.

[16] R. A. Gaussian09, *Inc., Wallingford CT* **2009**, *121*, 150-166.

[17] L. Schrodinger, *Schrodinger LLC, New York, NY* **2015**.

[18] H. G. Roth, N. A. Romero, D. A. Nicewicz, *Synlett* **2016**, *27*, 714-723.

[19] N. G. Connelly, W. E. Geiger, *Chem. Rev.* **1996**, *96*, 877-910.

^{1}H-, ^{13}C- and ^{19}F-NMR spectra

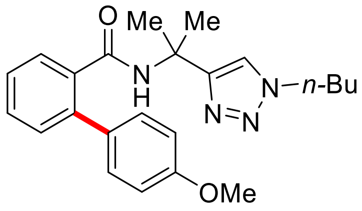

3

400 MHz, $CDCl_3$

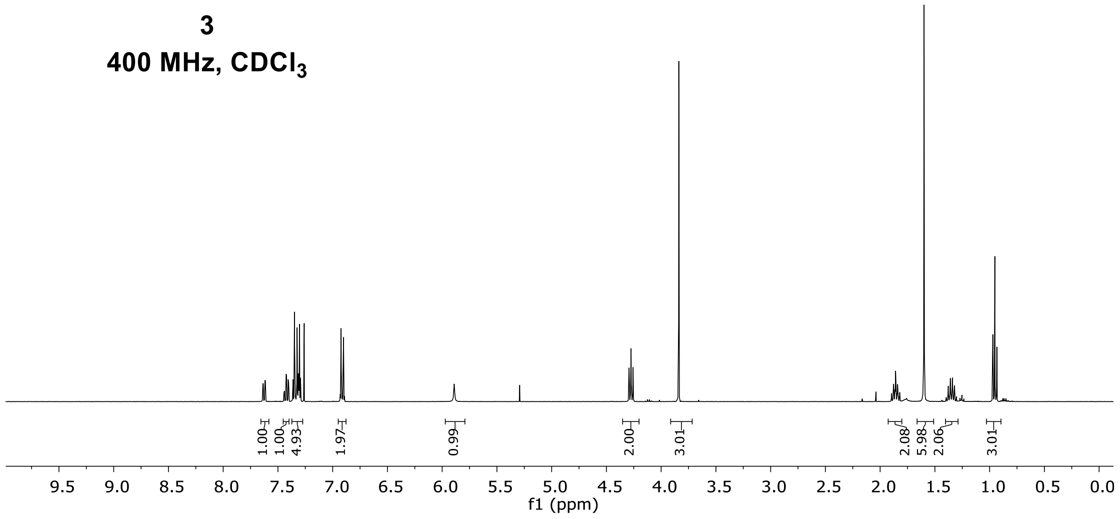

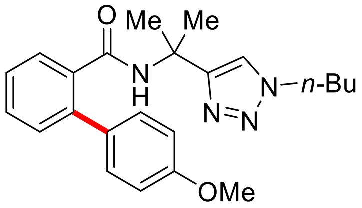

3

100 MHz, $CDCl_3$

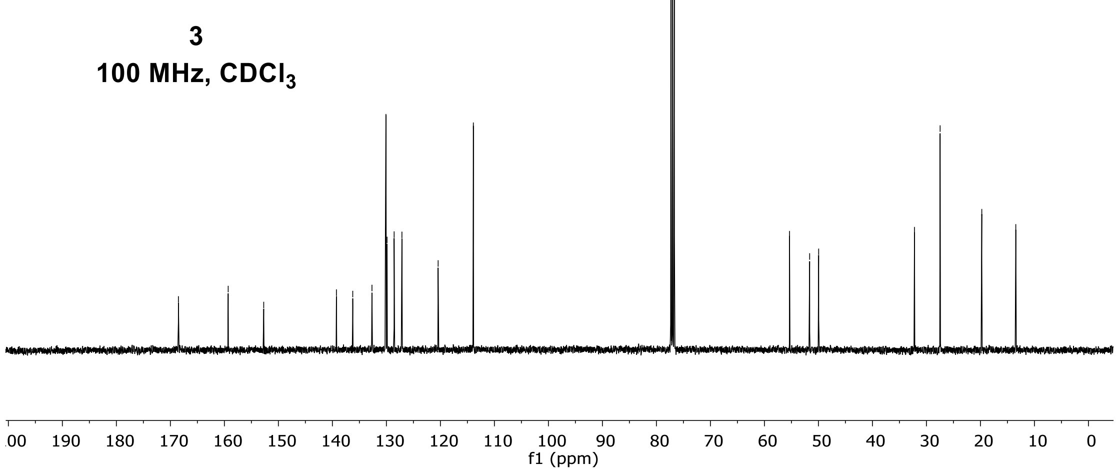

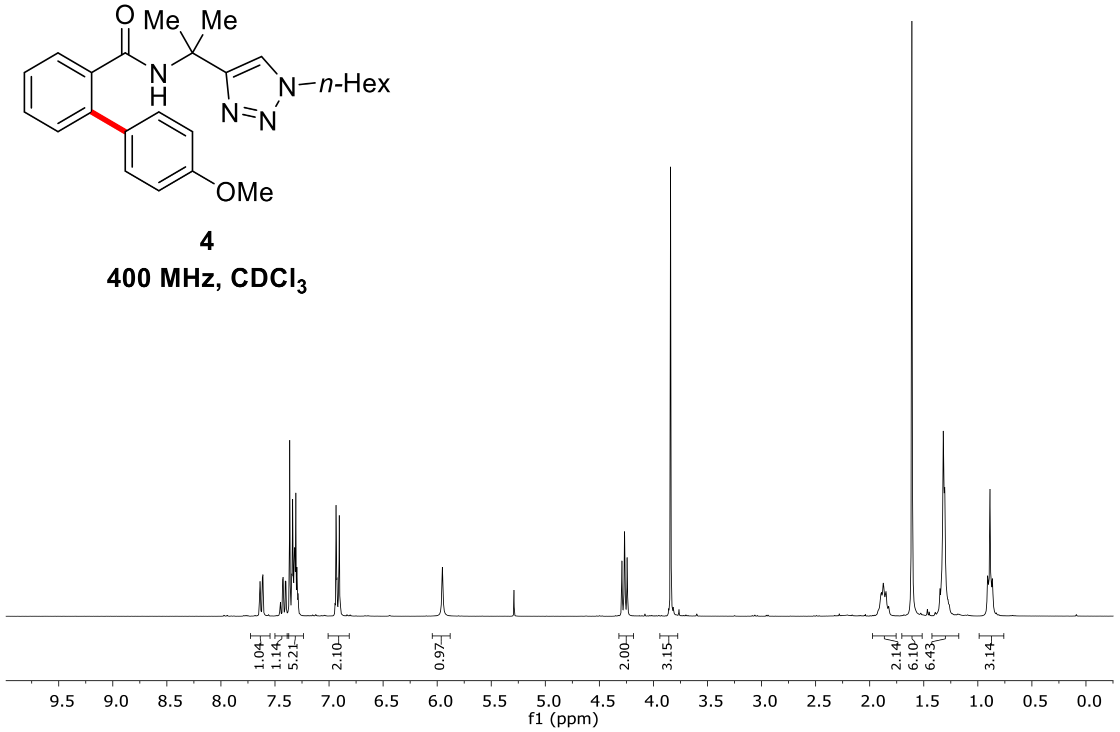

4
400 MHz, $CDCl_3$

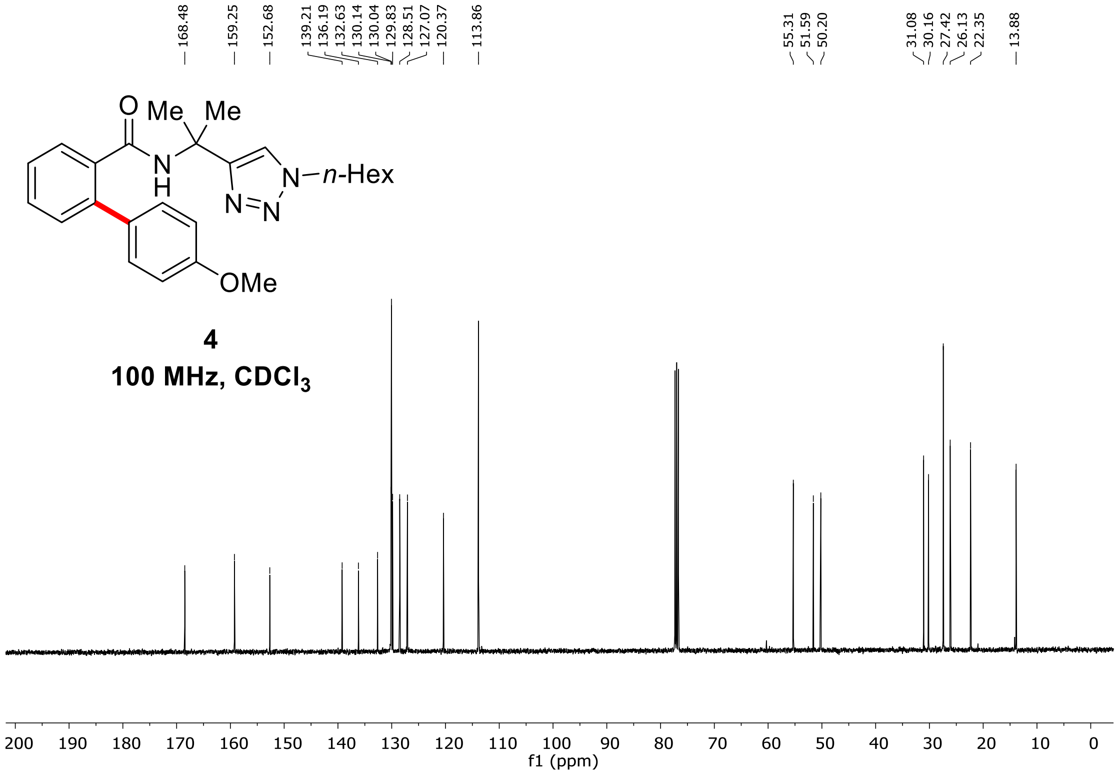

4
100 MHz, $CDCl_3$

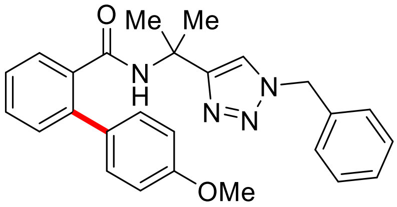

5

400 MHz, $CDCl_3$

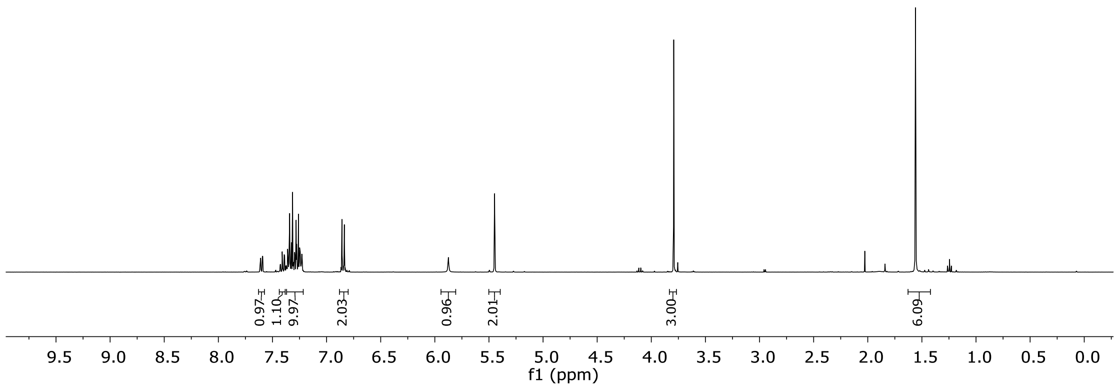

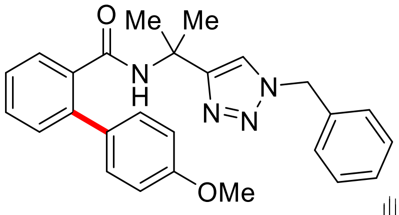

5

100 MHz, $CDCl_3$

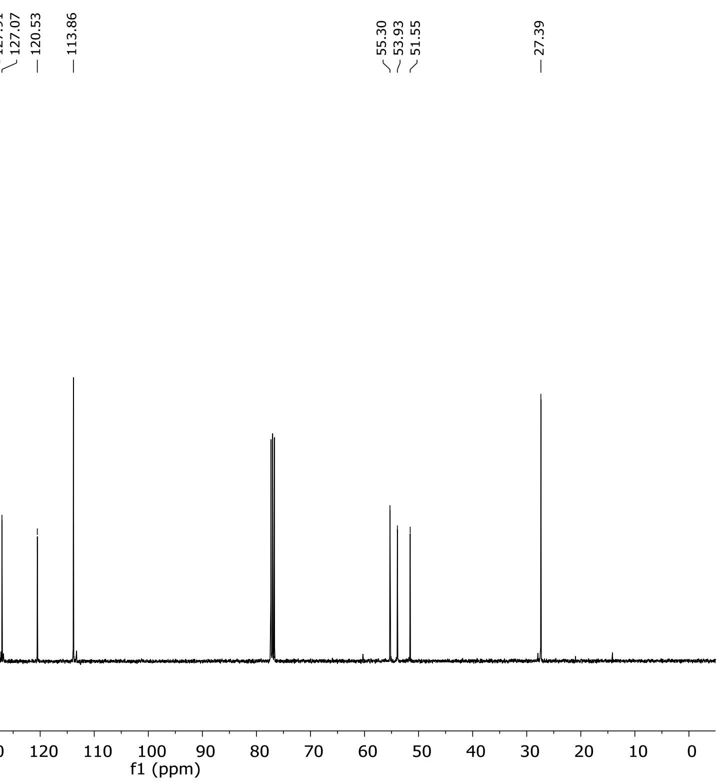

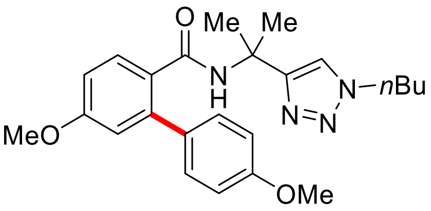

6

400 MHz, $CDCl_3$

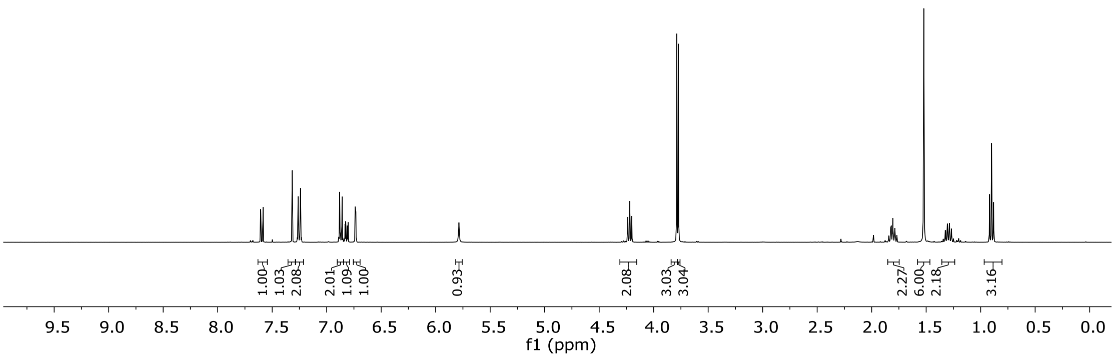

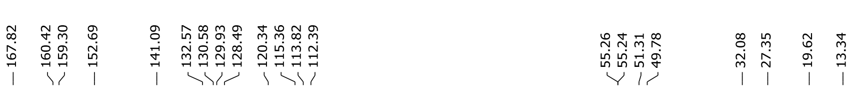

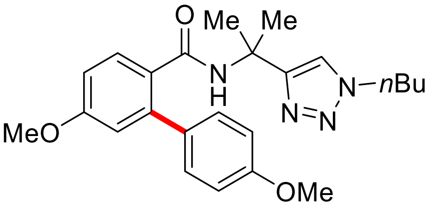

6

100 MHz, $CDCl_3$

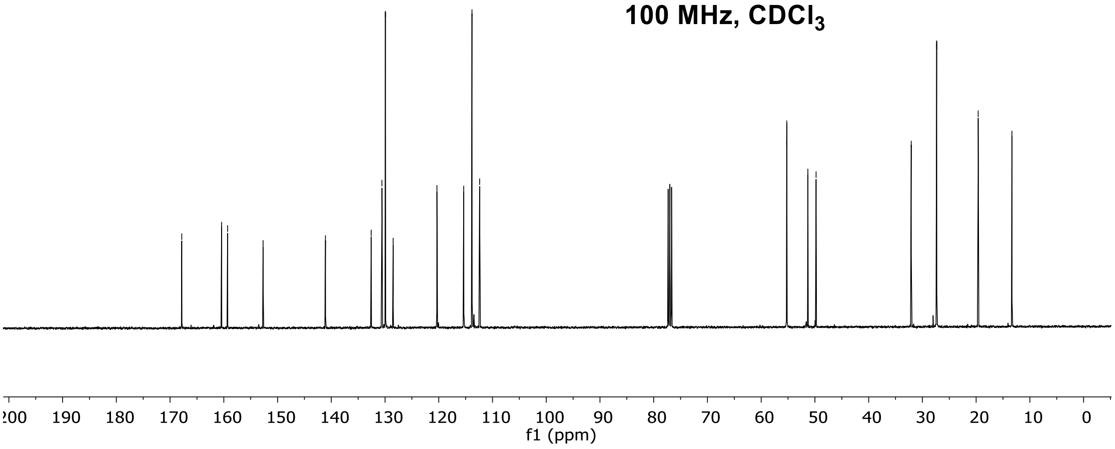

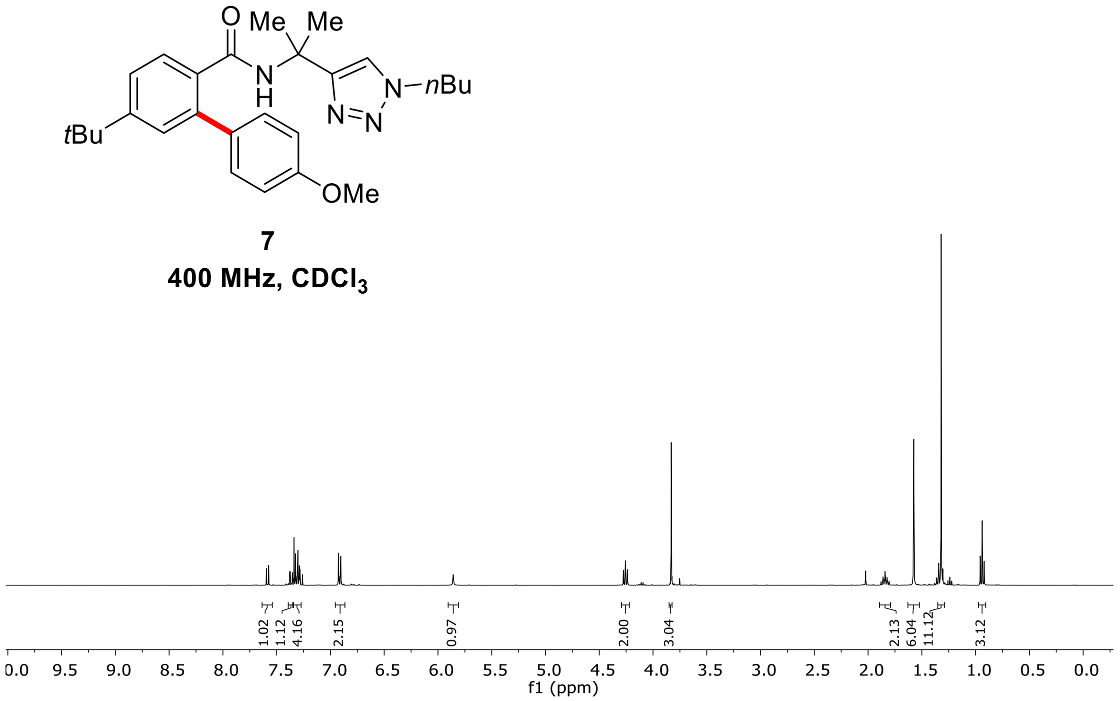
Me
Me
O
N
H
N−nBu
N=N
tBu
OMe
7
400 MHz, CDCl3
1.02
1.12
4.16
2.15
0.97
2.00
3.04
2.13
6.04
11.12
3.12
f1 (ppm)

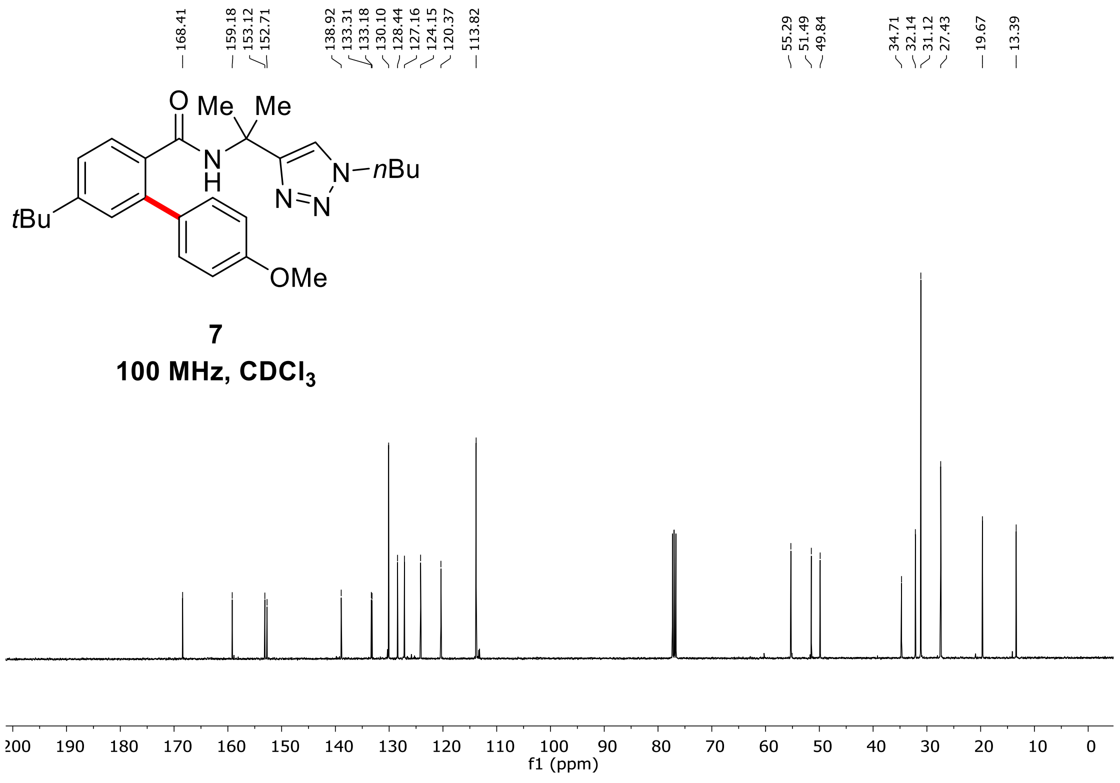
168.41
159.18
153.12
152.71
138.92
133.31
133.18
130.10
128.44
127.16
124.15
120.37
113.82
55.29
51.49
49.84
34.71
32.14
31.12
27.43
19.67
13.39
Me
Me
O
N
H
N−nBu
N=N
tBu
OMe
7
100 MHz, CDCl3
f1 (ppm)

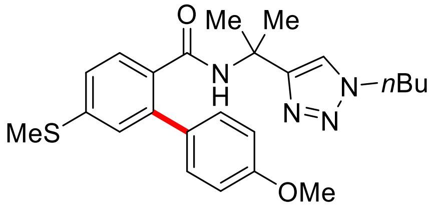

8

400 MHz, CDCl$_3$

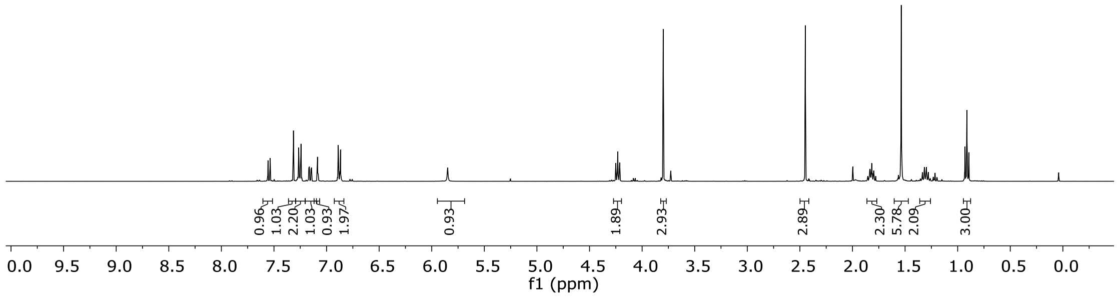

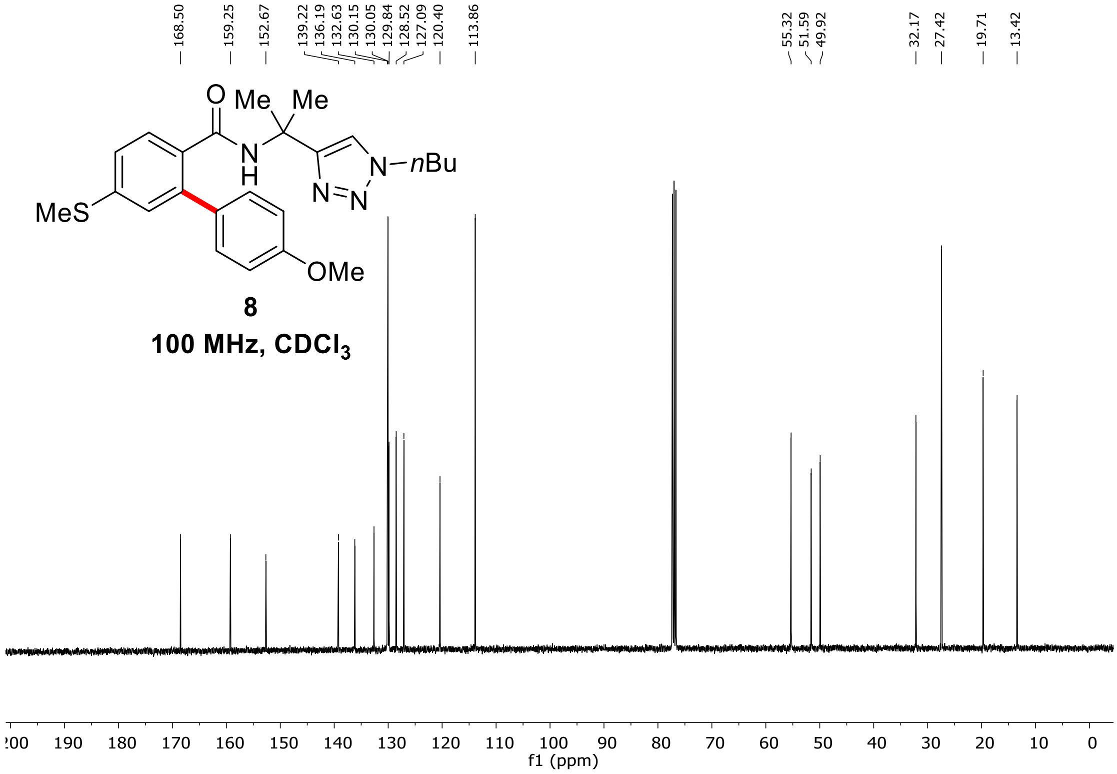

8

100 MHz, CDCl$_3$

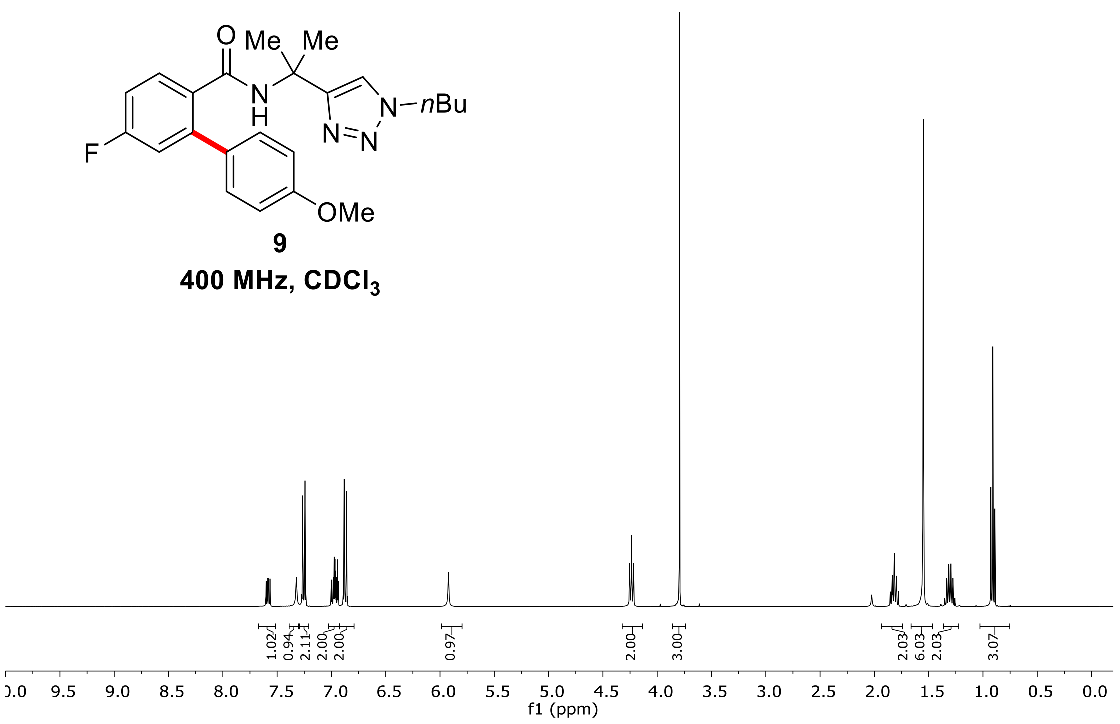

O Me Me
N H
N–nBu
N=N
F
OMe
9
400 MHz, CDCl3
1.02 0.94 2.11 2.00 2.00 0.97 2.00 3.00 2.03 6.03 2.03 3.07
10.0 9.5 9.0 8.5 8.0 7.5 7.0 6.5 6.0 5.5 5.0 4.5 4.0 3.5 3.0 2.5 2.0 1.5 1.0 0.5 0.0
f1 (ppm)

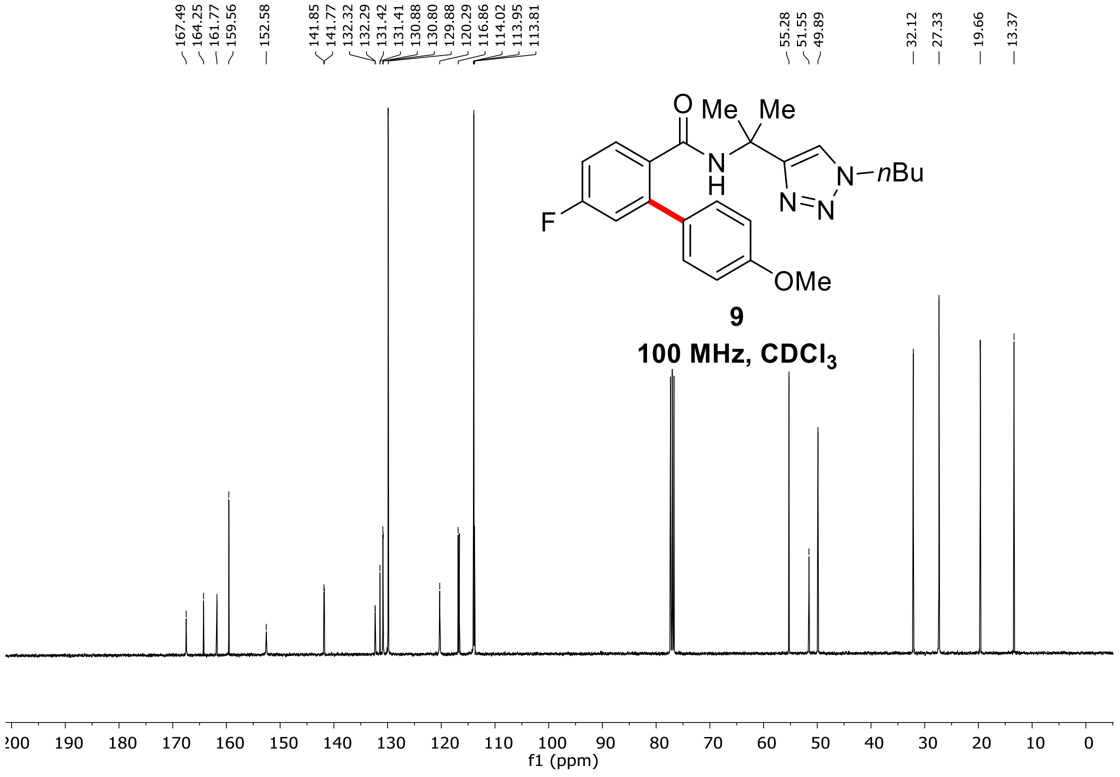

167.49 164.25 161.77 159.56 152.58 141.85 141.77 132.32 132.29 131.42 131.41 130.88 130.80 129.88 120.29 116.86 114.02 113.95 113.81
55.28 51.55 49.89 32.12 27.33 19.66 13.37
O Me Me
N H
N–nBu
N=N
F
OMe
9
100 MHz, CDCl3
200 190 180 170 160 150 140 130 120 110 100 90 80 70 60 50 40 30 20 10 0
f1 (ppm)

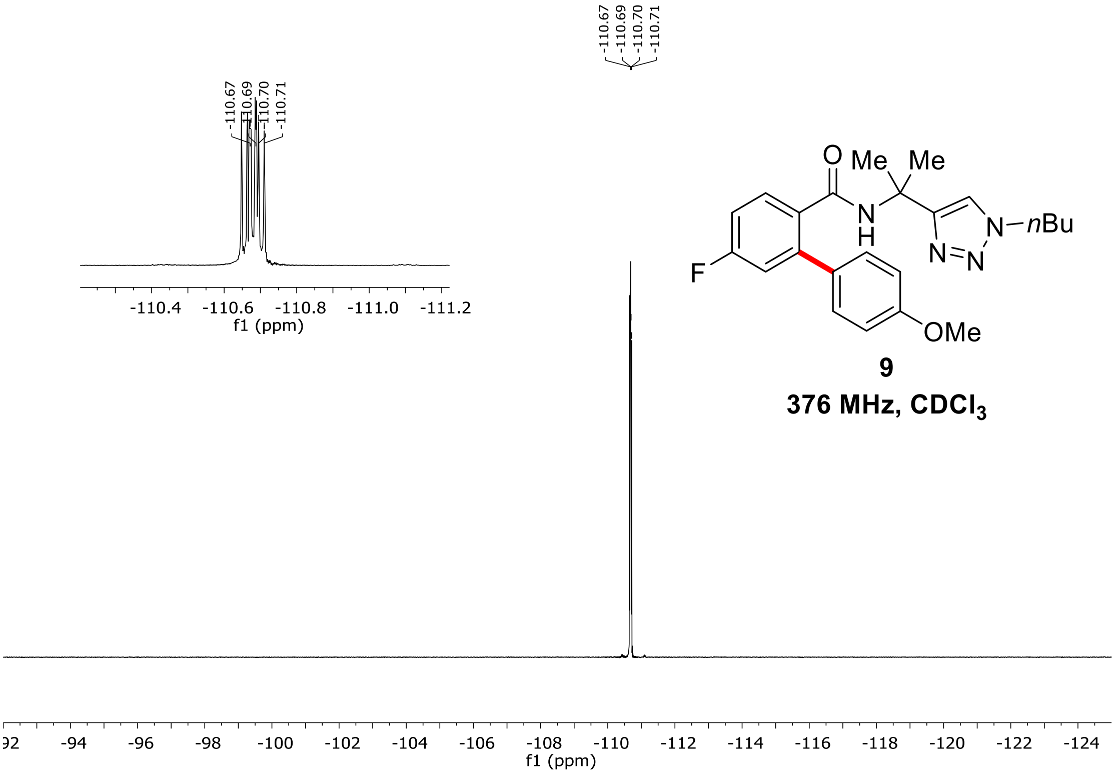

9

376 MHz, $CDCl_3$

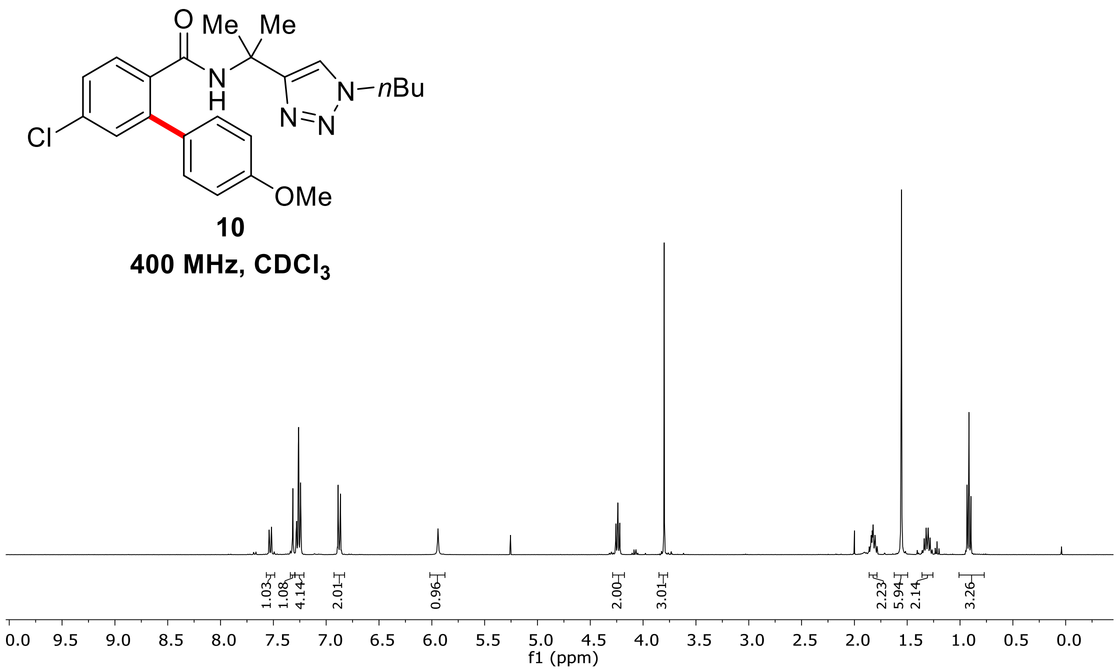
O
Me
Me
N
H
N−nBu
N=N
Cl
OMe
10
400 MHz, CDCl3
1.03
1.08
4.14
2.01
0.96
2.00
3.01
2.23
5.94
2.14
3.26
f1 (ppm)

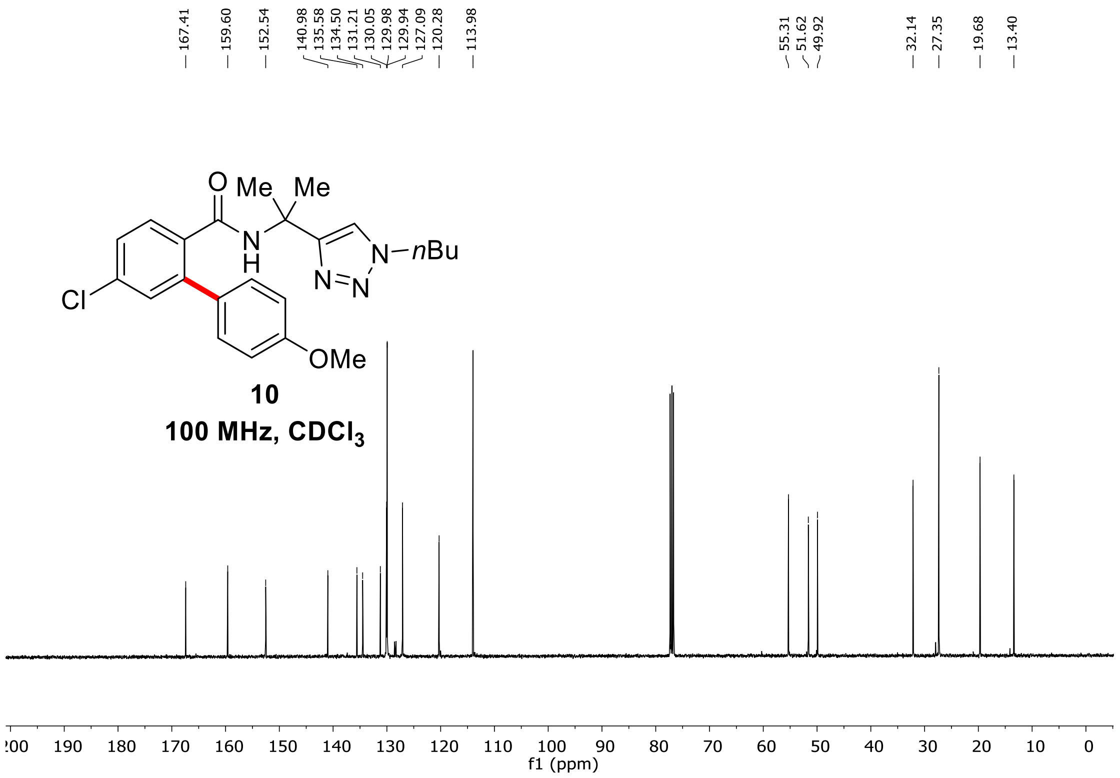
167.41
159.60
152.54
140.98
135.58
134.50
131.21
130.05
129.98
129.94
127.09
120.28
113.98
55.31
51.62
49.92
32.14
27.35
19.68
13.40
O
Me
Me
N
H
N−nBu
N=N
Cl
OMe
10
100 MHz, CDCl3
f1 (ppm)

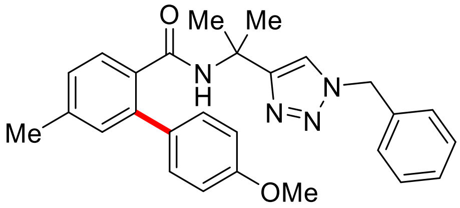

11

300 MHz, $CDCl_3$

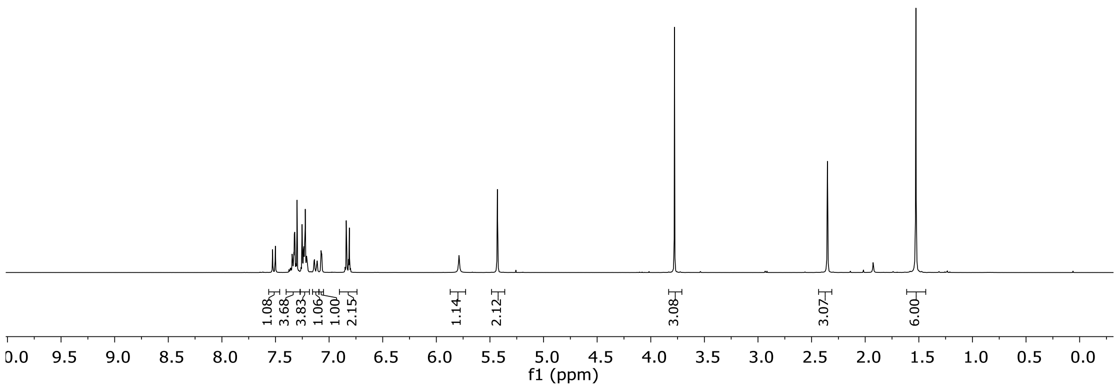

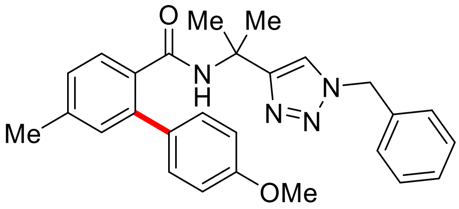

11

125 MHz, $CDCl_3$

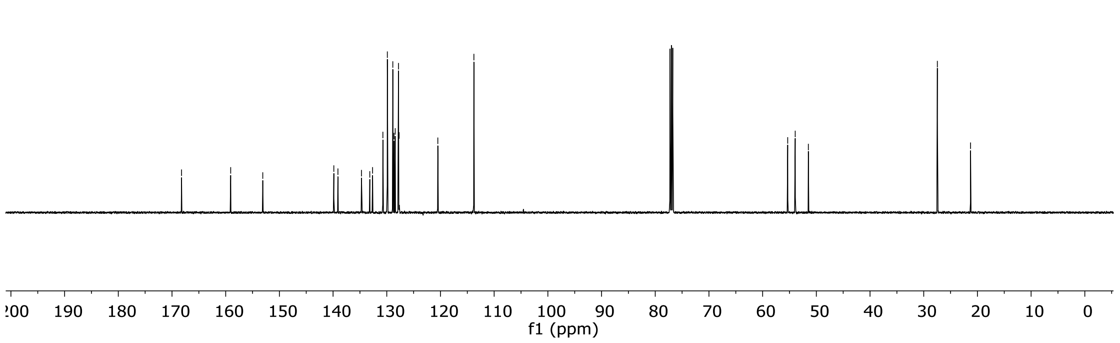

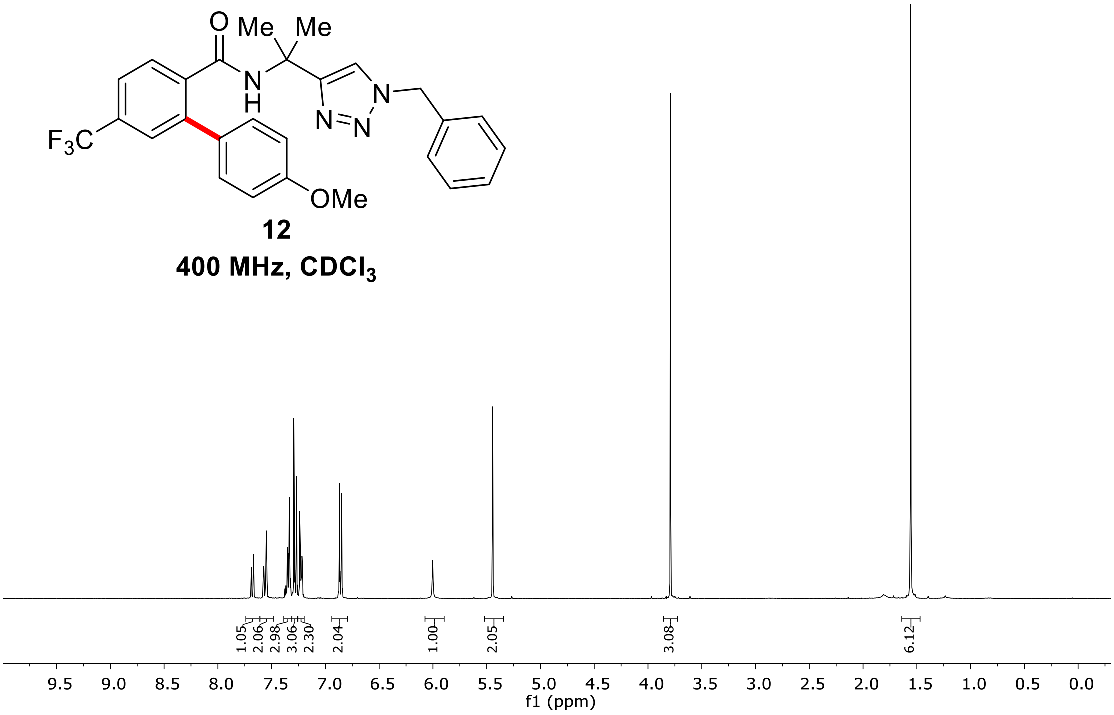
O
Me
Me
N
H
N
N
N
F3C
OMe
12
400 MHz, CDCl3
1.05
2.06
2.98
3.06
2.30
2.04
1.00
2.05
3.08
6.12
9.5 9.0 8.5 8.0 7.5 7.0 6.5 6.0 5.5 5.0 4.5 4.0 3.5 3.0 2.5 2.0 1.5 1.0 0.5 0.0
f1 (ppm)

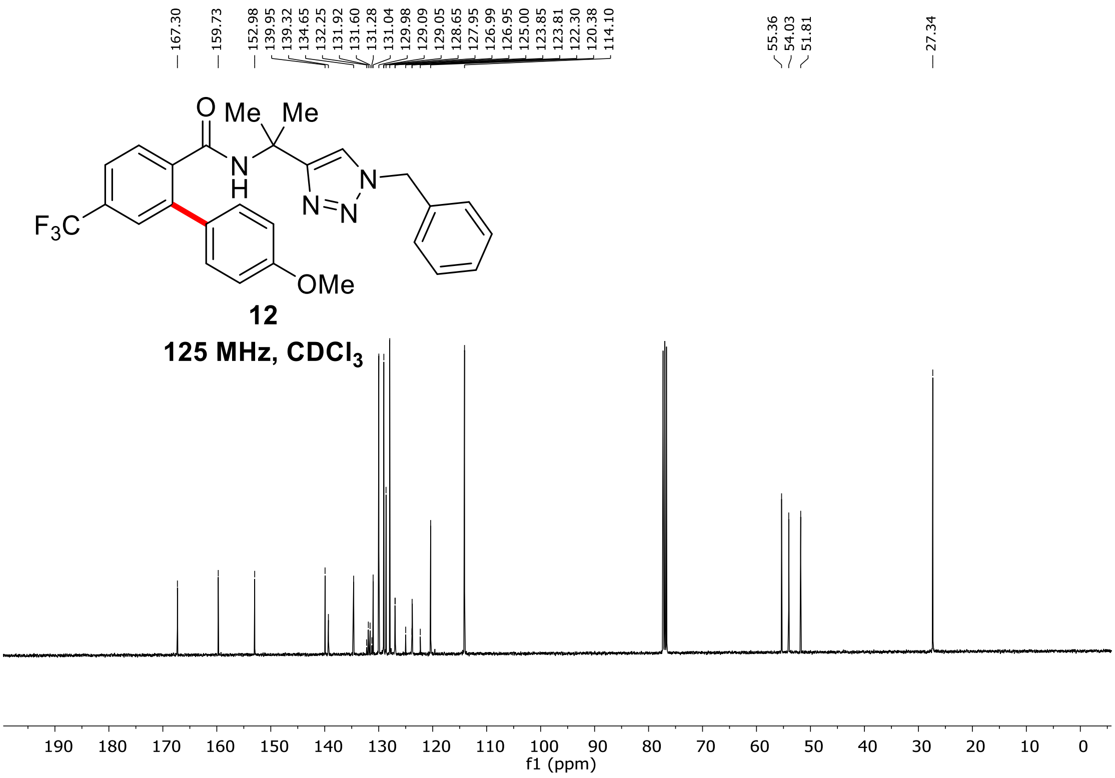
167.30
159.73
152.98
139.95
139.32
134.65
132.25
131.92
131.60
131.28
131.04
129.98
129.09
129.05
128.65
127.95
126.99
126.95
125.00
123.85
123.81
122.30
120.38
114.10
55.36
54.03
51.81
27.34
O
Me
Me
N
H
N
N
N
F3C
OMe
12
125 MHz, CDCl3
190 180 170 160 150 140 130 120 110 100 90 80 70 60 50 40 30 20 10 0
f1 (ppm)

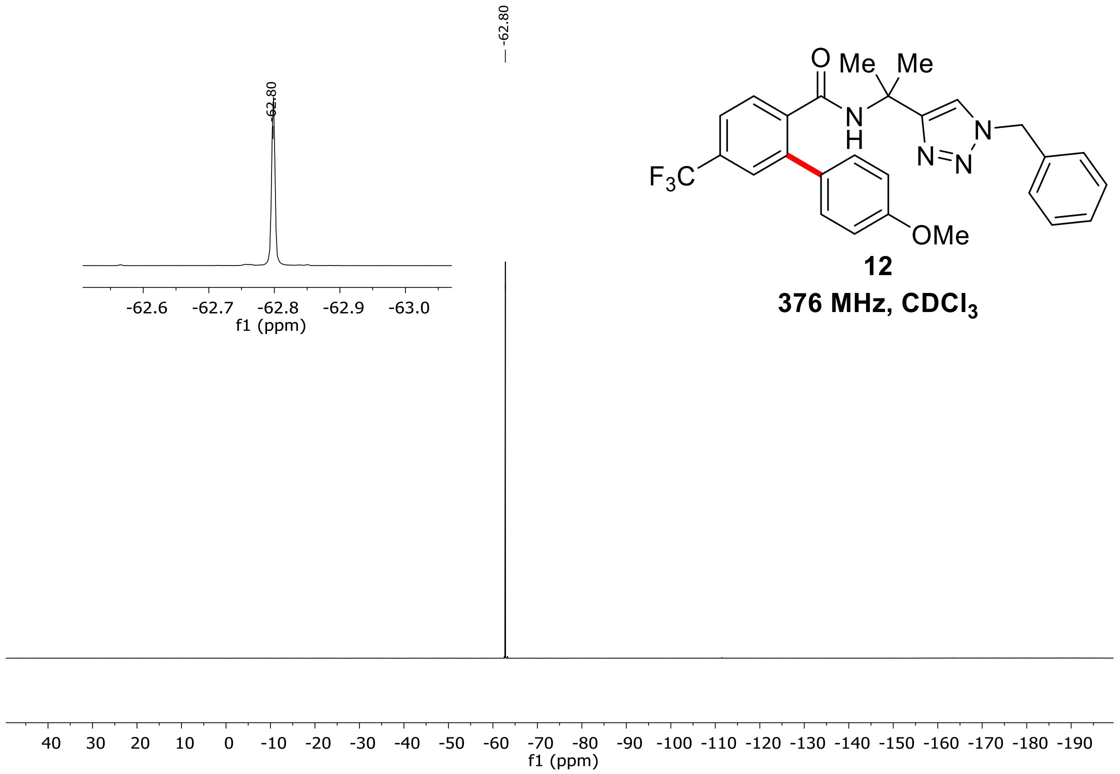

12

376 MHz, $CDCl_3$

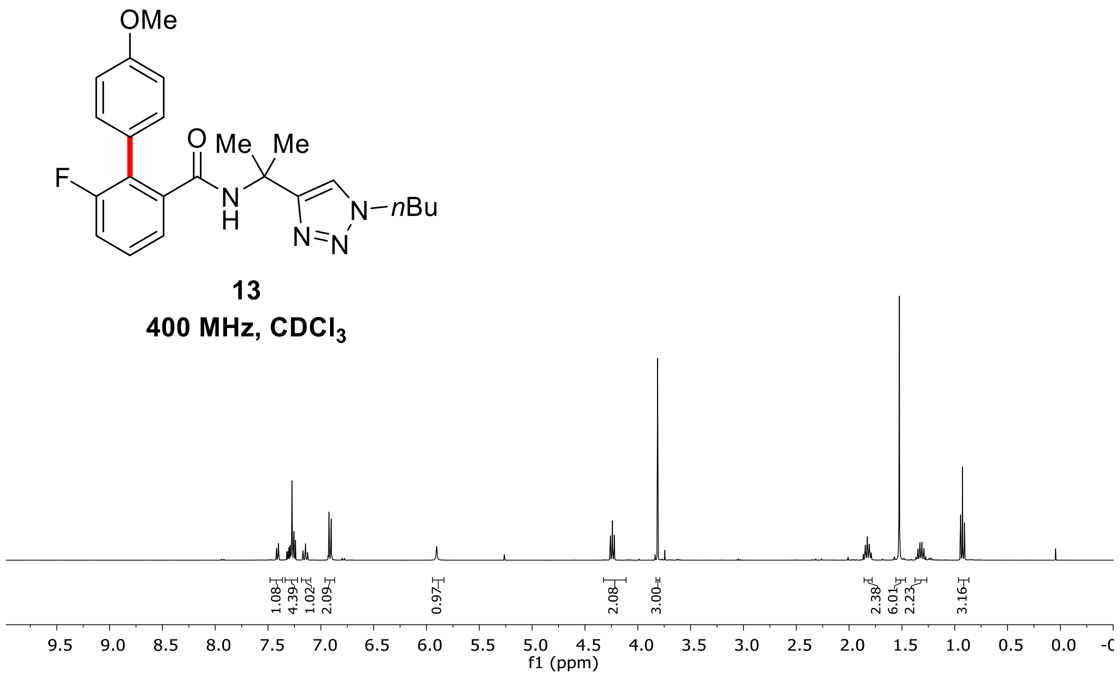
OMe
O
Me
Me
F
N
H
N
nBu
N
N
13
400 MHz, CDCl3
1.08
4.39
1.02
2.09
0.97
2.08
3.00
2.38
6.01
2.23
3.16
9.5 9.0 8.5 8.0 7.5 7.0 6.5 6.0 5.5 5.0 4.5 4.0 3.5 3.0 2.5 2.0 1.5 1.0 0.5 0.0
f1 (ppm)

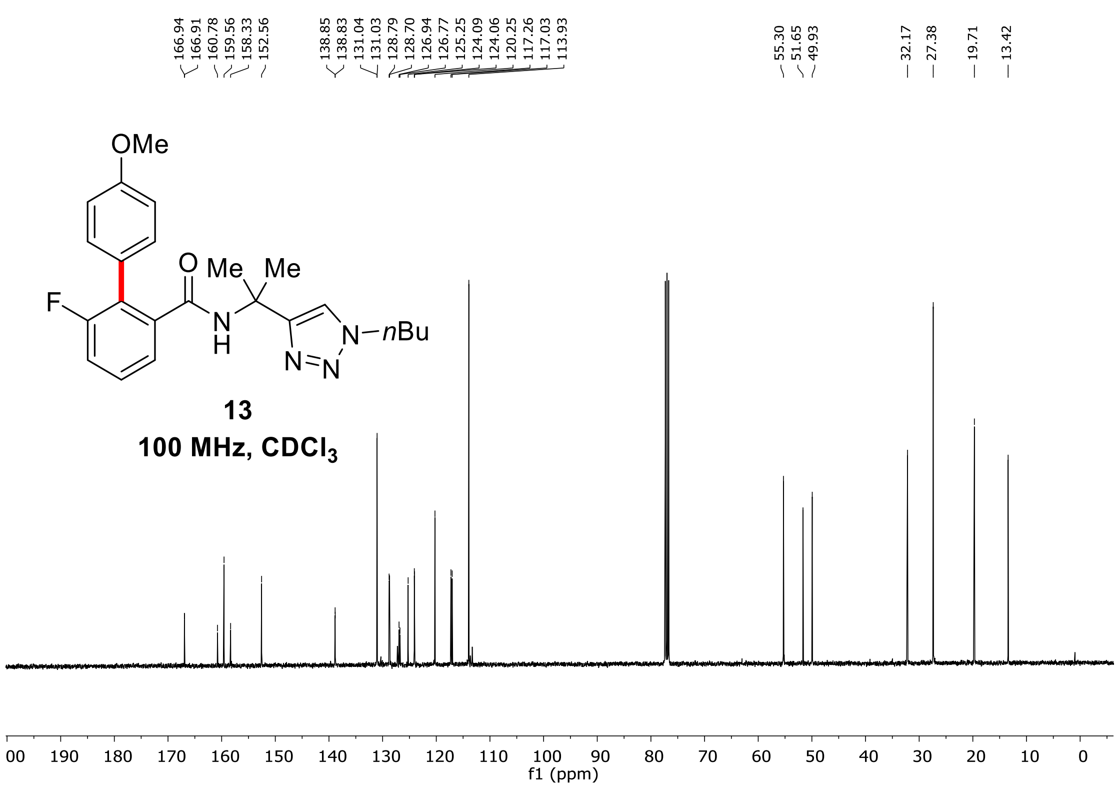
166.94
166.91
160.78
159.56
158.33
152.56
138.85
138.83
131.04
131.03
128.79
128.70
126.94
126.77
125.25
124.09
124.06
120.25
117.26
117.03
113.93
55.30
51.65
49.93
32.17
27.38
19.71
13.42
OMe
O
Me
Me
F
N
H
N
nBu
N
N
13
100 MHz, CDCl3
190 180 170 160 150 140 130 120 110 100 90 80 70 60 50 40 30 20 10 0
f1 (ppm)

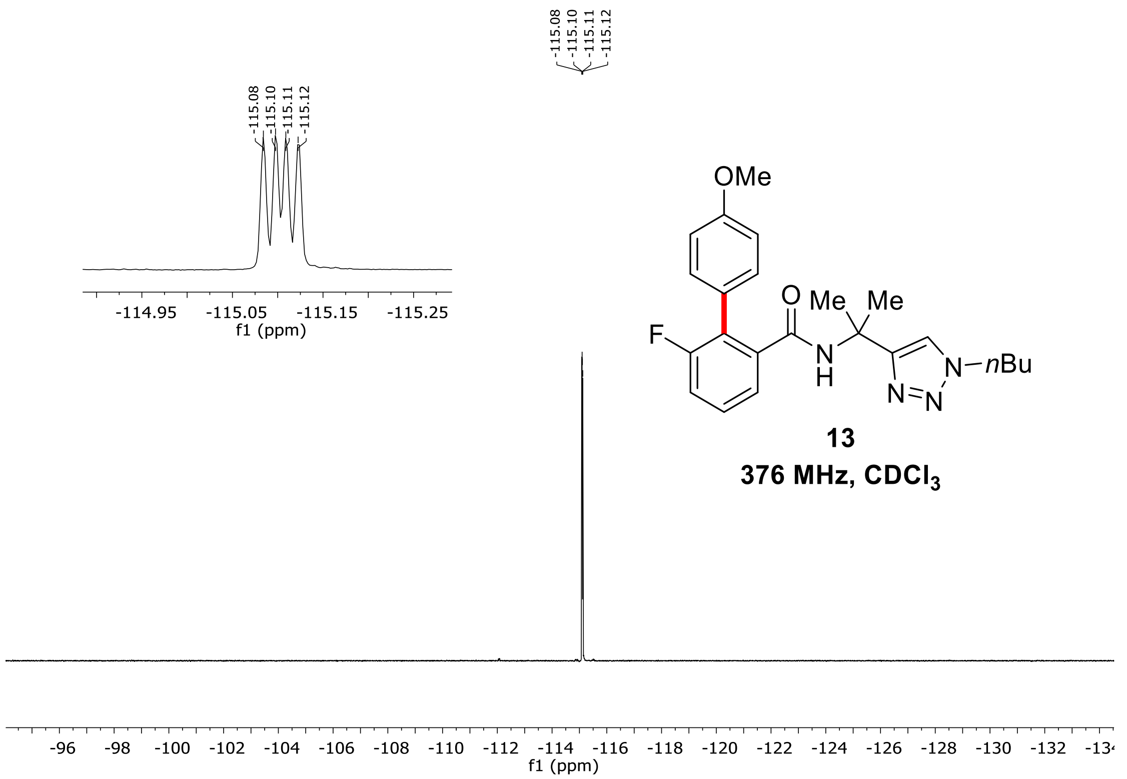
-115.08
-115.10
-115.11
-115.12
-114.95
-115.05
-115.15
-115.25
f1 (ppm)
OMe
F
O
Me
Me
N
H
N
nBu
N
N
13
376 MHz, CDCl3
-96
-98
-100
-102
-104
-106
-108
-110
-112
-114
-116
-118
-120
-122
-124
-126
-128
-130
-132
f1 (ppm)

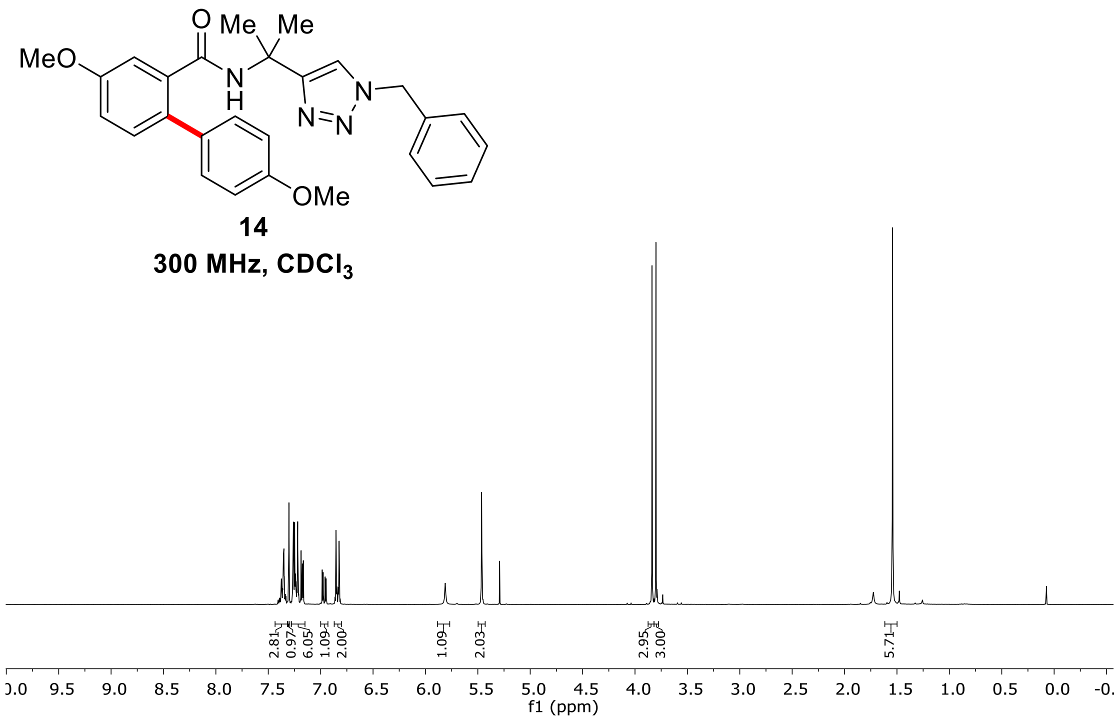
MeO
O
Me
Me
N
H
N
N
N
OMe
14
300 MHz, $CDCl_3$
2.81
0.97
6.05
1.09
2.00
1.09
2.03
2.95
3.00
5.71
f1 (ppm)

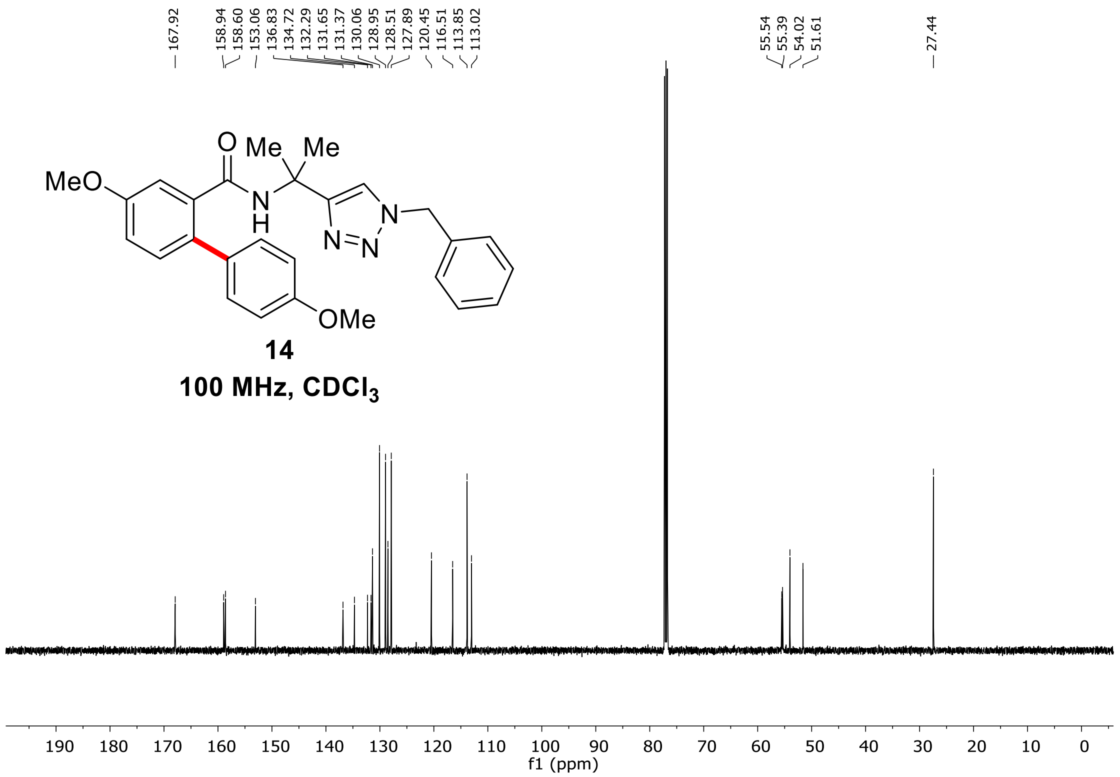
167.92
158.94
158.60
153.06
136.83
134.72
132.29
131.65
131.37
130.06
128.95
128.51
127.89
120.45
116.51
113.85
113.02
55.54
55.39
54.02
51.61
27.44
MeO
O
Me
Me
N
H
N
N
N
OMe
14
100 MHz, $CDCl_3$
f1 (ppm)

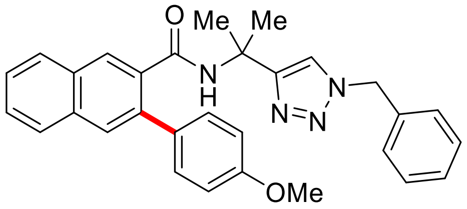

15

400 MHz, $CDCl_3$

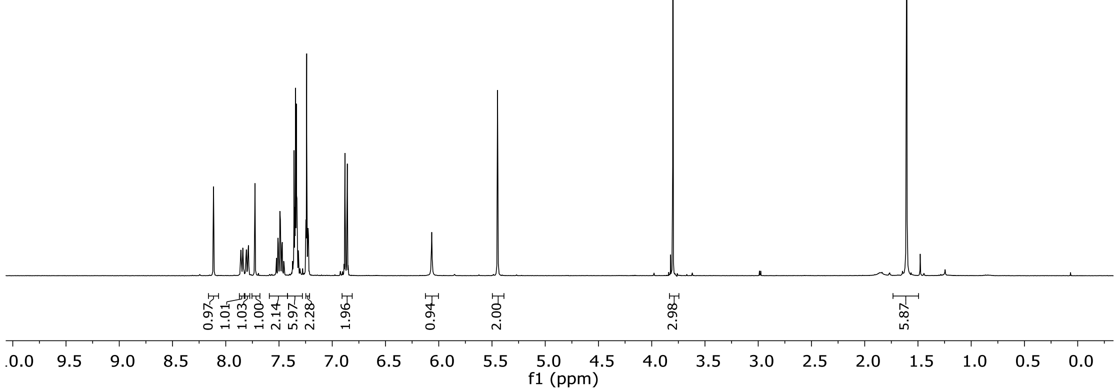

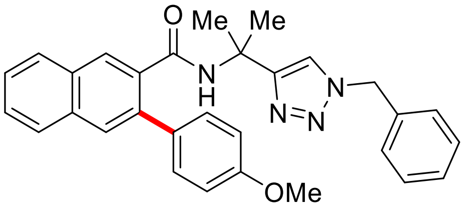

15

100 MHz, $CDCl_3$

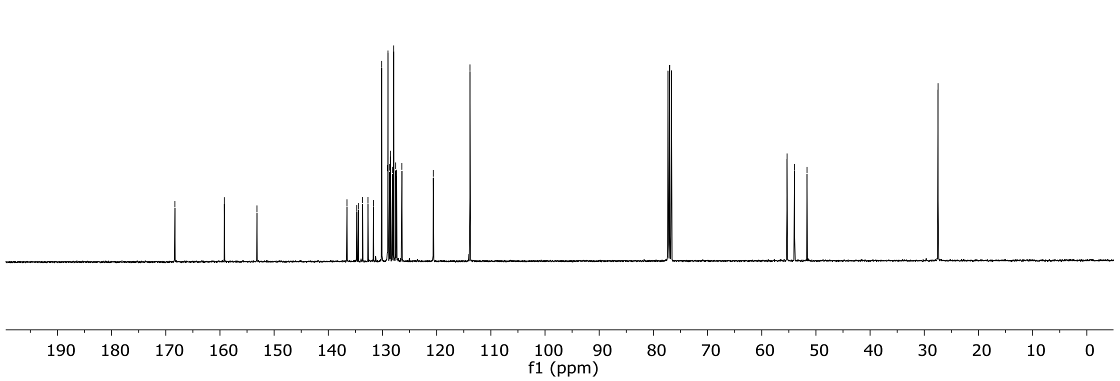

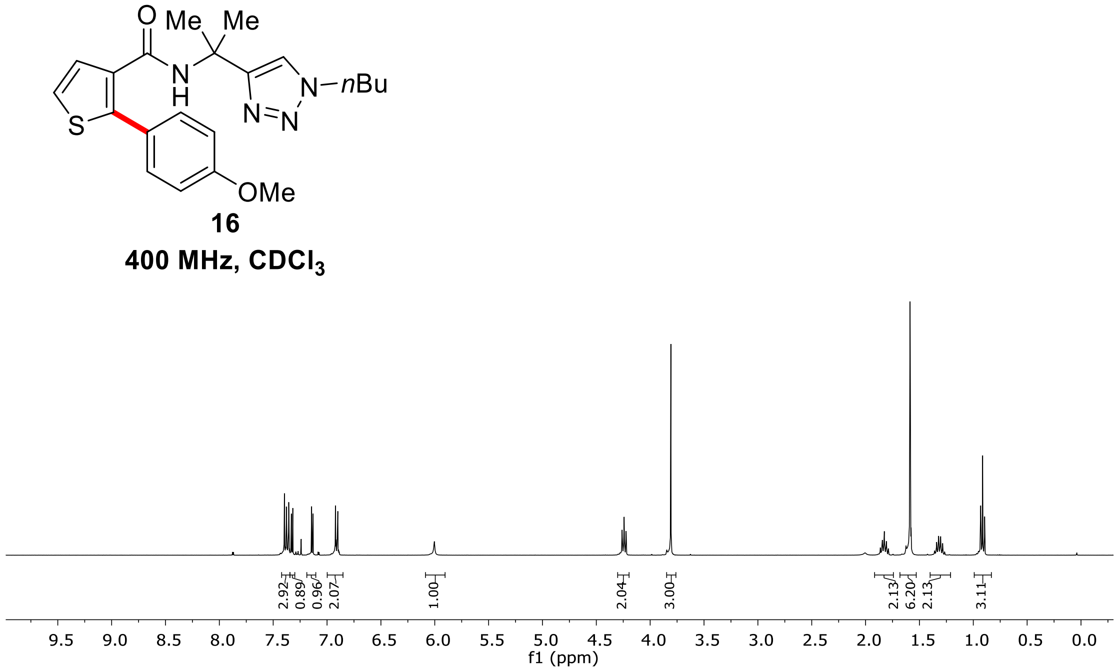
O
Me
Me
N
H
N−nBu
N=N
S
OMe
16
400 MHz, CDCl3
2.92
0.89
0.96
2.07
1.00
2.04
3.00
2.13
6.20
2.13
3.11
9.5 9.0 8.5 8.0 7.5 7.0 6.5 6.0 5.5 5.0 4.5 4.0 3.5 3.0 2.5 2.0 1.5 1.0 0.5 0.0
f1 (ppm)

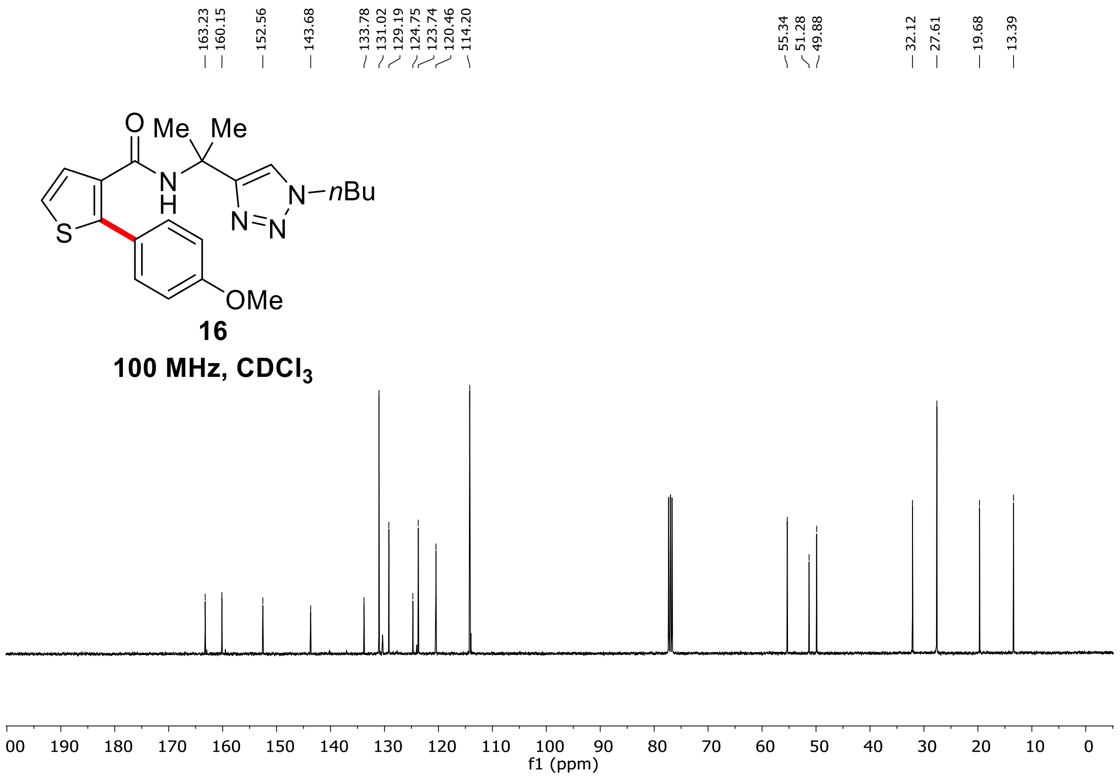
163.23
160.15
152.56
143.68
133.78
131.02
129.19
124.75
123.74
120.46
114.20
55.34
51.28
49.88
32.12
27.61
19.68
13.39
O
Me
Me
N
H
N−nBu
N=N
S
OMe
16
100 MHz, CDCl3
00 190 180 170 160 150 140 130 120 110 100 90 80 70 60 50 40 30 20 10 0
f1 (ppm)

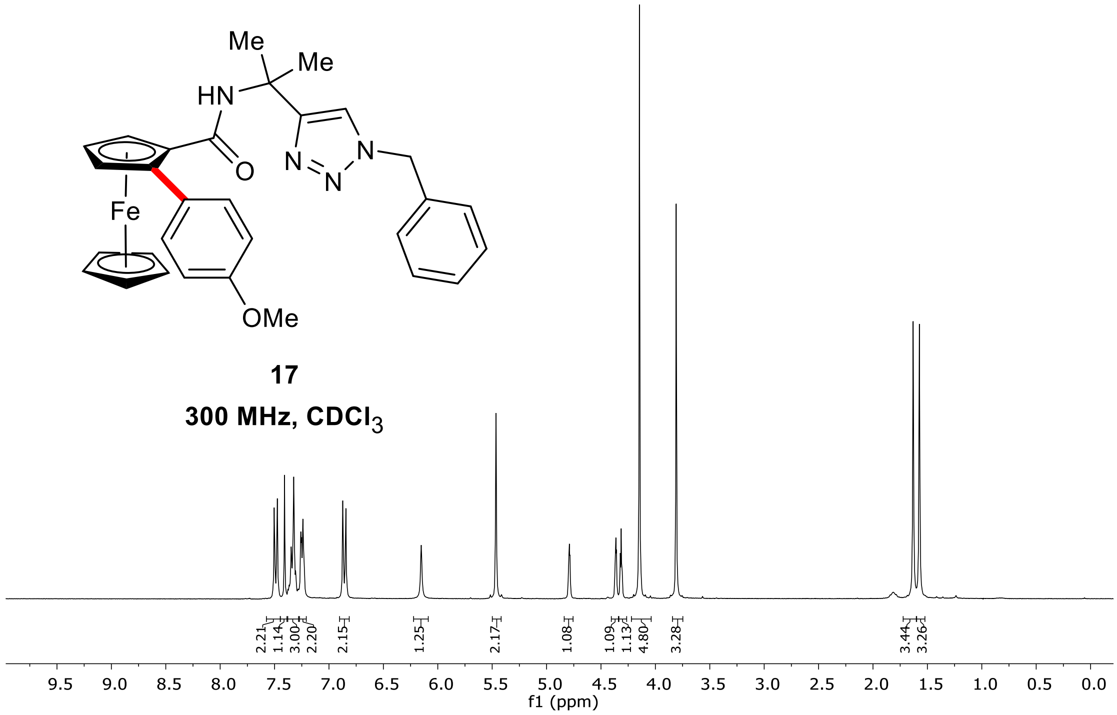
Me
Me
HN
O
N
N
N
Fe
OMe
17
300 MHz, CDCl$_3$
2.21
1.14
3.00
2.20
2.15
1.25
2.17
1.08
1.09
1.13
4.80
3.28
3.44
3.26
9.5 9.0 8.5 8.0 7.5 7.0 6.5 6.0 5.5 5.0 4.5 4.0 3.5 3.0 2.5 2.0 1.5 1.0 0.5 0.0
f1 (ppm)

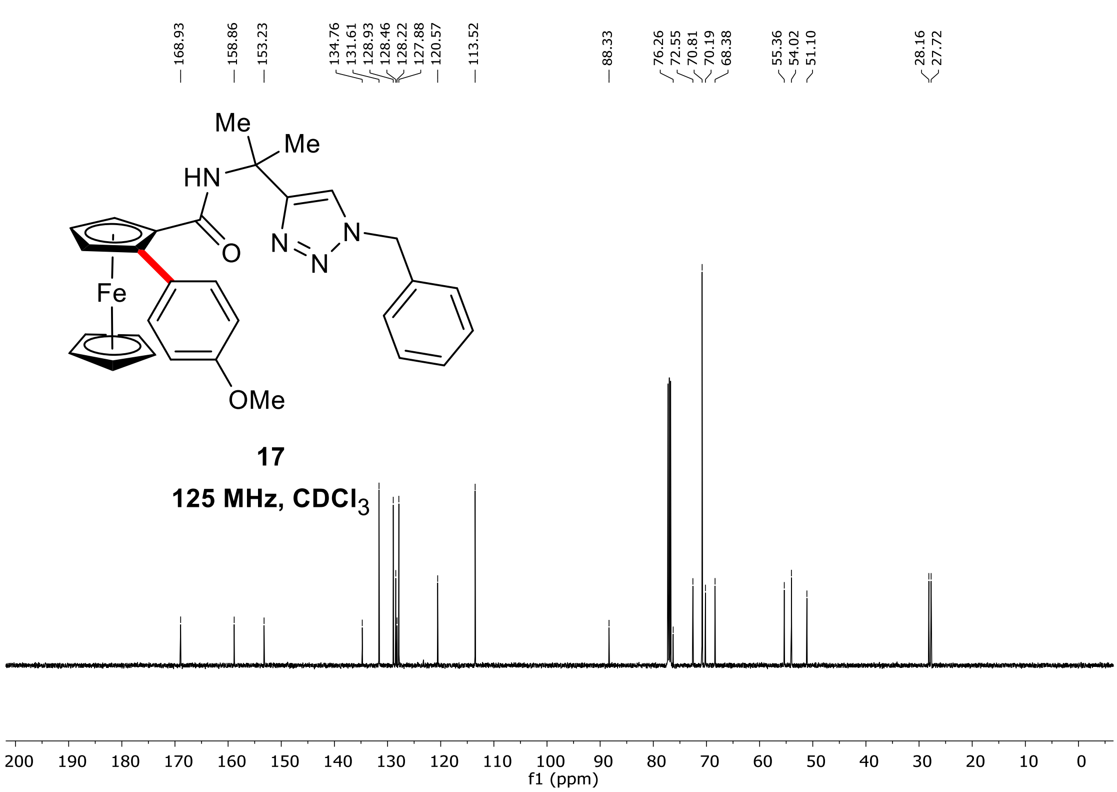
168.93
158.86
153.23
134.76
131.61
128.93
128.46
128.22
127.88
120.57
113.52
88.33
76.26
72.55
70.81
70.19
68.38
55.36
54.02
51.10
28.16
27.72
Me
Me
HN
O
N
N
N
Fe
OMe
17
125 MHz, CDCl$_3$
200 190 180 170 160 150 140 130 120 110 100 90 80 70 60 50 40 30 20 10 0
f1 (ppm)

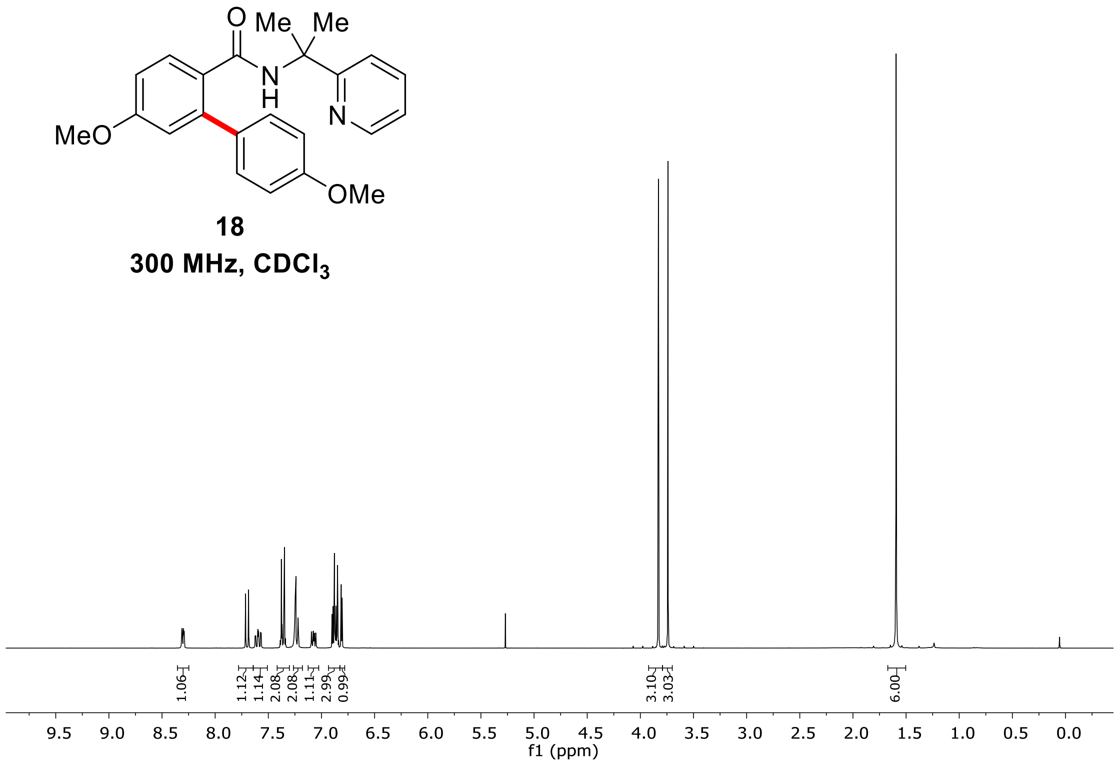

O
Me
Me
N
H
N
MeO
OMe
18
300 MHz, CDCl3
1.06
1.12
1.14
2.08
2.08
1.11
2.99
0.99
3.10
3.03
6.00
f1 (ppm)

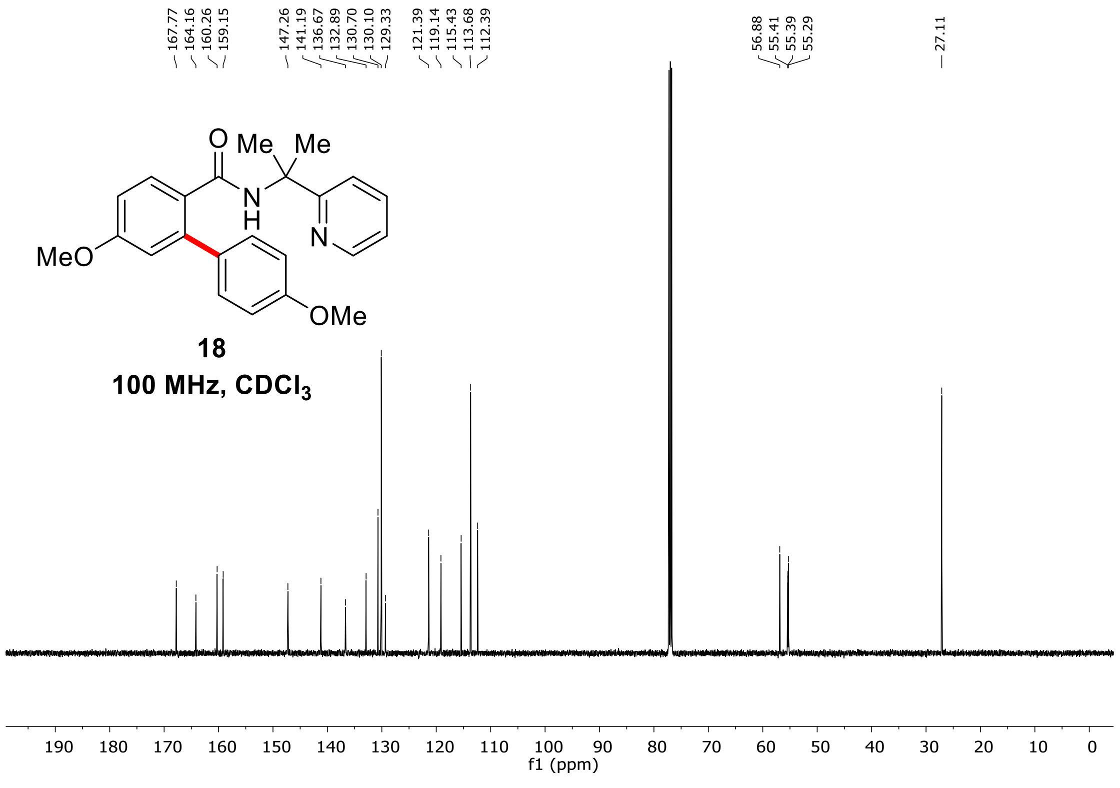

167.77
164.16
160.26
159.15
147.26
141.19
136.67
132.89
130.70
130.10
129.33
121.39
119.14
115.43
113.68
112.39
56.88
55.41
55.39
55.29
27.11
O
Me
Me
N
H
N
MeO
OMe
18
100 MHz, CDCl3
f1 (ppm)

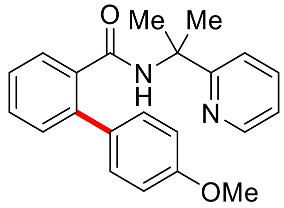

19
300 MHz, $CDCl_3$

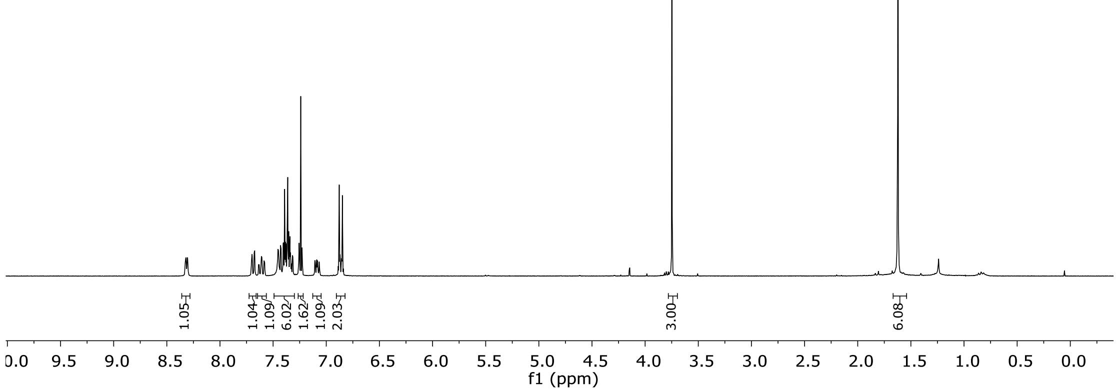

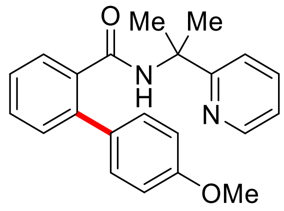

19
125 MHz, $CDCl_3$

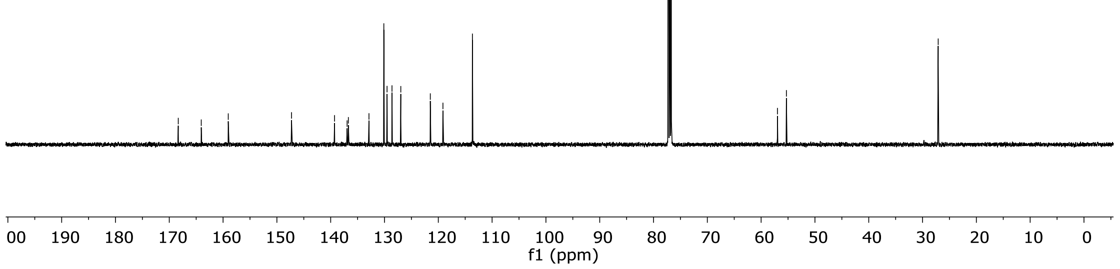

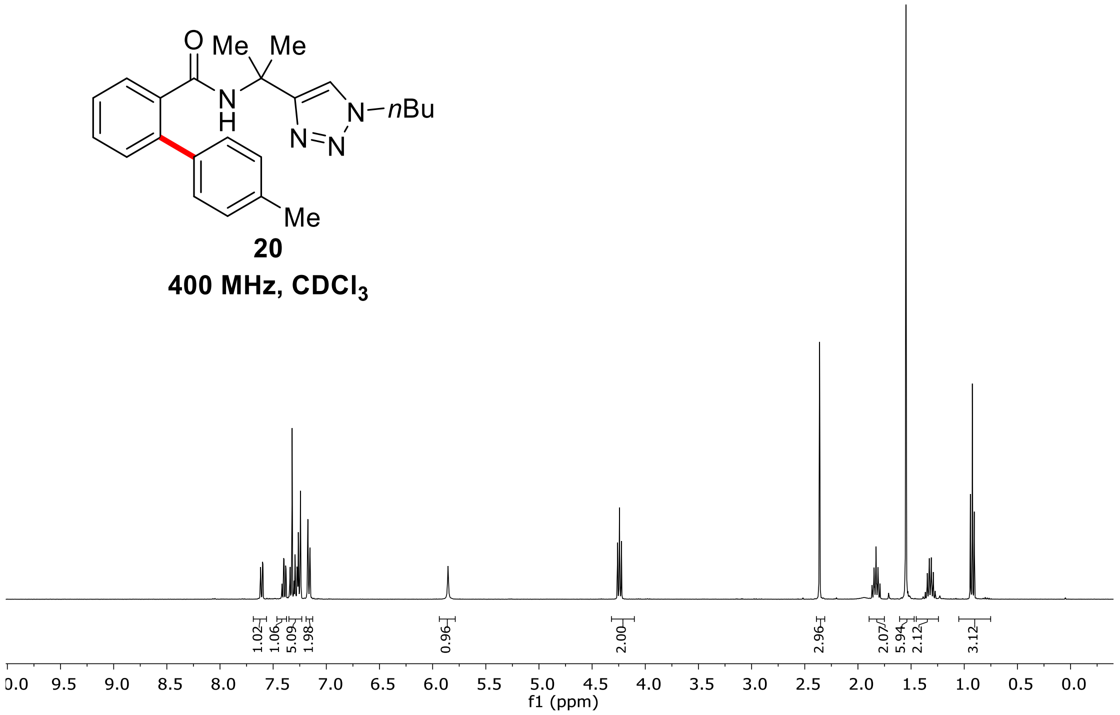
O
Me
Me
N
H
N
nBu
N
N
Me
20
400 MHz, CDCl3
1.02
1.06
5.09
1.98
0.96
2.00
2.96
2.07
5.94
2.12
3.12
10.0 9.5 9.0 8.5 8.0 7.5 7.0 6.5 6.0 5.5 5.0 4.5 4.0 3.5 3.0 2.5 2.0 1.5 1.0 0.5 0.0
f1 (ppm)

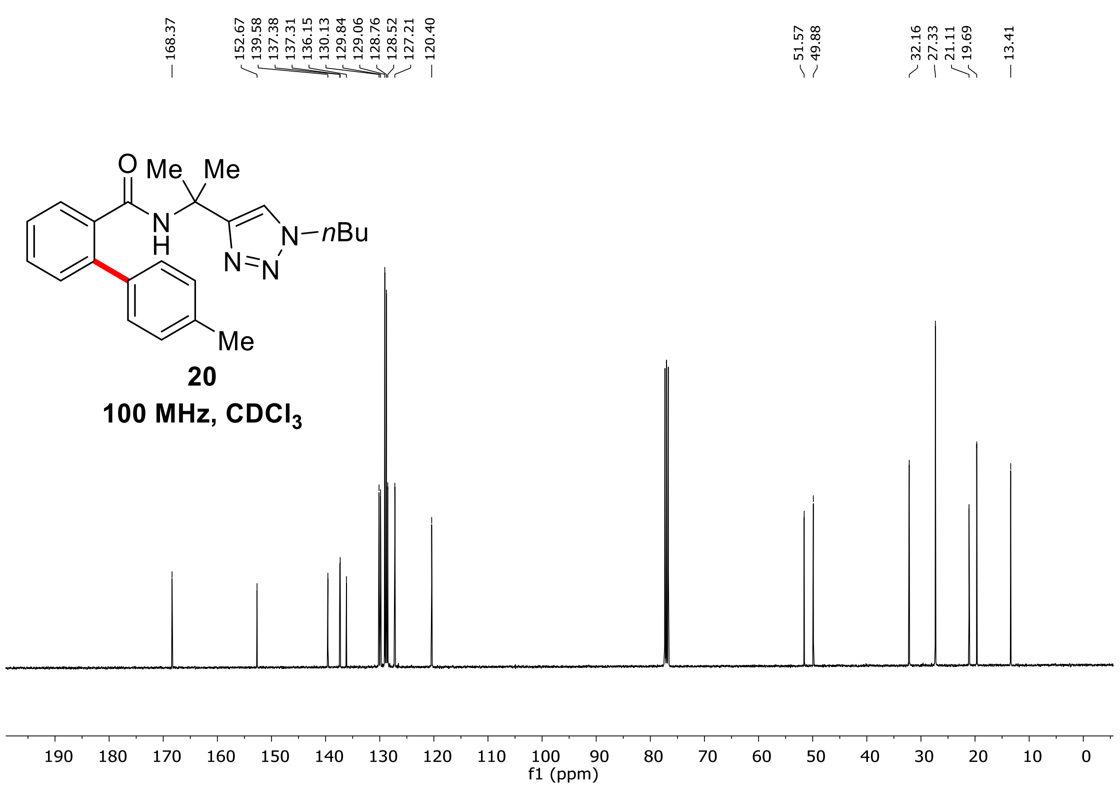
168.37
152.67
139.58
137.38
137.31
136.15
130.13
129.84
129.06
128.76
128.52
127.21
120.40
51.57
49.88
32.16
27.33
21.11
19.69
13.41
O
Me
Me
N
H
N
nBu
N
N
Me
20
100 MHz, CDCl3
190 180 170 160 150 140 130 120 110 100 90 80 70 60 50 40 30 20 10 0
f1 (ppm)

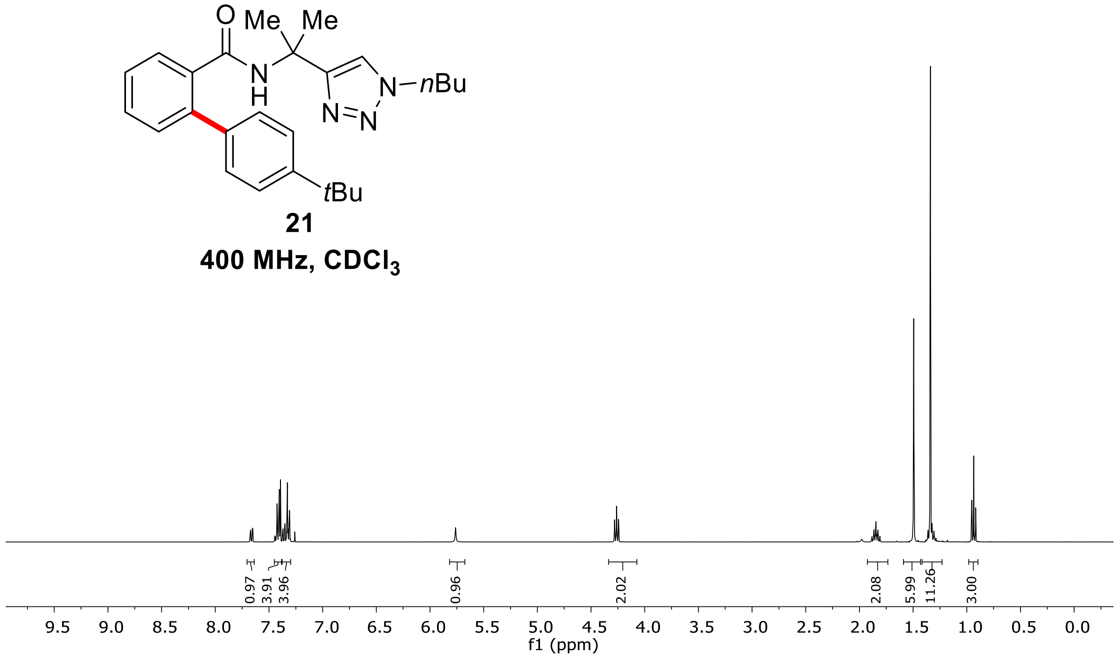
Me
Me
O
N
H
N
N
N
nBu
tBu
21
400 MHz, CDCl3
0.97
3.91
3.96
0.96
2.02
2.08
5.99
11.26
3.00
9.5 9.0 8.5 8.0 7.5 7.0 6.5 6.0 5.5 5.0 4.5 4.0 3.5 3.0 2.5 2.0 1.5 1.0 0.5 0.0
f1 (ppm)

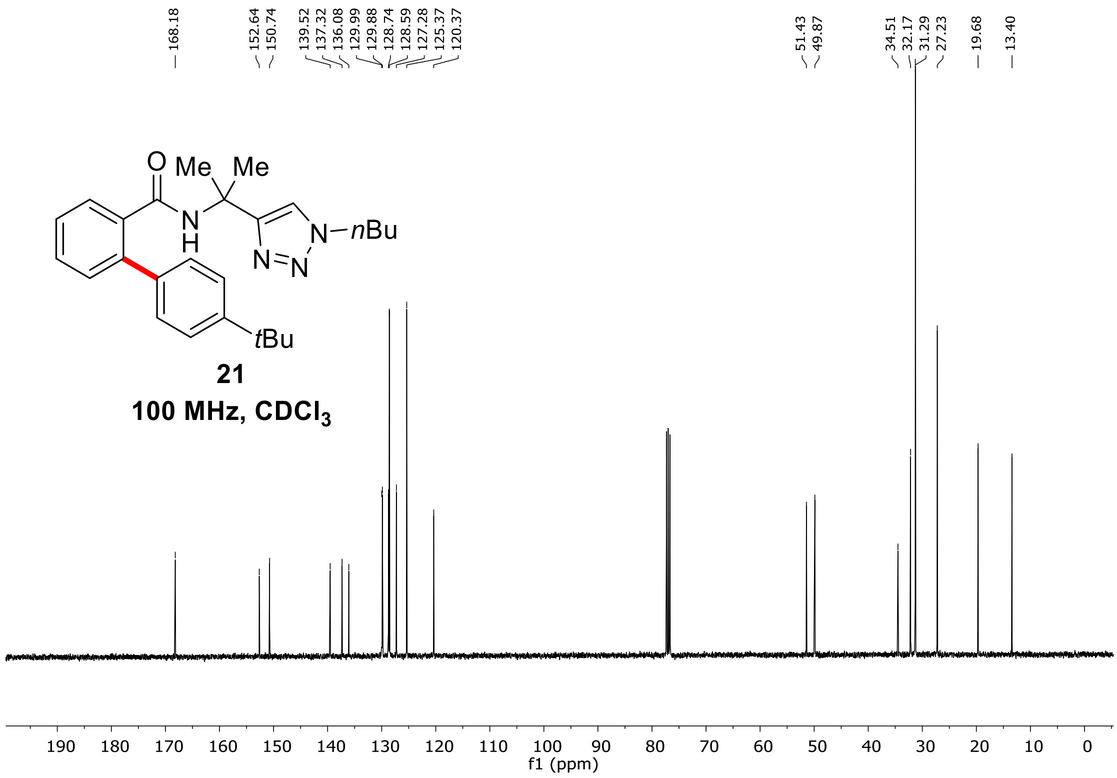
168.18
152.64
150.74
139.52
137.32
136.08
129.99
129.88
128.74
128.59
127.28
125.37
120.37
51.43
49.87
34.51
32.17
31.29
27.23
19.68
13.40
Me
Me
O
N
H
N
N
N
nBu
tBu
21
100 MHz, CDCl3
190 180 170 160 150 140 130 120 110 100 90 80 70 60 50 40 30 20 10 0
f1 (ppm)

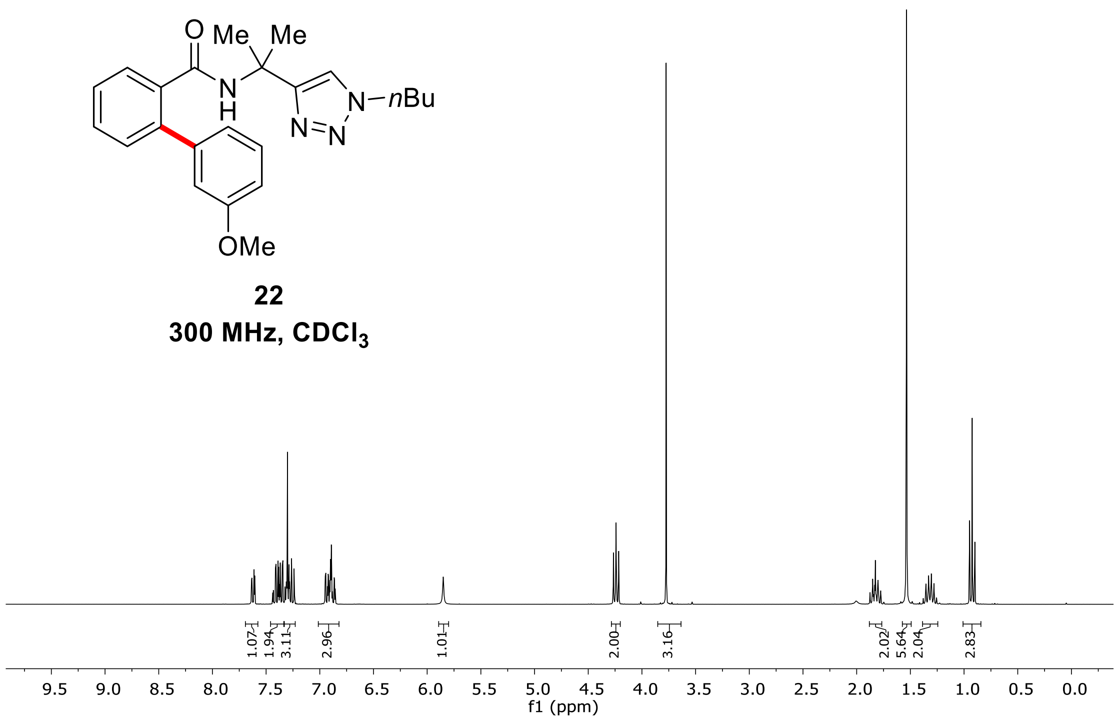
Me
Me
O
N
H
N−nBu
N=N
OMe
22
300 MHz, CDCl3
1.07
1.94
3.11
2.96
1.01
2.00
3.16
2.02
5.64
2.04
2.83
9.5 9.0 8.5 8.0 7.5 7.0 6.5 6.0 5.5 5.0 4.5 4.0 3.5 3.0 2.5 2.0 1.5 1.0 0.5 0.0
f1 (ppm)

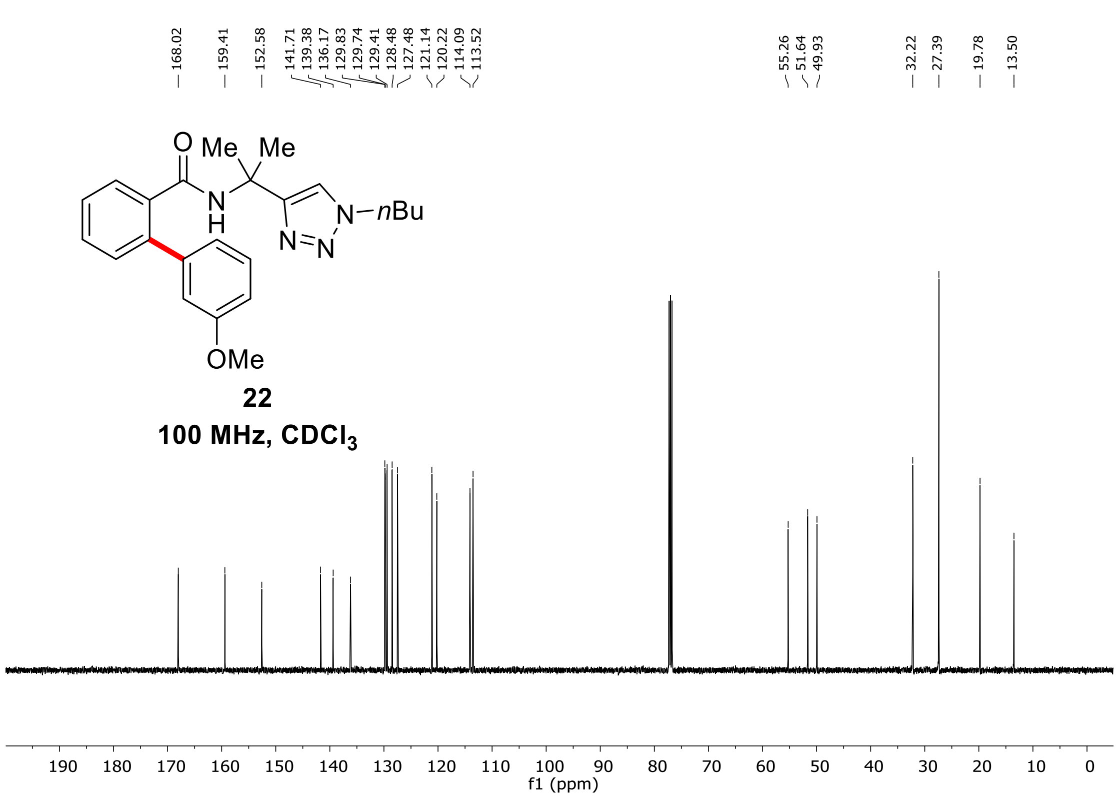
168.02
159.41
152.58
141.71
139.38
136.17
129.83
129.74
129.41
128.48
127.48
121.14
120.22
114.09
113.52
55.26
51.64
49.93
32.22
27.39
19.78
13.50
Me
Me
O
N
H
N−nBu
N=N
OMe
22
100 MHz, CDCl3
190 180 170 160 150 140 130 120 110 100 90 80 70 60 50 40 30 20 10 0
f1 (ppm)

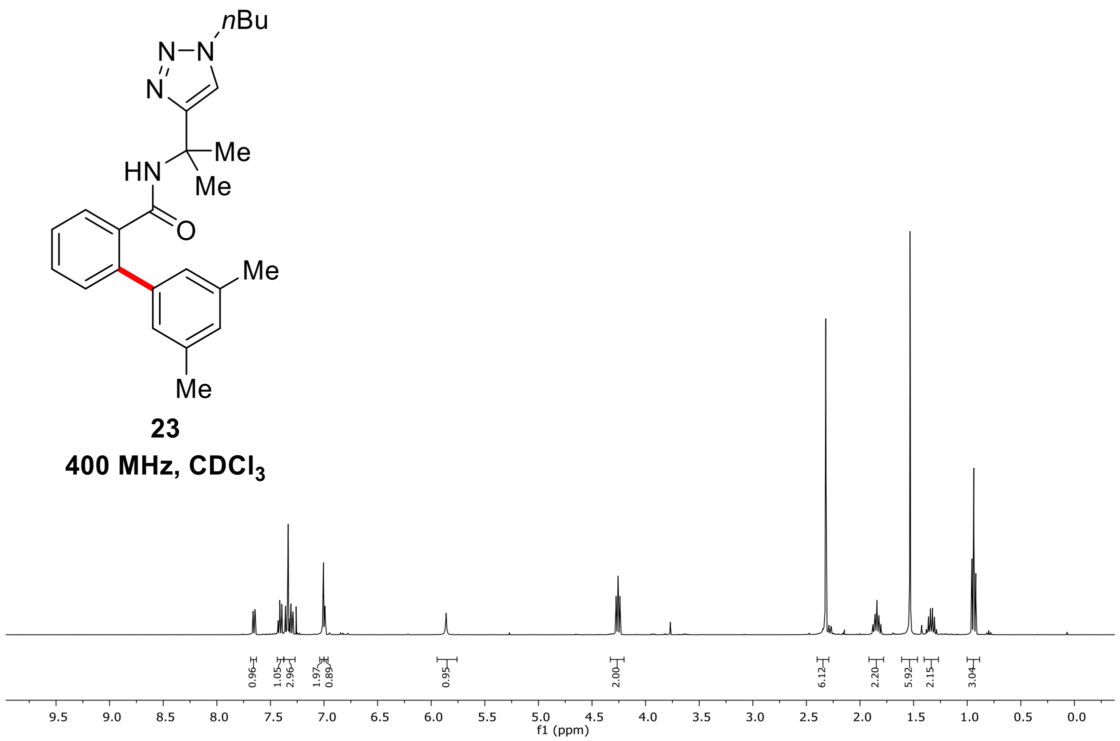

nBu
N
N
N
HN
Me
Me
O
Me
Me
23
400 MHz, CDCl3
0.96
1.05
2.96
1.97
0.89
0.95
2.00
6.12
2.20
5.92
2.15
3.04
f1 (ppm)

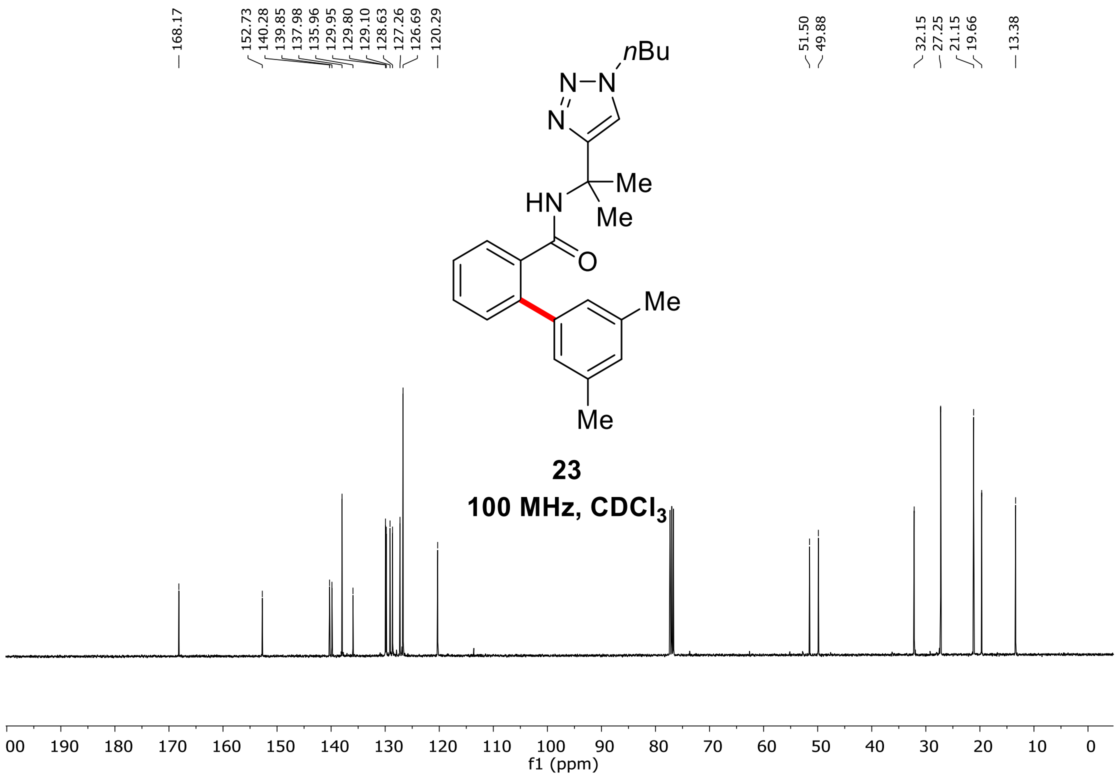

168.17
152.73
140.28
139.85
137.98
135.96
129.95
129.80
129.10
128.63
127.26
126.69
120.29
51.50
49.88
32.15
27.25
21.15
19.66
13.38
nBu
N
N
N
HN
Me
Me
O
Me
Me
23
100 MHz, CDCl3
f1 (ppm)

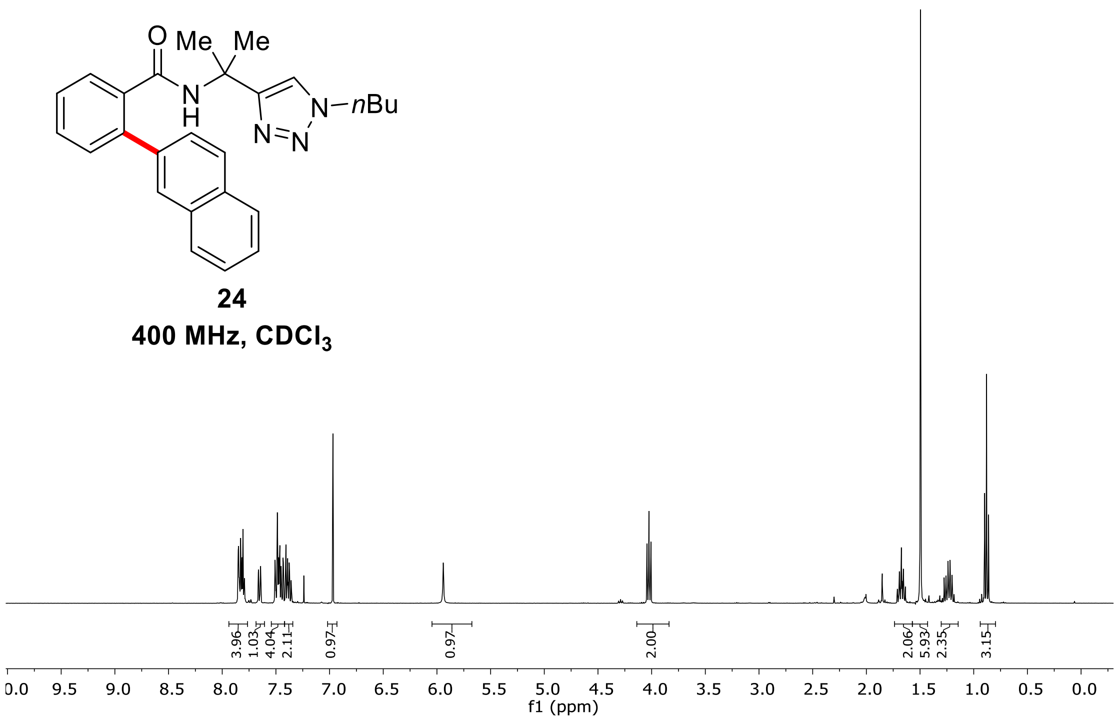
24
400 MHz, CDCl3
3.96
1.03
4.04
2.11
0.97
0.97
2.00
2.06
5.93
2.35
3.15
f1 (ppm)

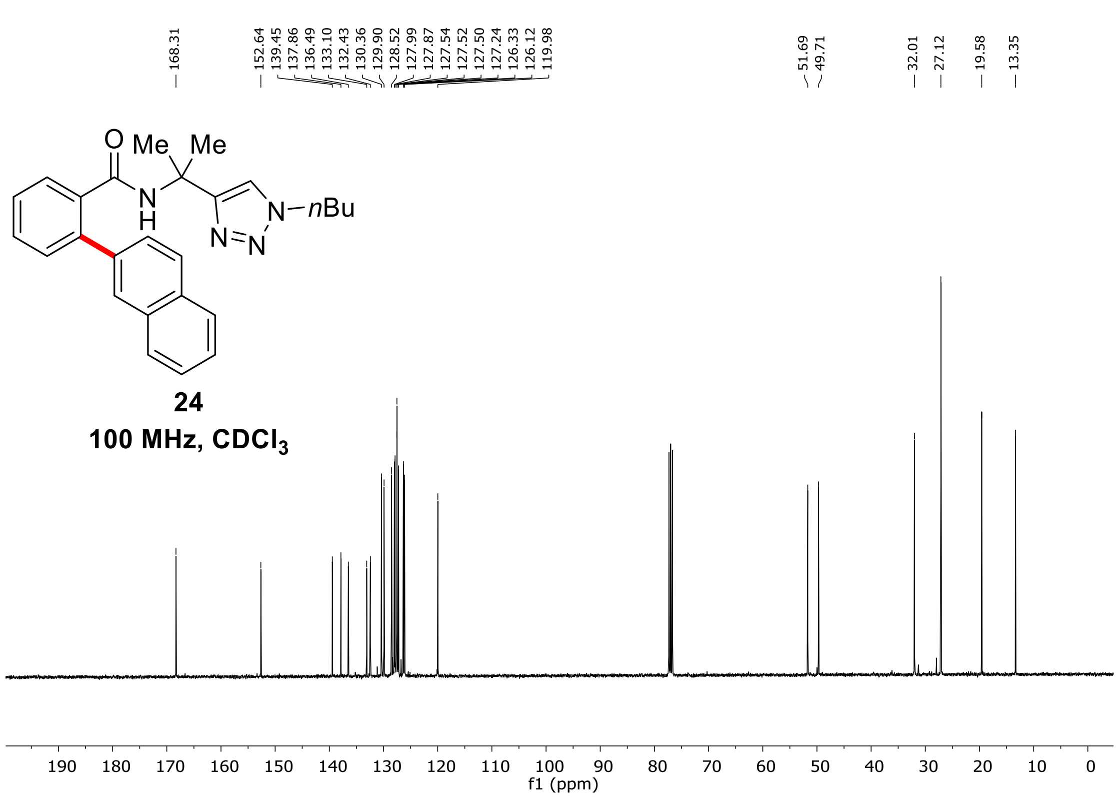
168.31
152.64
139.45
137.86
136.49
133.10
132.43
130.36
129.90
128.52
127.99
127.87
127.54
127.52
127.50
127.24
126.33
126.12
119.98
51.69
49.71
32.01
27.12
19.58
13.35
24
100 MHz, CDCl3
f1 (ppm)

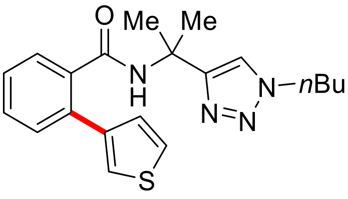

25
400 MHz, $CDCl_3$

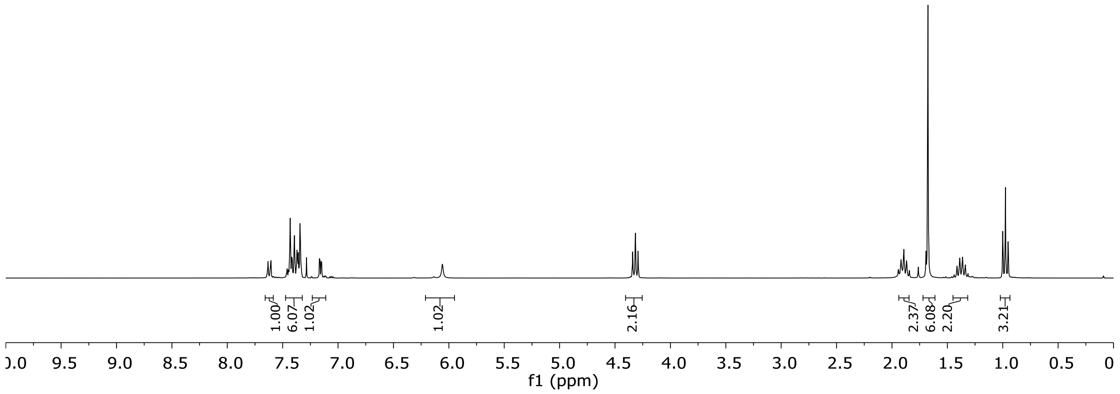

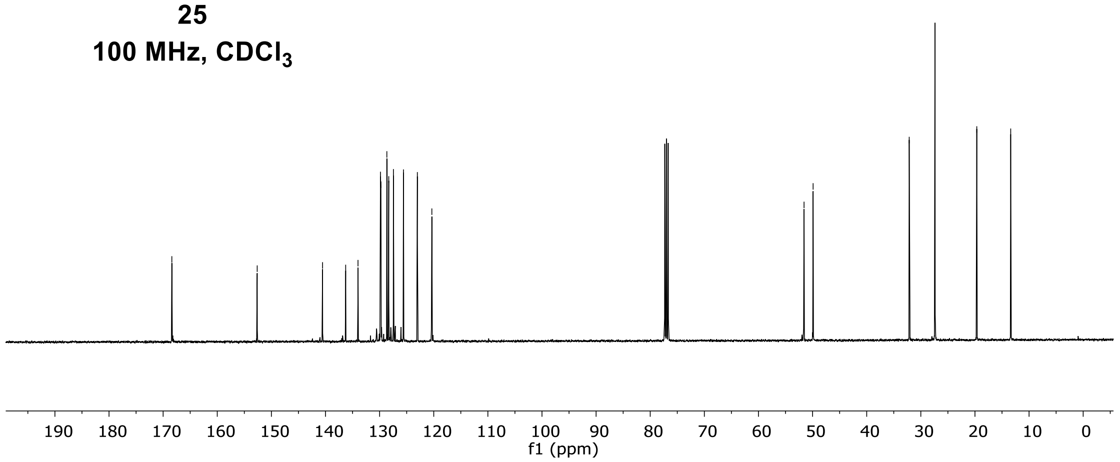

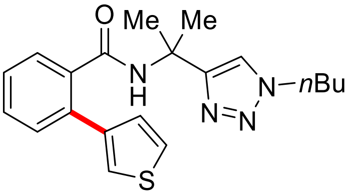

25
100 MHz, $CDCl_3$

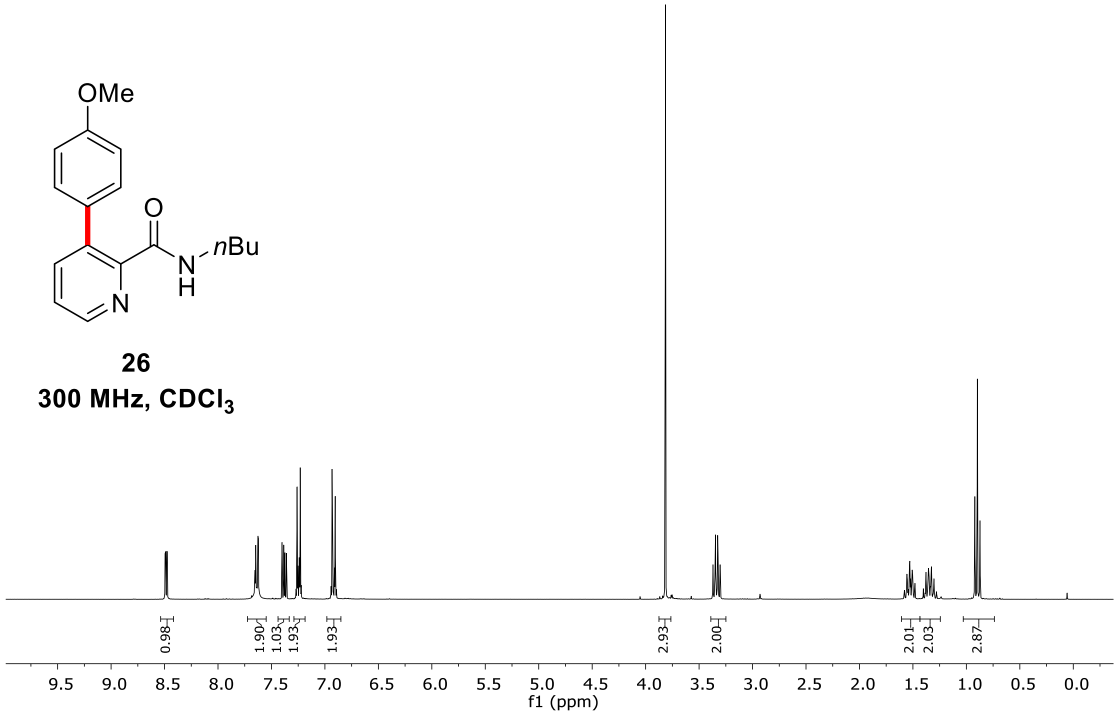
OMe
O
N
H
nBu
N
26
300 MHz, $CDCl_3$
0.98
1.90
1.03
1.93
1.93
2.93
2.00
2.01
2.03
2.87
9.5 9.0 8.5 8.0 7.5 7.0 6.5 6.0 5.5 5.0 4.5 4.0 3.5 3.0 2.5 2.0 1.5 1.0 0.5 0.0
f1 (ppm)

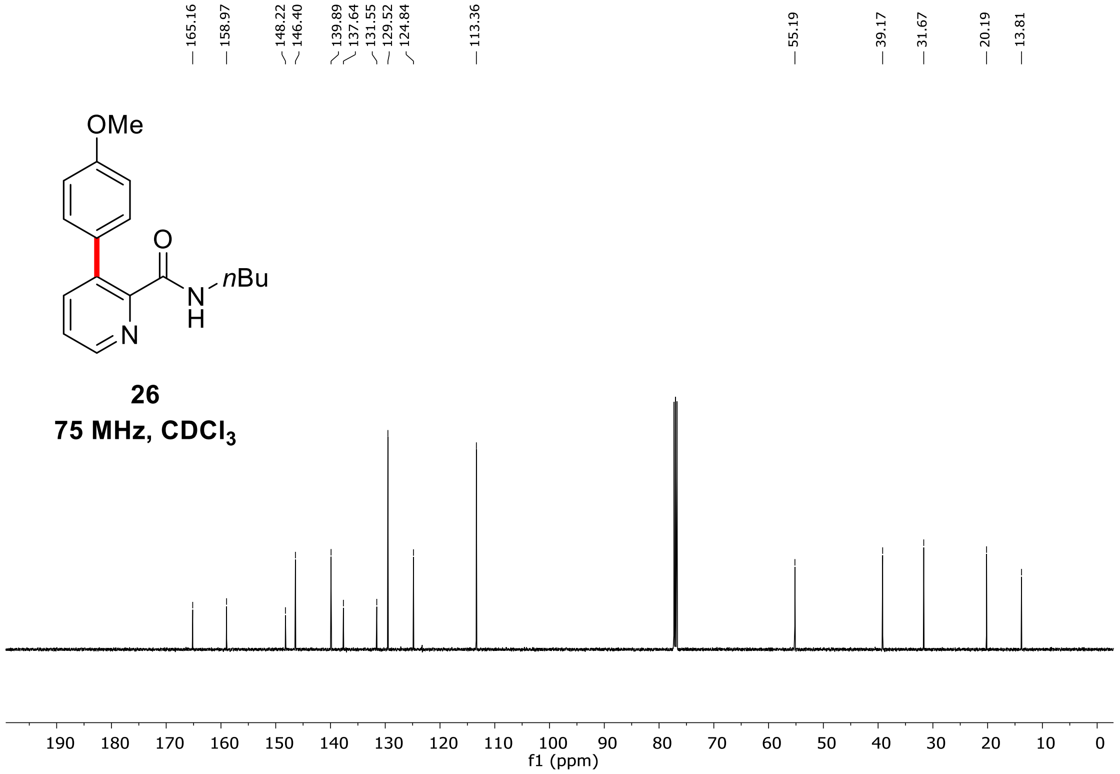
165.16
158.97
148.22
146.40
139.89
137.64
131.55
129.52
124.84
113.36
55.19
39.17
31.67
20.19
13.81
OMe
O
N
H
nBu
N
26
75 MHz, $CDCl_3$
190 180 170 160 150 140 130 120 110 100 90 80 70 60 50 40 30 20 10 0
f1 (ppm)
